# A Practical Framework for Novel Electronic Nicotine Delivery System Evaluation: Chemical and Toxicological Characterization of JUUL2 Aerosol and Comparison with Reference Cigarettes

**DOI:** 10.3390/toxics12010041

**Published:** 2024-01-04

**Authors:** David K. Cook, Guy Lalonde, Michael J. Oldham, Jiaming Wang, Austin Bates, Sifat Ullah, Christina Sulaiman, Karen Carter, Candice Jongsma, Gary Dull, I. Gene Gillman

**Affiliations:** JUUL Labs, 1000 F Street NW, Washington, DC 20004, USAmichael.oldham@juul.com (M.J.O.); sifat.ullah@juul.com (S.U.); christina.sulaiman@juul.com (C.S.);

**Keywords:** ENDS, e-cigarette, electronic cigarette, framework, chemical, toxicological, aerosol

## Abstract

Electronic nicotine delivery systems (ENDSs) are designed as a non-combustible alternative to cigarettes, aiming to deliver nicotine without the harmful byproducts of tobacco combustion. As the category evolves and new ENDS products emerge, it is important to continually assess the levels of toxicologically relevant chemicals in the aerosols and characterize any related toxicology. Herein, we present a proposed framework for characterizing novel ENDS products (i.e., devices and formulations) and determining the reduced risk potential utilizing analytical chemistry and in vitro toxicological studies with a qualitative risk assessment. To demonstrate this proposed framework, long-term stability studies (12 months) analyzing relevant toxicant emissions from six formulations of a next-generation product, JUUL2, were conducted and compared to reference combustible cigarette (CC) smoke under both non-intense and intense puffing regimes. In addition, in vitro cytotoxicity, mutagenicity, and genotoxicity assays were conducted on aerosol and smoke condensates. In all samples, relevant toxicants under both non-intense and intense puffing regimes were substantially lower than those observed in reference CC smoke. Furthermore, neither cytotoxicity, mutagenicity, nor genotoxicity was observed in aerosol condensates generated under both intense and non-intense puffing regimes, in contrast to results observed for reference cigarettes. Following the proposed framework, the results demonstrate that the ENDS products studied in this work generate significantly lower levels of toxicants relative to reference cigarettes and were not cytotoxic, mutagenic, or genotoxic under these in vitro assay conditions.

## 1. Introduction

Combustible cigarettes (CCs) are the number one cause of preventable death in the world [1]. Researchers, therefore, have worked for decades to characterize CCs and the harmfulness of smoking [2]. Resultantly, countries or regions (e.g., the European Union) have passed laws providing regulatory agencies with the authority to enact regulations and programs that mandate reporting and reducing harmful CC smoke constituents in the interest of protecting public health [3,4,5,6,7,8,9].

Electronic nicotine delivery systems (ENDSs), also known as e-cigarettes, have risen in popularity over the past 15 years as an alternative to CCs, and therefore, regulatory authorities have a concomitant interest in evaluating the potential toxicity of ENDS emissions relative to CC smoking. The harmful or potentially harmful constituents (HPHCs) in CC smoke are well documented to be linked to a number of tobacco-related diseases such as cancer and cardiovascular and respiratory diseases [1]. In contrast to cigarettes, ENDSs have been designed under the concept of delivering nicotine without combustion, as the byproducts of tobacco combustion, rather than nicotine itself, have been shown to be responsible for smoking-related diseases (cancer, cardiovascular and respiratory diseases, and reproductive effects) [10,11,12].

As a technology, ENDSs most often contain a battery, a heating element (comprising a wick and coil), and a nicotine-containing solution, commonly known as e-liquid [13]. The heating of the e-liquid produces an aerosol that is then inhaled by the consumer. Product characteristics (i.e., coil temperature, wicking, etc.) and formulation composition (i.e., ingredients, ingredient amount, etc.) can impact both chemical composition and biological activity [14,15,16]. Unlike tobacco smoke, which is a highly complex mixture (>5000 constituents), ENDS aerosols have been reported to exhibit significant reductions in both the number of measurable analytes and their abundance due to the absence of tobacco leaf and combustion byproducts [2,17,18,19,20,21]. A growing body of literature on ENDS products reports lower levels of specific aerosol toxicants such as tobacco-specific nitrosamines, carbonyls, and volatile organic compounds [13,15,22,23,24,25,26,27,28,29,30,31,32,33]. Correspondingly, depending on the device and formulation, e-liquids and ENDS aerosols have exhibited lower in vitro and in vivo biological activity as compared to CCs [16,34,35,36,37,38,39,40,41,42].

As new products continue to emerge with variations in device design, performance parameters, and formulation composition, regulatory agencies continue to advance science-based regulatory frameworks. Within the U.S., in 2016, a Deeming Rule was finalized granting the U.S. Food and Drug Administration (US FDA) the authority to regulate ENDSs [43]. In 2019, the US FDA announced a comprehensive plan outlining a continuum of risk for nicotine products, with CCs at the highest end of the spectrum and nicotine replacement therapies at the lowest end of the spectrum [44]. Later in 2019, the US FDA issued a proposed rule and further published industry guidance on obtaining market authorization for ENDS products, which included a selection of relevant HPHCs and other chemicals to measure in ENDS aerosol [45,46,47,48]. Globally, government authorities and standardization bodies such as the European Committee for Standardization (CEN), European Union (EU), British Standard Institute (BSI), French Standardization Association (AFNOR), Health Canada (HC), and National Standard of the Peoples Republic of China have established or adopted ENDS-pertinent regulations relative to product manufacturing, e-liquid constituents, emission characterization, or constituent limits [7,8,9,49,50,51,52,53,54,55,56,57,58,59,60,61,62]. To assist in compliance with the growing body of standards and established regulatory frameworks and to support the evaluation of potential health-related claims, we propose in this paper a simplified framework to guide manufacturers on the characterization and assessment of new/modified products before introduction to the market. While other product evaluation frameworks have been proposed, these frameworks may include recommendations such as clinical trials, long-term surveillance, and population modeling studies or involve comprehensive systems toxicology assessments [63,64,65,66]. These frameworks add valuable analyses but are less suited for early assessments of novel tobacco products and do not emphasize the thorough chemical analysis required for their evaluation.

Importantly, the recommendations presented within this manuscript are not intended as a substitute for the legal standards that regulatory agencies are authorized or required to apply.

## 2. A Proposed Framework: Chemical and Toxicological Evaluation

**Figure 1:**
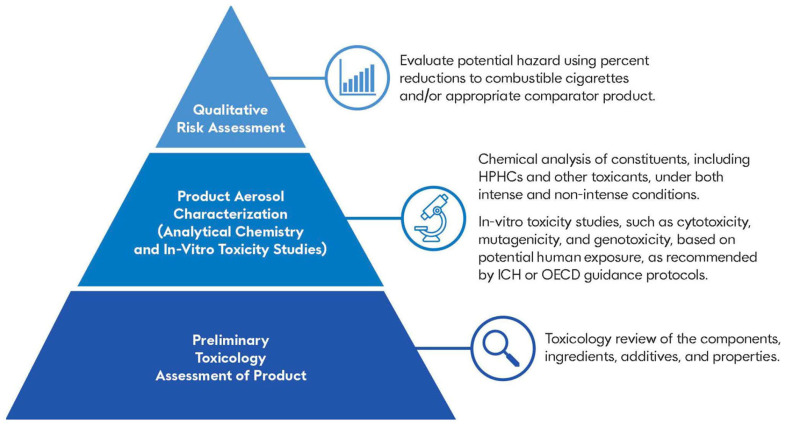
Chemical and toxicological evaluation framework.

There are three primary aspects of novel ENDS product evaluation, the e-liquid, the device (battery and pod components), and the aerosolized emissions. Presented in Figure 1, this framework proposes a three-step process to cover all aspects within this evaluation: (1) a preliminary toxicological assessment of product components, ingredients, additives, and aerosol properties; (2) characterization of the product aerosol via analytical chemistry and in vitro toxicity studies; and (3) qualitative risk assessment against CC smoke (or relevant comparator product) to evaluate the harm reduction potential of the novel ENDS product [46]. Fundamentally, the primary objective of this framework is to provide a practical approach to novel ENDS product characterization followed by a qualitative risk assessment for the determination of reduced risk potential. This paper applies the proposed framework to six test articles as a case study providing insights into how to conduct chemical and toxicological studies of this kind.

Prior to the chemical and toxicological evaluation of aerosols, the framework begins with a toxicological review of the novel ENDS device componentry/properties with a focus on components in the container closure system, components in the aerosol path, and toxicological evaluation of all ingredients used to make the e-liquid(s). The toxicological work on the ENDS product is usually performed in two parts. The first involves screening early in product development designed to avoid potentially carcinogenic, cytotoxic, mutagenic, reprotoxic, sensitizing, or inflammatory materials or additives that reduces the likelihood of potential downstream toxicological findings [64,67]. The second involves extractable and leachable studies on the container closure system and parts in the airpath. This evaluation could be similar to what is performed on medical devices or combination device/drug products [68,69,70,71,72,73,74,75,76,77]. Although depicted in Figure 1 as occurring prior to the chemical and toxicological evaluation of the product aerosol, these studies may be performed in parallel.

Some countries have proposed or finalized lists of allowed or prohibited ingredients for ENDS products (e.g., Canada, EU, China) [7,9,60]. Prohibited ingredients typically include those that are recognized as carcinogenic, mutagenic, or reproductive and developmental toxicants. Once regulatory requirements are satisfied, the preliminary toxicological assessment of ingredients uses well-established quantitative risk assessment techniques to evaluate the individual health risk of constituents from inhalation exposure [78,79,80,81,82,83]. This approach is consistent with the recent adoption of CEN EN 17648:2022 within the EU and builds upon a previously published approach to e-liquid risk assessment [55,67]. A key requirement of the ingredient quantitative risk assessment is to have an estimated daily product usage, which may change during product development.

In product aerosol characterization, differences in design and engineering play an important role in the variability of total aerosol yield, the level of aerosol constituents, and nicotine dose [15,84,85,86]. Differences in aerosol composition may result in key differences in toxicant reduction between ENDS products as compared to CCs [14,26,84]. Performing in vitro studies to assess cytotoxicity, mutagenicity, and genotoxicity provides information on key toxicological endpoints relevant to noncancer and cancer adverse effects associated with tobacco product use, and therefore, these studies are informative of the potential toxicity of new ENDS products. Concluding the evaluation, a qualitative risk assessment is performed on the chemical and toxicological studies with the focus of these assessments to establish that, compared to CCs or appropriate comparator products, these products have no new or increased hazards [87].

### Case Study Background: Characterization of JUUL2 System with Six Novel Formulations

The JUUL2 product launched in the U.K. in September 2021 and is a temperature-regulated ENDS that consists of a closed (1.2 mL capacity) pod and technologically advanced device (i.e., smartphone-compatible with device locking, tracking, and usage analytic features). Utilizing the JUUL2 System, six pre-filled e-liquid formulations at an 18 mg/mL nicotine concentration were chosen for evaluation: Virginia Tobacco (VT), Crisp Menthol (CM), Polar Menthol (PM), Autumn Tobacco (AT), Ruby Menthol (RM), and Summer Menthol (SM). Following the proposed framework, the device componentry and ingredient disclosures were toxicologically assessed, and products were cleared for further chemical and toxicological analyses.

## 3. Materials and Methods

### 3.1. Targeted Analytical Chemistry Stability Study

For the analysis of chemical constituent levels in ENDS aerosol, stability studies were conducted in general accordance with relevant regulatory frameworks [9,46] and guidance documents [88,89]. One, commercially representative, pre-production lot of each formulation was manufactured, shipped, and placed in stability for 12 months with analytical evaluation performed at 0, 3, 6, 9, and 12 months under long-term (LT) storage conditions (25 ± 2 °C/60% RH ± 5%).

To determine selected aerosol constituents for evaluation, U.S., U.K., Asian, EU, and European Member State guidance documents were considered. A total of fifty-four aerosol constituents were measured across ten analytical methodologies (Table 1). Based on chemical classification, measured constituents were grouped into the following categories for discussion and interpretation: (1) Primary Constituents, (2) Carbonyls, (3) Glycidol, (4) Nicotine Degradants, (5) Tobacco-Specific Nitrosamines (TSNAs), (6) Metals, (7) Organic Acids, (8) Volatile Organic Compounds (VOCs), (9) Esters and Alcohols. In addition, aerosol (10) pH was also measured. Five replicates (*n* = 5) were utilized per analytical assay, per time point evaluation.

Aerosol constituent analysis was performed by Enthalpy Analytical, LLC (Durham, NC, USA; Richmond, VA, USA). All analytical methods were validated for the analysis of ENDS aerosol according to International Council for Harmonisation (ICH) guidance Q2 (R1) [90]. All analytical method validations were reviewed by an independent accreditation body as part of their International Organization for Standardization (ISO) 17025 accreditation process; for more information, see the Supplementary Materials (File S1) [91].

For the collection of ENDS aerosol, whole-pod collections, determined from end-of-life (EOL) determination experiments, were utilized following the approach outlined by Jameson et al. (2023) [92]. Batteries were charged before use and changed after each collection. Two collection regimes were used for the JUUL2 System (e.g., pod and device), the puffing regime described in ISO 20768:2018 (non-intense (NI)) of a 55 mL puff volume, 3 s puff duration, and 30 s inter-puff interval (55/3/30) [93], and an intense (INT) puffing regime of a 110 mL puff volume, 6 s puff duration, and 30 s inter-puff interval (110/6/30). Both puffing regimes utilized a square wave puffing profile. For the JUUL2 products, puff counts utilized in targeted analyses ranged from 100 to 150 puffs under non-intense puffing and 68 to 90 puffs under intense puffing with an average cumulative DML (device mass loss) target of 1085 mg per collection.

Aerosols were collected utilizing a linear 20-port e-cigarette puffing machine (Cerulean SM450e, Milton Keynes, U.K.); see the Supplementary Materials for an example of the collection apparatus (File S1, Figure S1). Primary Constituents, Nicotine Degradants, and TSNAs methodologies trapped aerosol on a conditioned 55 mm glass fiber filter pad (GFFP). Pads were placed in a vial containing extraction solution and agitated for analysis. Organic Acids, Glycidol, and pH methodologies effervesced aerosols through one or more impingers containing trapping solution followed by sample clean-up or derivatization. For Carbonyls, Esters, and Alcohols, an in-tandem (conditioned) GFFP and impinger setup was utilized. Pads were mixed with impinger trapping solution followed by agitation, sample clean-up, or derivatization. For VOCs, a sorbent tube was utilized to trap aerosols; tubes were removed, added to an extraction vial containing trapping solution, agitated, and aliquoted for analysis. For Metals analyses, to minimize potential background contamination introduced from the collection setup, samples were collected using an SM450e equipped with a Halder Process Solutions HPS-EP5 electrostatic precipitator (EP) system (Halder Process Solutions, Moseley, VA, USA). This system includes a quartz glass tube equipped with a tungsten electrode whereby high voltage is applied between the tube and electrode causing aerosol particles to acquire charge and deposit on the tube walls. Tubes were subsequently rinsed with semiconductor-grade methanol to extract and digest the collected aerosol for analysis.

Select methodologies and constituents such as VOCs, TSNAs, Esters, and Alcohols were measured at initial timepoints and removed from subsequent analysis based on a lack of presence, minimal detection levels, or the unlikelihood of formation within the aerosol. For analysis and interpretation, all values were reported on a per-collection basis (also referenced as “whole-pod collection”). These results were compared to combustible reference cigarettes smoked under non-intense (35:2:60, ISO 3308:2012) and intense (55:2:30 ISO 20778:2018) puffing regimes compiled from published literature or internally sponsored studies [94,95]. See the Supplementary Materials (File S1) for additional method and comparison details.

### 3.2. In Vitro Toxicity Assessment

The in vitro toxicological evaluation was based upon the testing recommended for tobacco products by the Cooperation Center for Scientific Research Relative to Tobacco (CORESTA) [96]. Accordingly, three established toxicological assays, testing for cytotoxicity, mutagenicity, and mammalian genotoxicity, were performed using ENDS aerosol and CC smoke condensates (Section 3.3) in accordance with the standardized Organization for Economic Co-operation and Development (OECD) guidelines. All assays were also performed in accordance with U.S. FDA Good Laboratory Practice (GLP) regulations. The neutral red uptake (NRU) assay was performed using murine 3T3 and human A549 cells to evaluate the potential cytotoxicity of ENDS product aerosols and reference cigarette (3R4F) smoke in accordance with the OECD 129 guidance [97]. The ISO 10993-5 criterion of 70% cell viability was used to determine cytotoxicity [98]. Mutagenicity was measured in the TA98, TA100, TA102, TA1535, and TA1537 strains of Salmonella typhimurium using the reverse mutation (Ames) assay with preincubation in accordance with the OECD 471 guidance, and genotoxicity was evaluated in human TK6 cells using the in vitro micronucleus (MN) assay in accordance with the OECD 478 guidance [99,100,101]. Details have been previously published and are also provided in the Supplementary Materials (File S6) [102].

### 3.3. Aerosol and Smoke Generation and Chemical Characterization of Condensates Prepared for Toxicological Assays

Condensate production from JUUL2 ENDS pods was initiated alongside T = 0 month targeted analytical chemical stability studies and was performed using the same two puffing regimens and in the same manner as used for the stability studies. Each condensate was collected from a single lot of JUUL2 pods. Condensates were generated by collecting JUUL2 System aerosol on a non-conditioned 55 mm GFFP, connected in series to a glass impinger containing 20 mL of United States Pharmacopeia (USP)-grade ethanol, chilled in an ice bath (~0 °C). Batteries were charged before use and changed after each 50-puff collection until the pod EOL puff count was reached. Aerosol was collected across multiple pods until a target 1200 ± 100 mg aerosol collected mass (ACM) was reached on the GFFP. Upon completion of the aerosol collection, the GFFP was extracted in the contents of the impinger, and the mixture was filtered through gauze to produce the final condensate sample which was aliquoted in vials and stored frozen (<−65 °C) pending chemical and biological analysis.

The 3R4F reference cigarettes were obtained by Enthalpy Analytical and originally sourced from the University of Kentucky. Conditioning of the reference cigarettes conformed to ISO 3402 [103]. Smoke was produced with a rotary smoking machine (Borgwalt RM20, Hamburg, Germany) using the ISO 20778 smoking regime (55 mL puff volume, 2 s puff duration, 30 s interval with 100% ventilation holes blocked) [95]. Mainstream cigarette smoke was collected using a 92 mm GFFP connected in a series to an impinger as described above and stored frozen (<−65 °C) pending chemical and biological analysis.

To demonstrate the stability of the collected condensates during in vitro testing, the concentrations of primary constituents were analyzed at 0, 4, and 8 weeks (5 replicates) after ENDS aerosol condensate collection; see the Supplementary Materials (File S6) [102]. The chemical analysis methods were the same as those used in the targeted analytical chemistry stability studies and were performed by the same contract research organization.

## 4. Results

### 4.1. Chemical Evaluation

The complete list of JUUL2 aerosol quantitative chemistry stability results, applied calculations, source literature, and analytical CC values can be found in the Supplementary Materials (Files S1–S5).

#### 4.1.1. Aerosol Chemical Characterization

Following the proposed framework, over the 12-month study, quantifiable analyte stability trends were evaluated and whole-pod collection results were normalized on a mg/mg nicotine basis for CC comparison. Aerosols generated from the six JUUL2 formulations (VT, CM, PM, AT, RM, SM) were analyzed for fifty-four (54) measured constituents, including HPHCs (established and proposed) under non-intense and intense puffing regimes. The majority (34 out of 54) of the measured constituents were either below the limit of quantification (LOQ) or below the limit of detection (LOD) in the JUUL2 System aerosols. Twenty (20) constituents were present at levels greater than the LOQ: nicotine, glycerol, propylene glycol, water, menthol (in mentholated formulations), benzoic acid, acetaldehyde, acrolein, formaldehyde, glycidol, β-nicotyrine, cotinine, myosmine, nicotine-n-oxide, nornicotine, NNN, chromium, iron, selenium, and ethyl acetate; see the Supplementary Materials (Files S2–S5).

#### 4.1.2. Comparison to Combustible Cigarettes

The JUUL2 device is designed for adult smokers of combustible tobacco products, and available data indicate that smokers switch to these products when used as intended [104,105]. Therefore, an HPHC and chemical comparison to combusted cigarettes as a comparator product was deemed appropriate for the evaluation of changes in potential user exposures and subsequent changes in health risks associated with switching from combusted cigarettes to the JUUL2 System. Due to product and collection differences, aerosol and smoke comparisons were conducted on a per mg nicotine basis. The maximum nicotine-normalized value across T0-T12 was utilized for JUUL2 aerosol comparison. For the CC smoke, available published literature and internally conducted studies on 1R6F and 3R4F Kentucky reference cigarettes were utilized; see the Supplementary Materials (File S4).

Comparing ISO non-intense [94] CC smoke to ISO non-intense [93] JUUL2 aerosol, thirty-three (33) HPHC and chemical comparisons were evaluated. Excluded comparator constituents (21) include myosmine, arsenic, beryllium, cobalt, gold, iron, selenium, silver, tin, zinc, benzoic acid, propionic acid, 1,3, butadiene, isoprene, 1-butanol, benzyl acetate, ethyl acetate, ethyl acetoacetate, isoamyl acetate, isobutyl acetate, methyl acetate. Comparing ISO intense [95] CC smoke to intense JUUL2 aerosol, thirty-four (34) HPHC and chemical comparisons were evaluated. Excluded comparator constituents (20) include arsenic, beryllium, cobalt, gold, iron, selenium, silver, tin, zinc, benzoic acid, propionic acid, 1,3 butadiene, isoprene, 1-butanol, benzyl acetate, ethyl acetate, ethyl acetoacetate, isoamyl acetate, isobutyl acetate, methyl acetate. For all product (i.e., device and formulation)-specific comparisons between JUUL2 aerosol and CC smoke, see the Supplementary Materials (File S5).

**Figure 2:**
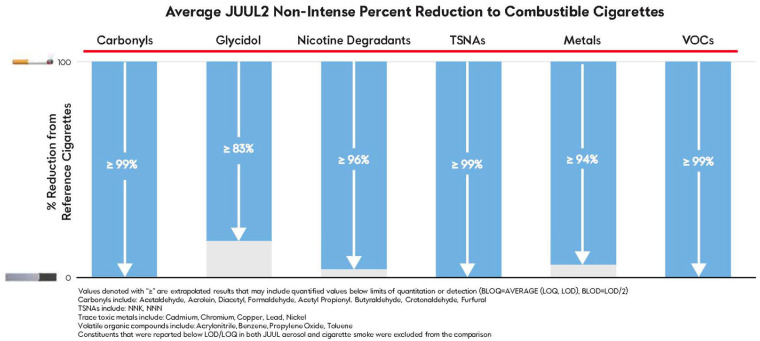
Percent reduction comparison of mean JUUL2 pod aerosol constituent aerosol levels and mean combustible cigarette smoke constituent levels under non-intense puffing regimes.

**Figure 3:**
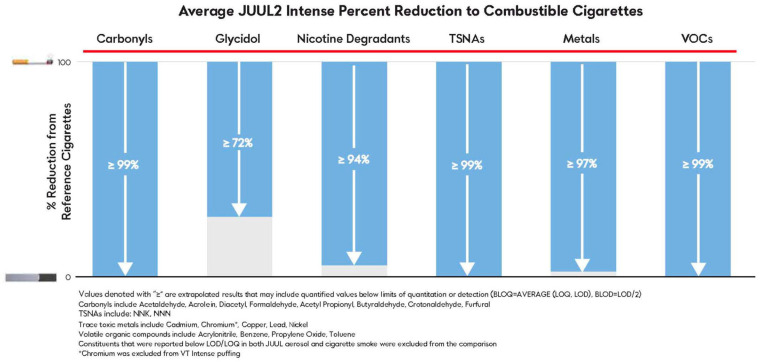
Percent reduction comparison of mean JUUL2 pod aerosol constituent aerosol levels and mean combustible cigarette smoke constituent levels under intense puffing regimes.

Figure 2 and Figure 3 demonstrate marked reductions in overall levels of select HPHCs and target constituents relative to CC smoke for non-intense and intense puffing regimes, respectively. Excluding primary components that form the base formulation (propylene glycol, glycerol, water, menthol (in mentholated formulations), and nicotine), all compared constituents were present at lower levels relative to the yields of CCs, and the total average HPHC and chemical reductions were 96% or greater across both non-intense and intense puff regimes (Table in Section S1.13; File S5). For a demonstration that these reductions are significant, see Supplementary Materials, Tables in Sections S1.14 and S1.15 (File S5) for a statistical analysis of JUUL2 VT compared to the certified reference values for the 1R6F cigarette.

Categorically, across non-intense and intense puff regimes, similar percent reductions in mean aerosol constituent levels compared to CCs were exhibited with the exception of glycidol. Increased levels of glycidol under intense puffing are likely attributed to the longer puff duration. Of the quantifiable analytes, only ethyl acetate and benzoic acid could not be compared due to a lack of CC source values. Mean levels of chromium were found to be quantifiable only under intense puffing, at one timepoint, in one sample (T0; VT). Given the lack of consistent quantifiable measurements across replicates, regimes, and timepoints, the chromium results were deemed equivocal and removed from comparison; see the Supplementary Materials (File S1) for additional details.

#### 4.1.3. Qualitative Risk Assessment

A qualitative risk assessment using the results from the targeted analytical chemistry stability studies conducted for each product was performed. Targeted constituents were grouped into either carcinogens or other toxicants that included respiratory toxicants, cardiovascular toxicants, and reproductive or developmental toxicants (Table 1) [4,47,48]. For the targeted analytes that were detected above the level of quantification, the highest value measured in the targeted analytical chemistry stability studies was used for comparison as a worst-case comparison with values from mainstream reference cigarette smoke. For carcinogens, there were significant reductions in levels measured in each JUUL2 product regardless of puffing regimen. For respiratory, cardiovascular, and reproductive and developmental toxicants, except for the main ENDS ingredients (nicotine, propylene glycol, and glycerin), there were also significant reductions that ranged from ≥40.5% to ≥99% in each JUUL2 product regardless of puffing regimen compared to levels in mainstream reference cigarette smoke.

Eight (8) of thirteen (13) carcinogenic HPHCs (established or proposed) evaluated were not detected above the limit of quantification or detection in any JUUL2 product. Four of the five carcinogens detected above the limit of quantification (acetaldehyde, formaldehyde, glycidol, and NNN) ranged from 66.7% to ≥99% lower in JUUL2 System aerosols compared to mainstream reference cigarette smoke. We believe the significant decreases in the levels of these carcinogens in JUUL2 System aerosols compared to reference mainstream cigarette smoke support substantial reductions in subsequent exposures and associated cancer risks from the use of the JUUL2 System relative to cigarette smoking.

### 4.2. Toxicological Evaluation

The complete in vitro toxicological results of VT, CM, PM, AT, RM, and SM JUUL2 pod aerosol condensate testing and 3R4F reference cigarette smoke condensate testing can be found in the Supplementary Materials, File S6.

#### 4.2.1. NRU Cytotoxicity Assay

**Figure 4:**
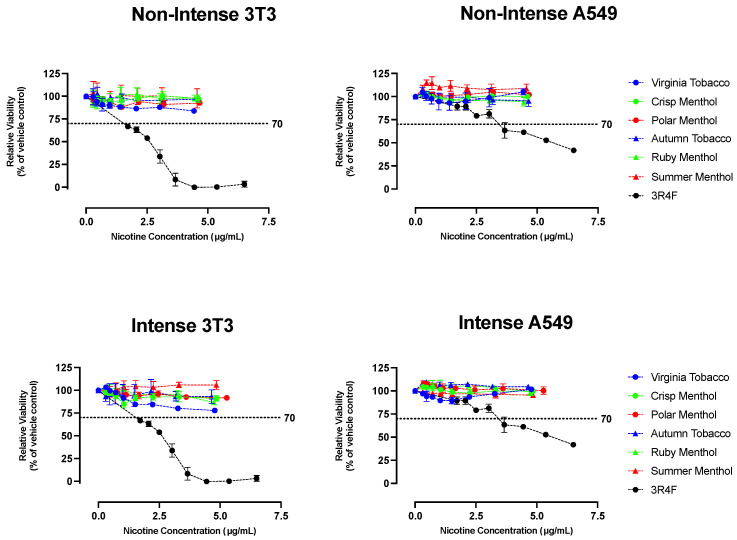
BALB/c 3T3 and A549 cytotoxicity of intense and non-intense ENDS aerosol and 3R4F smoke condensates, measured in the NRU assay. The dotted line represents the 70% relative viability cut-off defining cytotoxicity [98].

Results obtained with the vehicle and positive controls demonstrated the validity and sensitivity of the test system in each assay. None of the VT, CM, PM, AT, RM, or SM JUUL2 System condensates were found to be cytotoxic in the NRU assay using either BALB/c 3T3 and A549 cells regardless of puffing regimen (Figure 4) under the assay conditions. In contrast, the 3R4F condensate was found to be cytotoxic in both cell lines under the assay conditions, which is consistent with previous work [102,106]. See the Supplementary Materials (File S6) for complete NRU results on a mg ACM basis for ENDSs and mg total particulate mass (TPM) basis for 3R4F cigarettes.

#### 4.2.2. Ames Mutagenicity Assay

Results obtained with the vehicle and strain-specific positive controls demonstrated the validity and sensitivity of these test systems for detecting chemical-induced mutagenicity in each assay. None of the VT, CM, PM, AT, RM, or SM JUUL2 System condensates were found to be mutagenic in the Ames assay in any S. typhimurium strain regardless of puffing regimen or metabolic activation under the assay conditions (Table 2). In contrast, the S9-treated 3R4F condensate was found to be mutagenic in three S. typhimurium strains (TA98, TA100, and TA1537 all with metabolic activation) under the conditions of the assay (Table 2). Complete results for all Ames mutagenicity assays are in the Supplementary Materials (File S6).

#### 4.2.3. Micronucleus Genotoxicity Assay

The results obtained with the vehicle and positive controls demonstrated the suitability of each assay. For the JUUL2 System condensates, neither precipitation nor cytotoxicity were observed in any treatment condition, and therefore, MN frequencies were evaluated up to the solvent concentration limit (1% *v/v*). None of the JUUL2 VT, CM, PM, AT, RM, or SM condensates were found to be genotoxic in any of the test conditions of the MN assay regardless of the puffing regimen (Table S2; Supplementary Materials, File S6). In contrast, the 3R4F non-intense and intense condensates were respectively found to be equivocal and positive for genotoxicity (Table S2; Supplementary Materials, File S6).

## 5. Discussion

Following the principles of the “Tobacco Harm Reduction” strategy, a practical framework to evaluate novel ENDS products was presented. The application of the proposed framework on the novel JUUL2 product demonstrates that, as an alternative to combustible cigarettes, the JUUL2 device paired with JUUL2 formulations (VT, CM, PM, AT, RM, and SM) generates a significantly reduced number of HPHCs and chemicals quantified and does not exhibit cytotoxicity, mutagenicity, or genotoxicity under both non-intense and intense puffing conditions. The qualitative risk assessment confirmed that there were substantial reductions in the level of carcinogens in every JUUL2 product compared to CCs. Additionally, the qualitative risk assessment confirmed that except for the main ENDS ingredients, there was also a substantial reduction in levels of respiratory toxicants, cardiovascular toxicants, and reproductive or developmental toxicants in each JUUL2 product compared to CCs.

The proposed evaluation framework can be adapted for continual assessment throughout the product development lifecycle and, where applicable, be utilized to generate data in support of regulatory filings. As novel ENDS product development continues at a rapid pace with advancements in the device (cigalike, disposable, open tank, rechargeable, etc.), container system (pod-based, cartridge-based, non-refillable, refillable, etc.), aerosolization mechanism (cotton wick, silica wick, ceramic coil, etc.), formulation (free base nicotine, nicotine salt, natural vs. synthetic ingredients, etc.), and associated technology (Bluetooth, access restrictions, counterfeit compatibility, etc.), evaluation remains a critical step for manufacturers to have a role in understanding or reducing potential health risks from ENDSs. Despite progress, much work remains in terms of having a clear and complete list of recommended methods, guidance documents, and approaches that satisfy the multitude of regulatory requirements or total weight of evidence substantiation [107]. Offered within the JUUL2 case study are insights on study design and conduct, data analysis and interpretation, and qualitative risk assessment for both a chemical and in vitro toxicological characterization.

### 5.1. Study Design and Conduct Considerations

Various regulatory requirements and guidance documents helped define a list of assays (neutral red uptake cytotoxicity assay, mutagenicity with the Ames assay, and mammalian genotoxicity with the micronucleus assay) and select constituents for identification within the emissions but do not specifically address the manner in which studies are conducted. Associations such as CORESTA have formed subgroups (E-Vapour (EVAP) and In Vitro Toxicity Testing) that seek to align and standardize testing approaches primarily by adapting established CC methods for use with ENDSs [96]. Focus on this area of scientific research and study conduct further assists in generating robust methodologies that characterize e-liquids, aerosol emissions, and device properties potentially leading to the optimization of system characteristics that can minimize exposure.

Outlined within the JUUL2 case study are some examples of study conduct advancements that support analyses of the complete product, such as the use of “EOL” for reporting on a “whole-pod” basis. These advancements can become important when evaluating cross-product comparisons (e.g., per-cig yield vs. per-pod yield, 1 mL pod yield vs. 2 mL pod yield). Furthermore, the “EOL” puff collections also satisfy a more robust approach to aerosol condensate production for toxicological assay dosing. The chemical composition and stability of aerosol condensates utilized in toxicological evaluations are often minimally characterized, and there is a lack of a standardized condensate production approach [108]. This has led to oversight relative to the impact of factors such as the number of puffs per pod taken to achieve the target concentration and the nature of the condensate solvent. Additional collection methodology improvements such as the use of electrostatic precipitation within chemical metal analysis were incorporated to reduce potential contamination from the collection apparatus. Lowering the level of background trace metals also supports a lower effective quantification limit; however, it does not eliminate the possibility of random, sporadic quantifiable replicates (Section 4.1.2).

Undoubtedly, chemical and toxicological methods, techniques, and modifications will continue to develop alongside the advancement of product technology; therefore, critical review of the study conduct remains essential prior to data analysis and interpretation.

### 5.2. Data Analysis and Interpretation Considerations

For the aerosol chemical constituent comparison of ENDSs to CCs, several methods have been used to analyze and interpret results (e.g., per puff, normalized to per mg nicotine, per estimated daily exposure) [13,18,19,109]. One study has discussed the calculational impacts such as comparisons made on a per-nicotine basis versus per-puff and the impact of potential errors brought about by using imputed values and concluded that comparing results on a per-nicotine or per-puff basis exhibited minimal differences between the two approaches largely due to the significant amount of non-quantifiable analytes in the ENDS product [19]. However, when comparing only the quantifiable analytes on a per-nicotine basis, products with lower nicotine concentrations provided greater estimated toxicant exposures despite the greater mass of aerosol generated by the comparator product(s). Furthermore, the handling of non-quantifiable value imputation (midpoint of LOD/LOQ approach, LOD/√2, predicted values from models, and use of sub-detection limit values presented by the analytical method) was deemed inconsequential based on the substantial differences between CC smoke and ENDS aerosol [19].

Within the JUUL2 product case study, stability trends were assessed (Supplementary Materials, File S1) and aerosol chemical constituent comparisons were nicotine-normalized (mg/mg) on a “whole-pod” basis, negating the effect of any variable constituent generation during pod life [92,110]. Given the nicotine concentrations and deliveries were consistent across the JUUL2 product formulations and similar substantial decreases in ENDS aerosol constituent concentrations relative to CCs were observed, comparison on a per-nicotine basis was deemed appropriate. Where aerosol constituent levels below the LOD and LOQ were observed, values were imputed as LOD/2 and (LOQ + LOD/2), respectively. Across stability study timepoints, the highest value from any timepoint was used when comparing the JUUL2 System to CCs. The percent difference was not comparable (NC) if both the JUUL2 System and CCs were BLOQ or BLOD, if the JUUL2 System aerosol yield was quantifiable and the CC LOQ was greater than the JUUL2 System quantifiable yield, or if the CC yield was quantifiable and the JUUL2 LOQ was greater than the CC quantifiable yield.

Similar to chemistry, several methodologies have been utilized for a toxicological cross-product comparison [111,112,113,114,115]. Forest et al. (2022) discussed the different metrics and dose ranges used in studies for toxicological assessment of ENDSs, which included number of puffs, puffs/mL, puffs/m^2^, concentration of a compound (i.e., flavor), and ratio % (corresponding to the dilution of e-liquid, aerosol, or smoke extracts) [116]. While there are many ways to express doses and different dose ranges may be utilized, the question to balance is understanding the mechanistic aspect of the toxicological process or response (where unrealistically high doses can be used without inherent objection) versus exposure doses that mimic the real-life conditions of e-cigarette use [116]. Within the JUUL2 study, nicotine was chosen to characterize dosimetry across in vitro assays. The utilization of nicotine as a marker facilitates cross-product comparison and extrapolation of preclinical data and consumer use studies to help further explore the reduced risk potential of ENDS products [116].

### 5.3. Qualitative Risk Assessment Considerations

For each carcinogen measured above the LOD and LOQ, there was a 66.7% to ≥99% reduction across all JUUL2 System aerosols compared to mainstream reference cigarette smoke. The other carcinogens were not found above the LOQ or LOD and therefore were more than 99% reduced in all JUUL2 System aerosols compared to mainstream reference cigarette smoke. The World Health Organization (WHO) Study Group on Tobacco Product Regulation [117] identified nine toxicants as “most hazardous” in cigarette smoke, including five of the carcinogens reduced in all JUUL2 products’ aerosol relative to cigarette smoke:

- NNK and NNN (TSNAs);
- Benzo[a]pyrene (representative PAHs);
- VOCs, 1,3-butadiene, acrolein, acetaldehyde, formaldehyde, and benzene;
- Carbon monoxide (CO).

Fowles and Dybing (2003) estimated that combined, aldehydes and other small organic byproducts of combustion can account for approximately 44.3% (using EPA cancer risk values) to 62.4% (using Cal/EPA cancer risk values) of the cancer risk from mainstream cigarette smoke [118]. Acetaldehyde, formaldehyde, benzene, NNK, and NNN were measured in the targeted analytical chemistry stability studies and were found to be lower by 96% to ≥99% in all JUUL2 System aerosols compared to levels in mainstream reference cigarette smoke.

Carcinogens are generally assumed to have a linear dose–response relationship, which implies that there is an excess cancer risk associated with exposure to the constituent at any level, and the cancer risk for most carcinogens, including carcinogenic HPHCs, increases proportionally with increased exposure [119,120]. Taken together, of the 12 carcinogenic HPHCs evaluated in JUUL2 System aerosols, levels were not detected or were found at significantly lower levels compared to mainstream reference cigarette smoke, indicating a likely substantial reduction in user exposures and associated cancer risk.

With the exception of the primary e-liquid ingredients (nicotine, propylene glycol, and glycerin), the levels of established and proposed HPHCs with known respiratory, cardiovascular, or reproductive or developmental toxicities were also reduced in aerosol, regardless of puffing regimen, in all JUUL2 products compared to reference mainstream cigarette smoke. Substantially lower levels of these HPHCs support significant reductions in overall toxicant exposures and associated noncancer hazard from the use of the JUUL2 products relative to cigarette smoking. These conclusions based upon a comparison of aerosol constituent levels from targeted analytical chemistry stability studies between JUUL2 products and mainstream cigarette smoke are consistent with the in vitro assay results. The in vitro assay results showed that all JUUL2 products were not cytotoxic, not mutagenic, and not genotoxic within the assay conditions, while mainstream cigarette smoke was cytotoxic, mutagenic, and genotoxic within the assay conditions.

### 5.4. Regulatory Compliance

Dependent on the intended marketplace, country/region-specific regulatory standards or guidance documents should be followed. For example, in the US, modifications to the product characterization may include additional factors such as the requirement to analyze three (3) separate manufactured batches (seven replicates per constituent, per batch), the inclusion of e-liquid analyses, the inclusion of non-targeted analyses (on e-liquid and aerosol), or a battery of complementary clinical, behavioral, and populational studies. Contrasting the extensive characterization requirements in the U.S., Europe and European Member State guidance documents tend to emphasize the evaluation of ingredient sourcing and toxicological assessments thereof with additional considerations on banned ingredients, emission limits for specific analytes, and nicotine dose uniformity characterizations [9,62]. Within Asia, compliance attributes trend similar to the European entities, with a focus on factors such as permissions of e-liquid additives or “pollution” limits on specific analytes [60].

### 5.5. Limitations

The primary intent of this proposed framework was geared towards internal evaluation during the product development lifecycle. As such, this proposed framework is more limited than some other proposed product evaluation frameworks (Bergman et al., 2015; Murphy et al., 2017; Camacho et al., 2021), but also more extensive than others (Costigan and Meredith, 2015; Iskandar et al., 2016) [63,64,65,67,121]. This proposed framework is also consistent with the recent framework (Dempsey et al., 2023) proposed for heat-not-burn tobacco products [87]. While this practical framework and case study example focus on a reduction in toxicity relative to cigarettes, and could be performed relative to heat-not-burn tobacco products or other ENDS products, this framework does not cover aspects such as populational effects, which additional clinical, behavioral, or modeling studies work to address [64,65,121].

Discussed but not wholly presented within the JUUL2 case study is the critical aspect that all materials, components, or ingredients utilized within the novel ENDS product passed the preliminary toxicological assessment. Complete aerosol chemical analysis (e.g., non-targeted chemical analysis) was not included in the proposed framework because it is not universally required by regulators, although results for several U.S. products have been published [20,21]. For the intense puffing regimen chosen, no standardized regimen has been published to date; therefore, a maximum device cut-off (6 s) was selected; however, other puffing conditions have been deemed suitable for other products (including alternative non-intense puffing regimens).

Regarding the specific in vitro product testing within the proposed framework, although all in vitro product testing programs have limitations, the use of standardized OECD assays [97,100,101], ISO 17025 accredited laboratories [91], and similar aerosol collection techniques for all tested products minimizes these limitations to provide an adequate and scientifically valid in vitro toxicological assessment of generated aerosols. Our proposed framework does not incorporate the systems toxicology recommendations for in vitro assays by Iskandar et al. (2016) due to the limited availability of these methods at contract research organizations and, to date, their limited demonstrated regulatory value (IQOS TPL, 29 April 2019; page 39) [63,122].

## 6. Conclusions

In this study, a proposed framework for chemical and toxicological characterization was applied to the novel ENDS product JUUL2. Respective JUUL2 formulation, Virginia Tobacco, Crisp Menthol, Polar Menthol, Autumn Tobacco, Ruby Menthol, and Summer Menthol 18 mg/mL, aerosols were evaluated for relevant HPHCs and chemicals over a 12-month stability study, and cytotoxic, mutagenic, and genotoxic endpoints were assessed. Following the framework, the qualitative comparison to CCs demonstrates the products’ harm reduction potential through the significant reductions (>96% on average) in toxicant emissions coupled with the lack of toxicologically relevant responses under both non-intense and intense regimes.

## Figures and Tables

**Table 1:** Measured aerosol constituents and pH.

Primary Constituents	Nicotine (RDT, AD)	Propylene Glycol	Water	Glycerol
	Menthol	Diethylene Glycol (PC)	Ethylene Glycol (RT, RDT)	
Carbonyls	Acetaldehyde (CA, RT, AD)	Acrolein (RT, CT)	Diacetyl (RT)	Formaldehyde (CA, RT)
	Acetyl Propionyl (RT)	Butyraldehyde (RT)	Crotonaldehyde (CA)	Furfural (RT)
Glycidol	Glycidol (CA)			
Nicotine Degradants	β-Nicotyrine	Cotinine	Myosmine	Nicotine N Oxide
	Nornicotine (AD)	Anabasine (AD)	Anatabine	
TSNAs	NNN (CA)	NNK (CA)		
Metals	Arsenic (CA, CT, RDT)	Cobalt (CA, CT)	Lead (CA, CT, RDT)	Tin
	Beryllium (CA)	Copper	Nickel (CA, RT)	Selenium (RT)
	Chromium (CA, RT, RDT)	Iron	Silver	Gold
	Zinc	Cadmium (CA, RT, RDT)		
Organic Acids	Benzoic Acid	Propionic Acid (RT)		
VOCs	1,3-Butadiene (CA, RT, RDT)	Acrylonitrile (CA, RT)	Propylene Oxide (CA, RT)	Isoprene (CA)
	Toluene (RT, RDT)	Benzene (CA, CT, RDT)		
Esters and Alcohols	1-Butanol	Benzyl Acetate (RT)	Ethyl Acetate (RT)	Ethyl Acetoacetate (RT)
	Isoamyl Acetate (RT)	Isobutyl Acetate (RT)	Methyl Acetate (RT)	
pH	pH			

CA = carcinogen, RT = respiratory toxicant, CT = cardiovascular toxicant, RDT = reproductive or developmental toxicant, PC = poisonous chemical, AD = addictive.

**Table 2:** Mutagenicity (Ames) and genotoxicity (MN) results of intense and non-intense ENDS aerosol and 3R4F smoke condensates.

Formulation	Ames Assay		MN Assay	
	Non-Intense	Intense	Non-Intense	Intense
	Condensate	Condensate	Condensate	Condensate
Autumn Tobacco	Negative	Negative	Negative	Negative
Virginia Tobacco	Negative	Negative	Negative	Negative
Crisp Menthol	Negative	Negative	Negative	Negative
Polar Menthol	Negative	Negative	Negative	Negative
Ruby Menthol	Negative	Negative	Negative	Negative
Summer Menthol	Negative	Negative	Negative	Negative
3R4F	Positive	Positive	Equivocal	Positive

## Data Availability

The data presented in this study are available within the Supplementary Materials.

## Supplementary Materials

The following supporting information can be downloaded at https://www.mdpi.com/article/10.3390/toxics12010041/s1, File S1: Analysis of Chemical Constituents and Method Details; File S2: Per Collection Limit of Detection and Limit of Quantification Limits; File S3: Per Collection T0-T12 Analytical Stability Results; File S4: Nicotine Normalized T0-T12 Analytical Stability Results & Comparator Values; File S5: Percent Difference to Combustible Cigarettes; File S6: Analysis of Toxicity Studies and Method Details.
