# Supplementary material for: A Practical Framework for Novel Electronic Nicotine Delivery System Evaluation: Chemical and Toxicological Characterization of JUUL2 Aerosol and Comparison with Reference Cigarettes
Source: Toxics. 2024 Jan 4;12(1):41. doi: 10.3390/toxics12010041 (PMC10820849; doi:10.3390/toxics12010041)
Supplement: Supplementary file 1 [file toxics-12-00041-s001.zip › Supplementary_Material_File S4_Nicotine Normalized T0-T12 Analytical Stability Resul.pdf]

# *Supplementary Materials File S4*

## 1 Non-Intense and Intense Nicotine Normalized Analytical Stability Results

### 1.1 Virginia Tobacco 18 mg/mL Non-Intense Nicotine Normalized Analytical Stability Results

| Parameter/ Constituent | Units | T0 | T3 | T6 | T9 | T12 | Max |
|---|---|---|---|---|---|---|---|
| **# of puffs** | NA | 150 | 134 | 141 | 132 | 114 | 150 |
| **pH** | NA | 6.01 | 6.19 | 6.14 | 5.90 | 5.93 | 6.19 |
| **Primary Constituents** | | | | | | | |
| **Nicotine** | mg/mg nicotine | 1.00E+00 | 1.00E+00 | 1.00E+00 | 1.00E+00 | 1.00E+00 | 1.00E+00 |
| **Glycerol** | mg/mg nicotine | 3.82E+01 | 4.19E+01 | 4.03E+01 | 4.21E+01 | 4.35E+01 | 4.35E+01 |
| **Propylene Glycol** | mg/mg nicotine | 2.39E+01 | 2.50E+01 | 2.45E+01 | 2.44E+01 | 2.48E+01 | 2.50E+01 |
| **Water** | mg/mg nicotine | 4.52E+00 | 5.63E+00 | 5.68E+00 | 4.67E+00 | 5.21E+00 | 5.68E+00 |
| **Menthol** | mg/mg nicotine | ~ 4.30E-03 | ~ 5.26E-02 | ~ 4.57E-03 | ~ 4.29E-03 | ~ 5.69E-02 | ~ 5.69E-02 |
| **Diethylene Glycol** | mg/mg nicotine | ~ 4.57E-03 | ~ 4.90E-03 | ~ 4.69E-03 | ~ 4.40E-03 | ~ 5.33E-03 | ~ 5.33E-03 |
| **Ethylene Glycol** | mg/mg nicotine | ~ 4.86E-03 | ~ 5.24E-03 | ~ 5.01E-03 | ~ 4.70E-03 | ~ 5.71E-03 | ~ 5.71E-03 |
| **Carbonyls & Glycidol** | | | | | | | |
| **Acetaldehyde** | mg/mg nicotine | 1.93E-04 | 1.68E-04 | 1.82E-04 | 2.16E-04 | 2.74E-04 | 2.74E-04 |
| **Acrolein** | mg/mg nicotine | 8.82E-05 | 7.22E-05 | ~ 3.64E-05 | ~ 3.42E-05 | 1.78E-04 | 1.78E-04 |
| **Diacetyl** | mg/mg nicotine | ~ 3.45E-05 | ~ 3.78E-05 | ~ 3.26E-06 | ~ 3.06E-06 | ~ 3.90E-05 | ~ 3.90E-05 |
| **Formaldehyde** | mg/mg nicotine | 8.42E-04 | 9.52E-04 | 8.69E-04 | 1.04E-03 | 1.13E-03 | 1.13E-03 |
| **Glycidol** | mg/mg nicotine | 2.24E-04 | 1.96E-04 | 2.39E-04 | 2.35E-04 | 1.96E-04 | 2.39E-04 |
| **Acetyl Propionyl** | mg/mg nicotine | ~ 3.01E-06 | ~ 3.29E-06 | ~ 3.21E-06 | ~ 3.02E-06 | ~ 3.58E-06 | ~ 3.58E-06 |
| **Butyraldehyde** | mg/mg nicotine | ~ 3.33E-06 | ~ 3.64E-06 | ~ 3.21E-06 | ~ 3.01E-06 | ~ 3.58E-06 | ~ 3.64E-06 |
| **Crotonaldehyde** | mg/mg nicotine | ~ 3.05E-06 | ~ 3.33E-06 | ~ 5.15E-06 | ~ 4.84E-06 | ~ 5.74E-06 | ~ 5.74E-06 |
| **Furfural** | mg/mg nicotine | ~ 4.22E-06 | ~ 4.61E-06 | ~ 5.27E-06 | ~ 4.95E-06 | ~ 5.88E-06 | ~ 5.88E-06 |
| **Nicotine Degradants & TSNAs** | | | | | | | |

| | | | | | | | |
|---|---|---|---|---|---|---|---|
| **β-Nicotyrine** | mg/mg nicotine | 8.44E-05 | 6.92E-05 | 7.59E-05 | 1.35E-04 | 1.11E-04 | 1.35E-04 |
| **Cotinine** | mg/mg nicotine | 1.87E-03 | 2.41E-03 | 1.80E-03 | 2.09E-03 | 2.25E-03 | 2.41E-03 |
| **Myosmine** | mg/mg nicotine | 1.72E-03 | 1.99E-03 | 1.59E-03 | 1.61E-03 | 1.65E-03 | 1.99E-03 |
| **Nicotine N Oxide** | mg/mg nicotine | ~ 3.32E-05 | ~ 3.62E-05 | 7.73E-05 | ~ 2.65E-05 | ~ 3.14E-05 | 7.73E-05 |
| **Nornicotine** | mg/mg nicotine | 3.32E-04 | 5.48E-04 | 5.60E-04 | 6.62E-04 | 7.91E-04 | 7.91E-04 |
| **Anabasine** | mg/mg nicotine | ~ 2.50E-06 | ~ 2.73E-06 | ~ 2.58E-06 | ~ 2.43E-06 | ~ 2.88E-06 | ~ 2.88E-06 |
| **Anatabine** | mg/mg nicotine | ~ 2.49E-06 | ~ 2.72E-06 | ~ 2.57E-06 | ~ 2.66E-05 | ~ 2.87E-06 | ~ 2.66E-05 |
| **NNK** | mg/mg nicotine | ~ 7.49E-07 | NT | NT | NT | NT | ~ 7.49E-07 |
| **NNN** | mg/mg nicotine | ~ 8.99E-08 | NT | NT | NT | NT | ~ 8.99E-08 |
| **Metals** | | | | | | | |
| **Arsenic** | mg/mg nicotine | ~ 2.72E-07 | ~ 1.78E-06 | ~ 2.84E-07 | ~ 1.60E-06 | ~ 3.17E-07 | ~ 1.78E-06 |
| **Beryllium** | mg/mg nicotine | ~ 5.99E-08 | ~ 6.54E-08 | ~ 6.25E-08 | ~ 5.87E-08 | ~ 6.97E-08 | ~ 6.97E-08 |
| **Cadmium** | mg/mg nicotine | ~ 2.72E-08 | ~ 2.97E-08 | ~ 2.84E-08 | ~ 2.67E-08 | ~ 3.17E-08 | ~ 3.17E-08 |
| **Chromium** | mg/mg nicotine | ~ 1.63E-06 | ~ 2.97E-07 | ~ 2.84E-07 | ~ 2.67E-07 | ~ 1.90E-06 | ~ 1.90E-06 |
| **Cobalt** | mg/mg nicotine | ~ 1.36E-08 | ~ 1.49E-08 | ~ 1.42E-08 | ~ 1.33E-08 | ~ 1.58E-08 | ~ 1.58E-08 |
| **Copper** | mg/mg nicotine | ~ 2.72E-06 | ~ 7.43E-07 | ~ 7.11E-07 | ~ 6.67E-07 | ~ 7.92E-07 | ~ 2.72E-06 |
| **Gold** | mg/mg nicotine | ~ 4.63E-06 | NT | NT | NT | NT | ~ 4.63E-06 |
| **Iron** | mg/mg nicotine | ~ 8.17E-06 | ~ 2.97E-06 | ~ 2.84E-06 | ~ 2.67E-06 | ~ 3.17E-06 | ~ 8.17E-06 |
| **Lead** | mg/mg nicotine | ~ 6.81E-08 | ~ 7.43E-08 | ~ 7.11E-08 | ~ 6.67E-08 | ~ 7.92E-08 | ~ 7.92E-08 |
| **Nickel** | mg/mg nicotine | ~ 1.36E-07 | ~ 1.49E-07 | ~ 1.42E-07 | ~ 1.33E-07 | ~ 1.58E-07 | ~ 1.58E-07 |
| **Selenium** | mg/mg nicotine | ~ 6.51E-07 | 1.40E-06 | ~ 6.80E-07 | ~ 6.38E-07 | ~ 7.57E-07 | 1.40E-06 |
| **Silver** | mg/mg nicotine | ~ 2.72E-08 | ~ 2.97E-08 | ~ 2.84E-08 | ~ 2.67E-08 | ~ 3.17E-08 | ~ 3.17E-08 |
| **Tin** | mg/mg nicotine | ~ 1.63E-06 | ~ 2.97E-07 | ~ 2.84E-07 | ~ 1.60E-06 | ~ 3.17E-07 | ~ 1.63E-06 |
| **Zinc** | mg/mg nicotine | ~ 1.36E-05 | ~ 4.46E-06 | ~ 4.26E-06 | ~ 4.00E-06 | ~ 4.75E-06 | ~ 1.36E-05 |
| **Organic Acids** | | | | | | | |
| **Benzoic Acid** | mg/mg nicotine | 5.37E-01 | 4.90E-01 | 5.67E-01 | 4.96E-01 | 5.26E-01 | 5.67E-01 |
| **Propionic Acid** | mg/mg nicotine | ~ 9.60E-05 | ~ 1.05E-04 | ~ 1.76E-03 | ~ 1.04E-03 | ~ 1.12E-04 | ~ 1.76E-03 |
| **VOCs** | | | | | | | |

| | | | | | | | |
|---|---|---|---|---|---|---|---|
| **1,3-Butadiene** | mg/mg nicotine | ~ 1.49E-06 | NT | NT | NT | NT | ~ 1.49E-06 |
| **Acrylonitrile** | mg/mg nicotine | ~ 9.79E-07 | NT | NT | NT | NT | ~ 9.79E-07 |
| **Benzene** | mg/mg nicotine | ~ 1.71E-06 | NT | NT | NT | NT | ~ 1.71E-06 |
| **Isoprene** | mg/mg nicotine | ~ 2.30E-06 | NT | NT | NT | NT | ~ 2.30E-06 |
| **Propylene Oxide** | mg/mg nicotine | ~ 3.96E-06 | NT | NT | NT | NT | ~ 3.96E-06 |
| **Toluene** | mg/mg nicotine | ~ 1.21E-05 | NT | NT | NT | NT | ~ 1.21E-05 |
| **Esters & Alcohols** | | | | | | | |
| **1-Butanol** | mg/mg nicotine | ~ 4.41E-05 | NT | NT | NT | NT | ~ 4.41E-05 |
| **Benzyl Acetate** | mg/mg nicotine | ~ 6.84E-05 | NT | NT | NT | NT | ~ 6.84E-05 |
| **Ethyl Acetate** | mg/mg nicotine | ~ 7.83E-05 | NT | NT | NT | NT | ~ 7.83E-05 |
| **Ethyl Acetoacetate** | mg/mg nicotine | ~ 5.59E-05 | NT | NT | NT | NT | ~ 5.59E-05 |
| **Isoamyl Acetate** | mg/mg nicotine | ~ 4.77E-05 | NT | NT | NT | NT | ~ 4.77E-05 |
| **Isobutyl Acetate** | mg/mg nicotine | ~ 4.73E-05 | NT | NT | NT | NT | ~ 4.73E-05 |
| **Methyl Acetate** | mg/mg nicotine | ~ 5.08E-05 | NT | NT | NT | NT | ~ 5.08E-05 |

~ = approximated numeric value (see Supplemental Materials Appendix 1A) generated from the corresponding LOD and LOQ (See Supplemental Materials Appendix 1B)

## 1.2 Virginia Tobacco 18 mg/mL Intense Nicotine Normalized Analytical Stability Results

| Parameter/ Constituent | Units | T0 | T3 | T6 | T9 | T12 | Max |
|---|---|---|---|---|---|---|---|
| **# of puffs** | NA | 85 (*86) | 86 | 87 | 84 | 83 | 87 |
| **pH** | NA | 6.17 | 6.46 | 5.96 | 6.17 | 5.96 | 6.46 |
| ***Primary Constituents** | | | | | | | |
| **Nicotine** | mg/mg nicotine | 1.00E+00 | 1.00E+00 | 1.00E+00 | 1.00E+00 | 1.00E+00 | 1.00E+00 |
| **Glycerol** | mg/mg nicotine | 3.77E+01 | 4.12E+01 | 4.11E+01 | 4.06E+01 | 4.16E+01 | 4.16E+01 |
| **Propylene Glycol** | mg/mg nicotine | 2.40E+01 | 2.53E+01 | 2.46E+01 | 2.50E+01 | 2.47E+01 | 2.53E+01 |
| **Water** | mg/mg nicotine | 3.97E+00 | 4.61E+00 | 4.55E+00 | 5.00E+00 | 4.85E+00 | 5.00E+00 |
| **Menthol** | mg/mg nicotine | ~ 4.50E-03 | ~ 4.38E-03 | ~ 4.45E-03 | ~ 4.51E-03 | ~ 4.74E-03 | ~ 4.74E-03 |

| | | | | | | | |
|---|---|---|---|---|---|---|---|
| **Diethylene Glycol** | mg/mg nicotine | ~ 4.78E-03 | ~ 4.49E-03 | ~ 4.56E-03 | ~ 4.63E-03 | ~ 5.00E-03 | ~ 5.00E-03 |
| **Ethylene Glycol** | mg/mg nicotine | ~ 5.09E-03 | ~ 4.80E-03 | ~ 4.88E-03 | ~ 4.95E-03 | ~ 5.36E-03 | ~ 5.36E-03 |
| **Carbonyls & Glycidol** | | | | | | | |
| **Acetaldehyde** | mg/mg nicotine | 2.56E-04 | 2.10E-04 | 2.30E-04 | 2.49E-04 | 3.96E-04 | 3.96E-04 |
| **Acrolein** | mg/mg nicotine | 1.80E-04 | 1.26E-04 | 1.14E-04 | 1.23E-04 | 4.05E-04 | 4.05E-04 |
| **Diacetyl** | mg/mg nicotine | ~ 4.21E-05 | ~ 4.04E-05 | ~ 4.07E-05 | ~ 3.75E-06 | ~ 4.27E-05 | ~ 4.27E-05 |
| **Formaldehyde** | mg/mg nicotine | 1.30E-03 | 1.18E-03 | 1.27E-03 | 1.29E-03 | 1.48E-03 | 1.48E-03 |
| **Glycidol** | mg/mg nicotine | 3.37E-04 | 2.11E-04 | 2.95E-04 | 2.11E-04 | 2.65E-04 | 3.37E-04 |
| **Acetyl Propionyl** | mg/mg nicotine | ~ 3.68E-06 | ~ 3.52E-06 | ~ 3.65E-06 | ~ 3.70E-06 | ~ 3.92E-06 | ~ 3.92E-06 |
| **Butyraldehyde** | mg/mg nicotine | ~ 4.06E-06 | ~ 3.89E-06 | ~ 4.01E-05 | ~ 3.70E-06 | ~ 3.92E-06 | ~ 4.01E-05 |
| **Crotonaldehyde** | mg/mg nicotine | ~ 3.72E-06 | ~ 3.56E-06 | ~ 5.85E-06 | ~ 5.93E-06 | ~ 6.29E-06 | ~ 6.29E-06 |
| **Furfural** | mg/mg nicotine | ~ 5.15E-06 | ~ 4.92E-06 | ~ 5.99E-06 | ~ 4.32E-05 | ~ 6.43E-06 | ~ 4.32E-05 |
| **Nicotine Degradants & TSNAs** | | | | | | | |
| **β-Nicotyrine** | mg/mg nicotine | 1.37E-04 | 1.16E-04 | 1.59E-04 | 1.74E-04 | 1.42E-04 | 1.74E-04 |
| **Cotinine** | mg/mg nicotine | 2.02E-03 | 1.98E-03 | 2.29E-03 | 1.93E-03 | 2.54E-03 | 2.54E-03 |
| **Myosmine** | mg/mg nicotine | 1.88E-03 | 1.63E-03 | 1.98E-03 | 1.48E-03 | 1.92E-03 | 1.98E-03 |
| **Nicotine N Oxide** | mg/mg nicotine | ~ 4.05E-05 | ~ 3.87E-05 | 6.33E-05 | ~ 3.25E-05 | ~ 3.44E-05 | 6.33E-05 |
| **Nornicotine** | mg/mg nicotine | 3.75E-04 | 4.38E-04 | 6.63E-04 | 6.07E-04 | 8.71E-04 | 8.71E-04 |
| **Anabasine** | mg/mg nicotine | ~ 3.05E-06 | ~ 2.92E-06 | ~ 2.93E-06 | ~ 2.98E-06 | ~ 3.15E-06 | ~ 3.15E-06 |
| **Anatabine** | mg/mg nicotine | ~ 3.04E-06 | ~ 2.91E-06 | ~ 2.92E-06 | ~ 3.26E-05 | ~ 3.14E-06 | ~ 3.26E-05 |
| **NNK** | mg/mg nicotine | ~ 7.83E-07 | NT | NT | NT | NT | ~ 7.83E-07 |
| **NNN** | mg/mg nicotine | ~ 9.41E-08 | NT | NT | NT | NT | ~ 9.41E-08 |
| **Metals** | | | | | | | |
| **Arsenic** | mg/mg nicotine | ~ 2.85E-07 | ~ 2.73E-07 | ~ 2.77E-07 | ~ 2.81E-07 | ~ 2.97E-07 | ~ 2.97E-07 |
| **Beryllium** | mg/mg nicotine | ~ 6.27E-08 | ~ 6.00E-08 | ~ 6.09E-08 | ~ 6.17E-08 | ~ 6.54E-08 | ~ 6.54E-08 |
| **Cadmium** | mg/mg nicotine | ~ 2.85E-08 | ~ 2.73E-08 | ~ 2.77E-08 | ~ 2.81E-08 | ~ 2.97E-08 | ~ 2.97E-08 |
| **Chromium** | mg/mg nicotine | 4.11E-06 | ~ 2.73E-07 | ~ 2.77E-07 | ~ 2.81E-07 | ~ 2.97E-07 | 4.11E-06 |
| **Cobalt** | mg/mg nicotine | ~ 1.42E-08 | ~ 1.36E-08 | ~ 1.38E-08 | ~ 1.40E-08 | ~ 1.49E-08 | ~ 1.49E-08 |

| | | | | | | | |
|---|---|---|---|---|---|---|---|
| **Copper** | mg/mg nicotine | ~ 7.12E-07 | ~ 6.81E-07 | ~ 6.92E-07 | ~ 7.01E-07 | ~ 7.43E-07 | ~ 7.43E-07 |
| **Gold** | mg/mg nicotine | ~ 4.84E-06 | NT | NT | NT | NT | ~ 4.84E-06 |
| **Iron** | mg/mg nicotine | 3.40E-05 | ~ 2.73E-06 | ~ 2.77E-06 | ~ 2.81E-06 | ~ 2.97E-06 | 3.40E-05 |
| **Lead** | mg/mg nicotine | ~ 7.12E-08 | ~ 6.81E-08 | ~ 6.92E-08 | ~ 7.01E-08 | ~ 7.43E-08 | ~ 7.43E-08 |
| **Nickel** | mg/mg nicotine | ~ 4.27E-07 | ~ 1.36E-07 | ~ 1.38E-07 | ~ 1.40E-07 | ~ 1.49E-07 | ~ 4.27E-07 |
| **Selenium** | mg/mg nicotine | ~ 6.81E-07 | ~ 6.51E-07 | ~ 6.61E-07 | ~ 6.71E-07 | ~ 7.11E-07 | ~ 7.11E-07 |
| **Silver** | mg/mg nicotine | ~ 2.85E-08 | ~ 1.64E-07 | ~ 2.77E-08 | ~ 2.81E-08 | ~ 2.97E-08 | ~ 1.64E-07 |
| **Tin** | mg/mg nicotine | ~ 1.71E-06 | ~ 1.64E-06 | ~ 1.66E-06 | ~ 2.81E-07 | ~ 2.97E-07 | ~ 1.71E-06 |
| **Zinc** | mg/mg nicotine | ~ 4.27E-06 | ~ 4.09E-06 | ~ 4.15E-06 | ~ 4.21E-06 | ~ 4.46E-06 | ~ 4.46E-06 |
| **Organic Acids** | | | | | | | |
| **Benzoic Acid** | mg/mg nicotine | 5.30E-01 | 6.25E-01 | 7.84E-01 | 6.46E-01 | 5.86E-01 | 7.84E-01 |
| **Propionic Acid** | mg/mg nicotine | ~ 1.01E-04 | ~ 1.06E-03 | ~ 2.43E-03 | ~ 9.90E-05 | ~ 1.15E-03 | ~ 2.43E-03 |
| **VOCs** | | | | | | | |
| **1,3-Butadiene** | mg/mg nicotine | ~ 1.56E-06 | NT | NT | NT | NT | ~ 1.56E-06 |
| **Acrylonitrile** | mg/mg nicotine | ~ 1.02E-06 | NT | NT | NT | NT | ~ 1.02E-06 |
| **Benzene** | mg/mg nicotine | ~ 1.79E-06 | NT | NT | NT | NT | ~ 1.79E-06 |
| **Isoprene** | mg/mg nicotine | ~ 2.41E-06 | NT | NT | NT | NT | ~ 2.41E-06 |
| **Propylene Oxide** | mg/mg nicotine | ~ 4.15E-06 | NT | NT | NT | NT | ~ 4.15E-06 |
| **Toluene** | mg/mg nicotine | ~ 1.27E-05 | NT | NT | NT | NT | ~ 1.27E-05 |
| **Esters & Alcohols** | | | | | | | |
| **1-Butanol** | mg/mg nicotine | ~ 4.61E-05 | NT | NT | NT | NT | ~ 4.61E-05 |
| **Benzyl Acetate** | mg/mg nicotine | ~ 7.16E-05 | NT | NT | NT | NT | ~ 7.16E-05 |
| **Ethyl Acetate** | mg/mg nicotine | ~ 8.19E-05 | NT | NT | NT | NT | ~ 8.19E-05 |
| **Ethyl Acetoacetate** | mg/mg nicotine | ~ 5.85E-05 | NT | NT | NT | NT | ~ 5.85E-05 |
| **Isoamyl Acetate** | mg/mg nicotine | ~ 4.99E-05 | NT | NT | NT | NT | ~ 4.99E-05 |
| **Isobutyl Acetate** | mg/mg nicotine | ~ 4.94E-05 | NT | NT | NT | NT | ~ 4.94E-05 |
| **Methyl Acetate** | mg/mg nicotine | ~ 5.32E-05 | NT | NT | NT | NT | ~ 5.32E-05 |

~ = approximated numeric value (see Supplemental Materials Appendix 1A) generated from the corresponding LOD and LOQ (See Supplemental Materials Appendix 1B)

## 1.3 Crisp Menthol 18 mg/mL Non-Intense Nicotine Normalized Analytical Stability Results

| Parameter/ Constituent | Units | T0 | T3 | T6 | T9 | T12 | Max |
|---|---|---|---|---|---|---|---|
| **# of puffs** | NA | 125 | 112 | 121 | 120 | 115 | 125 |
| **pH** | NA | 6.26 | 6.36 | 6.33 | 6.30 | 6.11 | 6.36 |
| **Primary Constituents** | | | | | | | |
| **Nicotine** | mg/mg nicotine | 1.00E+00 | 1.00E+00 | 1.00E+00 | 1.00E+00 | 1.00E+00 | 1.00E+00 |
| **Glycerol** | mg/mg nicotine | 2.56E+01 | 2.69E+01 | 2.58E+01 | 2.64E+01 | 2.65E+01 | 2.69E+01 |
| **Propylene Glycol** | mg/mg nicotine | 3.48E+01 | 3.77E+01 | 3.80E+01 | 3.76E+01 | 3.67E+01 | 3.80E+01 |
| **Water** | mg/mg nicotine | 4.46E+00 | 3.79E+00 | 4.41E+00 | 4.07E+00 | 4.66E+00 | 4.66E+00 |
| **Menthol** | mg/mg nicotine | 7.17E-01 | 7.27E-01 | 7.17E-01 | 7.39E-01 | 7.03E-01 | 7.39E-01 |
| **Diethylene Glycol** | mg/mg nicotine | ~ 4.79E-03 | ~ 4.98E-03 | ~ 4.93E-03 | ~ 4.83E-03 | ~ 5.19E-03 | ~ 5.19E-03 |
| **Ethylene Glycol** | mg/mg nicotine | ~ 5.10E-03 | ~ 5.32E-03 | ~ 5.27E-03 | ~ 5.16E-03 | ~ 5.55E-03 | ~ 5.55E-03 |
| **Carbonyls & Glycidol** | | | | | | | |
| **Acetaldehyde** | mg/mg nicotine | 1.37E-04 | 1.60E-04 | 1.60E-04 | 1.46E-04 | 1.67E-04 | 1.67E-04 |
| **Acrolein** | mg/mg nicotine | ~ 3.55E-05 | ~ 3.76E-05 | ~ 3.83E-05 | ~ 3.76E-05 | 1.06E-04 | 1.06E-04 |
| **Diacetyl** | mg/mg nicotine | ~ 3.61E-05 | ~ 3.83E-05 | ~ 3.77E-05 | ~ 3.36E-06 | ~ 3.80E-05 | ~ 3.83E-05 |
| **Formaldehyde** | mg/mg nicotine | 5.11E-04 | 6.98E-04 | 6.18E-04 | 6.64E-04 | 7.17E-04 | 7.17E-04 |
| **Glycidol** | mg/mg nicotine | 1.66E-04 | 1.26E-04 | 1.39E-04 | 1.58E-04 | 1.65E-04 | 1.66E-04 |
| **Acetyl Propionyl** | mg/mg nicotine | ~ 3.16E-06 | ~ 3.34E-06 | ~ 3.38E-06 | ~ 3.31E-06 | ~ 3.49E-06 | ~ 3.49E-06 |
| **Butyraldehyde** | mg/mg nicotine | ~ 3.49E-06 | ~ 3.69E-06 | ~ 3.38E-06 | ~ 3.31E-06 | ~ 3.48E-06 | ~ 3.69E-06 |
| **Crotonaldehyde** | mg/mg nicotine | ~ 3.19E-06 | ~ 3.38E-06 | ~ 5.42E-06 | ~ 5.31E-06 | ~ 5.59E-06 | ~ 5.59E-06 |
| **Furfural** | mg/mg nicotine | ~ 4.42E-06 | ~ 4.67E-06 | ~ 5.55E-06 | ~ 5.43E-06 | ~ 5.72E-06 | ~ 5.72E-06 |
| **Nicotine Degradants & TSNAs** | | | | | | | |
| **β-Nicotyrine** | mg/mg nicotine | 1.07E-04 | 1.45E-04 | 1.69E-04 | 2.46E-04 | 2.25E-04 | 2.46E-04 |
| **Cotinine** | mg/mg nicotine | ~ 2.81E-05 | ~ 3.00E-05 | ~ 2.98E-05 | ~ 2.91E-05 | ~ 3.07E-05 | ~ 3.07E-05 |
| **Myosmine** | mg/mg nicotine | 8.75E-05 | 1.05E-04 | 1.32E-04 | 1.44E-04 | 1.66E-04 | 1.66E-04 |

| | | | | | | | |
|---|---|---|---|---|---|---|---|
| **Nicotine N Oxide** | mg/mg nicotine | ~ 3.48E-05 | 9.90E-05 | 8.09E-05 | 1.03E-04 | 6.04E-05 | 1.03E-04 |
| **Nornicotine** | mg/mg nicotine | 4.12E-04 | 5.39E-04 | 7.68E-04 | 7.26E-04 | 8.85E-04 | 8.85E-04 |
| **Anabasine** | mg/mg nicotine | ~ 2.62E-06 | ~ 2.77E-06 | ~ 2.72E-06 | ~ 2.66E-06 | ~ 2.80E-06 | ~ 2.80E-06 |
| **Anatabine** | mg/mg nicotine | ~ 2.61E-06 | ~ 2.76E-06 | ~ 2.71E-06 | ~ 2.65E-06 | ~ 2.79E-06 | ~ 2.79E-06 |
| **NNK** | mg/mg nicotine | ~ 7.85E-07 | NT | NT | NT | NT | ~ 7.85E-07 |
| **NNN** | mg/mg nicotine | ~ 9.42E-08 | NT | NT | NT | NT | ~ 9.42E-08 |
| **Metals** | | | | | | | |
| **Arsenic** | mg/mg nicotine | ~ 2.85E-07 | ~ 3.02E-07 | ~ 2.99E-07 | ~ 2.93E-07 | ~ 3.08E-07 | ~ 3.08E-07 |
| **Beryllium** | mg/mg nicotine | ~ 6.28E-08 | ~ 6.64E-08 | ~ 6.58E-08 | ~ 6.45E-08 | ~ 6.78E-08 | ~ 6.78E-08 |
| **Cadmium** | mg/mg nicotine | ~ 2.85E-08 | ~ 3.02E-08 | ~ 2.99E-08 | ~ 2.93E-08 | ~ 3.08E-08 | ~ 3.08E-08 |
| **Chromium** | mg/mg nicotine | ~ 1.71E-06 | ~ 3.02E-07 | ~ 2.99E-07 | ~ 2.93E-07 | ~ 3.08E-07 | ~ 1.71E-06 |
| **Cobalt** | mg/mg nicotine | ~ 1.43E-08 | ~ 1.51E-08 | ~ 1.50E-08 | ~ 1.46E-08 | ~ 1.54E-08 | ~ 1.54E-08 |
| **Copper** | mg/mg nicotine | ~ 2.85E-06 | ~ 7.55E-07 | ~ 7.48E-07 | ~ 7.32E-07 | ~ 7.71E-07 | ~ 2.85E-06 |
| **Gold** | mg/mg nicotine | ~ 4.85E-06 | NT | NT | NT | NT | ~ 4.85E-06 |
| **Iron** | mg/mg nicotine | 1.22E-05 | ~ 3.02E-06 | ~ 2.99E-06 | ~ 2.93E-06 | ~ 3.08E-06 | 1.22E-05 |
| **Lead** | mg/mg nicotine | ~ 7.13E-08 | ~ 7.55E-08 | ~ 7.48E-08 | ~ 7.32E-08 | ~ 7.71E-08 | ~ 7.71E-08 |
| **Nickel** | mg/mg nicotine | ~ 1.43E-07 | ~ 1.51E-07 | ~ 1.50E-07 | ~ 1.46E-07 | ~ 1.54E-07 | ~ 1.54E-07 |
| **Selenium** | mg/mg nicotine | ~ 6.82E-07 | ~ 7.21E-07 | ~ 7.15E-07 | ~ 7.00E-07 | ~ 7.37E-07 | ~ 7.37E-07 |
| **Silver** | mg/mg nicotine | ~ 2.85E-08 | ~ 3.02E-08 | ~ 2.99E-08 | ~ 2.93E-08 | ~ 3.08E-08 | ~ 3.08E-08 |
| **Tin** | mg/mg nicotine | ~ 1.71E-06 | ~ 1.81E-06 | ~ 2.99E-07 | ~ 2.93E-07 | ~ 3.08E-07 | ~ 1.81E-06 |
| **Zinc** | mg/mg nicotine | ~ 1.43E-05 | ~ 4.53E-06 | ~ 4.49E-06 | ~ 4.39E-06 | ~ 4.62E-06 | ~ 1.43E-05 |
| **Organic Acids** | | | | | | | |
| **Benzoic Acid** | mg/mg nicotine | 6.20E-01 | 5.16E-01 | 5.86E-01 | 5.39E-01 | 5.21E-01 | 6.20E-01 |
| **Propionic Acid** | mg/mg nicotine | ~ 1.77E-04 | ~ 1.87E-04 | ~ 1.05E-03 | ~ 1.14E-03 | ~ 1.20E-03 | ~ 1.20E-03 |
| **VOCs** | | | | | | | |
| **1,3-Butadiene** | mg/mg nicotine | ~ 1.57E-06 | NT | NT | NT | NT | ~ 1.57E-06 |
| **Acrylonitrile** | mg/mg nicotine | ~ 1.03E-06 | NT | NT | NT | NT | ~ 1.03E-06 |
| **Benzene** | mg/mg nicotine | ~ 1.80E-06 | NT | NT | NT | NT | ~ 1.80E-06 |
| **Isoprene** | mg/mg nicotine | ~ 2.41E-06 | NT | NT | NT | NT | ~ 2.41E-06 |

| | | | | | | | |
|---|---|---|---|---|---|---|---|
| **Propylene Oxide** | mg/mg nicotine | ~ 4.15E-06 | NT | NT | NT | NT | ~ 4.15E-06 |
| **Toluene** | mg/mg nicotine | ~ 2.64E-06 | NT | NT | NT | NT | ~ 2.64E-06 |
| **Esters & Alcohols** | | | | | | | |
| **1-Butanol** | mg/mg nicotine | ~ 4.62E-05 | NT | NT | NT | NT | ~ 4.62E-05 |
| **Benzyl Acetate** | mg/mg nicotine | ~ 7.17E-05 | NT | NT | NT | NT | ~ 7.17E-05 |
| **Ethyl Acetate** | mg/mg nicotine | 6.31E-03 | NT | NT | NT | NT | 6.31E-03 |
| **Ethyl Acetoacetate** | mg/mg nicotine | ~ 5.86E-05 | NT | NT | NT | NT | ~ 5.86E-05 |
| **Isoamyl Acetate** | mg/mg nicotine | ~ 5.00E-05 | NT | NT | NT | NT | ~ 5.00E-05 |
| **Isobutyl Acetate** | mg/mg nicotine | ~ 4.95E-05 | NT | NT | NT | NT | ~ 4.95E-05 |
| **Methyl Acetate** | mg/mg nicotine | ~ 5.33E-05 | NT | NT | NT | NT | ~ 5.33E-05 |

~ = approximated numeric value (see Supplemental Materials Appendix 1A) generated from the corresponding LOD and LOQ (See Supplemental Materials Appendix 1B)

## 1.4 Crisp Menthol 18 mg/mL Intense Nicotine Normalized Analytical Stability Results

| Parameter/ Constituent | Units | T0 | T3 | T6 | T9 | T12 | Max |
|---|---|---|---|---|---|---|---|
| **# of puffs** | NA | 81 | 88 | 82 | 87 | 81 | 88 |
| **pH** | NA | 6.24 | 6.53 | 6.27 | 6.20 | 6.17 | 6.53 |
| ***Primary Constituents** | | | | | | | |
| **Nicotine** | mg/mg nicotine | 1.00E+00 | 1.00E+00 | 1.00E+00 | 1.00E+00 | 1.00E+00 | 1.00E+00 |
| **Glycerol** | mg/mg nicotine | 2.52E+01 | 2.68E+01 | 2.60E+01 | 2.65E+01 | 2.67E+01 | 2.68E+01 |
| **Propylene Glycol** | mg/mg nicotine | 3.45E+01 | 3.76E+01 | 3.72E+01 | 3.75E+01 | 3.71E+01 | 3.76E+01 |
| **Water** | mg/mg nicotine | 3.83E+00 | 3.89E+00 | 4.28E+00 | 3.80E+00 | 4.29E+00 | 4.29E+00 |
| **Menthol** | mg/mg nicotine | 7.21E-01 | 7.09E-01 | 6.87E-01 | 7.18E-01 | 6.97E-01 | 7.21E-01 |
| **Diethylene Glycol** | mg/mg nicotine | ~ 4.67E-03 | ~ 4.57E-03 | ~ 4.62E-03 | ~ 4.19E-03 | ~ 4.61E-03 | ~ 4.67E-03 |
| **Ethylene Glycol** | mg/mg nicotine | ~ 4.97E-03 | ~ 4.89E-03 | ~ 4.93E-03 | ~ 4.48E-03 | ~ 4.93E-03 | ~ 4.97E-03 |
| **Carbonyls & Glycidol** | | | | | | | |
| **Acetaldehyde** | mg/mg nicotine | 1.54E-04 | 1.30E-04 | 1.47E-04 | 2.18E-04 | 2.23E-04 | 2.23E-04 |

| | | | | | | | |
|---|---|---|---|---|---|---|---|
| **Acrolein** | mg/mg nicotine | ~ 4.03E-05 | ~ 4.03E-05 | ~ 3.59E-05 | 8.46E-05 | 2.20E-04 | 2.20E-04 |
| **Diacetyl** | mg/mg nicotine | ~ 4.10E-05 | ~ 4.11E-05 | ~ 3.45E-05 | ~ 3.66E-05 | ~ 4.28E-05 | ~ 4.28E-05 |
| **Formaldehyde** | mg/mg nicotine | 7.76E-04 | 7.91E-04 | 7.23E-04 | 1.04E-03 | 7.74E-04 | 1.04E-03 |
| **Glycidol** | mg/mg nicotine | 1.63E-04 | 1.67E-04 | 2.80E-04 | 1.38E-04 | 1.47E-04 | 2.80E-04 |
| **Acetyl Propionyl** | mg/mg nicotine | ~ 3.59E-06 | ~ 3.58E-06 | ~ 3.17E-06 | ~ 3.35E-06 | ~ 4.96E-06 | ~ 4.96E-06 |
| **Butyraldehyde** | mg/mg nicotine | ~ 3.96E-06 | ~ 3.96E-06 | ~ 3.16E-06 | ~ 3.35E-06 | ~ 8.46E-06 | ~ 8.46E-06 |
| **Crotonaldehyde** | mg/mg nicotine | ~ 3.63E-06 | ~ 3.62E-06 | ~ 5.07E-06 | ~ 5.38E-06 | ~ 1.01E-05 | ~ 1.01E-05 |
| **Furfural** | mg/mg nicotine | ~ 5.02E-06 | ~ 5.01E-06 | ~ 5.19E-06 | ~ 5.50E-06 | ~ 8.32E-06 | ~ 8.32E-06 |
| **Nicotine Degradants & TSNAs** | | | | | | | |
| **β-Nicotyrine** | mg/mg nicotine | 1.26E-04 | 1.52E-04 | 1.94E-04 | 2.09E-04 | 2.03E-04 | 2.09E-04 |
| **Cotinine** | mg/mg nicotine | ~ 3.19E-05 | ~ 3.19E-05 | ~ 2.79E-05 | 6.08E-05 | ~ 3.18E-05 | 6.08E-05 |
| **Myosmine** | mg/mg nicotine | 1.04E-04 | 1.26E-04 | 1.44E-04 | 1.58E-04 | 1.46E-04 | 1.58E-04 |
| **Nicotine N Oxide** | mg/mg nicotine | 6.36E-05 | ~ 3.94E-05 | ~ 2.78E-05 | 6.01E-05 | 5.90E-05 | 6.36E-05 |
| **Nornicotine** | mg/mg nicotine | 4.48E-04 | 5.98E-04 | 7.51E-04 | 7.68E-04 | 7.12E-04 | 7.68E-04 |
| **Anabasine** | mg/mg nicotine | ~ 2.98E-06 | ~ 2.97E-06 | ~ 2.54E-06 | ~ 2.70E-06 | ~ 2.90E-06 | ~ 2.98E-06 |
| **Anatabine** | mg/mg nicotine | ~ 2.97E-06 | ~ 2.96E-06 | ~ 2.53E-06 | ~ 2.68E-06 | ~ 2.89E-06 | ~ 2.97E-06 |
| **NNK** | mg/mg nicotine | ~ 7.64E-07 | NT | NT | NT | NT | ~ 7.64E-07 |
| **NNN** | mg/mg nicotine | ~ 9.18E-08 | NT | NT | NT | NT | ~ 9.18E-08 |
| **Metals** | | | | | | | |
| **Arsenic** | mg/mg nicotine | ~ 2.78E-07 | ~ 2.77E-07 | ~ 2.80E-07 | ~ 2.54E-07 | ~ 2.74E-07 | ~ 2.80E-07 |
| **Beryllium** | mg/mg nicotine | ~ 6.11E-08 | ~ 6.10E-08 | ~ 6.16E-08 | ~ 5.59E-08 | ~ 6.02E-08 | ~ 6.16E-08 |
| **Cadmium** | mg/mg nicotine | ~ 2.78E-08 | ~ 2.77E-08 | ~ 2.80E-08 | ~ 2.54E-08 | ~ 2.74E-08 | ~ 2.80E-08 |
| **Chromium** | mg/mg nicotine | ~ 1.67E-06 | ~ 2.77E-07 | ~ 2.80E-07 | ~ 2.54E-07 | ~ 2.74E-07 | ~ 1.67E-06 |
| **Cobalt** | mg/mg nicotine | ~ 1.39E-08 | ~ 1.39E-08 | ~ 1.40E-08 | ~ 1.27E-08 | ~ 1.37E-08 | ~ 1.40E-08 |
| **Copper** | mg/mg nicotine | ~ 6.95E-07 | ~ 6.94E-07 | ~ 7.00E-07 | ~ 6.36E-07 | ~ 6.85E-07 | ~ 7.00E-07 |
| **Gold** | mg/mg nicotine | ~ 4.72E-06 | NT | NT | NT | NT | ~ 4.72E-06 |
| **Iron** | mg/mg nicotine | 1.23E-05 | ~ 2.77E-06 | ~ 2.80E-06 | ~ 2.54E-06 | ~ 2.74E-06 | 1.23E-05 |
| **Lead** | mg/mg nicotine | ~ 6.95E-08 | ~ 6.94E-08 | ~ 7.00E-08 | ~ 6.36E-08 | ~ 6.85E-08 | ~ 7.00E-08 |
| **Nickel** | mg/mg nicotine | ~ 1.39E-07 | ~ 1.39E-07 | ~ 1.40E-07 | ~ 1.27E-07 | ~ 1.37E-07 | ~ 1.40E-07 |

| | | | | | | | |
|---|---|---|---|---|---|---|---|
| **Selenium** | mg/mg nicotine | ~ 6.64E-07 | ~ 6.63E-07 | ~ 6.69E-07 | ~ 6.08E-07 | ~ 6.54E-07 | ~ 6.69E-07 |
| **Silver** | mg/mg nicotine | ~ 2.78E-08 | ~ 1.66E-07 | ~ 2.80E-08 | ~ 2.54E-08 | ~ 2.74E-08 | ~ 1.66E-07 |
| **Tin** | mg/mg nicotine | ~ 1.67E-06 | ~ 1.66E-06 | ~ 1.68E-06 | ~ 1.53E-06 | ~ 2.74E-07 | ~ 1.68E-06 |
| **Zinc** | mg/mg nicotine | ~ 4.17E-06 | ~ 4.16E-06 | ~ 4.20E-06 | ~ 3.81E-06 | ~ 4.11E-06 | ~ 4.20E-06 |
| **Organic Acids** | | | | | | | |
| **Benzoic Acid** | mg/mg nicotine | 7.34E-01 | 6.82E-01 | 5.36E-01 | 6.12E-01 | 6.29E-01 | 7.34E-01 |
| **Propionic Acid** | mg/mg nicotine | ~ 1.72E-04 | ~ 1.28E-04 | ~ 9.87E-05 | ~ 9.87E-04 | ~ 1.06E-03 | ~ 1.06E-03 |
| **VOCs** | | | | | | | |
| **1,3-Butadiene** | mg/mg nicotine | ~ 1.53E-06 | NT | NT | NT | NT | ~ 1.53E-06 |
| **Acrylonitrile** | mg/mg nicotine | ~ 9.99E-07 | NT | NT | NT | NT | ~ 9.99E-07 |
| **Benzene** | mg/mg nicotine | ~ 1.75E-06 | NT | NT | NT | NT | ~ 1.75E-06 |
| **Isoprene** | mg/mg nicotine | ~ 2.35E-06 | NT | NT | NT | NT | ~ 2.35E-06 |
| **Propylene Oxide** | mg/mg nicotine | ~ 4.05E-06 | NT | NT | NT | NT | ~ 4.05E-06 |
| **Toluene** | mg/mg nicotine | ~ 1.24E-05 | NT | NT | NT | NT | ~ 1.24E-05 |
| **Esters & Alcohols** | | | | | | | |
| **1-Butanol** | mg/mg nicotine | ~ 4.50E-05 | NT | NT | NT | NT | ~ 4.50E-05 |
| **Benzyl Acetate** | mg/mg nicotine | ~ 6.98E-05 | NT | NT | NT | NT | ~ 6.98E-05 |
| **Ethyl Acetate** | mg/mg nicotine | 6.60E-03 | NT | NT | NT | NT | 6.60E-03 |
| **Ethyl Acetoacetate** | mg/mg nicotine | ~ 5.71E-05 | NT | NT | NT | NT | ~ 5.71E-05 |
| **Isoamyl Acetate** | mg/mg nicotine | ~ 4.87E-05 | NT | NT | NT | NT | ~ 4.87E-05 |
| **Isobutyl Acetate** | mg/mg nicotine | ~ 4.82E-05 | NT | NT | NT | NT | ~ 4.82E-05 |
| **Methyl Acetate** | mg/mg nicotine | ~ 5.19E-05 | NT | NT | NT | NT | ~ 5.19E-05 |

~ = approximated numeric value (see Supplemental Materials Appendix 1A) generated from the corresponding LOD and LOQ (See Supplemental Materials Appendix 1B)

## 1.5 Polar Menthol 18 mg/mL Non-Intense Nicotine Normalized Analytical Stability Results

| Parameter/ Constituent | Units | T0 | T3 | T6 | T9 | T12 | Max |
|---|---|---|---|---|---|---|---|

| | | | | | | | |
|---|---|---|---|---|---|---|---|
| **# of puffs** | NA | 100 | 132 | 115 | 122 | 106 | 132 |
| **pH** | NA | 6.31 | 6.55 | 6.32 | 6.38 | 6.54 | 6.55 |
| **Primary Constituents** | | | | | | | |
| **Nicotine** | mg/mg nicotine | 1.00E+00 | 1.00E+00 | 1.00E+00 | 1.00E+00 | 1.00E+00 | 1.00E+00 |
| **Glycerol** | mg/mg nicotine | 2.56E+01 | 2.72E+01 | 2.66E+01 | 2.67E+01 | 2.71E+01 | 2.72E+01 |
| **Propylene Glycol** | mg/mg nicotine | 3.54E+01 | 3.84E+01 | 4.50E+01 | 3.78E+01 | 3.72E+01 | 4.50E+01 |
| **Water** | mg/mg nicotine | 3.66E+00 | 4.77E+00 | 4.43E+00 | 3.84E+00 | 4.92E+00 | 4.92E+00 |
| **Menthol** | mg/mg nicotine | 7.72E-01 | 7.65E-01 | 7.67E-01 | 7.88E-01 | 7.57E-01 | 7.88E-01 |
| **Diethylene Glycol** | mg/mg nicotine | ~ 5.24E-03 | ~ 4.65E-03 | ~ 4.98E-03 | ~ 4.27E-03 | ~ 5.53E-03 | ~ 5.53E-03 |
| **Ethylene Glycol** | mg/mg nicotine | ~ 5.58E-03 | ~ 4.97E-03 | ~ 5.32E-03 | ~ 4.56E-03 | ~ 5.92E-03 | ~ 5.92E-03 |
| **Carbonyls & Glycidol** | | | | | | | |
| **Acetaldehyde** | mg/mg nicotine | ~ 4.06E-05 | 1.76E-04 | 1.56E-04 | 1.90E-04 | 2.90E-04 | 2.90E-04 |
| **Acrolein** | mg/mg nicotine | ~ 3.88E-05 | ~ 3.51E-05 | ~ 3.87E-05 | ~ 3.32E-05 | 1.28E-04 | 1.28E-04 |
| **Diacetyl** | mg/mg nicotine | ~ 3.87E-06 | ~ 3.50E-06 | ~ 3.46E-06 | ~ 2.97E-06 | ~ 3.77E-06 | ~ 3.87E-06 |
| **Formaldehyde** | mg/mg nicotine | 3.18E-04 | 6.73E-04 | 5.43E-04 | 6.40E-04 | 8.77E-04 | 8.77E-04 |
| **Glycidol** | mg/mg nicotine | 1.14E-04 | 1.31E-04 | 1.44E-04 | 1.41E-04 | 1.64E-04 | 1.64E-04 |
| **Acetyl Propionyl** | mg/mg nicotine | ~ 3.45E-06 | ~ 3.12E-06 | ~ 3.41E-06 | ~ 2.93E-06 | ~ 3.72E-06 | ~ 3.72E-06 |
| **Butyraldehyde** | mg/mg nicotine | ~ 3.82E-06 | ~ 3.45E-06 | ~ 3.41E-06 | ~ 2.92E-06 | ~ 3.71E-06 | ~ 3.82E-06 |
| **Crotonaldehyde** | mg/mg nicotine | ~ 3.49E-06 | ~ 3.16E-06 | ~ 5.47E-06 | ~ 4.69E-06 | ~ 5.96E-06 | ~ 5.96E-06 |
| **Furfural** | mg/mg nicotine | ~ 4.83E-06 | ~ 4.37E-06 | ~ 5.60E-06 | ~ 4.80E-06 | ~ 6.10E-06 | ~ 6.10E-06 |
| **Nicotine Degradants & TSNAs** | | | | | | | |
| **β-Nicotyrine** | mg/mg nicotine | ~ 3.08E-05 | 1.41E-04 | 1.45E-04 | 2.75E-04 | 3.08E-04 | 3.08E-04 |
| **Cotinine** | mg/mg nicotine | ~ 3.07E-05 | 8.76E-05 | 8.64E-05 | 6.74E-05 | 1.57E-04 | 1.57E-04 |
| **Myosmine** | mg/mg nicotine | 8.57E-05 | 2.12E-04 | 2.31E-04 | 2.76E-04 | 4.00E-04 | 4.00E-04 |
| **Nicotine N Oxide** | mg/mg nicotine | ~ 3.80E-05 | 7.32E-05 | 1.17E-04 | 9.71E-05 | 6.25E-05 | 1.17E-04 |
| **Nornicotine** | mg/mg nicotine | 1.34E-04 | 4.23E-04 | 5.18E-04 | 6.30E-04 | 9.36E-04 | 9.36E-04 |
| **Anabasine** | mg/mg nicotine | ~ 2.86E-06 | ~ 2.59E-06 | ~ 2.74E-06 | ~ 2.35E-06 | ~ 2.99E-06 | ~ 2.99E-06 |
| **Anatabine** | mg/mg nicotine | ~ 3.11E-05 | ~ 2.81E-05 | ~ 3.00E-05 | ~ 2.58E-05 | ~ 3.27E-05 | ~ 3.27E-05 |
| **NNK** | mg/mg nicotine | ~ 8.58E-07 | NT | NT | NT | NT | ~ 8.58E-07 |

| | | | | | | | |
|---|---|---|---|---|---|---|---|
| **NNN** | mg/mg nicotine | ~ 1.03E-07 | NT | NT | NT | NT | ~ 1.03E-07 |
| **Metals** | | | | | | | |
| **Arsenic** | mg/mg nicotine | ~ 3.12E-07 | ~ 1.69E-06 | ~ 1.81E-06 | ~ 1.55E-06 | ~ 3.29E-07 | ~ 1.81E-06 |
| **Beryllium** | mg/mg nicotine | ~ 6.87E-08 | ~ 6.21E-08 | ~ 6.64E-08 | ~ 5.70E-08 | ~ 7.23E-08 | ~ 7.23E-08 |
| **Cadmium** | mg/mg nicotine | ~ 3.12E-08 | ~ 2.82E-08 | ~ 3.02E-08 | ~ 2.59E-08 | ~ 3.29E-08 | ~ 3.29E-08 |
| **Chromium** | mg/mg nicotine | ~ 1.87E-06 | ~ 2.82E-07 | ~ 3.02E-07 | ~ 2.59E-07 | ~ 3.29E-07 | ~ 1.87E-06 |
| **Cobalt** | mg/mg nicotine | ~ 1.56E-08 | ~ 1.41E-08 | ~ 1.51E-08 | ~ 1.29E-08 | ~ 1.64E-08 | ~ 1.64E-08 |
| **Copper** | mg/mg nicotine | ~ 3.12E-06 | ~ 7.06E-07 | ~ 3.02E-06 | ~ 6.47E-07 | ~ 3.29E-06 | ~ 3.29E-06 |
| **Gold** | mg/mg nicotine | ~ 5.31E-06 | NT | NT | NT | NT | ~ 5.31E-06 |
| **Iron** | mg/mg nicotine | ~ 9.36E-06 | ~ 2.82E-06 | ~ 3.02E-06 | ~ 2.59E-06 | ~ 3.29E-06 | ~ 9.36E-06 |
| **Lead** | mg/mg nicotine | ~ 7.80E-08 | ~ 7.06E-08 | ~ 7.55E-08 | ~ 6.47E-08 | ~ 8.22E-08 | ~ 8.22E-08 |
| **Nickel** | mg/mg nicotine | ~ 1.56E-07 | ~ 4.23E-07 | ~ 4.53E-07 | ~ 1.29E-07 | ~ 1.64E-07 | ~ 4.53E-07 |
| **Selenium** | mg/mg nicotine | ~ 7.46E-07 | ~ 6.75E-07 | 1.21E-06 | ~ 6.19E-07 | ~ 7.86E-07 | 1.21E-06 |
| **Silver** | mg/mg nicotine | ~ 3.12E-08 | ~ 2.82E-08 | ~ 3.02E-08 | ~ 2.59E-08 | ~ 3.29E-08 | ~ 3.29E-08 |
| **Tin** | mg/mg nicotine | ~ 3.12E-07 | ~ 2.82E-07 | ~ 3.02E-07 | ~ 2.59E-07 | ~ 3.29E-07 | ~ 3.29E-07 |
| **Zinc** | mg/mg nicotine | ~ 1.56E-05 | ~ 4.23E-06 | ~ 4.53E-06 | ~ 3.88E-06 | ~ 4.93E-06 | ~ 1.56E-05 |
| **Organic Acids** | | | | | | | |
| **Benzoic Acid** | mg/mg nicotine | 5.08E-01 | 5.75E-01 | 5.81E-01 | 5.21E-01 | 5.46E-01 | 5.81E-01 |
| **Propionic Acid** | mg/mg nicotine | ~ 1.10E-04 | ~ 9.96E-05 | ~ 1.33E-03 | ~ 1.01E-03 | ~ 2.03E-04 | ~ 1.33E-03 |
| **VOCs** | | | | | | | |
| **1,3-Butadiene** | mg/mg nicotine | ~ 1.71E-06 | NT | NT | NT | NT | ~ 1.71E-06 |
| **Acrylonitrile** | mg/mg nicotine | ~ 1.12E-06 | NT | NT | NT | NT | ~ 1.12E-06 |
| **Benzene** | mg/mg nicotine | ~ 1.97E-06 | NT | NT | NT | NT | ~ 1.97E-06 |
| **Isoprene** | mg/mg nicotine | ~ 2.64E-06 | NT | NT | NT | NT | ~ 2.64E-06 |
| **Propylene Oxide** | mg/mg nicotine | ~ 4.54E-06 | NT | NT | NT | NT | ~ 4.54E-06 |
| **Toluene** | mg/mg nicotine | ~ 2.89E-06 | NT | NT | NT | NT | ~ 2.89E-06 |
| **Esters & Alcohols** | | | | | | | |
| **1-Butanol** | mg/mg nicotine | ~ 5.06E-05 | NT | NT | NT | NT | ~ 5.06E-05 |

| | | | | | | | |
|---|---|---|---|---|---|---|---|
| **Benzyl Acetate** | mg/mg nicotine | ~ 7.84E-05 | NT | NT | NT | NT | ~ 7.84E-05 |
| **Ethyl Acetate** | mg/mg nicotine | ~ 9.87E-04 | NT | NT | NT | NT | ~ 9.87E-04 |
| **Ethyl Acetoacetate** | mg/mg nicotine | ~ 6.41E-05 | NT | NT | NT | NT | ~ 6.41E-05 |
| **Isoamyl Acetate** | mg/mg nicotine | ~ 5.47E-05 | NT | NT | NT | NT | ~ 5.47E-05 |
| **Isobutyl Acetate** | mg/mg nicotine | ~ 5.42E-05 | NT | NT | NT | NT | ~ 5.42E-05 |
| **Methyl Acetate** | mg/mg nicotine | ~ 5.82E-05 | NT | NT | NT | NT | ~ 5.82E-05 |

~ = approximated numeric value (see Supplemental Materials Appendix 1A) generated from the corresponding LOD and LOQ (See Supplemental Materials Appendix 1B)

## 1.6 Polar Menthol 18 mg/mL Intense Nicotine Normalized Analytical Stability Results

| Parameter/ Constituent | Units | T0 | T3 | T6 | T9 | T12 | Max |
|---|---|---|---|---|---|---|---|
| **# of puffs** | NA | 81 | 81 | 81 | 81 | 80 | 81 |
| **pH** | NA | 6.24 | 6.57 | 6.36 | 6.22 | 6.42 | 6.57 |
| **Primary Constituents** | | | | | | | |
| **Nicotine** | mg/mg nicotine | 1.00E+00 | 1.00E+00 | 1.00E+00 | 1.00E+00 | 1.00E+00 | 1.00E+00 |
| **Glycerol** | mg/mg nicotine | 2.56E+01 | 2.73E+01 | 2.66E+01 | 2.69E+01 | 2.74E+01 | 2.74E+01 |
| **Propylene Glycol** | mg/mg nicotine | 3.53E+01 | 4.04E+01 | 3.76E+01 | 3.79E+01 | 3.75E+01 | 4.04E+01 |
| **Water** | mg/mg nicotine | 4.06E+00 | 4.00E+00 | 4.08E+00 | 3.61E+00 | 4.21E+00 | 4.21E+00 |
| **Menthol** | mg/mg nicotine | 7.67E-01 | 7.58E-01 | 7.27E-01 | 7.70E-01 | 7.37E-01 | 7.70E-01 |
| **Diethylene Glycol** | mg/mg nicotine | ~ 4.71E-03 | ~ 4.65E-03 | ~ 4.69E-03 | ~ 4.22E-03 | ~ 4.67E-03 | ~ 4.71E-03 |
| **Ethylene Glycol** | mg/mg nicotine | ~ 5.01E-03 | ~ 4.98E-03 | ~ 5.02E-03 | ~ 4.51E-03 | ~ 5.00E-03 | ~ 5.02E-03 |
| **Carbonyls & Glycidol** | | | | | | | |
| **Acetaldehyde** | mg/mg nicotine | 1.11E-04 | 1.51E-04 | 1.82E-04 | 2.38E-04 | 3.14E-04 | 3.14E-04 |
| **Acrolein** | mg/mg nicotine | 9.22E-05 | 9.07E-05 | ~ 4.26E-05 | 7.38E-05 | 2.79E-04 | 2.79E-04 |
| **Diacetyl** | mg/mg nicotine | ~ 4.06E-06 | ~ 4.18E-05 | ~ 4.19E-05 | ~ 3.42E-06 | ~ 4.33E-05 | ~ 4.33E-05 |
| **Formaldehyde** | mg/mg nicotine | 6.67E-04 | 7.61E-04 | 7.57E-04 | 8.64E-04 | 8.65E-04 | 8.65E-04 |
| **Glycidol** | mg/mg nicotine | 2.06E-04 | 1.75E-04 | 1.91E-04 | 1.17E-04 | 1.98E-04 | 2.06E-04 |

| | | | | | | | |
|---|---|---|---|---|---|---|---|
| **Acetyl Propionyl** | mg/mg nicotine | ~ 3.62E-06 | ~ 3.64E-06 | ~ 3.76E-06 | ~ 3.37E-06 | ~ 5.03E-06 | ~ 5.03E-06 |
| **Butyraldehyde** | mg/mg nicotine | ~ 4.00E-06 | ~ 4.03E-06 | ~ 3.75E-06 | ~ 3.37E-06 | ~ 8.57E-06 | ~ 8.57E-06 |
| **Crotonaldehyde** | mg/mg nicotine | ~ 3.66E-06 | ~ 3.69E-06 | ~ 6.02E-06 | ~ 5.41E-06 | ~ 1.02E-05 | ~ 1.02E-05 |
| **Furfural** | mg/mg nicotine | ~ 5.07E-06 | ~ 5.10E-06 | ~ 6.16E-06 | ~ 5.53E-06 | ~ 8.43E-06 | ~ 8.43E-06 |
| **Nicotine Degradants & TSNAs** | | | | | | | |
| **β-Nicotyrine** | mg/mg nicotine | ~ 3.23E-05 | 1.39E-04 | 1.79E-04 | 3.38E-04 | 2.55E-04 | 3.38E-04 |
| **Cotinine** | mg/mg nicotine | ~ 3.22E-05 | 5.96E-05 | 7.76E-05 | 1.01E-04 | 1.15E-04 | 1.15E-04 |
| **Myosmine** | mg/mg nicotine | 1.22E-04 | 2.15E-04 | 2.82E-04 | 2.92E-04 | 3.28E-04 | 3.28E-04 |
| **Nicotine N Oxide** | mg/mg nicotine | ~ 3.99E-05 | ~ 4.01E-05 | ~ 3.30E-05 | ~ 2.96E-05 | ~ 3.21E-05 | ~ 4.01E-05 |
| **Nornicotine** | mg/mg nicotine | 1.48E-04 | 3.79E-04 | 4.78E-04 | 6.46E-04 | 7.08E-04 | 7.08E-04 |
| **Anabasine** | mg/mg nicotine | ~ 3.00E-06 | ~ 3.02E-06 | ~ 3.02E-06 | ~ 2.98E-05 | ~ 2.94E-06 | ~ 2.98E-05 |
| **Anatabine** | mg/mg nicotine | ~ 3.26E-05 | ~ 3.28E-05 | ~ 3.31E-05 | ~ 2.97E-05 | ~ 3.22E-05 | ~ 3.31E-05 |
| **NNK** | mg/mg nicotine | ~ 7.71E-07 | NT | NT | NT | NT | ~ 7.71E-07 |
| **NNN** | mg/mg nicotine | ~ 9.26E-08 | NT | NT | NT | NT | ~ 9.26E-08 |
| **Metals** | | | | | | | |
| **Arsenic** | mg/mg nicotine | ~ 2.81E-07 | ~ 2.82E-07 | ~ 1.71E-06 | ~ 2.56E-07 | ~ 2.77E-07 | ~ 1.71E-06 |
| **Beryllium** | mg/mg nicotine | ~ 6.17E-08 | ~ 6.21E-08 | ~ 6.26E-08 | ~ 5.63E-08 | ~ 6.10E-08 | ~ 6.26E-08 |
| **Cadmium** | mg/mg nicotine | ~ 2.81E-08 | ~ 2.82E-08 | ~ 2.85E-08 | ~ 2.56E-08 | ~ 2.77E-08 | ~ 2.85E-08 |
| **Chromium** | mg/mg nicotine | ~ 1.68E-06 | ~ 2.82E-07 | ~ 2.85E-07 | ~ 2.56E-07 | ~ 1.66E-06 | ~ 1.68E-06 |
| **Cobalt** | mg/mg nicotine | ~ 1.40E-08 | ~ 1.41E-08 | ~ 1.42E-08 | ~ 1.28E-08 | ~ 1.39E-08 | ~ 1.42E-08 |
| **Copper** | mg/mg nicotine | ~ 7.01E-07 | ~ 7.06E-07 | ~ 7.12E-07 | ~ 6.39E-07 | ~ 6.94E-07 | ~ 7.12E-07 |
| **Gold** | mg/mg nicotine | ~ 4.77E-06 | NT | NT | NT | NT | ~ 4.77E-06 |
| **Iron** | mg/mg nicotine | ~ 8.42E-06 | ~ 2.82E-06 | ~ 2.85E-06 | ~ 2.56E-06 | ~ 2.77E-06 | ~ 8.42E-06 |
| **Lead** | mg/mg nicotine | ~ 7.01E-08 | ~ 7.06E-08 | ~ 7.12E-08 | ~ 6.39E-08 | ~ 6.94E-08 | ~ 7.12E-08 |
| **Nickel** | mg/mg nicotine | ~ 1.40E-07 | ~ 4.23E-07 | ~ 1.42E-07 | ~ 1.28E-07 | ~ 1.39E-07 | ~ 4.23E-07 |
| **Selenium** | mg/mg nicotine | ~ 6.70E-07 | ~ 6.75E-07 | 1.40E-06 | ~ 6.11E-07 | ~ 6.63E-07 | 1.40E-06 |
| **Silver** | mg/mg nicotine | ~ 2.81E-08 | ~ 1.69E-07 | ~ 2.85E-08 | ~ 2.56E-08 | ~ 2.77E-08 | ~ 1.69E-07 |
| **Tin** | mg/mg nicotine | ~ 2.81E-07 | ~ 1.69E-06 | ~ 1.71E-06 | ~ 1.53E-06 | ~ 2.77E-07 | ~ 1.71E-06 |

| | | | | | | | |
|---|---|---|---|---|---|---|---|
| **Zinc** | mg/mg nicotine | ~ 4.21E-06 | ~ 4.23E-06 | ~ 1.42E-05 | ~ 3.84E-06 | ~ 4.16E-06 | ~ 1.42E-05 |
| **Organic Acids** | | | | | | | |
| **Benzoic Acid** | mg/mg nicotine | 7.22E-01 | 7.14E-01 | 6.36E-01 | 6.35E-01 | 6.00E-01 | 7.22E-01 |
| **Propionic Acid** | mg/mg nicotine | ~ 9.90E-05 | ~ 1.10E-03 | ~ 1.76E-03 | ~ 9.93E-04 | ~ 1.72E-04 | ~ 1.76E-03 |
| **VOCs** | | | | | | | |
| **1,3-Butadiene** | mg/mg nicotine | ~ 1.54E-06 | NT | NT | NT | NT | ~ 1.54E-06 |
| **Acrylonitrile** | mg/mg nicotine | ~ 1.01E-06 | NT | NT | NT | NT | ~ 1.01E-06 |
| **Benzene** | mg/mg nicotine | ~ 1.77E-06 | NT | NT | NT | NT | ~ 1.77E-06 |
| **Isoprene** | mg/mg nicotine | ~ 2.37E-06 | NT | NT | NT | NT | ~ 2.37E-06 |
| **Propylene Oxide** | mg/mg nicotine | ~ 4.08E-06 | NT | NT | NT | NT | ~ 4.08E-06 |
| **Toluene** | mg/mg nicotine | ~ 2.60E-06 | NT | NT | NT | NT | ~ 2.60E-06 |
| **Esters & Alcohols** | | | | | | | |
| **1-Butanol** | mg/mg nicotine | ~ 4.54E-05 | NT | NT | NT | NT | ~ 4.54E-05 |
| **Benzyl Acetate** | mg/mg nicotine | ~ 7.05E-05 | NT | NT | NT | NT | ~ 7.05E-05 |
| **Ethyl Acetate** | mg/mg nicotine | ~ 8.88E-04 | NT | NT | NT | NT | ~ 8.88E-04 |
| **Ethyl Acetoacetate** | mg/mg nicotine | ~ 5.76E-05 | NT | NT | NT | NT | ~ 5.76E-05 |
| **Isoamyl Acetate** | mg/mg nicotine | ~ 4.92E-05 | NT | NT | NT | NT | ~ 4.92E-05 |
| **Isobutyl Acetate** | mg/mg nicotine | ~ 4.87E-05 | NT | NT | NT | NT | ~ 4.87E-05 |
| **Methyl Acetate** | mg/mg nicotine | ~ 5.24E-05 | NT | NT | NT | NT | ~ 5.24E-05 |

~ = approximated numeric value (see Supplemental Materials Appendix 1A) generated from the corresponding LOD and LOQ (See Supplemental Materials Appendix 1B)

## 1.7 Autumn Tobacco 18 mg/mL Non-Intense Nicotine Normalized Analytical Stability Results

| Parameter/ Constituent | Units | T0 | T3 | T6 | T9 | T12 | Max |
|---|---|---|---|---|---|---|---|
| **# of puffs** | NA | 128 | 128 | 119 | 122 | 124 | 128 |
| **pH** | NA | 6.36 | 6.16 | 6.37 | 6.41 | 6.14 | 6.41 |
| **Primary Constituents** | | | | | | | |

| | | | | | | | |
|---|---|---|---|---|---|---|---|
| **Nicotine** | mg/mg nicotine | 1.00E+00 | 1.00E+00 | 1.00E+00 | 1.00E+00 | 1.00E+00 | 1.00E+00 |
| **Glycerol** | mg/mg nicotine | 2.59E+01 | 2.58E+01 | 2.48E+01 | 2.44E+01 | 2.45E+01 | 2.59E+01 |
| **Propylene Glycol** | mg/mg nicotine | 3.40E+01 | 3.32E+01 | 3.16E+01 | 3.17E+01 | 3.32E+01 | 3.40E+01 |
| **Water** | mg/mg nicotine | 3.23E+00 | 4.48E+00 | 4.18E+00 | 4.44E+00 | 6.24E+00 | 6.24E+00 |
| **Menthol** | mg/mg nicotine | ~ 1.46E-03 | ~ 1.46E-03 | ~ 4.33E-04 | ~ 4.09E-04 | ~ 2.01E-03 | ~ 2.01E-03 |
| **Diethylene Glycol** | mg/mg nicotine | ~ 1.34E-03 | ~ 1.34E-03 | ~ 3.97E-04 | ~ 3.76E-04 | ~ 1.84E-03 | ~ 1.84E-03 |
| **Ethylene Glycol** | mg/mg nicotine | ~ 9.36E-04 | ~ 9.31E-04 | ~ 2.77E-04 | ~ 2.62E-04 | ~ 1.28E-03 | ~ 1.28E-03 |
| **Carbonyls & Glycidol** | | | | | | | |
| **Acetaldehyde** | mg/mg nicotine | 1.03E-04 | 1.37E-04 | 1.60E-04 | 1.56E-04 | 1.43E-04 | 1.60E-04 |
| **Acrolein** | mg/mg nicotine | 7.06E-05 | ~ 3.88E-05 | ~ 3.86E-05 | ~ 3.65E-05 | ~ 3.54E-05 | 7.06E-05 |
| **Diacetyl** | mg/mg nicotine | ~ 3.48E-06 | ~ 3.73E-05 | ~ 3.43E-06 | ~ 3.52E-05 | ~ 3.57E-05 | ~ 3.73E-05 |
| **Formaldehyde** | mg/mg nicotine | 5.57E-04 | 4.40E-04 | 4.68E-04 | 4.97E-04 | 5.39E-04 | 5.57E-04 |
| **Glycidol** | mg/mg nicotine | 1.15E-04 | 1.31E-04 | 1.23E-04 | 8.56E-05 | 1.61E-04 | 1.61E-04 |
| **Acetyl Propionyl** | mg/mg nicotine | ~ 3.43E-06 | ~ 3.42E-06 | ~ 3.39E-06 | ~ 3.20E-06 | ~ 4.29E-06 | ~ 4.29E-06 |
| **Butyraldehyde** | mg/mg nicotine | ~ 3.43E-06 | ~ 3.42E-06 | ~ 3.38E-06 | ~ 3.52E-05 | ~ 3.44E-06 | ~ 3.52E-05 |
| **Crotonaldehyde** | mg/mg nicotine | ~ 5.51E-06 | ~ 5.48E-06 | ~ 5.43E-06 | ~ 5.13E-06 | ~ 4.24E-06 | ~ 5.51E-06 |
| **Furfural** | mg/mg nicotine | ~ 5.63E-06 | ~ 5.61E-06 | ~ 5.56E-06 | ~ 5.25E-06 | ~ 5.88E-06 | ~ 5.88E-06 |
| **Nicotine Degradants & TSNAs** | | | | | | | |
| **β-Nicotyrine** | mg/mg nicotine | 5.68E-05 | ~ 3.00E-05 | 1.17E-04 | 6.83E-05 | 1.02E-04 | 1.17E-04 |
| **Cotinine** | mg/mg nicotine | 8.29E-05 | ~ 3.00E-05 | 7.51E-05 | 1.89E-04 | 9.78E-05 | 1.89E-04 |
| **Myosmine** | mg/mg nicotine | 1.16E-04 | 1.52E-04 | 1.72E-04 | 1.82E-04 | 1.88E-04 | 1.88E-04 |
| **Nicotine N Oxide** | mg/mg nicotine | 8.49E-05 | ~ 3.00E-05 | 8.41E-05 | 2.51E-04 | 7.43E-05 | 2.51E-04 |
| **Nornicotine** | mg/mg nicotine | 2.14E-04 | 2.67E-04 | 4.69E-04 | 5.91E-04 | 5.53E-04 | 5.91E-04 |
| **Anabasine** | mg/mg nicotine | ~ 2.76E-06 | ~ 3.09E-06 | ~ 2.72E-06 | ~ 2.57E-06 | ~ 3.69E-06 | ~ 3.69E-06 |
| **Anatabine** | mg/mg nicotine | ~ 2.75E-06 | ~ 2.76E-06 | ~ 2.71E-06 | ~ 2.56E-06 | ~ 2.93E-06 | ~ 2.93E-06 |
| **NNK** | mg/mg nicotine | ~ 9.18E-08 | ~ 9.14E-08 | NT | NT | NT | ~ 9.18E-08 |
| **NNN** | mg/mg nicotine | ~ 2.47E-08 | ~ 2.46E-08 | NT | NT | NT | ~ 2.47E-08 |
| **Metals** | | | | | | | |

| | | | | | | | |
|---|---|---|---|---|---|---|---|
| **Arsenic** | mg/mg nicotine | ~ 1.82E-06 | ~ 3.02E-07 | ~ 1.80E-06 | ~ 2.83E-07 | ~ 2.78E-07 | ~ 1.82E-06 |
| **Beryllium** | mg/mg nicotine | ~ 6.68E-08 | ~ 6.65E-08 | ~ 6.59E-08 | ~ 6.23E-08 | ~ 6.11E-08 | ~ 6.68E-08 |
| **Cadmium** | mg/mg nicotine | ~ 3.04E-08 | ~ 3.02E-08 | ~ 3.00E-08 | ~ 2.83E-08 | ~ 2.78E-08 | ~ 3.04E-08 |
| **Chromium** | mg/mg nicotine | ~ 3.04E-07 | ~ 3.02E-07 | ~ 1.80E-06 | ~ 2.83E-07 | ~ 2.78E-07 | ~ 1.80E-06 |
| **Cobalt** | mg/mg nicotine | ~ 1.52E-08 | ~ 1.51E-08 | ~ 1.50E-08 | ~ 1.42E-08 | ~ 1.39E-08 | ~ 1.52E-08 |
| **Copper** | mg/mg nicotine | ~ 7.59E-07 | ~ 7.56E-07 | ~ 7.49E-07 | ~ 7.08E-07 | ~ 6.95E-07 | ~ 7.59E-07 |
| **Gold** | mg/mg nicotine | ~ 5.16E-06 | ~ 5.14E-06 | ~ 5.09E-06 | ~ 4.81E-06 | ~ 4.72E-06 | ~ 5.16E-06 |
| **Iron** | mg/mg nicotine | ~ 3.04E-06 | ~ 3.02E-06 | ~ 3.00E-06 | ~ 2.83E-06 | ~ 2.78E-06 | ~ 3.04E-06 |
| **Lead** | mg/mg nicotine | ~ 7.59E-08 | ~ 7.56E-08 | ~ 7.49E-08 | ~ 7.08E-08 | ~ 6.95E-08 | ~ 7.59E-08 |
| **Nickel** | mg/mg nicotine | ~ 1.52E-07 | ~ 1.51E-07 | ~ 1.50E-07 | ~ 1.42E-07 | ~ 1.39E-07 | ~ 1.52E-07 |
| **Selenium** | mg/mg nicotine | ~ 7.26E-07 | ~ 7.23E-07 | ~ 7.16E-07 | ~ 6.77E-07 | ~ 1.08E-07 | ~ 7.26E-07 |
| **Silver** | mg/mg nicotine | ~ 3.04E-08 | ~ 3.02E-08 | ~ 3.00E-08 | ~ 2.83E-08 | ~ 2.78E-08 | ~ 3.04E-08 |
| **Tin** | mg/mg nicotine | ~ 3.04E-07 | ~ 3.02E-07 | ~ 1.80E-06 | ~ 1.70E-06 | ~ 2.78E-07 | ~ 1.80E-06 |
| **Zinc** | mg/mg nicotine | ~ 4.56E-06 | ~ 4.54E-06 | ~ 4.49E-06 | ~ 4.25E-06 | ~ 4.17E-06 | ~ 4.56E-06 |
| **Organic Acids** | | | | | | | |
| **Benzoic Acid** | mg/mg nicotine | 5.66E-01 | 5.67E-01 | 6.29E-01 | 7.08E-01 | 5.02E-01 | 7.08E-01 |
| **Propionic Acid** | mg/mg nicotine | ~ 1.07E-03 | ~ 1.87E-03 | ~ 1.06E-03 | ~ 9.99E-04 | ~ 1.96E-03 | ~ 1.96E-03 |
| **VOCs** | | | | | | | |
| **1,3-butadiene** | mg/mg nicotine | ~ 1.67E-06 | ~ 2.12E-06 | NT | NT | NT | ~ 2.12E-06 |
| **Acrylonitrile** | mg/mg nicotine | ~ 1.09E-06 | ~ 7.44E-07 | NT | NT | NT | ~ 1.09E-06 |
| **Benzene** | mg/mg nicotine | ~ 1.12E-05 | ~ 1.11E-05 | NT | NT | NT | ~ 1.12E-05 |
| **Isoprene** | mg/mg nicotine | ~ 2.57E-06 | ~ 1.71E-06 | NT | NT | NT | ~ 2.57E-06 |
| **Propylene Oxide** | mg/mg nicotine | ~ 4.42E-06 | ~ 6.89E-06 | NT | NT | NT | ~ 6.89E-06 |
| **Toluene** | mg/mg nicotine | ~ 1.35E-05 | ~ 1.24E-06 | NT | NT | NT | ~ 1.35E-05 |
| **Esters & Alcohols** | | | | | | | |
| **1-Butanol** | mg/mg nicotine | ~ 5.81E-05 | ~ 6.54E-04 | NT | NT | NT | ~ 6.54E-04 |
| **Benzyl Acetate** | mg/mg nicotine | ~ 7.65E-05 | ~ 7.61E-05 | NT | NT | NT | ~ 7.65E-05 |
| **Ethyl Acetate** | mg/mg nicotine | ~ 6.09E-05 | ~ 6.06E-05 | NT | NT | NT | ~ 6.09E-05 |
| **Ethyl Acetoacetate** | mg/mg nicotine | ~ 1.19E-04 | ~ 1.18E-04 | NT | NT | NT | ~ 1.19E-04 |

| | | | | | | | |
|---|---|---|---|---|---|---|---|
| **Isoamyl Acetate** | mg/mg nicotine | ~ 6.15E-05 | ~ 6.13E-05 | NT | NT | NT | ~ 6.15E-05 |
| **Isobutyl Acetate** | mg/mg nicotine | ~ 6.12E-05 | ~ 6.10E-05 | NT | NT | NT | ~ 6.12E-05 |
| **Methyl Acetate** | mg/mg nicotine | ~ 6.34E-05 | ~ 7.00E-04 | NT | NT | NT | ~ 7.00E-04 |

~ = approximated numeric value (see Supplemental Materials Appendix 1A) generated from the corresponding LOD and LOQ (See Supplemental Materials Appendix 1B)

## 1.8 Autumn Tobacco 18 mg/mL Intense Nicotine Normalized Analytical Stability Results

| Parameter/ Constituent | Units | T0 | T3 | T6 | T9 | T12 | Max |
|---|---|---|---|---|---|---|---|
| **# of puffs** | NA | 80 | 76 | 68 | 72 | 68 | 80 |
| **pH** | NA | 6.43 | 6.27 | 6.35 | 6.06 | 6.12 | 6.43 |
| **Primary Constituents** | | | | | | | |
| **Nicotine** | mg/mg nicotine | 1.00E+00 | 1.00E+00 | 1.00E+00 | 1.00E+00 | 1.00E+00 | 1.00E+00 |
| **Glycerol** | mg/mg nicotine | 2.62E+01 | 2.59E+01 | 2.52E+01 | 2.50E+01 | 2.47E+01 | 2.62E+01 |
| **Propylene Glycol** | mg/mg nicotine | 3.41E+01 | 3.38E+01 | 3.17E+01 | 3.20E+01 | 3.32E+01 | 3.41E+01 |
| **Water** | mg/mg nicotine | 3.14E+00 | 4.61E+00 | 4.01E+00 | 4.57E+00 | 5.42E+00 | 5.42E+00 |
| **Menthol** | mg/mg nicotine | ~ 1.55E-03 | ~ 1.62E-03 | ~ 4.90E-03 | ~ 4.75E-04 | ~ 2.30E-03 | ~ 4.90E-03 |
| **Diethylene Glycol** | mg/mg nicotine | ~ 1.42E-03 | ~ 1.49E-03 | ~ 4.21E-04 | ~ 4.36E-04 | ~ 2.10E-03 | ~ 2.10E-03 |
| **Ethylene Glycol** | mg/mg nicotine | ~ 9.89E-04 | ~ 1.04E-03 | ~ 2.93E-04 | ~ 3.04E-04 | ~ 1.47E-03 | ~ 1.47E-03 |
| **Carbonyls & Glycidol** | | | | | | | |
| **Acetaldehyde** | mg/mg nicotine | 1.26E-04 | 1.70E-04 | 1.61E-04 | 1.92E-04 | 1.39E-04 | 1.92E-04 |
| **Acrolein** | mg/mg nicotine | 1.27E-04 | ~ 5.03E-05 | ~ 4.09E-05 | ~ 4.94E-05 | ~ 4.72E-05 | 1.27E-04 |
| **Diacetyl** | mg/mg nicotine | ~ 4.72E-05 | ~ 4.84E-05 | ~ 3.91E-05 | ~ 4.77E-05 | ~ 5.80E-06 | ~ 4.84E-05 |
| **Formaldehyde** | mg/mg nicotine | 6.69E-04 | 6.99E-04 | 5.22E-04 | 6.91E-04 | 6.54E-04 | 6.99E-04 |
| **Glycidol** | mg/mg nicotine | 2.49E-04 | 2.19E-04 | 1.78E-04 | 1.44E-04 | 2.70E-04 | 2.70E-04 |
| **Acetyl Propionyl** | mg/mg nicotine | ~ 4.23E-06 | ~ 4.44E-06 | ~ 3.59E-06 | ~ 4.79E-05 | ~ 5.71E-06 | ~ 4.79E-05 |
| **Butyraldehyde** | mg/mg nicotine | ~ 4.23E-06 | ~ 4.43E-06 | ~ 3.58E-06 | ~ 4.33E-06 | ~ 4.58E-06 | ~ 4.58E-06 |
| **Crotonaldehyde** | mg/mg nicotine | ~ 6.79E-06 | ~ 7.12E-06 | ~ 5.75E-06 | ~ 6.95E-06 | ~ 5.65E-06 | ~ 7.12E-06 |

| | | | | | | | |
|---|---|---|---|---|---|---|---|
| **Furfural** | mg/mg nicotine | ~ 6.95E-06 | ~ 7.28E-06 | ~ 5.89E-06 | ~ 7.11E-06 | ~ 7.84E-06 | ~ 7.84E-06 |
| **Nicotine Degradants & TSNAs** | | | | | | | |
| **β-Nicotyrine** | mg/mg nicotine | ~ 3.73E-05 | ~ 3.90E-05 | 1.17E-04 | 8.57E-05 | 1.07E-04 | 1.17E-04 |
| **Cotinine** | mg/mg nicotine | ~ 3.73E-05 | ~ 3.90E-05 | ~ 3.68E-05 | 2.75E-04 | 1.07E-04 | 2.75E-04 |
| **Myosmine** | mg/mg nicotine | 1.09E-04 | 1.84E-04 | 1.71E-04 | 2.12E-04 | 2.02E-04 | 2.12E-04 |
| **Nicotine N Oxide** | mg/mg nicotine | ~ 3.71E-05 | ~ 3.89E-05 | ~ 3.67E-05 | 2.92E-04 | ~ 4.10E-05 | 2.92E-04 |
| **Nornicotine** | mg/mg nicotine | 2.06E-04 | 3.72E-04 | 4.30E-04 | 7.23E-04 | 4.26E-04 | 7.23E-04 |
| **Anabasine** | mg/mg nicotine | ~ 3.40E-06 | ~ 4.01E-06 | ~ 3.37E-06 | ~ 3.48E-06 | ~ 4.92E-06 | ~ 4.92E-06 |
| **Anatabine** | mg/mg nicotine | ~ 3.39E-06 | ~ 3.58E-06 | ~ 3.35E-06 | ~ 3.47E-06 | ~ 3.91E-06 | ~ 3.91E-06 |
| **NNK** | mg/mg nicotine | ~ 9.70E-08 | ~ 1.02E-07 | NT | NT | NT | ~ 1.02E-07 |
| **NNN** | mg/mg nicotine | ~ 2.61E-08 | ~ 1.11E-07 | NT | NT | NT | ~ 1.11E-07 |
| **Metals** | | | | | | | |
| **Arsenic** | mg/mg nicotine | ~ 3.21E-07 | ~ 3.37E-07 | ~ 1.90E-06 | ~ 3.29E-07 | ~ 3.17E-07 | ~ 1.90E-06 |
| **Beryllium** | mg/mg nicotine | ~ 7.06E-08 | ~ 7.40E-08 | ~ 6.98E-08 | ~ 7.23E-08 | ~ 6.98E-08 | ~ 7.40E-08 |
| **Cadmium** | mg/mg nicotine | ~ 3.21E-08 | ~ 3.37E-08 | ~ 3.17E-08 | ~ 3.29E-08 | ~ 3.17E-08 | ~ 3.37E-08 |
| **Chromium** | mg/mg nicotine | ~ 3.21E-07 | ~ 3.37E-07 | ~ 1.90E-06 | ~ 3.29E-07 | ~ 3.17E-07 | ~ 1.90E-06 |
| **Cobalt** | mg/mg nicotine | ~ 1.60E-08 | ~ 1.68E-08 | ~ 1.59E-08 | ~ 1.64E-08 | ~ 1.59E-08 | ~ 1.68E-08 |
| **Copper** | mg/mg nicotine | ~ 8.02E-07 | ~ 8.41E-07 | ~ 7.93E-07 | ~ 8.21E-07 | ~ 7.94E-07 | ~ 8.41E-07 |
| **Gold** | mg/mg nicotine | ~ 5.46E-06 | ~ 5.72E-06 | ~ 5.39E-06 | ~ 5.59E-06 | ~ 5.40E-06 | ~ 5.72E-06 |
| **Iron** | mg/mg nicotine | ~ 3.21E-06 | ~ 3.37E-06 | ~ 3.17E-06 | ~ 3.29E-06 | ~ 3.17E-06 | ~ 3.37E-06 |
| **Lead** | mg/mg nicotine | ~ 8.02E-08 | ~ 8.41E-08 | ~ 7.93E-08 | ~ 8.21E-08 | ~ 7.94E-08 | ~ 8.41E-08 |
| **Nickel** | mg/mg nicotine | ~ 1.60E-07 | ~ 1.68E-07 | ~ 1.59E-07 | ~ 1.64E-07 | ~ 1.59E-07 | ~ 1.68E-07 |
| **Selenium** | mg/mg nicotine | ~ 7.67E-07 | ~ 8.04E-07 | ~ 7.58E-07 | ~ 7.85E-07 | ~ 7.59E-07 | ~ 8.04E-07 |
| **Silver** | mg/mg nicotine | ~ 3.21E-08 | ~ 3.37E-08 | ~ 3.17E-08 | ~ 3.29E-08 | ~ 3.17E-08 | ~ 3.37E-08 |
| **Tin** | mg/mg nicotine | ~ 3.21E-07 | ~ 2.02E-06 | ~ 1.90E-06 | ~ 1.97E-06 | ~ 3.17E-07 | ~ 2.02E-06 |
| **Zinc** | mg/mg nicotine | ~ 4.81E-06 | ~ 5.05E-06 | ~ 4.76E-06 | ~ 4.93E-06 | ~ 4.76E-06 | ~ 5.05E-06 |
| **Organic Acids** | | | | | | | |
| **Benzoic Acid** | mg/mg nicotine | 7.47E-01 | 7.67E-01 | 5.83E-01 | 8.34E-01 | 6.23E-01 | 8.34E-01 |
| **Propionic Acid** | mg/mg nicotine | ~ 1.13E-03 | ~ 2.08E-03 | ~ 1.96E-03 | ~ 1.16E-03 | ~ 2.24E-03 | ~ 2.24E-03 |

| | | | | | | | |
|---|---|---|---|---|---|---|---|
| **VOCs** | | | | | | | |
| **1,3-butadiene** | mg/mg nicotine | ~ 1.76E-06 | ~ 1.85E-06 | NT | NT | NT | ~ 1.85E-06 |
| **Acrylonitrile** | mg/mg nicotine | ~ 1.15E-06 | ~ 1.21E-06 | NT | NT | NT | ~ 1.21E-06 |
| **Benzene** | mg/mg nicotine | ~ 1.19E-05 | ~ 1.24E-05 | NT | NT | NT | ~ 1.24E-05 |
| **Isoprene** | mg/mg nicotine | ~ 2.71E-06 | ~ 2.85E-06 | NT | NT | NT | ~ 2.85E-06 |
| **Propylene Oxide** | mg/mg nicotine | ~ 4.67E-06 | ~ 4.90E-06 | NT | NT | NT | ~ 4.90E-06 |
| **Toluene** | mg/mg nicotine | ~ 1.44E-05 | ~ 1.50E-05 | NT | NT | NT | ~ 1.50E-05 |
| **Esters & Alcohols** | | | | | | | |
| **1-Butanol** | mg/mg nicotine | ~ 6.86E-04 | ~ 7.26E-04 | NT | NT | NT | ~ 7.26E-04 |
| **Benzyl Acetate** | mg/mg nicotine | ~ 8.08E-05 | ~ 8.47E-05 | NT | NT | NT | ~ 8.47E-05 |
| **Ethyl Acetate** | mg/mg nicotine | ~ 6.43E-05 | ~ 6.75E-05 | NT | NT | NT | ~ 6.75E-05 |
| **Ethyl Acetoacetate** | mg/mg nicotine | ~ 1.25E-04 | ~ 1.31E-04 | NT | NT | NT | ~ 1.31E-04 |
| **Isoamyl Acetate** | mg/mg nicotine | ~ 6.50E-05 | ~ 6.82E-05 | NT | NT | NT | ~ 6.82E-05 |
| **Isobutyl Acetate** | mg/mg nicotine | ~ 6.47E-05 | ~ 6.78E-05 | NT | NT | NT | ~ 6.78E-05 |
| **Methyl Acetate** | mg/mg nicotine | ~ 6.70E-05 | ~ 7.03E-05 | NT | NT | NT | ~ 7.03E-05 |

~ = approximated numeric value (see Supplemental Materials Appendix 1A) generated from the corresponding LOD and LOQ (See Supplemental Materials Appendix 1B)

## 1.9 Ruby Menthol 18 mg/mL Non-Intense Nicotine Normalized Analytical Stability Results

| Parameter/ Constituent | Units | T0 | T3 | T6 | T9 | T12 | Max |
|---|---|---|---|---|---|---|---|
| **# of puffs** | NA | 130 | 127 | 135 | 124 | 124 | 135 |
| **pH** | NA | 5.90 | 5.60 | 5.90 | 5.70 | 5.62 | 5.90 |
| **Primary Constituents** | | | | | | | |
| **Nicotine** | mg/mg nicotine | 1.00E+00 | 1.00E+00 | 1.00E+00 | 1.00E+00 | 1.00E+00 | 1.00E+00 |
| **Glycerol** | mg/mg nicotine | 2.59E+01 | 2.60E+01 | 2.43E+01 | 2.43E+01 | 2.46E+01 | 2.60E+01 |
| **Propylene Glycol** | mg/mg nicotine | 3.50E+01 | 3.48E+01 | 3.27E+01 | 3.25E+01 | 3.41E+01 | 3.50E+01 |
| **Water** | mg/mg nicotine | 2.22E+00 | 4.17E+00 | 3.82E+00 | 3.81E+00 | 8.20E+00 | 8.20E+00 |

| | | | | | | | |
|---|---|---|---|---|---|---|---|
| **Menthol** | mg/mg nicotine | 5.73E-01 | 5.51E-01 | 5.33E-01 | 5.37E-01 | 5.23E-01 | 5.73E-01 |
| **Diethylene Glycol** | mg/mg nicotine | ~ 1.34E-03 | ~ 1.39E-03 | ~ 3.69E-04 | ~ 3.69E-04 | ~ 2.02E-03 | ~ 2.02E-03 |
| **Ethylene Glycol** | mg/mg nicotine | ~ 9.30E-04 | ~ 9.70E-04 | ~ 2.57E-04 | ~ 2.57E-04 | ~ 1.41E-03 | ~ 1.41E-03 |
| **Carbonyls & Glycidol** | | | | | | | |
| **Acetaldehyde** | mg/mg nicotine | 2.11E-04 | 2.22E-04 | 2.93E-04 | 2.84E-04 | 2.85E-04 | 2.93E-04 |
| **Acrolein** | mg/mg nicotine | ~ 3.87E-05 | ~ 4.04E-05 | ~ 3.59E-05 | ~ 3.59E-05 | ~ 3.89E-05 | ~ 4.04E-05 |
| **Diacetyl** | mg/mg nicotine | ~ 3.81E-05 | ~ 3.88E-05 | ~ 3.43E-05 | ~ 3.47E-05 | ~ 3.93E-05 | ~ 3.93E-05 |
| **Formaldehyde** | mg/mg nicotine | 5.88E-04 | 5.34E-04 | 6.40E-04 | 6.92E-04 | 8.18E-04 | 8.18E-04 |
| **Glycidol** | mg/mg nicotine | 1.24E-04 | 1.61E-04 | 1.41E-04 | 1.33E-04 | 1.84E-04 | 1.84E-04 |
| **Acetyl Propionyl** | mg/mg nicotine | ~ 3.42E-06 | ~ 3.56E-06 | ~ 3.15E-06 | ~ 3.15E-06 | ~ 4.71E-06 | ~ 4.71E-06 |
| **Butyraldehyde** | mg/mg nicotine | ~ 3.41E-06 | ~ 3.56E-06 | ~ 3.15E-06 | ~ 3.15E-06 | ~ 3.78E-06 | ~ 3.78E-06 |
| **Crotonaldehyde** | mg/mg nicotine | ~ 5.48E-06 | ~ 5.71E-06 | ~ 5.05E-06 | ~ 5.05E-06 | ~ 4.65E-06 | ~ 5.71E-06 |
| **Furfural** | mg/mg nicotine | ~ 5.60E-06 | ~ 5.84E-06 | ~ 5.17E-06 | ~ 5.17E-06 | ~ 6.46E-06 | ~ 6.46E-06 |
| **Nicotine Degradants & TSNAs** | | | | | | | |
| **β-Nicotyrine** | mg/mg nicotine | 9.08E-05 | 9.39E-05 | 2.07E-04 | 1.67E-04 | 1.62E-04 | 2.07E-04 |
| **Cotinine** | mg/mg nicotine | 6.27E-05 | ~ 3.13E-05 | 1.23E-04 | 2.42E-04 | 1.56E-04 | 2.42E-04 |
| **Myosmine** | mg/mg nicotine | 1.22E-04 | 1.96E-04 | 2.60E-04 | 2.90E-04 | 3.01E-04 | 3.01E-04 |
| **Nicotine N Oxide** | mg/mg nicotine | 5.90E-05 | 6.75E-05 | 5.08E-05 | 2.56E-04 | ~ 3.38E-05 | 2.56E-04 |
| **Nornicotine** | mg/mg nicotine | 1.81E-04 | 3.78E-04 | 4.46E-04 | 6.68E-04 | 6.40E-04 | 6.68E-04 |
| **Anabasine** | mg/mg nicotine | ~ 2.75E-06 | ~ 3.22E-06 | ~ 2.53E-06 | ~ 2.53E-06 | ~ 4.05E-06 | ~ 4.05E-06 |
| **Anatabine** | mg/mg nicotine | ~ 2.73E-06 | ~ 2.87E-06 | ~ 2.52E-06 | ~ 2.52E-06 | ~ 3.22E-06 | ~ 3.22E-06 |
| **NNK** | mg/mg nicotine | ~ 9.13E-08 | ~ 9.52E-08 | NT | NT | NT | ~ 9.52E-08 |
| **NNN** | mg/mg nicotine | 3.53E-07 | ~ 1.04E-07 | NT | NT | NT | 3.53E-07 |
| **Metals** | | | | | | | |
| **Arsenic** | mg/mg nicotine | ~ 3.02E-07 | ~ 3.15E-07 | ~ 2.79E-07 | ~ 2.79E-07 | ~ 3.05E-07 | ~ 3.15E-07 |
| **Beryllium** | mg/mg nicotine | ~ 6.65E-08 | ~ 6.93E-08 | ~ 6.13E-08 | ~ 6.13E-08 | ~ 6.71E-08 | ~ 6.93E-08 |
| **Cadmium** | mg/mg nicotine | ~ 3.02E-08 | ~ 3.15E-08 | ~ 2.79E-08 | ~ 2.79E-08 | ~ 3.05E-08 | ~ 3.15E-08 |
| **Chromium** | mg/mg nicotine | ~ 3.02E-07 | ~ 3.15E-07 | ~ 2.79E-07 | ~ 2.79E-07 | ~ 3.05E-07 | ~ 3.15E-07 |
| **Cobalt** | mg/mg nicotine | ~ 1.51E-08 | ~ 1.57E-08 | ~ 1.39E-08 | ~ 1.39E-08 | ~ 1.53E-08 | ~ 1.57E-08 |

| | | | | | | | |
|---|---|---|---|---|---|---|---|
| **Copper** | mg/mg nicotine | ~ 7.55E-07 | ~ 7.87E-07 | ~ 6.96E-07 | ~ 6.97E-07 | ~ 7.63E-07 | ~ 7.87E-07 |
| **Gold** | mg/mg nicotine | ~ 5.14E-06 | ~ 5.35E-06 | ~ 4.74E-06 | ~ 4.74E-06 | ~ 5.19E-06 | ~ 5.35E-06 |
| **Iron** | mg/mg nicotine | ~ 3.02E-06 | ~ 3.15E-06 | ~ 2.79E-06 | ~ 2.79E-06 | ~ 3.05E-06 | ~ 3.15E-06 |
| **Lead** | mg/mg nicotine | ~ 7.55E-08 | ~ 7.87E-08 | ~ 6.96E-08 | ~ 6.97E-08 | ~ 7.63E-08 | ~ 7.87E-08 |
| **Nickel** | mg/mg nicotine | ~ 1.51E-07 | ~ 1.57E-07 | ~ 1.39E-07 | ~ 1.39E-07 | ~ 1.53E-07 | ~ 1.57E-07 |
| **Selenium** | mg/mg nicotine | ~ 7.22E-07 | ~ 7.52E-07 | ~ 6.66E-07 | ~ 6.66E-07 | ~ 7.29E-07 | ~ 7.52E-07 |
| **Silver** | mg/mg nicotine | ~ 3.02E-08 | ~ 3.15E-08 | ~ 1.67E-07 | ~ 2.79E-08 | ~ 3.05E-08 | ~ 1.67E-07 |
| **Tin** | mg/mg nicotine | ~ 3.02E-07 | ~ 3.15E-07 | ~ 2.79E-07 | ~ 2.79E-07 | ~ 3.05E-07 | ~ 3.15E-07 |
| **Zinc** | mg/mg nicotine | ~ 4.53E-06 | ~ 4.72E-06 | ~ 4.18E-06 | ~ 4.18E-06 | ~ 4.58E-06 | ~ 4.72E-06 |
| **Organic Acids** | | | | | | | |
| **Benzoic Acid** | mg/mg nicotine | 5.03E-01 | 7.19E-01 | 6.42E-01 | 6.68E-01 | 5.72E-01 | 7.19E-01 |
| **Propionic Acid** | mg/mg nicotine | ~ 1.39E-03 | ~ 1.95E-03 | ~ 9.83E-04 | ~ 9.83E-04 | ~ 2.15E-03 | ~ 2.15E-03 |
| **VOCs** | | | | | | | |
| **1,3-butadiene** | mg/mg nicotine | ~ 1.66E-06 | ~ 2.20E-06 | NT | NT | NT | ~ 2.20E-06 |
| **Acrylonitrile** | mg/mg nicotine | ~ 1.09E-06 | ~ 7.75E-07 | NT | NT | NT | ~ 1.09E-06 |
| **Benzene** | mg/mg nicotine | ~ 1.11E-05 | ~ 1.15E-05 | NT | NT | NT | ~ 1.15E-05 |
| **Isoprene** | mg/mg nicotine | ~ 2.55E-06 | ~ 1.78E-06 | NT | NT | NT | ~ 2.55E-06 |
| **Propylene Oxide** | mg/mg nicotine | ~ 4.40E-06 | ~ 7.17E-06 | NT | NT | NT | ~ 7.17E-06 |
| **Toluene** | mg/mg nicotine | ~ 1.35E-05 | ~ 1.29E-06 | NT | NT | NT | ~ 1.35E-05 |
| **Esters & Alcohols** | | | | | | | |
| **1-Butanol** | mg/mg nicotine | ~ 5.78E-05 | ~ 6.02E-05 | NT | NT | NT | ~ 6.02E-05 |
| **Benzyl Acetate** | mg/mg nicotine | ~ 7.60E-05 | ~ 7.92E-05 | NT | NT | NT | ~ 7.92E-05 |
| **Ethyl Acetate** | mg/mg nicotine | ~ 6.06E-05 | ~ 6.31E-05 | NT | NT | NT | ~ 6.31E-05 |
| **Ethyl Acetoacetate** | mg/mg nicotine | ~ 1.18E-04 | ~ 1.23E-04 | NT | NT | NT | ~ 1.23E-04 |
| **Isoamyl Acetate** | mg/mg nicotine | ~ 6.12E-05 | ~ 6.38E-05 | NT | NT | NT | ~ 6.38E-05 |
| **Isobutyl Acetate** | mg/mg nicotine | ~ 6.09E-05 | ~ 6.35E-05 | NT | NT | NT | ~ 6.35E-05 |
| **Methyl Acetate** | mg/mg nicotine | ~ 6.31E-05 | ~ 7.29E-04 | NT | NT | NT | ~ 7.29E-04 |

~ = approximated numeric value (see Supplemental Materials Appendix 1A) generated from the corresponding LOD and LOQ (See Supplemental Materials Appendix 1B)

## 1.10 Ruby Menthol 18 mg/mL Intense Nicotine Normalized Analytical Stability Results

| Parameter/ Constituent | Units | T0 | T3 | T6 | T9 | T12 | Max |
|---|---|---|---|---|---|---|---|
| **# of puffs** | NA | 90 | 76 | 79 | 85 | 73 | 90 |
| **pH** | NA | 6.09 | 5.96 | 5.89 | 5.61 | 5.68 | 6.09 |
| **Primary Constituents** | | | | | | | |
| **Nicotine** | mg/mg nicotine | 1.00E+00 | 1.00E+00 | 1.00E+00 | 1.00E+00 | 1.00E+00 | 1.00E+00 |
| **Glycerol** | mg/mg nicotine | 2.62E+01 | 2.59E+01 | 2.48E+01 | 2.38E+01 | 2.48E+01 | 2.62E+01 |
| **Propylene Glycol** | mg/mg nicotine | 3.50E+01 | 3.48E+01 | 3.22E+01 | 3.22E+01 | 3.44E+01 | 3.50E+01 |
| **Water** | mg/mg nicotine | 2.97E+00 | 3.86E+00 | 4.02E+00 | 3.87E+00 | 6.18E+00 | 6.18E+00 |
| **Menthol** | mg/mg nicotine | 5.58E-01 | 5.47E-01 | 5.30E-01 | 5.17E-01 | 5.20E-01 | 5.58E-01 |
| **Diethylene Glycol** | mg/mg nicotine | ~ 1.51E-03 | ~ 1.47E-03 | ~ 3.95E-04 | ~ 3.54E-04 | ~ 2.09E-03 | ~ 2.09E-03 |
| **Ethylene Glycol** | mg/mg nicotine | ~ 1.05E-03 | ~ 1.03E-03 | ~ 2.75E-04 | ~ 2.47E-04 | ~ 1.46E-03 | ~ 1.46E-03 |
| **Carbonyls & Glycidol** | | | | | | | |
| **Acetaldehyde** | mg/mg nicotine | 2.61E-04 | 2.97E-04 | 3.23E-04 | 3.62E-04 | 2.97E-04 | 3.62E-04 |
| **Acrolein** | mg/mg nicotine | 1.25E-04 | ~ 4.99E-05 | 6.82E-05 | ~ 4.02E-05 | ~ 4.68E-05 | 1.25E-04 |
| **Diacetyl** | mg/mg nicotine | ~ 5.03E-05 | ~ 4.79E-05 | ~ 3.67E-05 | ~ 3.88E-05 | ~ 4.73E-05 | ~ 5.03E-05 |
| **Formaldehyde** | mg/mg nicotine | 7.08E-04 | 7.44E-04 | 6.94E-04 | 8.32E-04 | 9.31E-04 | 9.31E-04 |
| **Glycidol** | mg/mg nicotine | 3.21E-04 | 2.00E-04 | 2.22E-04 | 1.68E-04 | 3.04E-04 | 3.21E-04 |
| **Acetyl Propionyl** | mg/mg nicotine | ~ 4.51E-06 | ~ 4.40E-06 | ~ 3.37E-06 | ~ 3.53E-06 | ~ 5.67E-06 | ~ 5.67E-06 |
| **Butyraldehyde** | mg/mg nicotine | ~ 4.51E-06 | ~ 4.39E-06 | ~ 3.37E-06 | ~ 3.52E-06 | ~ 4.55E-06 | ~ 4.55E-06 |
| **Crotonaldehyde** | mg/mg nicotine | ~ 7.24E-06 | ~ 7.05E-06 | ~ 5.40E-06 | ~ 5.65E-06 | ~ 5.60E-06 | ~ 7.24E-06 |
| **Furfural** | mg/mg nicotine | ~ 7.41E-06 | ~ 7.21E-06 | ~ 5.53E-06 | ~ 5.78E-06 | ~ 7.78E-06 | ~ 7.78E-06 |
| **Nicotine Degradants & TSNAs** | | | | | | | |
| **β-Nicotyrine** | mg/mg nicotine | ~ 3.97E-05 | 1.30E-04 | 2.44E-04 | 2.94E-04 | 2.86E-04 | 2.94E-04 |
| **Cotinine** | mg/mg nicotine | 7.36E-05 | ~ 3.86E-05 | 1.24E-04 | 2.65E-04 | 1.68E-04 | 2.65E-04 |
| **Myosmine** | mg/mg nicotine | 1.21E-04 | 2.31E-04 | 2.71E-04 | 3.21E-04 | 3.03E-04 | 3.21E-04 |

| | | | | | | | |
|---|---|---|---|---|---|---|---|
| **Nicotine N Oxide** | mg/mg nicotine | ~ 3.96E-05 | ~ 3.86E-05 | ~ 3.45E-05 | 2.35E-04 | ~ 4.07E-05 | 2.35E-04 |
| **Nornicotine** | mg/mg nicotine | 1.93E-04 | 3.40E-04 | 4.85E-04 | 8.86E-04 | 7.34E-04 | 8.86E-04 |
| **Anabasine** | mg/mg nicotine | ~ 3.63E-06 | ~ 3.97E-06 | ~ 3.16E-06 | ~ 2.84E-06 | ~ 4.88E-06 | ~ 4.88E-06 |
| **Anatabine** | mg/mg nicotine | ~ 3.61E-06 | ~ 3.55E-06 | ~ 3.15E-06 | ~ 2.82E-06 | ~ 3.88E-06 | ~ 3.88E-06 |
| **NNK** | mg/mg nicotine | ~ 1.03E-07 | ~ 1.01E-07 | NT | NT | NT | ~ 1.03E-07 |
| **NNN** | mg/mg nicotine | 4.46E-07 | 2.60E-07 | NT | NT | NT | 4.46E-07 |
| **Metals** | | | | | | | |
| **Arsenic** | mg/mg nicotine | ~ 3.42E-07 | ~ 3.33E-07 | ~ 2.98E-07 | ~ 2.67E-07 | ~ 3.15E-07 | ~ 3.42E-07 |
| **Beryllium** | mg/mg nicotine | ~ 7.53E-08 | ~ 7.33E-08 | ~ 6.56E-08 | ~ 5.88E-08 | ~ 6.93E-08 | ~ 7.53E-08 |
| **Cadmium** | mg/mg nicotine | ~ 3.42E-08 | ~ 3.33E-08 | ~ 2.98E-08 | ~ 2.67E-08 | ~ 3.15E-08 | ~ 3.42E-08 |
| **Chromium** | mg/mg nicotine | ~ 3.42E-07 | ~ 3.33E-07 | ~ 2.98E-07 | ~ 2.67E-07 | ~ 3.15E-07 | ~ 3.42E-07 |
| **Cobalt** | mg/mg nicotine | ~ 1.71E-08 | ~ 1.67E-08 | ~ 1.49E-08 | ~ 1.34E-08 | ~ 1.58E-08 | ~ 1.71E-08 |
| **Copper** | mg/mg nicotine | ~ 8.56E-07 | ~ 8.33E-07 | ~ 7.45E-07 | ~ 6.68E-07 | ~ 7.88E-07 | ~ 8.56E-07 |
| **Gold** | mg/mg nicotine | ~ 5.82E-06 | ~ 5.67E-06 | ~ 5.07E-06 | ~ 4.54E-06 | ~ 5.36E-06 | ~ 5.82E-06 |
| **Iron** | mg/mg nicotine | ~ 3.42E-06 | ~ 3.33E-06 | ~ 2.98E-06 | ~ 2.67E-06 | ~ 3.15E-06 | ~ 3.42E-06 |
| **Lead** | mg/mg nicotine | ~ 8.56E-08 | ~ 4.17E-07 | ~ 7.45E-08 | ~ 6.68E-08 | ~ 7.88E-08 | ~ 4.17E-07 |
| **Nickel** | mg/mg nicotine | ~ 1.71E-07 | ~ 1.67E-07 | ~ 1.49E-07 | ~ 1.34E-07 | ~ 1.58E-07 | ~ 1.71E-07 |
| **Selenium** | mg/mg nicotine | ~ 8.18E-07 | ~ 7.97E-07 | ~ 7.12E-07 | ~ 6.39E-07 | ~ 7.53E-07 | ~ 8.18E-07 |
| **Silver** | mg/mg nicotine | ~ 3.42E-08 | ~ 3.33E-08 | ~ 1.79E-07 | ~ 2.67E-08 | ~ 3.15E-08 | ~ 1.79E-07 |
| **Tin** | mg/mg nicotine | ~ 3.42E-07 | ~ 2.00E-06 | ~ 2.98E-07 | ~ 2.67E-07 | ~ 3.15E-07 | ~ 2.00E-06 |
| **Zinc** | mg/mg nicotine | ~ 5.13E-06 | ~ 5.00E-06 | ~ 4.47E-06 | ~ 4.01E-06 | ~ 4.73E-06 | ~ 5.13E-06 |
| **Organic Acids** | | | | | | | |
| **Benzoic Acid** | mg/mg nicotine | 8.02E-01 | 7.94E-01 | 6.24E-01 | 7.45E-01 | 5.56E-01 | 8.02E-01 |
| **Propionic Acid** | mg/mg nicotine | ~ 2.12E-03 | ~ 2.06E-03 | ~ 1.84E-03 | ~ 1.35E-03 | ~ 2.22E-03 | ~ 2.22E-03 |
| **VOCs** | | | | | | | |
| **1,3-butadiene** | mg/mg nicotine | ~ 1.88E-06 | ~ 1.83E-06 | NT | NT | NT | ~ 1.88E-06 |
| **Acrylonitrile** | mg/mg nicotine | ~ 1.23E-06 | ~ 1.20E-06 | NT | NT | NT | ~ 1.23E-06 |
| **Benzene** | mg/mg nicotine | ~ 1.27E-05 | ~ 1.23E-05 | NT | NT | NT | ~ 1.27E-05 |

| | | | | | | | |
|---|---|---|---|---|---|---|---|
| **Isoprene** | mg/mg nicotine | ~ 2.89E-06 | ~ 2.82E-06 | NT | NT | NT | ~ 2.89E-06 |
| **Propylene Oxide** | mg/mg nicotine | ~ 4.98E-06 | ~ 4.85E-06 | NT | NT | NT | ~ 4.98E-06 |
| **Toluene** | mg/mg nicotine | ~ 1.53E-05 | ~ 1.48E-05 | NT | NT | NT | ~ 1.53E-05 |
| **Esters & Alcohols** | | | | | | | |
| **1-Butanol** | mg/mg nicotine | ~ 6.55E-05 | ~ 6.38E-05 | NT | NT | NT | ~ 6.55E-05 |
| **Benzyl Acetate** | mg/mg nicotine | ~ 8.62E-05 | ~ 8.39E-05 | NT | NT | NT | ~ 8.62E-05 |
| **Ethyl Acetate** | mg/mg nicotine | ~ 6.86E-05 | ~ 6.68E-05 | NT | NT | NT | ~ 6.86E-05 |
| **Ethyl Acetoacetate** | mg/mg nicotine | ~ 1.34E-04 | ~ 1.30E-04 | NT | NT | NT | ~ 1.34E-04 |
| **Isoamyl Acetate** | mg/mg nicotine | ~ 6.93E-05 | ~ 6.75E-05 | NT | NT | NT | ~ 6.93E-05 |
| **Isobutyl Acetate** | mg/mg nicotine | ~ 6.90E-05 | ~ 6.72E-05 | NT | NT | NT | ~ 6.90E-05 |
| **Methyl Acetate** | mg/mg nicotine | ~ 7.15E-05 | ~ 8.02E-04 | NT | NT | NT | ~ 8.02E-04 |

~ = approximated numeric value (see Supplemental Materials Appendix 1A) generated from the corresponding LOD and LOQ (See Supplemental Materials Appendix 1B)

## 1.11 Summer Menthol 18 mg/mL Non-Intense Nicotine Normalized Analytical Stability Results

| Parameter/ Constituent | Units | T0 | T3 | T6 | T9 | T12 | Max |
|---|---|---|---|---|---|---|---|
| **# of puffs** | NA | 126 | 127 | 138 | 127 | 117 | 138 |
| **pH** | NA | 6.42 | 6.31 | 6.46 | 6.29 | 6.28 | 6.46 |
| **Primary Constituents** | | | | | | | |
| **Nicotine** | mg/mg nicotine | 1.00E+00 | 1.00E+00 | 1.00E+00 | 1.00E+00 | 1.00E+00 | 1.00E+00 |
| **Glycerol** | mg/mg nicotine | 2.57E+01 | 2.57E+01 | 2.37E+01 | 2.33E+01 | 2.42E+01 | 2.57E+01 |
| **Propylene Glycol** | mg/mg nicotine | 3.34E+01 | 3.32E+01 | 3.13E+01 | 3.08E+01 | 3.25E+01 | 3.34E+01 |
| **Water** | mg/mg nicotine | 2.50E+00 | 4.31E+00 | 4.32E+00 | 4.19E+00 | 8.38E+00 | 8.38E+00 |
| **Menthol** | mg/mg nicotine | 5.65E-01 | 5.43E-01 | 5.24E-01 | 5.26E-01 | 5.23E-01 | 5.65E-01 |
| **Diethylene Glycol** | mg/mg nicotine | ~ 1.32E-03 | ~ 1.37E-03 | ~ 3.56E-04 | ~ 3.50E-04 | ~ 2.09E-03 | ~ 2.09E-03 |
| **Ethylene Glycol** | mg/mg nicotine | ~ 9.18E-04 | ~ 9.54E-04 | ~ 2.48E-04 | ~ 2.44E-04 | ~ 1.46E-03 | ~ 1.46E-03 |
| **Carbonyls & Glycidol** | | | | | | | |

| | | | | | | | |
|---|---|---|---|---|---|---|---|
| **Acetaldehyde** | mg/mg nicotine | 7.62E-05 | 9.50E-05 | 9.51E-05 | 9.13E-05 | 1.12E-04 | 1.12E-04 |
| **Acrolein** | mg/mg nicotine | ~ 3.82E-05 | ~ 3.97E-05 | ~ 3.46E-05 | ~ 3.40E-05 | ~ 4.02E-05 | ~ 4.02E-05 |
| **Diacetyl** | mg/mg nicotine | ~ 3.42E-06 | ~ 3.82E-05 | ~ 3.07E-06 | ~ 3.28E-05 | ~ 4.06E-05 | ~ 4.06E-05 |
| **Formaldehyde** | mg/mg nicotine | 4.90E-04 | 4.00E-04 | 4.26E-04 | 4.08E-04 | 5.38E-04 | 5.38E-04 |
| **Glycidol** | mg/mg nicotine | 1.09E-04 | 1.17E-04 | 1.00E-04 | 9.36E-05 | 1.12E-04 | 1.17E-04 |
| **Acetyl Propionyl** | mg/mg nicotine | ~ 3.37E-06 | ~ 3.50E-06 | ~ 3.03E-06 | ~ 3.30E-05 | ~ 4.87E-06 | ~ 3.30E-05 |
| **Butyraldehyde** | mg/mg nicotine | ~ 3.37E-06 | ~ 3.50E-06 | ~ 3.03E-06 | ~ 2.98E-06 | ~ 3.91E-06 | ~ 3.91E-06 |
| **Crotonaldehyde** | mg/mg nicotine | ~ 5.40E-06 | ~ 5.62E-06 | ~ 4.86E-06 | ~ 4.78E-06 | ~ 4.81E-06 | ~ 5.62E-06 |
| **Furfural** | mg/mg nicotine | ~ 5.53E-06 | ~ 5.75E-06 | ~ 4.98E-06 | ~ 4.89E-06 | ~ 6.68E-06 | ~ 6.68E-06 |
| **Nicotine Degradants & TSNAs** | | | | | | | |
| **β-Nicotyrine** | mg/mg nicotine | ~ 2.97E-05 | ~ 3.08E-05 | 7.87E-05 | 5.43E-05 | ~ 3.09E-05 | 7.87E-05 |
| **Cotinine** | mg/mg nicotine | ~ 2.97E-05 | ~ 2.74E-06 | ~ 2.67E-05 | 1.18E-04 | ~ 3.44E-05 | 1.18E-04 |
| **Myosmine** | mg/mg nicotine | ~ 2.96E-05 | 8.95E-05 | 8.50E-05 | 9.55E-05 | 1.13E-04 | 1.13E-04 |
| **Nicotine N Oxide** | mg/mg nicotine | 9.65E-05 | ~ 3.07E-05 | 5.04E-05 | 1.86E-04 | ~ 3.50E-05 | 1.86E-04 |
| **Nornicotine** | mg/mg nicotine | 1.40E-04 | 2.56E-04 | 3.08E-04 | 4.54E-04 | 4.14E-04 | 4.54E-04 |
| **Anabasine** | mg/mg nicotine | ~ 2.71E-06 | ~ 3.16E-06 | ~ 2.44E-06 | ~ 2.40E-06 | ~ 4.19E-06 | ~ 4.19E-06 |
| **Anatabine** | mg/mg nicotine | ~ 2.70E-06 | ~ 2.83E-06 | ~ 2.43E-06 | ~ 2.39E-06 | ~ 3.33E-06 | ~ 3.33E-06 |
| **NNK** | mg/mg nicotine | ~ 9.01E-08 | ~ 9.37E-08 | NT | NT | NT | ~ 9.37E-08 |
| **NNN** | mg/mg nicotine | ~ 2.42E-08 | ~ 2.52E-08 | NT | NT | NT | ~ 2.52E-08 |
| **Metals** | | | | | | | |
| **Arsenic** | mg/mg nicotine | ~ 2.98E-07 | ~ 3.10E-07 | ~ 2.68E-07 | ~ 2.64E-07 | ~ 3.16E-07 | ~ 3.16E-07 |
| **Beryllium** | mg/mg nicotine | ~ 6.56E-08 | ~ 6.82E-08 | ~ 5.90E-08 | ~ 5.80E-08 | ~ 6.95E-08 | ~ 6.95E-08 |
| **Cadmium** | mg/mg nicotine | ~ 2.98E-08 | ~ 3.10E-08 | ~ 2.68E-08 | ~ 2.64E-08 | ~ 3.16E-08 | ~ 3.16E-08 |
| **Chromium** | mg/mg nicotine | ~ 2.98E-07 | ~ 3.10E-07 | ~ 2.68E-07 | ~ 2.64E-07 | ~ 3.16E-07 | ~ 3.16E-07 |
| **Cobalt** | mg/mg nicotine | ~ 1.49E-08 | ~ 1.55E-08 | ~ 1.34E-08 | ~ 1.32E-08 | ~ 1.58E-08 | ~ 1.58E-08 |
| **Copper** | mg/mg nicotine | ~ 7.45E-07 | ~ 7.75E-07 | ~ 6.71E-07 | ~ 6.60E-07 | ~ 7.89E-07 | ~ 7.89E-07 |
| **Gold** | mg/mg nicotine | ~ 5.07E-06 | ~ 5.27E-06 | ~ 4.56E-06 | ~ 4.48E-06 | ~ 5.37E-06 | ~ 5.37E-06 |
| **Iron** | mg/mg nicotine | ~ 2.98E-06 | ~ 3.10E-06 | ~ 2.68E-06 | ~ 2.64E-06 | ~ 3.16E-06 | ~ 3.16E-06 |

| | | | | | | | |
|---|---|---|---|---|---|---|---|
| **Lead** | mg/mg nicotine | ~ 7.45E-08 | ~ 7.75E-08 | ~ 6.71E-08 | ~ 6.60E-08 | ~ 7.89E-08 | ~ 7.89E-08 |
| **Nickel** | mg/mg nicotine | ~ 1.49E-07 | ~ 1.55E-07 | ~ 1.34E-07 | ~ 1.32E-07 | ~ 1.58E-07 | ~ 1.58E-07 |
| **Selenium** | mg/mg nicotine | ~ 7.13E-07 | ~ 7.41E-07 | ~ 6.41E-07 | ~ 1.03E-07 | ~ 1.23E-07 | ~ 7.41E-07 |
| **Silver** | mg/mg nicotine | ~ 2.98E-08 | ~ 3.10E-08 | ~ 1.61E-07 | ~ 2.64E-08 | ~ 3.16E-08 | ~ 1.61E-07 |
| **Tin** | mg/mg nicotine | ~ 2.98E-07 | ~ 3.10E-07 | ~ 2.68E-07 | ~ 2.64E-07 | ~ 3.16E-07 | ~ 3.16E-07 |
| **Zinc** | mg/mg nicotine | ~ 1.49E-05 | ~ 4.65E-06 | ~ 4.02E-06 | ~ 3.96E-06 | ~ 4.74E-06 | ~ 1.49E-05 |
| **Organic Acids** | | | | | | | |
| **Benzoic Acid** | mg/mg nicotine | 5.01E-01 | 6.87E-01 | 5.84E-01 | 6.80E-01 | 5.77E-01 | 6.87E-01 |
| **Propionic Acid** | mg/mg nicotine | ~ 1.38E-03 | ~ 1.92E-03 | ~ 9.46E-04 | ~ 1.06E-03 | ~ 2.23E-03 | ~ 2.23E-03 |
| **VOCs** | | | | | | | |
| **1,3-butadiene** | mg/mg nicotine | ~ 1.64E-06 | ~ 2.17E-06 | NT | NT | NT | ~ 2.17E-06 |
| **Acrylonitrile** | mg/mg nicotine | ~ 1.07E-06 | ~ 7.62E-07 | NT | NT | NT | ~ 1.07E-06 |
| **Benzene** | mg/mg nicotine | ~ 1.10E-05 | ~ 1.13E-05 | NT | NT | NT | ~ 1.13E-05 |
| **Isoprene** | mg/mg nicotine | ~ 2.52E-06 | ~ 1.75E-06 | NT | NT | NT | ~ 2.52E-06 |
| **Propylene Oxide** | mg/mg nicotine | ~ 4.34E-06 | ~ 7.06E-06 | NT | NT | NT | ~ 7.06E-06 |
| **Toluene** | mg/mg nicotine | ~ 1.33E-05 | ~ 1.27E-06 | NT | NT | NT | ~ 1.33E-05 |
| **Esters & Alcohols** | | | | | | | |
| **1-Butanol** | mg/mg nicotine | ~ 5.70E-05 | ~ 5.93E-05 | NT | NT | NT | ~ 5.93E-05 |
| **Benzyl Acetate** | mg/mg nicotine | ~ 7.51E-05 | ~ 7.80E-05 | NT | NT | NT | ~ 7.80E-05 |
| **Ethyl Acetate** | mg/mg nicotine | 1.02E-02 | 8.11E-03 | NT | NT | NT | 1.02E-02 |
| **Ethyl Acetoacetate** | mg/mg nicotine | ~ 1.16E-04 | ~ 1.21E-04 | NT | NT | NT | ~ 1.21E-04 |
| **Isoamyl Acetate** | mg/mg nicotine | ~ 6.04E-05 | ~ 6.28E-05 | NT | NT | NT | ~ 6.28E-05 |
| **Isobutyl Acetate** | mg/mg nicotine | ~ 6.01E-05 | ~ 6.25E-05 | NT | NT | NT | ~ 6.25E-05 |
| **Methyl Acetate** | mg/mg nicotine | ~ 6.23E-05 | ~ 7.18E-04 | NT | NT | NT | ~ 7.18E-04 |

~ = approximated numeric value (see Supplemental Materials Appendix 1A) generated from the corresponding LOD and LOQ (See Supplemental Materials Appendix 1B)

## 1.12 Summer Menthol 18 mg/mL Intense Nicotine Normalized Analytical Stability Results

| Parameter/ Constituent | Units | T0 | T3 | T6 | T9 | T12 | Max |
|---|---|---|---|---|---|---|---|
| **# of puffs** | NA | 86 | 80 | 74 | 86 | 69 | 86 |
| **pH** | NA | 6.40 | 6.51 | 6.40 | 6.25 | 6.29 | 6.51 |
| **Primary Constituents** | | | | | | | |
| **Nicotine** | mg/mg nicotine | 1.00E+00 | 1.00E+00 | 1.00E+00 | 1.00E+00 | 1.00E+00 | 1.00E+00 |
| **Glycerol** | mg/mg nicotine | 2.59E+01 | 2.57E+01 | 2.44E+01 | 2.36E+01 | 2.45E+01 | 2.59E+01 |
| **Propylene Glycol** | mg/mg nicotine | 3.36E+01 | 3.33E+01 | 3.09E+01 | 3.08E+01 | 3.27E+01 | 3.36E+01 |
| **Water** | mg/mg nicotine | 3.17E+00 | 4.08E+00 | 4.14E+00 | 4.40E+00 | 6.47E+00 | 6.47E+00 |
| **Menthol** | mg/mg nicotine | 5.60E-01 | 5.46E-01 | 5.28E-01 | 5.14E-01 | 5.13E-01 | 5.60E-01 |
| **Diethylene Glycol** | mg/mg nicotine | ~ 1.32E-03 | ~ 1.31E-03 | ~ 3.80E-04 | ~ 3.57E-04 | ~ 2.19E-03 | ~ 2.19E-03 |
| **Ethylene Glycol** | mg/mg nicotine | ~ 9.20E-04 | ~ 9.14E-04 | ~ 2.65E-04 | ~ 2.49E-04 | ~ 1.52E-03 | ~ 1.52E-03 |
| **Carbonyls & Glycidol** | | | | | | | |
| **Acetaldehyde** | mg/mg nicotine | 1.16E-04 | 1.12E-04 | 8.92E-05 | 1.20E-04 | ~ 5.08E-05 | 1.20E-04 |
| **Acrolein** | mg/mg nicotine | 1.24E-04 | ~ 4.44E-05 | ~ 3.70E-05 | ~ 4.05E-05 | ~ 4.90E-05 | 1.24E-04 |
| **Diacetyl** | mg/mg nicotine | ~ 3.99E-06 | ~ 4.26E-05 | ~ 3.53E-05 | ~ 3.91E-05 | ~ 4.95E-05 | ~ 4.95E-05 |
| **Formaldehyde** | mg/mg nicotine | 6.56E-04 | 5.85E-04 | 4.44E-04 | 5.30E-04 | 5.42E-04 | 6.56E-04 |
| **Glycidol** | mg/mg nicotine | 2.13E-04 | 1.82E-04 | 1.65E-04 | 1.27E-04 | 2.62E-04 | 2.62E-04 |
| **Acetyl Propionyl** | mg/mg nicotine | ~ 3.94E-06 | ~ 3.91E-06 | ~ 3.24E-06 | ~ 3.55E-06 | ~ 5.94E-06 | ~ 5.94E-06 |
| **Butyraldehyde** | mg/mg nicotine | ~ 3.93E-06 | ~ 3.91E-06 | ~ 3.24E-06 | ~ 3.55E-06 | ~ 4.76E-06 | ~ 4.76E-06 |
| **Crotonaldehyde** | mg/mg nicotine | ~ 6.31E-06 | ~ 6.27E-06 | ~ 5.20E-06 | ~ 5.70E-06 | ~ 5.87E-06 | ~ 6.31E-06 |
| **Furfural** | mg/mg nicotine | ~ 6.46E-06 | ~ 6.42E-06 | ~ 5.32E-06 | ~ 5.83E-06 | ~ 8.15E-06 | ~ 8.15E-06 |
| **Nicotine Degradants & TSNAs** | | | | | | | |
| **β-Nicotyrine** | mg/mg nicotine | ~ 3.47E-05 | ~ 3.44E-05 | 7.81E-05 | 6.46E-05 | 7.54E-05 | 7.81E-05 |
| **Cotinine** | mg/mg nicotine | ~ 3.47E-05 | ~ 3.07E-06 | ~ 3.33E-05 | 1.23E-04 | ~ 4.20E-05 | 1.23E-04 |
| **Myosmine** | mg/mg nicotine | ~ 3.46E-05 | 1.00E-04 | 8.38E-05 | 1.08E-04 | 8.78E-05 | 1.08E-04 |
| **Nicotine N Oxide** | mg/mg nicotine | ~ 3.46E-05 | ~ 3.43E-05 | ~ 3.32E-05 | 1.72E-04 | ~ 4.26E-05 | 1.72E-04 |
| **Nornicotine** | mg/mg nicotine | 1.59E-04 | 2.64E-04 | 2.59E-04 | 4.49E-04 | 3.36E-04 | 4.49E-04 |
| **Anabasine** | mg/mg nicotine | ~ 3.17E-06 | ~ 3.54E-06 | ~ 3.04E-06 | ~ 2.86E-06 | ~ 5.11E-06 | ~ 5.11E-06 |

| | | | | | | | |
|---|---|---|---|---|---|---|---|
| **Anatabine** | mg/mg nicotine | ~ 3.15E-06 | ~ 3.16E-06 | ~ 3.03E-06 | ~ 2.84E-06 | ~ 4.06E-06 | ~ 4.06E-06 |
| **NNK** | mg/mg nicotine | ~ 9.03E-08 | ~ 8.97E-08 | NT | NT | NT | ~ 9.03E-08 |
| **NNN** | mg/mg nicotine | ~ 2.43E-08 | ~ 2.41E-08 | NT | NT | NT | ~ 2.43E-08 |
| **Metals** | | | | | | | |
| **Arsenic** | mg/mg nicotine | ~ 2.99E-07 | ~ 2.97E-07 | ~ 2.87E-07 | ~ 2.69E-07 | ~ 3.30E-07 | ~ 3.30E-07 |
| **Beryllium** | mg/mg nicotine | ~ 6.57E-08 | ~ 6.53E-08 | ~ 6.31E-08 | ~ 5.93E-08 | ~ 7.26E-08 | ~ 7.26E-08 |
| **Cadmium** | mg/mg nicotine | ~ 2.99E-08 | ~ 2.97E-08 | ~ 2.87E-08 | ~ 2.69E-08 | ~ 3.30E-08 | ~ 3.30E-08 |
| **Chromium** | mg/mg nicotine | ~ 2.99E-07 | ~ 2.97E-07 | ~ 2.87E-07 | ~ 2.69E-07 | ~ 3.30E-07 | ~ 3.30E-07 |
| **Cobalt** | mg/mg nicotine | ~ 1.49E-08 | ~ 1.48E-08 | ~ 1.43E-08 | ~ 1.35E-08 | ~ 1.65E-08 | ~ 1.65E-08 |
| **Copper** | mg/mg nicotine | ~ 7.46E-07 | ~ 7.42E-07 | ~ 7.17E-07 | ~ 6.74E-07 | ~ 8.25E-07 | ~ 8.25E-07 |
| **Gold** | mg/mg nicotine | ~ 5.08E-06 | ~ 5.04E-06 | ~ 4.87E-06 | ~ 4.58E-06 | ~ 5.61E-06 | ~ 5.61E-06 |
| **Iron** | mg/mg nicotine | ~ 2.99E-06 | ~ 2.97E-06 | ~ 2.87E-06 | ~ 2.69E-06 | ~ 3.30E-06 | ~ 3.30E-06 |
| **Lead** | mg/mg nicotine | ~ 7.46E-08 | ~ 7.42E-08 | ~ 7.17E-08 | ~ 6.74E-08 | ~ 8.25E-08 | ~ 8.25E-08 |
| **Nickel** | mg/mg nicotine | ~ 1.49E-07 | ~ 1.48E-07 | ~ 1.43E-07 | ~ 1.35E-07 | ~ 1.65E-07 | ~ 1.65E-07 |
| **Selenium** | mg/mg nicotine | ~ 7.14E-07 | ~ 7.09E-07 | ~ 6.85E-07 | ~ 6.44E-07 | ~ 7.89E-07 | ~ 7.89E-07 |
| **Silver** | mg/mg nicotine | ~ 2.99E-08 | ~ 2.97E-08 | ~ 1.72E-07 | ~ 2.69E-08 | ~ 3.30E-08 | ~ 1.72E-07 |
| **Tin** | mg/mg nicotine | ~ 2.99E-07 | ~ 2.97E-07 | ~ 2.87E-07 | ~ 2.69E-07 | ~ 3.30E-07 | ~ 3.30E-07 |
| **Zinc** | mg/mg nicotine | ~ 4.48E-06 | ~ 4.45E-06 | ~ 4.30E-06 | ~ 4.04E-06 | ~ 4.95E-06 | ~ 4.95E-06 |
| **Organic Acids** | | | | | | | |
| **Benzoic Acid** | mg/mg nicotine | 7.84E-01 | 6.83E-01 | 5.76E-01 | 7.68E-01 | 5.78E-01 | 7.84E-01 |
| **Propionic Acid** | mg/mg nicotine | ~ 1.85E-03 | ~ 1.84E-03 | ~ 1.77E-03 | ~ 1.49E-03 | ~ 2.33E-03 | ~ 2.33E-03 |
| **VOCs** | | | | | | | |
| **1,3-butadiene** | mg/mg nicotine | ~ 1.64E-06 | ~ 1.63E-06 | NT | NT | NT | ~ 1.64E-06 |
| **Acrylonitrile** | mg/mg nicotine | ~ 1.07E-06 | ~ 1.07E-06 | NT | NT | NT | ~ 1.07E-06 |
| **Benzene** | mg/mg nicotine | ~ 1.11E-05 | ~ 1.10E-05 | NT | NT | NT | ~ 1.11E-05 |
| **Isoprene** | mg/mg nicotine | ~ 2.52E-06 | ~ 2.51E-06 | NT | NT | NT | ~ 2.52E-06 |
| **Propylene Oxide** | mg/mg nicotine | ~ 4.35E-06 | ~ 4.32E-06 | NT | NT | NT | ~ 4.35E-06 |
| **Toluene** | mg/mg nicotine | ~ 1.34E-05 | ~ 1.32E-05 | NT | NT | NT | ~ 1.34E-05 |
| **Esters & Alcohols** | | | | | | | |

| | | | | | | | |
|---|---|---|---|---|---|---|---|
| **1-Butanol** | mg/mg nicotine | ~ 5.71E-05 | ~ 5.68E-05 | NT | NT | NT | ~ 5.71E-05 |
| **Benzyl Acetate** | mg/mg nicotine | ~ 7.52E-05 | ~ 7.47E-05 | NT | NT | NT | ~ 7.52E-05 |
| **Ethyl Acetate** | mg/mg nicotine | 1.07E-02 | 8.57E-03 | NT | NT | NT | 1.07E-02 |
| **Ethyl Acetoacetate** | mg/mg nicotine | ~ 1.17E-04 | ~ 1.16E-04 | NT | NT | NT | ~ 1.17E-04 |
| **Isoamyl Acetate** | mg/mg nicotine | ~ 6.05E-05 | ~ 6.01E-05 | NT | NT | NT | ~ 6.05E-05 |
| **Isobutyl Acetate** | mg/mg nicotine | ~ 6.02E-05 | ~ 5.98E-05 | NT | NT | NT | ~ 6.02E-05 |
| **Methyl Acetate** | mg/mg nicotine | ~ 6.23E-05 | ~ 7.14E-04 | NT | NT | NT | ~ 7.14E-04 |

~ = approximated numeric value (see Supplemental Materials Appendix 1A) generated from the corresponding LOD and LOQ (See Supplemental Materials Appendix 1B)

## 2 Non-Intense (ISO 3308:2012) Combustible Reference Cigarette Constituent Values

| Parameter/ Constituent BLUE= 3R4F BLACK=1R6F | ISO 3308:2012 Mean | Unit | Imputed Formatted | Unit Conversion (mg) | Nicotine (mg/cig) | Nicotine Concentration Source | Nicotine Normalized (mg/mg nicotine) | Analyte Concentration Source |
|---|---|---|---|---|---|---|---|---|
| **Primary Constituents** | | | | | | | | |
| **Nicotine** | 0.721 | mg/cig | 7.21E-01 | 7.21E-01 | 0.72 | CoA 1R6F (University of Kentucky) | 1.00E+00 | CoA 1R6F (University of Kentucky) |
| **Propylene Glycol** | 3.20 | mcg/cig | 3.20E+00 | 3.20E-03 | 0.76 | Uchiyama et al. 2018 | 4.21E-03 | Uchiyama et al. 2018 |
| **Glycerol** | 680.00 | mcg/cig | 6.80E-01 | 6.80E-01 | 0.76 | Uchiyama et al. 2018 | 8.95E-01 | Uchiyama et al. 2018 |
| **Water** | 3.20 | mg/cig | 3.20E+00 | 3.20E+00 | 0.76 | Uchiyama et al. 2018 | 4.21E+00 | Uchiyama et al. 2018 |
| **Menthol** | <0.01 | mcg/cig | <0.01 | 1.00E-02 | 0.76 | Uchiyama et al. 2018 | ~1.32E-05 | Uchiyama et al. 2018 |
| **Diethylene Glycol** | BLOD | mg/cig | BLOD [~0.00] | 1.00E-03 | 0.70 | GRPT-02816 | ~1.42E-03 | Internal Study |
| **Ethylene Glycol** | 0.007 | mg/cig | 7.00E-03 | 7.00E-03 | 0.70 | GRPT-02816 | 9.96E-03 | Internal Study |
| **Carbonyls & Glycidol** | | | | | | | | |
| **Acetaldehyde** | 522 | mcg/cig | 5.22E+02 | 5.22E-01 | 0.72 | CoA 1R6F (University of Kentucky) | 7.24E-01 | CoA 1R6F (University of Kentucky) |
| **Acrolein** | 43 | mcg/cig | 4.30E+01 | 4.30E-02 | 0.72 | CoA 1R6F (University of Kentucky) | 5.96E-02 | CoA 1R6F (University of Kentucky) |
| **Diacetyl** | 114.78 | mcg/cig | 1.15E+02 | 1.15E-01 | 0.70 | Jaccard et al. 2019 | 1.64E-01 | Moldoveanu et al. 2017 |
| **Formaldehyde** | 27 | mcg/cig | 2.70E+01 | 2.70E-02 | 0.72 | CoA 1R6F (University of Kentucky) | 3.74E-02 | CoA 1R6F (University of Kentucky) |
| **Glycidol** | 0.75 | mcg/cig | 7.47E-01 | 7.47E-04 | 0.72 | CoA 1R6F (University of Kentucky) | 1.04E-03 | Internal Study |
| **Acetoin** | BLOQ | mcg/cig | BLOQ [~1.22] | 1.22E-03 | 0.70 | GRPT-02816 | ~1.73E-03 | GRPT-02816 |
| **Butyraldehyde** | 25.90 | mcg/cig | 2.59E+01 | 2.59E-02 | 0.78 | Jaccard et al. 2019 | 3.33E-02 | Jaccard et al. 2019 |

| Parameter/ Constituent BLUE= 3R4F BLACK=1R6F | ISO 3308:2012 Mean | Unit | Imputed Formatted | Unit Conversion (mg) | Nicotine (mg/cig) | Nicotine Concentration Source | Nicotine Normalized (mg/mg nicotine) | Analyte Concentration Source |
|---|---|---|---|---|---|---|---|---|
| **Crotonaldehyde** | 11 | mcg/cig | 1.10E+01 | 1.10E-02 | 0.72 | CoA 1R6F (University of Kentucky) | 1.53E-02 | CoA 1R6F (University of Kentucky) |
| **Furfural** | 14.00 | mcg/cig | 14 | 0.014 | 0.76 | Uchiyama et al. 2018 | 1.84E-02 | Uchiyama et al. 2018 |
| **Acetyl Propionyl** | 29.52 | mcg/cig | 2.95E+01 | 2.95E-02 | 0.70 | Jaccard et al. 2019 | 4.21E-02 | Moldoveanu et al. 2017 |
| **Nicotine Degradants & TSNAs** | | | | | | | | |
| **Beta Nicotyrine** | 7.49 | mcg/cig | 7.49E+00 | 7.49E-03 | 0.70 | GRPT-02816 | 1.07E-02 | Internal Study |
| **Cotinine** | 4.89 | mcg/cig | 4.89E+00 | 4.89E-03 | 0.70 | GRPT-02816 | 6.96E-03 | Internal Study |
| **Myosmine** | NA | NA | NA | NA | NA | NA | NA | NA |
| **Nicotine N Oxide** | BLOQ | mcg/cig | BLOQ [~0.38] | 3.78E-04 | 0.70 | GRPT-02816 | ~5.37E-04 | GRPT-02816 |
| **Nornicotine** | 5.70 | mcg/cig | 5.70E+00 | 5.70E-03 | 0.60 | Jain et al. 2019 | 9.50E-03 | Jain et al. 2019 |
| **Anabasine** | 0.50 | mcg/cig | 5.00E-01 | 5.00E-04 | 0.60 | Jain et al. 2019 | 8.33E-04 | Jain et al. 2019 |
| **Anatabine** | 3.60 | mcg/cig | 3.60E+00 | 3.60E-03 | 0.60 | Jain et al. 2019 | 6.00E-03 | Jain et al. 2019 |
| **NNK** | 71 | ng/cig | 7.10E+01 | 7.10E-05 | 0.72 | CoA 1R6F (University of Kentucky) | 9.85E-05 | CoA 1R6F (University of Kentucky) |
| **NNN** | 85 | ng/cig | 8.50E+01 | 8.50E-05 | 0.72 | CoA 1R6F (University of Kentucky) | 1.18E-04 | CoA 1R6F (University of Kentucky) |
| **Metals** | | | | | | | | |
| **Cadmium** | 26.10 | ng/cig | 2.61E+01 | 2.61E-05 | 0.78 | Jaccard et al. 2019 | 3.35E-05 | Jaccard et al. 2019 |
| **Chromium** | BLOD | ng/cig | BLOD [~0.44] | 4.40E-07 | 0.70 | Jaccard et al. 2019 | ~6.27E-07 | Pappas et al. 2014 |
| **Copper** | 11.5 | ng/cig | 1.15E+01 | 1.15E-05 | 0.70 | GRPT-02816 | 1.64E-05 | GRPT-02816 |

| Parameter/ Constituent BLUE= 3R4F BLACK=1R6F | ISO 3308:2012 Mean | Unit | Imputed Formatted | Unit Conversion (mg) | Nicotine (mg/cig) | Nicotine Concentration Source | Nicotine Normalized (mg/mg nicotine) | Analyte Concentration Source |
|---|---|---|---|---|---|---|---|---|
| **Lead** | BLOQ | ng/cig | BLOQ [~0.33] | 3.25E-07 | 0.78 | Jaccard et al. 2019 | ~4.18E-07 | Jaccard et al. 2019 |
| **Nickel** | BLOD | ng/cig | BLOD [~0.75] | 7.50E-07 | 0.78 | Jaccard et al. 2019 | ~9.64E-07 | Jaccard et al. 2019 |
| **Organic Acids** | | | | | | | | |
| **Benzoic Acid** | NA | NA | NA | NA | NA | NA | NA | NA |
| **Acetic Acid** | NA | NA | NA | NA | NA | NA | NA | NA |
| **Lactic Acid** | NA | NA | NA | NA | NA | NA | NA | NA |
| **Propionic Acid** | NA | NA | NA | NA | NA | NA | NA | NA |
| **VOCs** | | | | | | | | |
| **Acrylonitrile** | 7.0 | mcg/cig | 7.00E+00 | 7.00E-03 | 0.72 | CoA 1R6F (University of Kentucky) | 9.71E-03 | CoA 1R6F (University of Kentucky) |
| **Benzene** | 33 | mcg/cig | 3.30E+01 | 3.30E-02 | 0.72 | CoA 1R6F (University of Kentucky) | 4.58E-02 | CoA 1R6F (University of Kentucky) |
| **Propylene oxide** | 657.00 | mcg/cig | 657 | 0.657 | 0.78 | Jaccard et al. 2019 | 8.44E-01 | Jaccard et al. 2019* |
| **Toluene** | 53 | mcg/cig | 53 | 0.053 | 0.72 | CoA 1R6F (University of Kentucky) | 7.35E-02 | CoA 1R6F (University of Kentucky) |
| **Esters and Alcohols** | | | | | | | | |
| **1-Butanol** | NA | NA | NA | NA | NA | NA | NA | NA |
| **Benzyl acetate** | NA | NA | NA | NA | NA | NA | NA | NA |
| **Ethyl Acetate** | NA | NA | NA | NA | NA | NA | NA | NA |
| **Ethyl acetoacetate** | NA | NA | NA | NA | NA | NA | NA | NA |
| **Isoamyl acetate** | NA | NA | NA | NA | NA | NA | NA | NA |
| **Isobutyl acetate** | NA | NA | NA | NA | NA | NA | NA | NA |
| **Methyl Acetate** | NA | NA | NA | NA | NA | NA | NA | NA |
| **Carbon Monoxide** | | | | | | | | |

| Parameter/ Constituent BLUE= 3R4F BLACK=1R6F | ISO 3308:2012 Mean | Unit | Imputed Formatted | Unit Conversion (mg) | Nicotine (mg/cig) | Nicotine Concentration Source | Nicotine Normalized (mg/mg nicotine) | Analyte Concentration Source |
|---|---|---|---|---|---|---|---|---|
| **Carbon Monoxide** | 10.1 | mg/cig | 1.01E+01 | 1.01E+01 | 0.72 | CoA 1R6F (University of Kentucky) | 1.40E+01 | CoA 1R6F (University of Kentucky) |

## 3 Intense (ISO 20778:2018) Combustible Reference Cigarette Constituent Values

| Parameter/ Constituent BLUE= 3R4F BLACK=1R6F | ISO 20778: 2018 Mean | Unit | Imputed Formatted | Unit Conversion (mg) | Nicotine (mg/cig) | Nicotine Concentration Source | Nicotine Normalized (mg/mg nicotine) | Analyte Concentration Source |
|---|---|---|---|---|---|---|---|---|
| **Primary Constituents** | | | | | | | | |
| **Nicotine** | 1.896 | mg/cig | 1.90E+00 | 1.90E+00 | 1.90 | CoA 1R6F (University of Kentucky) | 1.00E+00 | CoA 1R6F (University of Kentucky) |
| **Glycerol** | 2.08 | mg/cig | 2.08E+00 | 2.08E+00 | 1.74 | St. Helen et al. 2018 | 1.20E+00 | St. Helen et al. 2018 |
| **Propylene Glycol** | 23.7 | mcg/cig | 2.37E+01 | 2.37E-02 | 1.74 | St. Helen et al. 2018 | 1.36E-02 | St. Helen et al. 2018 |
| **Water** | 15.8 | mg/cig | 1.58E+01 | 1.58E+01 | 1.99 | Jaccard et al. 2019 | 7.94E+00 | Schaller et al. 2016 |
| **Menthol** | <0.01 | mcg/cig | <0.01 | 1.00E-02 | 2.10 | Uchiyama et al. 2018 | ~4.76E-03 | Uchiyama et al. 2018 |
| **Diethylene Glycol** | BLOD | mg/cig | BLOD [~0.00] | 2.00E-03 | 2.02 | Forster et al. 2018 | ~9.90E-04 | Forster et al. 2018, Internal Study LOD/LOQ |
| **Ethylene Glycol** | 0.035 | mg/cig | 3.50E-02 | 3.50E-02 | 2.02 | Forster et al. 2018 | 1.73E-02 | Forster et al. 2018 |
| **Carbonyls & Glycidol** | | | | | | | | |
| **Acetaldehyde** | 1552 | mcg/cig | 1.55E+03 | 1.55E+00 | 1.90 | CoA 1R6F (University of Kentucky) | 8.19E-01 | CoA 1R6F (University of Kentucky) |
| **Acrolein** | 154 | mcg/cig | 1.54E+02 | 1.54E-01 | 1.90 | CoA 1R6F (University of Kentucky) | 8.12E-02 | CoA 1R6F (University of Kentucky) |

| Parameter/ Constituent BLUE= 3R4F BLACK=1R6F | ISO 20778: 2018 Mean | Unit | Imputed Formatted | Unit Conversion (mg) | Nicotine (mg/cig) | Nicotine Concentration Source | Nicotine Normalized (mg/mg nicotine) | Analyte Concentration Source |
|---|---|---|---|---|---|---|---|---|
| **Diacetyl** | 336.93 | mcg/cig | 3.37E+02 | 3.37E-01 | 1.99 | Jaccard et al. 2019 | 1.69E-01 | Moldoveanu et al. 2017 |
| **Formaldehyde** | 104.00 | mcg/cig | 1.04E+02 | 1.04E-01 | 1.90 | Jaccard et al. 2019 | 5.47E-02 | Jaccard et al. 2019 |
| **Glycidol** | 1.76 | mcg/cig | 1.76E+00 | 1.76E-03 | 1.74 | St. Helen et al. 2018 | 1.01E-03 | Jaccard et al. 2019 |
| **Acetoin** | BLOQ | mg/cig | BLOQ [~3.65] | 3.65E+00 | 2.02 | Forster et al. 2018 | ~1.80E+00 | Forster et al. 2018, Internal Study LOD/LOQ |
| **Acetyl Propionyl** | 91.87 | mcg/cig | 9.19E+01 | 9.19E-02 | 1.99 | Jaccard et al. 2019 | 4.62E-02 | Moldoveanu et al. 2017 |
| **Butyraldehyde** | 80.30 | mcg/cig | 8.03E+01 | 8.03E-02 | 1.90 | Jaccard et al. 2019 | 4.23E-02 | Jaccard et al. 2019 |
| **Crotonaldehyde** | 55.00 | mcg/cig | 5.50E+01 | 5.50E-02 | 1.90 | Jaccard et al. 2019 | 2.89E-02 | Jaccard et al. 2019 |
| **Furfural** | 25.9 | mcg/cig | 2.59E+01 | 2.59E-02 | 1.74 | St. Helen et al. 2018 | 1.49E-02 | St. Helen et al. 2018 |
| **Nicotine Degradants & TSNAs** | | | | | | | | |
| **Beta Nicotyrine** | 7071.00 | ng/cig | 7.07E+03 | 7.07E-03 | 2.02 | Forster et al. 2018 | 3.50E-03 | Forster et al. 2018 |
| **Cotinine** | 14320.00 | ng/cig | 1.43E+04 | 1.43E-02 | 2.02 | Forster et al. 2018 | 7.09E-03 | Forster et al. 2018 |
| **Myosmine** | 13226.00 | ng/cig | 1.32E+04 | 1.32E-02 | 2.02 | Forster et al. 2018 | 6.55E-03 | Forster et al. 2018 |
| **Nicotine N Oxide** | BLOQ | mcg/cig | BLOQ [~0.63] | 6.30E-04 | 1.93 | GRPT-02816 | ~3.26E-04 | Internal Study |
| **Nornicotine** | 22117.00 | ng/cig | 2.21E+04 | 2.21E-02 | 2.02 | Forster et al. 2018 | 1.09E-02 | Forster et al. 2018 |
| **Anabasine** | 1030.00 | ng/cig | 1.03E+03 | 1.03E-03 | 2.02 | Forster et al. 2018 | 5.10E-04 | Forster et al. 2018 |
| **Anatabine** | 6218.00 | ng/cig | 6.22E+03 | 6.22E-03 | 2.02 | Forster et al. 2018 | 3.08E-03 | Forster et al. 2018 |
| **NNK** | 187 | ng/cig | 1.87E+02 | 1.87E-04 | 1.90 | CoA 1R6F (University of Kentucky) | 9.86E-05 | CoA 1R6F (University of Kentucky) |

| Parameter/ Constituent BLUE= 3R4F BLACK=1R6F | ISO 20778: 2018 Mean | Unit | Imputed Formatted | Unit Conversion (mg) | Nicotine (mg/cig) | Nicotine Concentration Source | Nicotine Normalized (mg/mg nicotine) | Analyte Concentration Source |
|---|---|---|---|---|---|---|---|---|
| **NNN** | 212 | ng/cig | 2.12E+02 | 2.12E-04 | 1.90 | CoA 1R6F (University of Kentucky) | 1.12E-04 | CoA 1R6F (University of Kentucky) |
| **Metals** | | | | | | | | |
| **Cadmium** | 76.10 | ng/cig | 7.61E+01 | 7.61E-05 | 1.90 | Jaccard et al. 2019 | 4.01E-05 | Jaccard et al. 2019 |
| **Chromium** | BLOD | ng/cig | BLOD [~0.75] | 7.50E-07 | 1.90 | Jaccard et al. 2019 | ~3.95E-07 | Forster et al. 2018, Internal Study LOD/LOQ |
| **Copper** | 24.80 | ng/cig | 2.48E+01 | 2.48E-05 | 2.02 | Forster et al. 2018 | 1.23E-05 | Forster et al. 2018 |
| **Lead** | 31.2 | ng/cig | 3.12E+01 | 3.12E-05 | 1.74 | St. Helen et al. 2018 | 1.79E-05 | St. Helen et al. 2018 |
| **Nickel** | BLOD | ng/cig | BLOD [~1.50] | 1.50E-06 | 1.90 | Jaccard et al. 2019 | ~7.89E-07 | Forster et al. 2018, Internal Study LOD/LOQ |
| **Organic Acids** | | | | | | | | |
| **Benzoic Acid** | NA | NA | NA | NA | NA | NA | NA | NA |
| **Acetic Acid** | NA | NA | NA | NA | NA | NA | NA | NA |
| **Lactic Acid** | NA | NA | NA | NA | NA | NA | NA | NA |
| **Propionic Acid** | NA | NA | NA | NA | NA | NA | NA | NA |
| **VOCs** | | | | | | | | |
| **Acrylonitrile** | 24.00 | mcg/cig | 2.40E+01 | 2.40E-02 | 1.90 | CoA 1R6F (University of Kentucky) | 1.27E-02 | CoA 1R6F (University of Kentucky) |
| **Benzene** | 88 | mcg/cig | 8.80E+01 | 8.80E-02 | 1.9 | CoA 1R6F (University of Kentucky) | 4.67E-02 | CoA 1R6F (University of Kentucky) |
| **Propylene oxide** | 1710 | ng/cig | 1.71E+03 | 1.71E-03 | 1.9 | Jaccard et al. 2019 | 9.00E-04 | Jaccard et al. 2019 |

| Parameter/ Constituent BLUE= 3R4F BLACK=1R6F | ISO 20778: 2018 Mean | Unit | Imputed Formatted | Unit Conversion (mg) | Nicotine (mg/cig) | Nicotine Concentration Source | Nicotine Normalized (mg/mg nicotine) | Analyte Concentration Source |
|---|---|---|---|---|---|---|---|---|
| **Toluene** | 150 | mcg/cig | 1.50E+02 | 1.50E-01 | 1.9 | CoA 1R6F (University of Kentucky) | 7.91E-02 | CoA 1R6F (University of Kentucky) |
| **Esters and Alcohols** | | | | | | | | |
| **1-Butanol** | NA | NA | NA | NA | NA | NA | NA | NA |
| **Benzyl acetate** | NA | NA | NA | NA | NA | NA | NA | NA |
| **Ethyl Acetate** | NA | NA | NA | NA | NA | NA | NA | NA |
| **Ethyl acetoacetate** | NA | NA | NA | NA | NA | NA | NA | NA |
| **Isoamyl acetate** | NA | NA | NA | NA | NA | NA | NA | NA |
| **Isobutyl acetate** | NA | NA | NA | NA | NA | NA | NA | NA |
| **Methyl Acetate** | NA | NA | NA | NA | NA | NA | NA | NA |
| **Carbon Monoxide** | | | | | | | | |
| **Carbon Monoxide** | 28.0 | mg/cig | 2.80E+01 | 2.80E+01 | 1.90 | CoA 1R6F (University of Kentucky) | 1.48E+01 | CoA 1R6F (University of Kentucky) |
