# Supplementary material for: A Practical Framework for Novel Electronic Nicotine Delivery System Evaluation: Chemical and Toxicological Characterization of JUUL2 Aerosol and Comparison with Reference Cigarettes
Source: Toxics. 2024 Jan 4;12(1):41. doi: 10.3390/toxics12010041 (PMC10820849; doi:10.3390/toxics12010041)
Supplement: Supplementary file 1 [file toxics-12-00041-s001.zip › Supplementary_Material_File S5_Percent Difference to Combustible Cigarettes_Final_Clean.pdf]

Supplementary Materials File S5

1 Percent Difference to Combustible Cigarettes

1.1 Virginia Tobacco 18 mg/mL Non-Intense Percent Difference to Combustible Cigarettes

Parameter/ Constituent	Aerosol or Smoke Yields Normalized by Nicotine (mg/mg)		% Diff vs CC
	AEROSOL MAX	CC	
**# of puffs**	150	NA	NA
**pH**	6.19	NA	NA
**Primary Constituents**			
**Nicotine**	1.00E+00	1.00E+00	NA
**Glycerol**	4.35E+01	8.95E-01	↑ 4762.55%
**Propylene Glycol**	2.50E+01	4.21E-03	↑ 593469.80%
**Water**	5.68E+00	4.21E+00	↑ 34.96%
**Menthol**	~ 5.69E-02	~1.32E-05	NC
**Diethylene Glycol**	~ 5.33E-03	~1.42E-03	NC
**Ethylene Glycol**	~ 5.71E-03	9.96E-03	↓ ≥ 42.68%
**Carbonyls & Glycidol**			
**Acetaldehyde**	2.74E-04	7.24E-01	↓ 99.96%
**Acrolein**	1.78E-04	5.96E-02	↓ 99.70%
**Diacetyl**	~ 3.90E-05	1.64E-01	↓ ≥ 99.98%
**Formaldehyde**	1.13E-03	3.74E-02	↓ 96.98%
**Glycidol**	2.39E-04	1.04E-03	↓ 76.91%
**Acetyl Propionyl**	~ 3.58E-06	4.21E-02	↓ ≥ 99.99%
**Butyraldehyde**	~ 3.64E-06	3.33E-02	↓ ≥ 99.99%
**Crotonaldehyde**	~ 5.74E-06	1.53E-02	↓ ≥ 99.96%
**Furfural**	~ 5.88E-06	1.84E-02	↓ ≥ 99.97%
**Nicotine Degradants & TSNAs**			
**β-Nicotyrine**	1.35E-04	1.07E-02	↓ 98.73%
**Cotinine**	2.41E-03	6.96E-03	↓ 65.35%
**Myosmine**	1.99E-03	NA	NA
**Nicotine N Oxide**	7.73E-05	~5.37E-04	NC
**Nornicotine**	7.91E-04	9.50E-03	↓ 91.67%
**Anabasine**	~ 2.88E-06	8.33E-04	↓ ≥ 99.65%
**Anatabine**	~ 2.66E-05	6.00E-03	↓ ≥ 99.56%
**NNK**	~ 7.49E-07	9.85E-05	↓ ≥ 99.24%
**NNN**	~ 8.99E-08	1.18E-04	↓ ≥ 99.92%
**Metals**			
**Arsenic**	~ 1.78E-06	NA	NA

**Beryllium**	~ 6.97E-08	NA	NA
**Cadmium**	~ 3.17E-08	3.35E-05	↓ ≥ 99.91%
**Chromium**	~ 1.90E-06	~6.27E-07	NC
**Cobalt**	~ 1.58E-08	NA	NA
**Copper**	~ 2.72E-06	1.64E-05	↓ ≥ 83.36%
**Gold**	~ 4.63E-06	NA	NA
**Iron**	~ 8.17E-06	NA	NA
**Lead**	~ 7.92E-08	~4.18E-07	NC
**Nickel**	~ 1.58E-07	~9.64E-07	NC
**Selenium**	1.40E-06	NA	NA
**Silver**	~ 3.17E-08	NA	NA
**Tin**	~ 1.63E-06	NA	NA
**Zinc**	~ 1.36E-05	NA	NA
**Organic Acids**			
**Benzoic Acid**	5.67E-01	NA	NA
**Propionic Acid**	~ 1.76E-03	NA	NA
**VOCs**			
**1,3-Butadiene**	~ 1.49E-06	NA	NA
**Acrylonitrile**	~ 9.79E-07	9.71E-03	↓ ≥ 99.99%
**Benzene**	~ 1.71E-06	4.58E-02	↓ ≥ 99.99%
**Isoprene**	~ 2.30E-06	NA	NA
**Propylene Oxide**	~ 3.96E-06	8.44E-01	↓ ≥ 99.99%
**Toluene**	~ 1.21E-05	7.35E-02	↓ ≥ 99.98%
**Esters & Alcohols**			
**1-Butanol**	~ 4.41E-05	NA	NA
**Benzyl Acetate**	~ 6.84E-05	NA	NA
**Ethyl Acetate**	~ 7.83E-05	NA	NA
**Ethyl Acetoacetate**	~ 5.59E-05	NA	NA
**Isoamyl Acetate**	~ 4.77E-05	NA	NA
**Isobutyl Acetate**	~ 4.73E-05	NA	NA
**Methyl Acetate**	~ 5.08E-05	NA	NA
		Average Difference	↑ 22927.46%
		Average Difference without PG, VG, and Water	↓ 93.63%

1.2 Virginia Tobacco 18 mg/mL Intense Percent Difference to Combustible Cigarettes

**Parameter/ Constituent**	**Aerosol or Smoke Yields Normalized by Nicotine (mg/mg)**		**% Diff vs CC**
	**AEROSOL MAX**	**CC**	
**# of puffs**	87	NA	NA

**pH**	6.46	NA	NA
**Primary Constituents**			
**Nicotine**	1.00E+00	1.00E+00	NA
**Glycerol**	4.16E+01	1.20E+00	↑ 3375.92%
**Propylene Glycol**	2.53E+01	1.36E-02	↑ 185337.92%
**Water**	5.00E+00	7.94E+00	↓ 37.02%
**Menthol**	~ 4.74E-03	~4.76E-06	NC
**Diethylene Glycol**	~ 5.00E-03	~9.90E-04	NC
**Ethylene Glycol**	~ 5.36E-03	1.73E-02	↓ ≥ 69.09%
**Carbonyls & Glycidol**			
**Acetaldehyde**	3.96E-04	8.19E-01	↓ 99.95%
**Acrolein**	4.05E-04	8.12E-02	↓ 99.50%
**Diacetyl**	~ 4.27E-05	1.69E-01	↓ ≥ 99.97%
**Formaldehyde**	1.48E-03	5.47E-02	↓ 97.29%
**Glycidol**	3.37E-04	1.01E-03	↓ 66.69%
**Acetyl Propionyl**	~ 3.92E-06	4.62E-02	↓ ≥ 99.99%
**Butyraldehyde**	~ 4.01E-05	4.23E-02	↓ ≥ 99.91%
**Crotonaldehyde**	~ 6.29E-06	2.89E-02	↓ ≥ 99.98%
**Furfural**	~ 4.32E-05	1.49E-02	↓ ≥ 99.71%
**Nicotine Degradants & TSNAs**			
**β-Nicotyrine**	1.74E-04	3.50E-03	↓ 95.04%
**Cotinine**	2.54E-03	7.09E-03	↓ 64.14%
**Myosmine**	1.98E-03	6.55E-03	↓ 69.80%
**Nicotine N Oxide**	6.33E-05	~3.26E-04	NC
**Nornicotine**	8.71E-04	1.09E-02	↓ 92.05%
**Anabasine**	~ 3.15E-06	5.10E-04	↓ ≥ 99.38%
**Anatabine**	~ 3.26E-05	3.08E-03	↓ ≥ 98.94%
**NNK**	~ 7.83E-07	9.86E-05	↓ ≥ 99.21%
**NNN**	~ 9.41E-08	1.12E-04	↓ ≥ 99.92%
**Metals**			
**Arsenic**	~ 2.97E-07	NA	NA
**Beryllium**	~ 6.54E-08	NA	NA
**Cadmium**	~ 2.97E-08	4.01E-05	↓ ≥ 99.93%
**Chromium**	4.11E-06	~3.95E-07	↑ ≥ 940.13%
**Cobalt**	~ 1.49E-08	NA	NA
**Copper**	~ 7.43E-07	1.23E-05	↓ ≥ 93.95%
**Gold**	~ 4.84E-06	NA	NA
**Iron**	3.40E-05	NA	NA
**Lead**	~ 7.43E-08	1.79E-05	↓ ≥ 99.59%
**Nickel**	~ 4.27E-07	~7.89E-07	NC

**Selenium**	~ 7.11E-07	NA	NA
**Silver**	~ 1.64E-07	NA	NA
**Tin**	~ 1.71E-06	NA	NA
**Zinc**	~ 4.46E-06	NA	NA
**Organic Acids**			
**Benzoic Acid**	7.84E-01	NA	NA
**Propionic Acid**	~ 2.43E-03	NA	NA
**VOCs**			
**1,3-Butadiene**	~ 1.56E-06	NA	NA
**Acrylonitrile**	~ 1.02E-06	1.27E-02	↓ ≥ 99.99%
**Benzene**	~ 1.79E-06	4.64E-02	↓ ≥ 99.99%
**Isoprene**	~ 2.41E-06	NA	NA
**Propylene Oxide**	~ 4.15E-06	9.00E-04	↓ ≥ 99.54%
**Toluene**	~ 1.27E-05	7.91E-02	↓ ≥ 99.98%
**Esters & Alcohols**			
**1-Butanol**	~ 4.61E-05	NA	NA
**Benzyl Acetate**	~ 7.16E-05	NA	NA
**Ethyl Acetate**	~ 8.19E-05	NA	NA
**Ethyl Acetoacetate**	~ 5.85E-05	NA	NA
**Isoamyl Acetate**	~ 4.99E-05	NA	NA
**Isobutyl Acetate**	~ 4.94E-05	NA	NA
**Methyl Acetate**	~ 5.32E-05	NA	NA
		Average Difference	↑ 6654.76%
		Average Difference without PG, VG, Water, and Chromium	↓ 93.74%

1.3 Crisp Menthol 18 mg/mL Non-Intense Percent Difference to Combustible Cigarettes

Parameter/ Constituent	Aerosol or Smoke Yields Normalized by Nicotine (mg/mg)		% Diff vs CC
	AEROSOL MAX	CC	
**# of puffs**	125	NA	NA
**pH**	6.36	NA	NA
**Primary Constituents**			
**Nicotine**	1.00E+00	1.00E+00	NA
**Glycerol**	2.69E+01	8.95E-01	↑ 2901.15%
**Propylene Glycol**	3.80E+01	4.21E-03	↑ 902232.62%
**Water**	4.66E+00	4.21E+00	↑ 10.57%
**Menthol**	7.39E-01	~1.32E-05	↑ ≥ 5615211.37%
**Diethylene Glycol**	~ 5.19E-03	~1.42E-03	NC
**Ethylene Glycol**	~ 5.55E-03	9.96E-03	↓ ≥ 44.22%
**Carbonyls & Glycidol**			

**Acetaldehyde**	1.67E-04	7.24E-01	↓ 99.98%
**Acrolein**	1.06E-04	5.96E-02	↓ 99.82%
**Diacetyl**	~ 3.83E-05	1.64E-01	↓ ≥ 99.98%
**Formaldehyde**	7.17E-04	3.74E-02	↓ 98.09%
**Glycidol**	1.66E-04	1.04E-03	↓ 84.02%
**Acetyl Propionyl**	~ 3.49E-06	4.21E-02	↓ ≥ 99.99%
**Butyraldehyde**	~ 3.69E-06	3.33E-02	↓ ≥ 99.99%
**Crotonaldehyde**	~ 5.59E-06	1.53E-02	↓ ≥ 99.96%
**Furfural**	~ 5.72E-06	1.84E-02	↓ ≥ 99.97%
**Nicotine Degradants & TSNAs**			
**β-Nicotyrine**	2.46E-04	1.07E-02	↓ 97.69%
**Cotinine**	~ 3.07E-05	6.96E-03	↓ ≥ 99.56%
**Myosmine**	1.66E-04	NA	NA
**Nicotine N Oxide**	1.03E-04	~5.37E-04	NC
**Nornicotine**	8.85E-04	9.50E-03	↓ 90.68%
**Anabasine**	~ 2.80E-06	8.33E-04	↓ ≥ 99.66%
**Anatabine**	~ 2.79E-06	6.00E-03	↓ ≥ 99.95%
**NNK**	~ 7.85E-07	9.85E-05	↓ ≥ 99.20%
**NNN**	~ 9.42E-08	1.18E-04	↓ ≥ 99.92%
**Metals**			
**Arsenic**	~ 3.08E-07	NA	NA
**Beryllium**	~ 6.78E-08	NA	NA
**Cadmium**	~ 3.08E-08	3.35E-05	↓ ≥ 99.91%
**Chromium**	~ 1.71E-06	~6.27E-07	NC
**Cobalt**	~ 1.54E-08	NA	NA
**Copper**	~ 2.85E-06	1.64E-05	↓ ≥ 82.56%
**Gold**	~ 4.85E-06	NA	NA
**Iron**	1.22E-05	NA	NA
**Lead**	~ 7.71E-08	~4.18E-07	NC
**Nickel**	~ 1.54E-07	~9.64E-07	NC
**Selenium**	~ 7.37E-07	NA	NA
**Silver**	~ 3.08E-08	NA	NA
**Tin**	~ 1.81E-06	NA	NA
**Zinc**	~ 1.43E-05	NA	NA
**Organic Acids**			
**Benzoic Acid**	6.20E-01	NA	NA
**Propionic Acid**	~ 1.20E-03	NA	NA
**VOCs**			
**1,3-Butadiene**	~ 1.57E-06	NA	NA
**Acrylonitrile**	~ 1.03E-06	9.71E-03	↓ ≥ 99.99%

**Benzene**	~ 1.80E-06	4.58E-02	↓ ≥ 99.99%
**Isoprene**	~ 2.41E-06	NA	NA
**Propylene Oxide**	~ 4.15E-06	8.44E-01	↓ ≥ 99.99%
**Toluene**	~ 2.64E-06	7.35E-02	↓ ≥ 99.99%
**Esters & Alcohols**			
**1-Butanol**	~ 4.62E-05	NA	NA
**Benzyl Acetate**	~ 7.17E-05	NA	NA
**Ethyl Acetate**	6.31E-03	NA	NA
**Ethyl Acetoacetate**	~ 5.86E-05	NA	NA
**Isoamyl Acetate**	~ 5.00E-05	NA	NA
**Isobutyl Acetate**	~ 4.95E-05	NA	NA
**Methyl Acetate**	~ 5.33E-05	NA	NA
		Average Difference	↑ 241413.36%
		Average Difference without PG, VG, Water, and Menthol	↓ 95.44%

1.4 Crisp Menthol 18 mg/mL Intense Percent Difference to Combustible Cigarettes

**Parameter/ Constituent**	**Aerosol or Smoke Yields Normalized by Nicotine (mg/mg)**		**% Diff vs CC**
	**AEROSOL MAX**	**CC**	
**# of puffs**	88	NA	NA
**pH**	6.53	NA	NA
**Primary Constituents**			
**Nicotine**	1.00E+00	1.00E+00	NA
**Glycerol**	2.68E+01	1.20E+00	↑ 2143.99%
**Propylene Glycol**	3.76E+01	1.36E-02	↑ 276111.72%
**Water**	4.29E+00	7.94E+00	↓ 45.95%
**Menthol**	7.21E-01	~4.76E-06	↑ ≥ 15145814.61%
**Diethylene Glycol**	~ 4.67E-03	~9.90E-04	NC
**Ethylene Glycol**	~ 4.97E-03	1.73E-02	↓ ≥ 71.34%
**Carbonyls & Glycidol**			
**Acetaldehyde**	2.23E-04	8.19E-01	↓ 99.97%
**Acrolein**	2.20E-04	8.12E-02	↓ 99.73%
**Diacetyl**	~ 4.28E-05	1.69E-01	↓ ≥ 99.97%
**Formaldehyde**	1.04E-03	5.47E-02	↓ 98.10%
**Glycidol**	2.80E-04	1.01E-03	↓ 72.35%
**Acetyl Propionyl**	~ 4.96E-06	4.62E-02	↓ ≥ 99.99%
**Butyraldehyde**	~ 8.46E-06	4.23E-02	↓ ≥ 99.98%
**Crotonaldehyde**	~ 1.01E-05	2.89E-02	↓ ≥ 99.97%
**Furfural**	~ 8.32E-06	1.49E-02	↓ ≥ 99.94%
**Nicotine Degradants & TSNAs**			

**β-Nicotyrine**	2.09E-04	3.50E-03	↓ 94.04%
**Cotinine**	6.08E-05	7.09E-03	↓ 99.14%
**Myosmine**	1.58E-04	6.55E-03	↓ 97.59%
**Nicotine N Oxide**	6.36E-05	~3.26E-04	NC
**Nornicotine**	7.68E-04	1.09E-02	↓ 92.99%
**Anabasine**	~ 2.98E-06	5.10E-04	↓ ≥ 99.42%
**Anatabine**	~ 2.97E-06	3.08E-03	↓ ≥ 99.90%
**NNK**	~ 7.64E-07	9.86E-05	↓ ≥ 99.23%
**NNN**	~ 9.18E-08	1.12E-04	↓ ≥ 99.92%
**Metals**			
**Arsenic**	~ 2.80E-07	NA	NA
**Beryllium**	~ 6.16E-08	NA	NA
**Cadmium**	~ 2.80E-08	4.01E-05	↓ ≥ 99.93%
**Chromium**	~ 1.67E-06	~3.95E-07	NC
**Cobalt**	~ 1.40E-08	NA	NA
**Copper**	~ 7.00E-07	1.23E-05	↓ ≥ 94.30%
**Gold**	~ 4.72E-06	NA	NA
**Iron**	1.23E-05	NA	NA
**Lead**	~ 7.00E-08	1.79E-05	↓ ≥ 99.61%
**Nickel**	~ 1.40E-07	~7.89E-07	NC
**Selenium**	~ 6.69E-07	NA	NA
**Silver**	~ 1.66E-07	NA	NA
**Tin**	~ 1.68E-06	NA	NA
**Zinc**	~ 4.20E-06	NA	NA
**Organic Acids**			
**Benzoic Acid**	7.34E-01	NA	NA
**Propionic Acid**	~ 1.06E-03	NA	NA
**VOCs**			
**1,3-Butadiene**	~ 1.53E-06	NA	NA
**Acrylonitrile**	~ 9.99E-07	1.27E-02	↓ ≥ 99.99%
**Benzene**	~ 1.75E-06	4.64E-02	↓ ≥ 99.99%
**Isoprene**	~ 2.35E-06	NA	NA
**Propylene Oxide**	~ 4.05E-06	9.00E-04	↓ ≥ 99.55%
**Toluene**	~ 1.24E-05	7.91E-02	↓ ≥ 99.98%
**Esters & Alcohols**			
**1-Butanol**	~ 4.50E-05	NA	NA
**Benzyl Acetate**	~ 6.98E-05	NA	NA
**Ethyl Acetate**	6.60E-03	NA	NA
**Ethyl Acetoacetate**	~ 5.71E-05	NA	NA
**Isoamyl Acetate**	~ 4.87E-05	NA	NA

**Isobutyl Acetate**	~ 4.82E-05	NA	NA
**Methyl Acetate**	~ 5.19E-05	NA	NA
		Average Difference	↑ 531779.57%
		Average Difference without PG, VG, Water, and Menthol	↓ 96.68%

1.5 Polar Menthol 18 mg/mL Non-Intense Percent Difference to Combustible Cigarettes

Parameter/ Constituent	Aerosol or Smoke Yields Normalized by Nicotine (mg/mg)		% Diff vs CC
	AEROSOL MAX	CC	
**# of puffs**	132	NA	NA
**pH**	6.55	NA	NA
**Primary Constituents**			
**Nicotine**	1.00E+00	1.00E+00	NA
**Glycerol**	2.72E+01	8.95E-01	↑ 2943.60%
**Propylene Glycol**	4.50E+01	4.21E-03	↑ 1067675.68%
**Water**	4.92E+00	4.21E+00	↑ 16.79%
**Menthol**	7.88E-01	~1.32E-05	↑ ≥ 5991318.81%
**Diethylene Glycol**	~ 5.53E-03	~1.42E-03	NC
**Ethylene Glycol**	~ 5.92E-03	9.96E-03	↓ ≥ 40.53%
**Carbonyls & Glycidol**			
**Acetaldehyde**	2.90E-04	7.24E-01	↓ 99.96%
**Acrolein**	1.28E-04	5.96E-02	↓ 99.78%
**Diacetyl**	~ 3.87E-06	1.64E-01	↓ ≥ 99.99%
**Formaldehyde**	8.77E-04	3.74E-02	↓ 97.66%
**Glycidol**	1.64E-04	1.04E-03	↓ 84.13%
**Acetyl Propionyl**	~ 3.72E-06	4.21E-02	↓ ≥ 99.99%
**Butyraldehyde**	~ 3.82E-06	3.33E-02	↓ ≥ 99.99%
**Crotonaldehyde**	~ 5.96E-06	1.53E-02	↓ ≥ 99.96%
**Furfural**	~ 6.10E-06	1.84E-02	↓ ≥ 99.97%
**Nicotine Degradants & TSNAs**			
**β-Nicotyrine**	3.08E-04	1.07E-02	↓ 97.11%
**Cotinine**	1.57E-04	6.96E-03	↓ 97.74%
**Myosmine**	4.00E-04	NA	NA
**Nicotine N Oxide**	1.17E-04	~5.37E-04	NC
**Nornicotine**	9.36E-04	9.50E-03	↓ 90.15%
**Anabasine**	~ 2.99E-06	8.33E-04	↓ ≥ 99.64%
**Anatabine**	~ 3.27E-05	6.00E-03	↓ ≥ 99.45%
**NNK**	~ 8.58E-07	9.85E-05	↓ ≥ 99.13%
**NNN**	~ 1.03E-07	1.18E-04	↓ ≥ 99.91%
**Metals**			

**Arsenic**	~ 1.81E-06	NA	NA
**Beryllium**	~ 7.23E-08	NA	NA
**Cadmium**	~ 3.29E-08	3.35E-05	↓ ≥ 99.90%
**Chromium**	~ 1.87E-06	~6.27E-07	NC
**Cobalt**	~ 1.64E-08	NA	NA
**Copper**	~ 3.29E-06	1.64E-05	↓ ≥ 79.90%
**Gold**	~ 5.31E-06	NA	NA
**Iron**	~ 9.36E-06	NA	NA
**Lead**	~ 8.22E-08	~4.18E-07	NC
**Nickel**	~ 4.53E-07	~9.64E-07	NC
**Selenium**	1.21E-06	NA	NA
**Silver**	~ 3.29E-08	NA	NA
**Tin**	~ 3.29E-07	NA	NA
**Zinc**	~ 1.56E-05	NA	NA
**Organic Acids**			
**Benzoic Acid**	5.81E-01	NA	NA
**Propionic Acid**	~ 1.33E-03	NA	NA
**VOCs**			
**1,3-Butadiene**	~ 1.71E-06	NA	NA
**Acrylonitrile**	~ 1.12E-06	9.71E-03	↓ ≥ 99.99%
**Benzene**	~ 1.97E-06	4.58E-02	↓ ≥ 99.99%
**Isoprene**	~ 2.64E-06	NA	NA
**Propylene Oxide**	~ 4.54E-06	8.44E-01	↓ ≥ 99.99%
**Toluene**	~ 2.89E-06	7.35E-02	↓ ≥ 99.99%
**Esters & Alcohols**			
**1-Butanol**	~ 5.06E-05	NA	NA
**Benzyl Acetate**	~ 7.84E-05	NA	NA
**Ethyl Acetate**	~ 9.87E-04	NA	NA
**Ethyl Acetoacetate**	~ 6.41E-05	NA	NA
**Isoamyl Acetate**	~ 5.47E-05	NA	NA
**Isobutyl Acetate**	~ 5.42E-05	NA	NA
**Methyl Acetate**	~ 5.82E-05	NA	NA
		Average Difference	↑ 261472.96%
		Average Difference without PG, VG, Water, and Menthol	↓ 94.99%

1.6 Polar Menthol 18 mg/mL Intense Percent Difference to Combustible Cigarettes

**Parameter/ Constituent**	**Aerosol or Smoke Yields Normalized by Nicotine (mg/mg)**		**% Diff vs CC**
	**AEROSOL MAX**	**CC**	
**# of puffs**	81	NA	NA
**pH**	6.57	NA	NA

**Primary Constituents**			
**Nicotine**	1.00E+00	1.00E+00	NA
**Glycerol**	2.74E+01	1.20E+00	↑ 2187.99%
**Propylene Glycol**	4.04E+01	1.36E-02	↑ 296295.19%
**Water**	4.21E+00	7.94E+00	↓ 46.98%
**Menthol**	7.70E-01	~4.76E-06	↑ ≥ 16168585.56%
**Diethylene Glycol**	~ 4.71E-03	~9.90E-04	NC
**Ethylene Glycol**	~ 5.02E-03	1.73E-02	↓ ≥ 71.04%
**Carbonyls & Glycidol**			
**Acetaldehyde**	3.14E-04	8.19E-01	↓ 99.96%
**Acrolein**	2.79E-04	8.12E-02	↓ 99.66%
**Diacetyl**	~ 4.33E-05	1.69E-01	↓ ≥ 99.97%
**Formaldehyde**	8.65E-04	5.47E-02	↓ 98.42%
**Glycidol**	2.06E-04	1.01E-03	↓ 79.64%
**Acetyl Propionyl**	~ 5.03E-06	4.62E-02	↓ ≥ 99.99%
**Butyraldehyde**	~ 8.57E-06	4.23E-02	↓ ≥ 99.98%
**Crotonaldehyde**	~ 1.02E-05	2.89E-02	↓ ≥ 99.96%
**Furfural**	~ 8.43E-06	1.49E-02	↓ ≥ 99.94%
**Nicotine Degradants & TSNAs**			
**β-Nicotyrine**	3.38E-04	3.50E-03	↓ 90.35%
**Cotinine**	1.15E-04	7.09E-03	↓ 98.37%
**Myosmine**	3.28E-04	6.55E-03	↓ 94.99%
**Nicotine N Oxide**	~ 4.01E-05	~3.26E-04	NC
**Nornicotine**	7.08E-04	1.09E-02	↓ 93.54%
**Anabasine**	~ 2.98E-05	5.10E-04	↓ ≥ 94.15%
**Anatabine**	~ 3.31E-05	3.08E-03	↓ ≥ 98.93%
**NNK**	~ 7.71E-07	9.86E-05	↓ ≥ 99.22%
**NNN**	~ 9.26E-08	1.12E-04	↓ ≥ 99.92%
**Metals**			
**Arsenic**	~ 1.71E-06	NA	NA
**Beryllium**	~ 6.26E-08	NA	NA
**Cadmium**	~ 2.85E-08	4.01E-05	↓ ≥ 99.93%
**Chromium**	~ 1.68E-06	~3.95E-07	NC
**Cobalt**	~ 1.42E-08	NA	NA
**Copper**	~ 7.12E-07	1.23E-05	↓ ≥ 94.20%
**Gold**	~ 4.77E-06	NA	NA
**Iron**	~ 8.42E-06	NA	NA
**Lead**	~ 7.12E-08	1.79E-05	↓ ≥ 99.60%
**Nickel**	~ 4.23E-07	~7.89E-07	NC

**Selenium**	1.40E-06	NA	NA
**Silver**	~ 1.69E-07	NA	NA
**Tin**	~ 1.71E-06	NA	NA
**Zinc**	~ 1.42E-05	NA	NA
**Organic Acids**			
**Benzoic Acid**	7.22E-01	NA	NA
**Propionic Acid**	~ 1.76E-03	NA	NA
**VOCs**			
**1,3-Butadiene**	~ 1.54E-06	NA	NA
**Acrylonitrile**	~ 1.01E-06	1.27E-02	↓ ≥ 99.99%
**Benzene**	~ 1.77E-06	4.64E-02	↓ ≥ 99.99%
**Isoprene**	~ 2.37E-06	NA	NA
**Propylene Oxide**	~ 4.08E-06	9.00E-04	↓ ≥ 99.55%
**Toluene**	~ 2.60E-06	7.91E-02	↓ ≥ 99.99%
**Esters & Alcohols**			
**1-Butanol**	~ 4.54E-05	NA	NA
**Benzyl Acetate**	~ 7.05E-05	NA	NA
**Ethyl Acetate**	~ 8.88E-04	NA	NA
**Ethyl Acetoacetate**	~ 5.76E-05	NA	NA
**Isoamyl Acetate**	~ 4.92E-05	NA	NA
**Isobutyl Acetate**	~ 4.87E-05	NA	NA
**Methyl Acetate**	~ 5.24E-05	NA	NA
		Average Difference	↑ 567745.19%
		Average Difference without PG, VG, Water, and Menthol	↓ 96.45%

1.7 Autumn Tobacco 18 mg/mL Non-Intense Percent Difference to Combustible Cigarettes

**Parameter/ Constituent**	**Aerosol or Smoke Yields Normalized by Nicotine (mg/mg)**		**% Diff vs CC**
	**AEROSOL MAX**	**CC**	
**# of puffs**	128	NA	NA
**pH**	6.41	NA	NA
**Primary Constituents**			
**Nicotine**	1.00E+00	1.00E+00	NA
**Glycerol**	2.59E+01	8.95E-01	↑ 2799.86%
**Propylene Glycol**	3.40E+01	4.21E-03	↑ 807778.37%
**Water**	6.24E+00	4.21E+00	↑ 48.28%
**Menthol**	~ 2.01E-03	~1.32E-05	NC
**Diethylene Glycol**	~ 1.84E-03	~1.42E-03	NC
**Ethylene Glycol**	~ 1.28E-03	9.96E-03	↓ ≥ 87.11%

**Carbonyls & Glycidol**			
**Acetaldehyde**	1.60E-04	7.24E-01	↓ 99.98%
**Acrolein**	7.06E-05	5.96E-02	↓ 99.88%
**Diacetyl**	~ 3.73E-05	1.64E-01	↓ ≥ 99.98%
**Formaldehyde**	5.57E-04	3.74E-02	↓ 98.51%
**Glycidol**	1.61E-04	1.04E-03	↓ 84.43%
**Acetyl Propionyl**	~ 4.29E-06	4.21E-02	↓ ≥ 99.99%
**Butyraldehyde**	~ 3.52E-05	3.33E-02	↓ ≥ 99.89%
**Crotonaldehyde**	~ 5.51E-06	1.53E-02	↓ ≥ 99.96%
**Furfural**	~ 5.88E-06	1.84E-02	↓ ≥ 99.97%
**Nicotine Degradants & TSNAs**			
**β-Nicotyrine**	1.17E-04	1.07E-02	↓ 98.90%
**Cotinine**	1.89E-04	6.96E-03	↓ 97.29%
**Myosmine**	1.88E-04	NA	NA
**Nicotine N Oxide**	2.51E-04	~5.37E-04	NC
**Nornicotine**	5.91E-04	9.50E-03	↓ 93.78%
**Anabasine**	~ 3.69E-06	8.33E-04	↓ ≥ 99.56%
**Anatabine**	~ 2.93E-06	6.00E-03	↓ ≥ 99.95%
**NNK**	~ 9.18E-08	9.85E-05	↓ ≥ 99.91%
**NNN**	~ 2.47E-08	1.18E-04	↓ ≥ 99.98%
**Metals**			
**Arsenic**	~ 1.82E-06	NA	NA
**Beryllium**	~ 6.68E-08	NA	NA
**Cadmium**	~ 3.04E-08	3.35E-05	↓ ≥ 99.91%
**Chromium**	~ 1.80E-06	~6.27E-07	NC
**Cobalt**	~ 1.52E-08	NA	NA
**Copper**	~ 7.59E-07	1.64E-05	↓ ≥ 95.36%
**Gold**	~ 5.16E-06	NA	NA
**Iron**	~ 3.04E-06	NA	NA
**Lead**	~ 7.59E-08	~4.18E-07	NC
**Nickel**	~ 1.52E-07	~9.64E-07	NC
**Selenium**	~ 7.26E-07	NA	NA
**Silver**	~ 3.04E-08	NA	NA
**Tin**	~ 1.80E-06	NA	NA
**Zinc**	~ 4.56E-06	NA	NA
**Organic Acids**			
**Benzoic Acid**	7.08E-01	NA	NA
**Propionic Acid**	~ 1.96E-03	NA	NA
**VOCs**			

**1,3-butadiene**	~ 2.12E-06	NA	NA
**Acrylonitrile**	~ 1.09E-06	9.71E-03	↓ ≥ 99.99%
**Benzene**	~ 1.12E-05	4.58E-02	↓ ≥ 99.98%
**Isoprene**	~ 2.57E-06	NA	NA
**Propylene Oxide**	~ 6.89E-06	8.44E-01	↓ ≥ 99.99%
**Toluene**	~ 1.35E-05	7.35E-02	↓ ≥ 99.98%
**Esters & Alcohols**			
**1-Butanol**	~ 6.54E-04	NA	NA
**Benzyl Acetate**	~ 7.65E-05	NA	NA
**Ethyl Acetate**	~ 6.09E-05	NA	NA
**Ethyl Acetoacetate**	~ 1.19E-04	NA	NA
**Isoamyl Acetate**	~ 6.15E-05	NA	NA
**Isobutyl Acetate**	~ 6.12E-05	NA	NA
**Methyl Acetate**	~ 7.00E-04	NA	NA
		Average Difference	↑ 31091.24%
		Average Difference without PG, VG, and Water	↓ 98.01%

1.8 Autumn Tobacco 18 mg/mL Intense Percent Difference to Combustible Cigarettes

**Parameter/ Constituent**	**Aerosol or Smoke Yields Normalized by Nicotine (mg/mg)**		**% Diff vs CC**
	**AEROSOL MAX**	**CC**	
**# of puffs**	80	NA	NA
**pH**	6.43	NA	NA
**Primary Constituents**			
**Nicotine**	1.00E+00	1.00E+00	NA
**Glycerol**	2.62E+01	1.20E+00	↑ 2095.82%
**Propylene Glycol**	3.41E+01	1.36E-02	↑ 249907.01%
**Water**	5.42E+00	7.94E+00	↓ 31.79%
**Menthol**	~ 4.90E-03	~4.76E-06	NC
**Diethylene Glycol**	~ 2.10E-03	~9.90E-04	NC
**Ethylene Glycol**	~ 1.47E-03	1.73E-02	↓ ≥ 91.53%
**Carbonyls & Glycidol**			
**Acetaldehyde**	1.92E-04	8.19E-01	↓ 99.98%
**Acrolein**	1.27E-04	8.12E-02	↓ 99.84%
**Diacetyl**	~ 4.84E-05	1.69E-01	↓ ≥ 99.97%
**Formaldehyde**	6.99E-04	5.47E-02	↓ 98.72%
**Glycidol**	2.70E-04	1.01E-03	↓ 73.35%
**Acetyl Propionyl**	~ 4.79E-05	4.62E-02	↓ ≥ 99.90%
**Butyraldehyde**	~ 4.58E-06	4.23E-02	↓ ≥ 99.99%

**Crotonaldehyde**	~ 7.12E-06	2.89E-02	↓ ≥ 99.98%
**Furfural**	~ 7.84E-06	1.49E-02	↓ ≥ 99.95%
**Nicotine Degradants & TSNAs**			
**β-Nicotyrine**	1.17E-04	3.50E-03	↓ 96.65%
**Cotinine**	2.75E-04	7.09E-03	↓ 96.12%
**Myosmine**	2.12E-04	6.55E-03	↓ 96.76%
**Nicotine N Oxide**	2.92E-04	~3.26E-04	NC
**Nornicotine**	7.23E-04	1.09E-02	↓ 93.40%
**Anabasine**	~ 4.92E-06	5.10E-04	↓ ≥ 99.04%
**Anatabine**	~ 3.91E-06	3.08E-03	↓ ≥ 99.87%
**NNK**	~ 1.02E-07	9.86E-05	↓ ≥ 99.90%
**NNN**	~ 1.11E-07	1.12E-04	↓ ≥ 99.90%
**Metals**			
**Arsenic**	~ 1.90E-06	NA	NA
**Beryllium**	~ 7.40E-08	NA	NA
**Cadmium**	~ 3.37E-08	4.01E-05	↓ ≥ 99.92%
**Chromium**	~ 1.90E-06	~3.95E-07	NC
**Cobalt**	~ 1.68E-08	NA	NA
**Copper**	~ 8.41E-07	1.23E-05	↓ ≥ 93.15%
**Gold**	~ 5.72E-06	NA	NA
**Iron**	~ 3.37E-06	NA	NA
**Lead**	~ 8.41E-08	1.79E-05	↓ ≥ 99.53%
**Nickel**	~ 1.68E-07	~7.89E-07	NC
**Selenium**	~ 8.04E-07	NA	NA
**Silver**	~ 3.37E-08	NA	NA
**Tin**	~ 2.02E-06	NA	NA
**Zinc**	~ 5.05E-06	NA	NA
**Organic Acids**			
**Benzoic Acid**	8.34E-01	NA	NA
**Propionic Acid**	~ 2.24E-03	NA	NA
**VOCs**			
**1,3-butadiene**	~ 1.85E-06	NA	NA
**Acrylonitrile**	~ 1.21E-06	1.27E-02	↓ ≥ 99.99%
**Benzene**	~ 1.24E-05	4.64E-02	↓ ≥ 99.97%
**Isoprene**	~ 2.85E-06	NA	NA
**Propylene Oxide**	~ 4.90E-06	9.00E-04	↓ ≥ 99.46%
**Toluene**	~ 1.50E-05	7.91E-02	↓ ≥ 99.98%
**Esters & Alcohols**			
**1-Butanol**	~ 7.26E-04	NA	NA

**Benzyl Acetate**	~ 8.47E-05	NA	NA
**Ethyl Acetate**	~ 6.75E-05	NA	NA
**Ethyl Acetoacetate**	~ 1.31E-04	NA	NA
**Isoamyl Acetate**	~ 6.82E-05	NA	NA
**Isobutyl Acetate**	~ 6.78E-05	NA	NA
**Methyl Acetate**	~ 7.03E-05	NA	NA
		Average Difference	↑ 8911.94%
		Average Difference without PG, VG, and Water	↓ 97.47%

1.9 Ruby Menthol 18 mg/mL Non-Intense Percent Difference to Combustible Cigarettes

Parameter/ Constituent	Aerosol or Smoke Yields Normalized by Nicotine (mg/mg)		% Diff vs CC
	AEROSOL MAX	CC	
**# of puffs**	135	NA	NA
**pH**	5.90	NA	NA
**Primary Constituents**			
**Nicotine**	1.00E+00	1.00E+00	NA
**Glycerol**	2.60E+01	8.95E-01	↑ 2802.08%
**Propylene Glycol**	3.50E+01	4.21E-03	↑ 830431.54%
**Water**	8.20E+00	4.21E+00	↑ 94.74%
**Menthol**	5.73E-01	~1.32E-05	↑ ≥ 4357200.89%
**Diethylene Glycol**	~ 2.02E-03	~1.42E-03	NC
**Ethylene Glycol**	~ 1.41E-03	9.96E-03	↓ ≥ 85.84%
**Carbonyls & Glycidol**			
**Acetaldehyde**	2.93E-04	7.24E-01	↓ 99.96%
**Acrolein**	~ 4.04E-05	5.96E-02	↓ ≥ 99.93%
**Diacetyl**	~ 3.93E-05	1.64E-01	↓ ≥ 99.98%
**Formaldehyde**	8.18E-04	3.74E-02	↓ 97.82%
**Glycidol**	1.84E-04	1.04E-03	↓ 82.21%
**Acetyl Propionyl**	~ 4.71E-06	4.21E-02	↓ ≥ 99.99%
**Butyraldehyde**	~ 3.78E-06	3.33E-02	↓ ≥ 99.99%
**Crotonaldehyde**	~ 5.71E-06	1.53E-02	↓ ≥ 99.96%
**Furfural**	~ 6.46E-06	1.84E-02	↓ ≥ 99.96%
**Nicotine Degradants & TSNAs**			
**β-Nicotyrine**	2.07E-04	1.07E-02	↓ 98.05%
**Cotinine**	2.42E-04	6.96E-03	↓ 96.52%
**Myosmine**	3.01E-04	NA	NA
**Nicotine N Oxide**	2.56E-04	~5.37E-04	NC
**Nornicotine**	6.68E-04	9.50E-03	↓ 92.97%

**Anabasine**	~ 4.05E-06	8.33E-04	↓ ≥ 99.51%
**Anatabine**	~ 3.22E-06	6.00E-03	↓ ≥ 99.95%
**NNK**	~ 9.52E-08	9.85E-05	↓ ≥ 99.90%
**NNN**	3.53E-07	1.18E-04	↓ 99.70%
**Metals**			
**Arsenic**	~ 3.15E-07	NA	NA
**Beryllium**	~ 6.93E-08	NA	NA
**Cadmium**	~ 3.15E-08	3.35E-05	↓ ≥ 99.91%
**Chromium**	~ 3.15E-07	~6.27E-07	NC
**Cobalt**	~ 1.57E-08	NA	NA
**Copper**	~ 7.87E-07	1.64E-05	↓ ≥ 95.19%
**Gold**	~ 5.35E-06	NA	NA
**Iron**	~ 3.15E-06	NA	NA
**Lead**	~ 7.87E-08	~4.18E-07	NC
**Nickel**	~ 1.57E-07	~9.64E-07	NC
**Selenium**	~ 7.52E-07	NA	NA
**Silver**	~ 1.67E-07	NA	NA
**Tin**	~ 3.15E-07	NA	NA
**Zinc**	~ 4.72E-06	NA	NA
**Organic Acids**			
**Benzoic Acid**	7.19E-01	NA	NA
**Propionic Acid**	~ 2.15E-03	NA	NA
**VOCs**			
**1,3-butadiene**	~ 2.20E-06	NA	NA
**Acrylonitrile**	~ 1.09E-06	9.71E-03	↓ ≥ 99.99%
**Benzene**	~ 1.15E-05	4.58E-02	↓ ≥ 99.97%
**Isoprene**	~ 2.55E-06	NA	NA
**Propylene Oxide**	~ 7.17E-06	8.44E-01	↓ ≥ 99.99%
**Toluene**	~ 1.35E-05	7.35E-02	↓ ≥ 99.98%
**Esters & Alcohols**			
**1-Butanol**	~ 6.02E-05	NA	NA
**Benzyl Acetate**	~ 7.92E-05	NA	NA
**Ethyl Acetate**	~ 6.31E-05	NA	NA
**Ethyl Acetoacetate**	~ 1.23E-04	NA	NA
**Isoamyl Acetate**	~ 6.38E-05	NA	NA
**Isobutyl Acetate**	~ 6.35E-05	NA	NA
**Methyl Acetate**	~ 7.29E-04	NA	NA
		Average Difference	↑ 192158.59%
		Average Difference without PG, VG, Water, and Menthol	↓ 97.71%

1.10 Ruby Menthol 18 mg/mL Intense Percent Difference to Combustible Cigarettes

Parameter/ Constituent	Aerosol or Smoke Yields Normalized by Nicotine (mg/mg)		% Diff vs CC
	AEROSOL MAX	CC	
**# of puffs**	90	NA	NA
**pH**	6.09	NA	NA
**Primary Constituents**			
**Nicotine**	1.00E+00	1.00E+00	NA
**Glycerol**	2.62E+01	1.20E+00	↑ 2092.98%
**Propylene Glycol**	3.50E+01	1.36E-02	↑ 257107.51%
**Water**	6.18E+00	7.94E+00	↓ 22.10%
**Menthol**	5.58E-01	~4.76E-06	↑ ≥ 11720092.34%
**Diethylene Glycol**	~ 2.09E-03	~9.90E-04	NC
**Ethylene Glycol**	~ 1.46E-03	1.73E-02	↓ ≥ 91.60%
**Carbonyls & Glycidol**			
**Acetaldehyde**	3.62E-04	8.19E-01	↓ 99.96%
**Acrolein**	1.25E-04	8.12E-02	↓ 99.85%
**Diacetyl**	~ 5.03E-05	1.69E-01	↓ ≥ 99.97%
**Formaldehyde**	9.31E-04	5.47E-02	↓ 98.30%
**Glycidol**	3.21E-04	1.01E-03	↓ 68.28%
**Acetyl Propionyl**	~ 5.67E-06	4.62E-02	↓ ≥ 99.99%
**Butyraldehyde**	~ 4.55E-06	4.23E-02	↓ ≥ 99.99%
**Crotonaldehyde**	~ 7.24E-06	2.89E-02	↓ ≥ 99.97%
**Furfural**	~ 7.78E-06	1.49E-02	↓ ≥ 99.95%
**Nicotine Degradants & TSNAs**			
**β-Nicotyrine**	2.94E-04	3.50E-03	↓ 91.59%
**Cotinine**	2.65E-04	7.09E-03	↓ 96.27%
**Myosmine**	3.21E-04	6.55E-03	↓ 95.09%
**Nicotine N Oxide**	2.35E-04	~3.26E-04	NC
**Nornicotine**	8.86E-04	1.09E-02	↓ 91.91%
**Anabasine**	~ 4.88E-06	5.10E-04	↓ ≥ 99.04%
**Anatabine**	~ 3.88E-06	3.08E-03	↓ ≥ 99.87%
**NNK**	~ 1.03E-07	9.86E-05	↓ ≥ 99.90%
**NNN**	4.46E-07	1.12E-04	↓ 99.60%
**Metals**			
**Arsenic**	~ 3.42E-07	NA	NA
**Beryllium**	~ 7.53E-08	NA	NA
**Cadmium**	~ 3.42E-08	4.01E-05	↓ ≥ 99.91%
**Chromium**	~ 3.42E-07	~3.95E-07	NC

**Cobalt**	~ 1.71E-08	NA	NA
**Copper**	~ 8.56E-07	1.23E-05	↓ ≥ 93.03%
**Gold**	~ 5.82E-06	NA	NA
**Iron**	~ 3.42E-06	NA	NA
**Lead**	~ 4.17E-07	1.79E-05	↓ ≥ 97.68%
**Nickel**	~ 1.71E-07	~7.89E-07	NC
**Selenium**	~ 8.18E-07	NA	NA
**Silver**	~ 1.79E-07	NA	NA
**Tin**	~ 2.00E-06	NA	NA
**Zinc**	~ 5.13E-06	NA	NA
**Organic Acids**			
**Benzoic Acid**	8.02E-01	NA	NA
**Propionic Acid**	~ 2.22E-03	NA	NA
**VOCs**			
**1,3-butadiene**	~ 1.88E-06	NA	NA
**Acrylonitrile**	~ 1.23E-06	1.27E-02	↓ ≥ 99.99%
**Benzene**	~ 1.27E-05	4.64E-02	↓ ≥ 99.97%
**Isoprene**	~ 2.89E-06	NA	NA
**Propylene Oxide**	~ 4.98E-06	9.00E-04	↓ ≥ 99.45%
**Toluene**	~ 1.53E-05	7.91E-02	↓ ≥ 99.98%
**Esters & Alcohols**			
**1-Butanol**	~ 6.55E-05	NA	NA
**Benzyl Acetate**	~ 8.62E-05	NA	NA
**Ethyl Acetate**	~ 6.86E-05	NA	NA
**Ethyl Acetoacetate**	~ 1.34E-04	NA	NA
**Isoamyl Acetate**	~ 6.93E-05	NA	NA
**Isobutyl Acetate**	~ 6.90E-05	NA	NA
**Methyl Acetate**	~ 8.02E-04	NA	NA
		Average Difference	↑ 412994.81%
		Average Difference without PG, VG, Water, and Menthol	↓ 96.85%

1.11 Summer Menthol 18 mg/mL Non-Intense Percent Difference to Combustible Cigarettes

**Parameter/ Constituent**	**Aerosol or Smoke Yields Normalized by Nicotine (mg/mg)**		**% Diff vs CC**
	**AEROSOL MAX**	**CC**	
**# of puffs**	138	NA	NA
**pH**	6.46	NA	NA
**Primary Constituents**			
**Nicotine**	1.00E+00	1.00E+00	NA

**Glycerol**	2.57E+01	8.95E-01	↑ 2777.39%
**Propylene Glycol**	3.34E+01	4.21E-03	↑ 793277.57%
**Water**	8.38E+00	4.21E+00	↑ 99.11%
**Menthol**	5.65E-01	~1.32E-05	↑ ≥ 4297635.85%
**Diethylene Glycol**	~ 2.09E-03	~1.42E-03	NC
**Ethylene Glycol**	~ 1.46E-03	9.96E-03	↓ ≥ 85.35%
**Carbonyls & Glycidol**			
**Acetaldehyde**	1.12E-04	7.24E-01	↓ 99.98%
**Acrolein**	~ 4.02E-05	5.96E-02	↓ ≥ 99.93%
**Diacetyl**	~ 4.06E-05	1.64E-01	↓ ≥ 99.98%
**Formaldehyde**	5.38E-04	3.74E-02	↓ 98.56%
**Glycidol**	1.17E-04	1.04E-03	↓ 88.75%
**Acetyl Propionyl**	~ 3.30E-05	4.21E-02	↓ ≥ 99.92%
**Butyraldehyde**	~ 3.91E-06	3.33E-02	↓ ≥ 99.99%
**Crotonaldehyde**	~ 5.62E-06	1.53E-02	↓ ≥ 99.96%
**Furfural**	~ 6.68E-06	1.84E-02	↓ ≥ 99.96%
**Nicotine Degradants & TSNAs**			
**β-Nicotyrine**	7.87E-05	1.07E-02	↓ 99.26%
**Cotinine**	1.18E-04	6.96E-03	↓ 98.31%
**Myosmine**	1.13E-04	NA	NA
**Nicotine N Oxide**	1.86E-04	~5.37E-04	NC
**Nornicotine**	4.54E-04	9.50E-03	↓ 95.22%
**Anabasine**	~ 4.19E-06	8.33E-04	↓ ≥ 99.50%
**Anatabine**	~ 3.33E-06	6.00E-03	↓ ≥ 99.94%
**NNK**	~ 9.37E-08	9.85E-05	↓ ≥ 99.90%
**NNN**	~ 2.52E-08	1.18E-04	↓ ≥ 99.98%
**Metals**			
**Arsenic**	~ 3.16E-07	NA	NA
**Beryllium**	~ 6.95E-08	NA	NA
**Cadmium**	~ 3.16E-08	3.35E-05	↓ ≥ 99.91%
**Chromium**	~ 3.16E-07	~6.27E-07	NC
**Cobalt**	~ 1.58E-08	NA	NA
**Copper**	~ 7.89E-07	1.64E-05	↓ ≥ 95.17%
**Gold**	~ 5.37E-06	NA	NA
**Iron**	~ 3.16E-06	NA	NA
**Lead**	~ 7.89E-08	~4.18E-07	NC
**Nickel**	~ 1.58E-07	~9.64E-07	NC
**Selenium**	~ 7.41E-07	NA	NA
**Silver**	~ 1.61E-07	NA	NA
**Tin**	~ 3.16E-07	NA	NA

**Zinc**	~ 1.49E-05	NA	NA
**Organic Acids**			
**Benzoic Acid**	6.87E-01	NA	NA
**Propionic Acid**	~ 2.23E-03	NA	NA
**VOCs**			
**1,3-butadiene**	~ 2.17E-06	NA	NA
**Acrylonitrile**	~ 1.07E-06	9.71E-03	↓ ≥ 99.99%
**Benzene**	~ 1.13E-05	4.58E-02	↓ ≥ 99.98%
**Isoprene**	~ 2.52E-06	NA	NA
**Propylene Oxide**	~ 7.06E-06	8.44E-01	↓ ≥ 99.99%
**Toluene**	~ 1.33E-05	7.35E-02	↓ ≥ 99.98%
**Esters & Alcohols**			
**1-Butanol**	~ 5.93E-05	NA	NA
**Benzyl Acetate**	~ 7.80E-05	NA	NA
**Ethyl Acetate**	1.02E-02	NA	NA
**Ethyl Acetoacetate**	~ 1.21E-04	NA	NA
**Isoamyl Acetate**	~ 6.28E-05	NA	NA
**Isobutyl Acetate**	~ 6.25E-05	NA	NA
**Methyl Acetate**	~ 7.18E-04	NA	NA
		Average Difference	↑ 188575.20%
		Average Difference without PG, VG, Water, and Menthol	↓ 98.24%

1.12 Summer Menthol 18 mg/mL Intense Percent Difference to Combustible Cigarettes

Parameter/ Constituent	Aerosol or Smoke Yields Normalized by Nicotine (mg/mg)		% Diff vs CC
	AEROSOL MAX	CC	
**# of puffs**	86	NA	NA
**pH**	6.51	NA	NA
**Primary Constituents**			
**Nicotine**	1.00E+00	1.00E+00	NA
**Glycerol**	2.59E+01	1.20E+00	↑ 2062.53%
**Propylene Glycol**	3.36E+01	1.36E-02	↑ 246305.78%
**Water**	6.47E+00	7.94E+00	↓ 18.53%
**Menthol**	5.60E-01	~4.76E-06	↑ ≥ 11751564.85%
**Diethylene Glycol**	~ 2.19E-03	~9.90E-04	NC
**Ethylene Glycol**	~ 1.52E-03	1.73E-02	↓ ≥ 91.20%
**Carbonyls & Glycidol**			
**Acetaldehyde**	1.20E-04	8.19E-01	↓ 99.99%
**Acrolein**	1.24E-04	8.12E-02	↓ 99.85%

**Diacetyl**	~ 4.95E-05	1.69E-01	↓ ≥ 99.97%
**Formaldehyde**	6.56E-04	5.47E-02	↓ 98.80%
**Glycidol**	2.62E-04	1.01E-03	↓ 74.15%
**Acetyl Propionyl**	~ 5.94E-06	4.62E-02	↓ ≥ 99.99%
**Butyraldehyde**	~ 4.76E-06	4.23E-02	↓ ≥ 99.99%
**Crotonaldehyde**	~ 6.31E-06	2.89E-02	↓ ≥ 99.98%
**Furfural**	~ 8.15E-06	1.49E-02	↓ ≥ 99.95%
**Nicotine Degradants & TSNAs**			
**β-Nicotyrine**	7.81E-05	3.50E-03	↓ 97.77%
**Cotinine**	1.23E-04	7.09E-03	↓ 98.26%
**Myosmine**	1.08E-04	6.55E-03	↓ 98.35%
**Nicotine N Oxide**	1.72E-04	~3.26E-04	NC
**Nornicotine**	4.49E-04	1.09E-02	↓ 95.90%
**Anabasine**	~ 5.11E-06	5.10E-04	↓ ≥ 99.00%
**Anatabine**	~ 4.06E-06	3.08E-03	↓ ≥ 99.87%
**NNK**	~ 9.03E-08	9.86E-05	↓ ≥ 99.91%
**NNN**	~ 2.43E-08	1.12E-04	↓ ≥ 99.98%
**Metals**			
**Arsenic**	~ 3.30E-07	NA	NA
**Beryllium**	~ 7.26E-08	NA	NA
**Cadmium**	~ 3.30E-08	4.01E-05	↓ ≥ 99.92%
**Chromium**	~ 3.30E-07	~3.95E-07	NC
**Cobalt**	~ 1.65E-08	NA	NA
**Copper**	~ 8.25E-07	1.23E-05	↓ ≥ 93.28%
**Gold**	~ 5.61E-06	NA	NA
**Iron**	~ 3.30E-06	NA	NA
**Lead**	~ 8.25E-08	1.79E-05	↓ ≥ 99.54%
**Nickel**	~ 1.65E-07	~7.89E-07	NC
**Selenium**	~ 7.89E-07	NA	NA
**Silver**	~ 1.72E-07	NA	NA
**Tin**	~ 3.30E-07	NA	NA
**Zinc**	~ 4.95E-06	NA	NA
**Organic Acids**			
**Benzoic Acid**	7.84E-01	NA	NA
**Propionic Acid**	~ 2.33E-03	NA	NA
**VOCs**			
**1,3-butadiene**	~ 1.64E-06	NA	NA
**Acrylonitrile**	~ 1.07E-06	1.27E-02	↓ ≥ 99.99%
**Benzene**	~ 1.11E-05	4.64E-02	↓ ≥ 99.98%
**Isoprene**	~ 2.52E-06	NA	NA

**Propylene Oxide**	~ 4.35E-06	9.00E-04	↓ ≥ 99.52%
**Toluene**	~ 1.34E-05	7.91E-02	↓ ≥ 99.98%
**Esters & Alcohols**			
**1-Butanol**	~ 5.71E-05	NA	NA
**Benzyl Acetate**	~ 7.52E-05	NA	NA
**Ethyl Acetate**	1.07E-02	NA	NA
**Ethyl Acetoacetate**	~ 1.17E-04	NA	NA
**Isoamyl Acetate**	~ 6.05E-05	NA	NA
**Isobutyl Acetate**	~ 6.02E-05	NA	NA
**Methyl Acetate**	~ 7.14E-04	NA	NA
Average Difference			↑ 413705.85%
Average Difference without PG, VG, Water, and Menthol			↓ 97.80%

1.13 Average Non-Intense and Intense Percent Difference to Combustible Cigarettes

Parameter/ Constituent	Aerosol or Smoke Yields Normalized by Nicotine (mg/mg)			
	MAX Non-Intense Average % Difference vs CC	Range of Non-Intense % Difference vs CC Results	MAX Intense Average % Difference vs CC	Range of Intense % Difference vs CC Results
**# of puffs**	NA	NA	NA	
**pH**	NA	NA	NA	
**Primary Constituents**				
**Nicotine**	NA	NA	NA	NA
**Glycerol**	↑ 3164%	↑ 2777% - ↑ 4763%	↑ 2327%	↑ 2063% - ↑ 3376%
**Propylene Glycol**	↑ 832447%	↑ 593470% - ↑ 1067676%	↑ 251844%	↑185338% - ↑296295%
**Water**	↑ 51%	↑ 10.57% - ↑ 99.11%	↓ 33.73%	↓ 18.53% - ↓ 46.98%
**Menthol***	↑ ≥ 5065342%	↑ ≥ 4297636% - ↑ ≥ 5991319%	13696514%	↑ 11720092% - ↑ 16168586%
**Diethylene Glycol**	NC	NC	NC	NC
**Ethylene Glycol**	↓ ≥ 64%	↓ ≥40.53% - ↓ ≥ 87.11%	↓ ≥ 81%	↓ ≥ 69.09% - ↓ ≥ 91.6%
**Carbonyls & Glycidol**				
**Acetaldehyde**	↓ 99.97%	↓ 99.96% - ↓ 99.98%	↓ 99.97%	↓ 99.95% - ↓ 99.99%
**Acrolein**	↓ 99.84%	↓ 99.7% - ↓ 99.93%	↓ 99.74%	↓ 99.5% - ↓ 99.85%
**Diacetyl**	↓ ≥ 99.98%	↓ ≥ 99.98 - ↓ ≥ 99.99	↓ ≥ 99.97%	↓ 99.97% - ↓ 99.97%
**Formaldehyde**	↓ 97.94%	↓ 98.56% - ↓ 96.98%	↓ 98.27%	↓ 98.29% - ↓ 97.8%
**Glycidol**	↓ 83.41%	↓ 76.91% - ↓ 88.75%	↓ 72.41%	↓ 66.69% - ↓ 79.64%
**Acetyl Propionyl**	↓ ≥ 99.98%	↓ ≥ 99.92% - ↓ ≥ 99.99%	↓ ≥ 99.98%	↓ ≥ 99.9% - ↓ ≥ 99.99%
**Butyraldehyde**	↓ ≥ 99.97%	↓ ≥ 99.89% - ↓ ≥ 99.99%	↓ ≥ 99.97%	↓ ≥ 99.91% - ↓ ≥ 99.99%
**Crotonaldehyde**	↓ ≥ 99.96%	↓ ≥99.96% - ↓ ≥ 99.96%	↓ ≥99.97%	↓ ≥ 99.96% - ↓ ≥ 99.98%
**Furfural**	↓ ≥ 99.97%	↓ ≥ 99.96%- ↓ ≥ 99.97%	↓ ≥ 99.91%	↓ ≥ 99.71% - ↓ ≥ 99.95%
**Nicotine Degradants & TSNAs**				

**β-Nicotyrine**	↓ 98.29%	↓ 97.11% - ↓ 99.26%	↓ 94.24%	↓ 90.35% - ↓ 97.77%
**Cotinine**	↓ 92.46%	↓ 65.35% - ↓ 99.56%	↓ 92.05%	↓ 64.14% - ↓ 99.14%
**Myosmine**	NA	NA	↓ 92.10%	↓ 69.8% - ↓ 98.35%
**Nicotine N Oxide**	NC	NC	NC	NC
**Nornicotine**	↓ 92.41%	↓ 90.15% - ↓ 95.22%	↓ 93.30%	↓ 91.91% - ↓ 95.9%
**Anabasine**	↓ ≥ 99.59%	↓ ≥ 99.5% - ↓ ≥ 99.66%	↓ ≥ 98.34%	↓ ≥ 94.15% - ↓ ≥ 99.42%
**Anatabine**	↓ ≥ 99.8%	↓ ≥ 99.45% - ↓ ≥ 99.95%	↓ ≥ 99.56%	↓ ≥ 99.9% - ↓ ≥ 98.93%
**NNK**	↓ ≥ 99.55%	↓ ≥ 99.13% - ↓ ≥ 99.91%	↓ ≥ 99.56%	↓ ≥ 99.21% - ↓ ≥ 99.91%
**NNN**	↓ ≥ 99.90%	↓ ≥ 99.70% - ↓ ≥ 99.98%	↓ ≥ 99.87%	↓ ≥ 99.6% - ↓ ≥ 99.98%
**Metals**				
**Arsenic**	NA	NA	NA	NA
**Beryllium**	NA	NA	NA	NA
**Cadmium**	↓ ≥ 99.91%	↓ ≥ 99.9% - ↓ ≥ 99.91%	↓ ≥ 99.92%	↓ ≥ 99.91% - ↓ ≥ 99.93%
**Chromium**	NC	NC	NC	NC
**Cobalt**	NA	NA	NA	NA
**Copper**	↓ ≥ 88.59%	↓ ≥ 79.9% - ↓ ≥ 95.36%	↓ ≥ 93.65%	↓ ≥ 93.03% -↓ ≥94.3%
**Gold**	NA	NA	NA	NA
**Iron**	NA	NA	NA	NA
**Lead**	NC	NC	↓ ≥ 99.26%	↓ ≥ 97.68% - ↓ ≥ 99.61%
**Nickel**	NC	NC	NC	NC
**Selenium**	NA	NA	NA	NA
**Silver**	NA	NA	NA	NA
**Tin**	NA	NA	NA	NA
**Zinc**	NA	NA	NA	NA
**Organic Acids**				
**Benzoic Acid**	NA	NA	NA	NA
**Propionic Acid**	NA	NA	NA	NA
**VOCs**				

**1,3-butadiene**	NA	NA	NA	NA
**Acrylonitrile**	↓ ≥ 99.99%	↓ ≥ 99.99% - ↓ ≥ 99.99%	↓ ≥ 99.99%	↓ ≥ 99.99% - ↓ ≥ 99.99%
**Benzene**	↓ ≥ 99.98%	↓ ≥ 99.97% - ↓ ≥ 99.99%	↓ ≥ 99.98%	↓ ≥ 99.97% - ↓ ≥ 99.99%
**Isoprene**	NA	NA	NA	NA
**Propylene Oxide**	↓ ≥ 99.99%	↓ ≥ 99.99% -↓ ≥99.99%	↓ ≥ 99.51%	↓ ≥ 99.45% - ↓ ≥ 99.55%
**Toluene**	↓ ≥ 99.98%	↓ ≥ 99.98% - ↓ ≥ 99.99%	↓ ≥ 99.98%	↓ ≥ 99.98% - ↓ ≥ 99.99%
**Esters & Alcohols**				
**1-Butanol**	NA	NA	NA	NA
**Benzyl Acetate**	NA	NA	NA	NA
**Ethyl Acetate**	NA	NA	NA	NA
**Ethyl Acetoacetate**	NA	NA	NA	NA
**Isoamyl Acetate**	NA	NA	NA	NA
**Isobutyl Acetate**	NA	NA	NA	NA
**Methyl Acetate**	NA	NA	NA	NA
Average Max % Difference	218475%		480974%	
Average Max % Difference without PG, VG, and Water	96.34%		96.50%	
Non-Intense and Intense Average Max % Difference without PG, VG, and Water	96.42%			

1.14 Statistical Comparison of Non-Intense 1R6F COA Values to Quantifiable Non-Intense JUUL2 Virginia Tobacco Constituents

Parameter/ Constituent	Certified 1R6F Value	Certified 1R6F Uncertainty	Nicotine Normalized Certified Value	Nicotine Normalized Certified Uncertainty	Maximium T0-T12 VT Mean Value	SD	Nicotine Normalized Mean Value	Nicotine Normalized SD	P Value
	1R6F ISO 3308:2012				JUUL2 Virginia Tobacco ISO 20768:2018				
	mg/cigarette		mg/mg nicotine		mg/collection		mg/mg nicotine		
Nicotine	7.21E-01	1.03E-01	NA	NA	1.58E+01	4.30E-01	NA	NA	NA
Acetaldehyde	5.22E-01	7.10E-02	7.24E-01	9.85E-02	4.32E-03	3.60E-04	2.74E-04	2.28E-05	0.0001
Acrolein	4.30E-02	1.40E-02	5.96E-02	1.94E-02	2.81E-03	2.70E-04	1.78E-04	1.71E-05	0.0001
Formaldehyde	2.70E-02	1.00E-02	3.74E-02	1.39E-02	1.79E-02	3.30E-04	1.13E-03	2.09E-05	0.002

1.15 Statistical Comparison of Intense 1R6F COA Values to Quantifiable Intense JUUL2 Virginia Tobacco Constituents

Parameter/ Constituent	Certified 1R6F Value	Certified 1R6F Uncertainty	Nicotine Normalized Certified Value	Nicotine Normalized Certified Uncertainty	Maximium T0-T12 VT Mean Value	SD	Nicotine Normalized Mean Value	Nicotine Normalized SD	P Value
	1R6F ISO 20778:2018				JUUL2 Virginia Tobacco Intense				
	mg/cigarette		mg/mg nicotine		mg/collection		mg/mg nicotine		
Nicotine	1.90E+00	1.30E-01	NA	NA	1.68E+01	2.66E+00	NA	NA	NA
Acetaldehyde	1.55E+00	2.39E-01	8.19E-01	1.26E-01	6.66E-03	5.10E-04	3.96E-04	3.03E-05	0.0001
Acrolein	1.54E-01	3.30E-02	8.12E-02	1.74E-02	6.82E-03	5.80E-04	4.05E-04	3.45E-05	0.0001
Formaldehyde	1.04E-01	3.40E-02	5.49E-02	1.79E-02	2.50E-02	1.13E-03	1.48E-03	6.72E-05	0.0001
