# Supplementary material for: A Practical Framework for Novel Electronic Nicotine Delivery System Evaluation: Chemical and Toxicological Characterization of JUUL2 Aerosol and Comparison with Reference Cigarettes
Source: Toxics. 2024 Jan 4;12(1):41. doi: 10.3390/toxics12010041 (PMC10820849; doi:10.3390/toxics12010041)
Supplement: Supplementary file 1 [file toxics-12-00041-s001.zip › Supplementary_Material_File S6_Analysis of Toxicity Studies and Method Details_Final_Clean.pdf]

Supplementary Material File S6

1 In Vitro Methodologies for Toxicological Characterization of Aerosol

NRU cytotoxicity and Ames mutagenicity assays were performed as previously described (Lalonde et al. 2022).

1.1 Cell Culture

Human adenocarcinoma A549 cells and Mouse embryonic fibroblast BALBc/3T3 cells used in this assay were obtained from ATCC (Manassas, VA). Cells were propagated at Enthalpy Analytical, and subcultures stored in liquid nitrogen. The cells were tested for the absence of mycoplasma contamination and were not used beyond the 16th passage after removal from liquid nitrogen. A549 cells were cultured in complete F-12K medium supplemented with 10% Fetal bovine serum (FBS), penicillin G (100 units/mL) and streptomycin (100 µg/mL), as recommended by the manufacturer. BALBc/3T3 cells were grown in DMEM (Dulbecco's Modified Eagles medium) supplemented with non-heat-inactivated 10% bovine calf serum (BCS), penicillin G (100 units/mL) and streptomycin (100 µg/mL). Cultures were grown in a humidified incubator at 37 ± 1 °C in an atmosphere of 5 ± 1% CO^2 in air.

The *Salmonella typhimurium* tester strains TA1537, TA98, TA100, TA1535 were originally purchased from Molecular Toxicology, Inc. (Boone, NC). The *S. typhimurium* strain TA102 was originally obtained from Pfizer Global Research and Development (Groton, CT). Isolates of the tester strains were stored at -60°C to -80°C until use. For each experiment, an inoculum from an isolate culture was grown in Oxoid nutrient broth with shaking (5-12 hours) at approximately 37°C ± 1°C. Bacterial cell titers were determined for these assays, as per the applicable SOP.

TK6 cells, a human lymphoblast cell line were originally obtained from Pfizer Global Research and Development (Groton, CT) and subsequently subcloned at Charles River. Cell passage number was limited to 24 passages. Stock cultures were maintained in RPMI 1640 + L-glutamine supplemented with 10% heat inactivated fetal bovine serum (FBS) and 1% penicillin-streptomycin (Complete Culture Medium, CCM). Cultures were incubated at 36°C to 38°C and 4% to 6% CO_2.

1.2 S9 Metabolic Activation System

The 9000 × g liver supernatant fraction (S9) from phenobarbital/5,6-benzoflavone-treated male rats, glucose-6-phosphate and $MgCl_2$-KCl in 0.1M phosphate buffer (Regensys "A"), and lyophilized nicotinamide adenine dinucleotide phosphate (NADP; Regensys "B") were obtained from Molecular Toxicology, Inc. (Boone, NC). These reagents were combined to create the metabolic activation mixture (S9), maintained at 2°C to 8°C or on ice prior to use, on the day of testing.

Regensys "A" and Regensys "B" were received in pre-measured aliquots and stored refrigerated (2°C to 8°C) and frozen (below -20°C), respectively. Prior to use, the appropriate aliquot(s) of Regensys "B" was removed from storage and maintained at ambient temperature. Regensys "A" was maintained at 2°C to 8°C or on ice. Regensys "A" was used to reconstitute an appropriate aliquot of Regensys "B". The Regensys A and B solution was mixed prior to formulating the S9 mixture (7.5% v/v S9).

1.3 Neutral Red Uptake (NRU) Cytotoxicity (OECD guidance 129)

The test articles for the NRU assay were aerosol condensates from the relevant sample. Cell cultures were initiated by seeding approximately 10,000 (A549) or 3,000 (BALBc/3T3) exponentially growing cells in 0.2 mL (A549) or 0.1 mL (BALBc/3T3) cell culture medium per microwell of 96 microwell plates. Cultures were incubated overnight (24 ± 2 hrs.) in a humidified incubator at 37 ± 1° C in an atmosphere of 5 ± 1% CO^2 in air prior to use in the NRU assay. One day after culture initiation (24 ± 2 hrs.), the medium was aspirated and replaced with fresh dosing medium containing vehicle control (USP ethanol), positive control (SLS), or the test article and then returned to the incubator at 37 ± 1°C and 5 ± 1% CO^2 for an exposure period of ~48 hours.

The eight non-zero concentrations of aerosol condensate in ethanol were 20, 30, 45, 65, 95, 140, 205, and 300 µg/mL (approximately 0.033, 0.050, 0.075, 0.109, 0.159, 0.234, 0.344, 0.500%)(v/v). The final concentration of EtOH in the dosing media was normalized and did not exceed 0.5% per OECD standards (see Table 1). The eight non-zero concentrations of SLS were 6.8, 10.0, 14.7, 21.5, 31.6, 46.4, 68.1, and 100 µg/mL. All tests were performed in six replicates. Osmolarity and pH measurements will be performed on the two highest doses of the e-vapor ethanol condensate tested. Osmolarity and pH was also measured for the tissue culture medium, tissue culture medium+vehicle control, and tissue culture medium+test sample.

Table S1 Neutral Red Uptake Dosing

Dose number	Dose (µg/mL)	Sample (µL)	Ethanol (µL)	Media (µL)	% test article v/v
**1**	300	5	0	995	0.503
**2**	205	3.42	1.58	995	0.344
**3**	140	2.33	2.67	995	0.234
**4**	95	1.58	3.42	995	0.159
**5**	65	1.08	3.92	995	0.109
**6**	45	0.75	4.25	995	0.075
**7**	30	0.5	4.5	995	0.050
**8**	20	0.33	4.67	995	0.033
**VC**	VC	0	5	995	0.000

Plates were visually inspected for precipitation and/or changes in morphology of the cells due to cytotoxic effects after 48 hours. Medium was removed from the wells by aspiration and the cells were rinsed with 250 µL/well pre-warmed PBS. The rinsing solution was removed by plate inversion. Then 250 µL of a “crystal free” neutral red dye solution (25 ug/mL NR Dye solution in complete growth medium; crystals removed by centrifugation at 1500 rpm for ≥8 min) was added to each well. Plates were subsequently incubated for 3 ± 0.25 hrs. After incubation, NR medium was removed, cells were rinsed with 250 µL/well pre-warmed PBS, and 100 µL NR desorb solution (freshly prepared 50% ethanol and 1% acetic acid in water) was added to all wells (including blanks) to extract the dye. The plates were then shaken rapidly on a microtiter plate shaker for 20-45 min while protected from light.

Optical density [OD] was measured at 540 ±10 nm (OD540) using a spectrophotometer (SYNERGY MX™ MULTI-MODE MICROPLATE READER (MSPEC-01) with the SkanIt DDE for MSS 2.4.4 Software) within 60 minutes of adding NR desorb solution. Blanks of each

plate were subtracted and the relative cell survival (% of vehicle control) of each culture exposed to the individual doses of the test articles was calculated (Equation 1). Hill function analysis of cell viability for each concentration to calculate the half maximal inhibitor concentration (IC_{50}) for the positive control and test articles was performed. The average, standard deviation and relative standard deviation was calculated for the uncorrected (raw) absorbance, the blank corrected absorbance, and the normalized (relative to the vehicle control) absorbance of each replicate value for each dose of the positive controls and the test articles.

Equation 1: % Relative viability = (NRU-Blank corrected absorbance of test article / NRU blank corrected absorbance for Vehicle control) *100

1.4 Ames Reverse Mutation Assay (OECD guidance 471)

The test articles for the Ames assay were aerosol condensates from the relevant sample. A preincubation procedure was performed using the appropriate positive controls (see Table 2), vehicle control (200 proof ethanol), and at least 5 test article concentrations in triplicate with and without metabolic activation (±S9). Controls were shared between the test articles if they were dosed concurrently. Test article was diluted in 200 proof ethanol to form the following test concentrations: 0.781, 1.56, 3.13, 6.25, 12.5, 25.0, 50.0, and 100 µL/plate.

Table S2 Ames Treatment Groups

Treatment Groups	Concentration/ Plate	Metabolic Activation	Strains[a]
1. Vehicle Control	≤ 200 µL	-/+	All
2. Concentrations of test article	Specified in protocol[b]	-/+	All
3. Positive Control ICR 191 acridine	0.5 µg	–	TA1537
4. Positive Control 2 Nitrofluorene	2.5 µg	–	TA98
5. Positive Control Sodium azide	1.0 µg	–	TA100, TA1535
6. Positive Control 4-Nitroquinoline-N-oxide	2.0 µg	–	TA102
7. Positive Control 2-Aminoanthracene	10.0 µg	+	TA102
8. Positive Control 2-Aminoanthracene	2.5 µg	+	All except TA102

Pre-incubation was performed by adding 0.10 mL of the vehicle control or test article, or 0.05 mL of the positive control; 0.5 mL of S9 mixture for plates with metabolic activation or 0.5 mL of PBS for plates without metabolic activation; and 0.1 mL of tester strain to sterile tubes and incubated with shaking (100-150 rpm) at 36°C to 38°C for 20 minutes. After pre-incubation, tubes were removed from the shaker and 2 mL of molten top agar, supplemented with 10% of a 0.5 mM histidine/biotin/tryptophan solution, was added and vortexed gently. The complete mixture was then poured onto minimal glucose agar plates and allowed to set. Plates were incubated at 36°C to 38°C for approximately 2 days.

After 2 days incubation period, plates were evaluated macroscopically for precipitation of test article and microscopically for thinning of the background lawn. The number of revertant colonies were counted by hand or with an automatic colony counter and recorded. Plates that had a cytotoxic reduction in background lawn growth (> 50% reduction in the background lawn)

were not counted. A minimum of five non-cytotoxic concentrations were required to evaluate an assay. Concentrations of > 50% reduction in mean number of revertants/plate accompanied by a concentration-dependent drop in mean revertants, or a > 50% reduction in background lawn were considered cytotoxic. Positive results for mutagenicity were considered an increase of revertants/plate at least 2 times the vehicle control background frequency for strains with high spontaneous levels (i.e., TA100 and TA102) and 3 times for those with low spontaneous levels (TA1537, TA98, and TA1535) with increasing concentration.

1.5 Micronucleus (MN) Assay (OECD guidance 487)

The in vitro micronucleus assay was performed in human lymphoblast TK6 cells, according to OECD Test Guidance 487. In brief, on the day prior to treatment, cells were sub-cultured and exponentially growing cultures of TK6 cells were treated with the vehicle control, positive controls or test article (aerosol condensate) in the presence and absence of metabolic activation for short incubations (4 hours) and in the absence of activation for the long incubation (27 hours). The metabolic activation mixture, consisting of 0.3 mL of S9 fraction, 1.5 mL of dilution medium, and 36.5 mg nicotinamide adenine dinucleotide phosphate reduced form was prepared daily. Positive controls for each of the incubation conditions were, mitomycin C, vinblastine sulfate and cyclophosphamide for the 4 hour without S9, 27 Hour without S9 and 4 hour with S9, respectively.

During the short exposures, cells were treated with the test articles for 4 hours either with or without metabolic activation. This was followed by a 40-hour recovery period, for a total incubation time of 44 hours. During the long exposures, cells were treated with the test articles without S9 for 27 hours, and with no recovery period. Osmolality and pH of the test mixtures were measured to assure that they had an osmolarity of <120% of the vehicle control and a pH in an acceptable range.

Two cultures were set up for each treatment condition and condensate concentration. Each culture was sampled to assess cytotoxicity using a Coulter counter and for the percentage of micronuclei by flow cytometric analysis using the FACSDiva software package and the MicroFlow kit (Litron, Rochester, NY). Ten thousand cells were analyzed in each culture for a total of at least 20,000 cells/ concentration.

1.6 Condensate Stability

Concentrations of primary constituents and water in the JUUL2 ENDS aerosol condensates measured at 0, 4 and 8 weeks after production, indicate condensate stability (stored at -65°C) over the period of biological testing. Concentrations at 4 and 8 weeks after condensate production expressed as a percentage of concentration measured at Week 0 is shown in Table 3. All primary constituents are present at concentrations > 85% of their concentration at Week 0.

Table S3: Concentrations of Water and Primary Constituents in Condensates Measured 4 and 8 Weeks After Production Expressed as Percentage of Analyte Concentration at week 0.

Assay	Condensate	Puffing Regime	Timepoint (Weeks)	% of analyte concentration at T=0					
				PG	Menthol	Nicotine	Glycerol	Water	Benzoic Acid
NRU - 3T3	Virginia Tobacco	Non Intense	4	96.8	NA	95.8	96.9	109.4	0.1
NRU - A549	Virginia Tobacco	Non Intense	4	109.9	NA	108.2	108.4	118.3	96.4
Ames, MN	Virginia Tobacco	Non Intense	4	97.5	NA	96.8	99.2	102.9	97.3
	Virginia Tobacco	Non Intense	8	101	NA	100.9	99.7	121.1	104.7
NRU - 3T3	Virginia Tobacco	Intense	4	95.2	NA	95.9	94.6	105.1	95.1
NRU - A549	Virginia Tobacco	Intense	4	125.6	NA	126.4	123.0	142.7	92.6
Ames, MN	Virginia Tobacco	Intense	4	98.2	NA	97.4	97.0	100.9	98.0
	Virginia Tobacco	Intense	8	102.7	NA	102.2	99.5	117.1	104.7
NRU - 3T3	Crisp Menthol	Non Intense	7	107.6	107.8	106.5	100.9	104.0	110.1
NRU - A549	Crisp Menthol	Non Intense	7	96.0	98.4	95.0	89.3	81.3	109.4
Ames, MN	Crisp Menthol	Non Intense	4	99.1	98.8	98.8	102.1	130.1	108.6
	Crisp Menthol	Non Intense	8	98.4	97.9	96.9	99.2	110.2	93.3
NRU - 3T3	Crisp Menthol	Intense	7	107.6	109.0	106.4	99.2	99.0	109.1
NRU - A549	Crisp Menthol	Intense	7	103.9	105.5	102.6	97.3	95.4	107.0
Ames, MN	Crisp Menthol	Intense	4	101.2	101.0	101.2	105.3	130.6	106.5
	Crisp Menthol	Intense	8	96.5	95.9	95.3	98.0	116.6	92.2
NRU - 3T3	Polar Menthol	Non Intense	4	103.9	103.1	105.1	105.4	95.6	112.3
NRU - A549	Polar Menthol	Non Intense	4	88.6	87.0	89.4	88.0	83.0	110.9
Ames, MN	Polar Menthol	Non Intense	4	96.0	97.3	94.4	98.3	101.3	98.9
	Polar Menthol	Non Intense	8	98.2	100.3	96.5	95.3	115.6	98.0
	Polar Menthol	Intense	26	99.1	100.3	92.8	97.9	111.0	111.0
NRU - 3T3	Polar Menthol	Intense	4	98.5	96.5	100.3	102.2	85.1	108.3
NRU - A549	Polar Menthol	Intense	4	98.6	97.2	100.8	102.9	95.8	112.0

Ames, MN	Polar Menthol	Intense	4	86.3	86.9	85.3	87.6	84.6	100.4
	Polar Menthol	Intense	8	90.2	92.7	89.0	88.0	102.8	98.4
	Polar Menthol	Intense	26	89.2	90.0	84.0	88.4	95.0	111.5
NRU (3T3/A549), Ames, MN	Autumn Tobacco	Non Intense	4	98.0	NA	96.6	97.9	93.5	86.1
	Autumn Tobacco	Non Intense	8	102.0	NA	102.5	102.1	105.6	91.4
	Autumn Tobacco	Intense	4	99.7	NA	97.7	104.0	97.5	87.1
	Autumn Tobacco	Intense	8	100.0	NA	100.6	105.3	122.1	93.6
NRU (3T3/A549), Ames, MN	Ruby Menthol	Non Intense	4	96.8	97.0	96.7	97.8	98.9	85.6
	Ruby Menthol	Non Intense	9	104.9	103.8	105.1	102.7	101.2	95.6
	Ruby Menthol	Intense	4	98.5	97.8	96.4	103.1	97.3	86.3
	Ruby Menthol	Intense	9	103.7	101.3	102.2	107.0	98.2	94.5
NRU (3T3/A549), Ames, MN	Summer Menthol	Non Intense	4	96.3	96.7	94.6	99.1	89.2	93.6
	Summer Menthol	Non Intense	9	99.3	100.4	98.9	105.0	90.4	107.2
	Summer Menthol	Intense	4	97.8	98.0	95.7	99.6	93.3	96.5
	Summer Menthol	Intense	9	99.1	100.5	98.1	103.8	93.6	107.7

2 Supplemental Biological Results

Figure S1. BALB/c 3T3 and A549 Cytotoxicity of Intense and Non-intense ENDS Aerosol (reported as ACM) and 3R4F Smoke Condensates (reported as TPM), Measured in the NRU Assay. The Dotted Line Represented the 70% Relative Viability Cut-Off Defining Cytotoxicity.

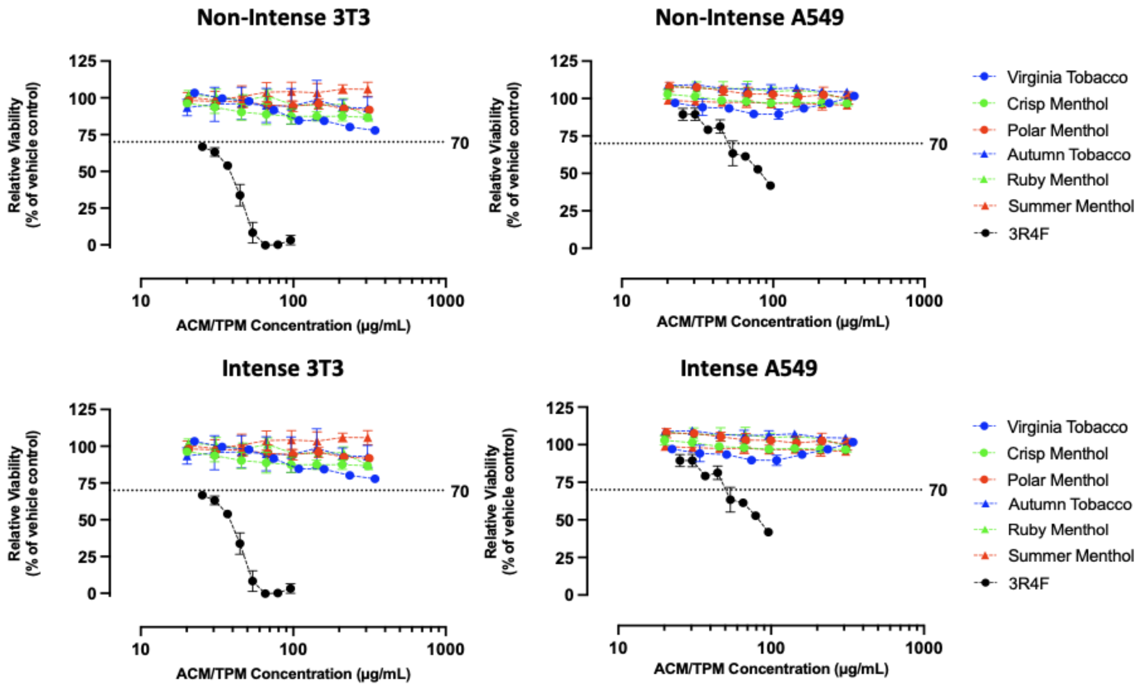

Figure S2. Ames assay results in *S. typhimurium* strains with and without metabolic activation conditions in which no increase in revertant counts were observed.

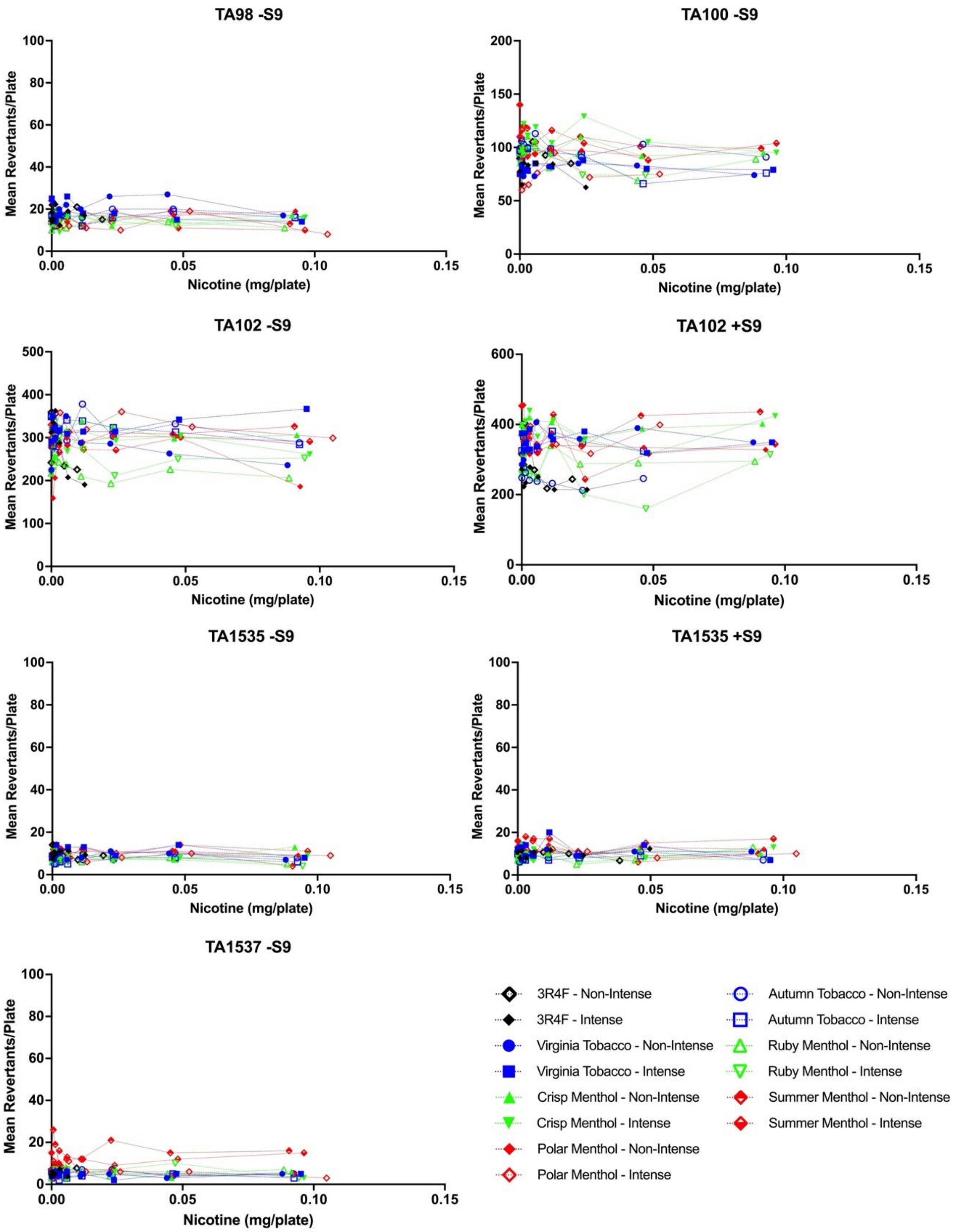

3R4F = Reference Cigarette 3R4F

Table S4. Vehicle and Positive Control Mean and SD Revertant Counts/Plate for Ames Assays of all JUUL2 products.

	Treatment Group	µg/plate	TA98		TA100		TA1535		TA1537		TA102	
			Mean	SD	Mean	SD	Mean	SD	Mean	SD	Mean	SD
Virginia Tobacco (18 mg/mL) Non Intense	WITHOUT ACTIVATION											
	200 Proof Ethanol	100 µL	25	5	75	2	9	3	4	1	225	37
	2-Nitrofluorene	2.5	1435	188								
	Sodium Azide	1.0			443	24	681	286				
	ICR-191 Acridine	0.5							618	58		
	4-Nitroquinoline-N-oxide	2.0									988	35
	WITH ACTIVATION											
	200 Proof Ethanol	100 µL	28	4	92	6	12	1	5	3	286	33
	2-Aminoanthracene	2.5	3140	128	2938	369	264	27	393	64		
	2-Aminoanthracene	10.0									2514	39
Virginia Tobacco (18 mg/mL) Intense	WITHOUT ACTIVATION											
	200 Proof Ethanol	100 µL	25	5	75	2	9	3	4	1	291	25
	2-Nitrofluorene	2.5	1435	188								
	Sodium Azide	1.0			443	24	681	286				
	ICR-191 Acridine	0.5							618	58		
	4-Nitroquinoline-N-oxide	2.0									1715	259
	WITH ACTIVATION											
	200 Proof Ethanol	100 µL	28	4	92	6	12	1	5	3	375	46
	2-Aminoanthracene	2.5	3140	128	2938	369	264	27	393	64		
	2-Aminoanthracene	10.0									2734	369

	Treatment Group	µg/plate	TA98		TA100		TA1535		TA1537		TA102	
			Mean	SD	Mean	SD	Mean	SD	Mean	SD	Mean	SD
**Crisp Menthol (18 mg/mL) Non Intense**	WITHOUT ACTIVATION											
	200 Proof Ethanol	100 µL	16	2	85	11	7	4	4	5	318	25
	2-Nitrofluorene	2.5	876	72								
	Sodium Azide	1.0			455	60	466	136				
	ICR-191 Acridine	0.5							670	463		
	4-Nitroquinoline-N-oxide	2.0									955	150
	WITH ACTIVATION											
	200 Proof Ethanol	100 µL	24	5	116	30	8	6	8	2	399	104
	2-Aminoanthracene	2.5	2207	224	2890	27	235	35	425	21		
	2-Aminoanthracene	10.0									3136	292
**Crisp Menthol (18 mg/mL) Intense**	WITHOUT ACTIVATION											
	200 Proof Ethanol	100 µL	16	2	85	11	7	4	4	5	318	25
	2-Nitrofluorene	2.5	876	72								
	Sodium Azide	1.0			455	60	466	136				
	ICR-191 Acridine	0.5							670	463		
	4-Nitroquinoline-N-oxide	2.0									955	150
	WITH ACTIVATION											
	200 Proof Ethanol	100 µL	24	5	116	30	8	6	8	2	399	104
	2-Aminoanthracene	2.5	2207	224	2890	27	235	35	425	21		
	2-Aminoanthracene	10.0									3136	292

	Treatment Group	µg/plate	TA98		TA100		TA1535		TA1537		TA102	
			**Mean**	**SD**	**Mean**	**SD**	**Mean**	**SD**	**Mean**	**SD**	**Mean**	**SD**
**Polar Menthol (18 mg/mL) Non Intense**	WITHOUT ACTIVATION											
	200 Proof Ethanol	100 µL	24	5	140	24	8	2	4	1	311	40
	2-Nitrofluorene	2.5	844	84								
	Sodium Azide	1.0			485	27	481	13				
	ICR-191 Acridine	0.5							297	90		
	4-Nitroquinoline-N-oxide	2.0									900	16
	WITH ACTIVATION											
	200 Proof Ethanol	100 µL	20[a]	1	152	12	16	5	7	2	453	22
	2-Aminoanthracene	2.5	1980	210	1319	250	89	23	139	26		
	2-Aminoanthracene	10.0									1053	187
**Polar Menthol (18 mg/mL) Intense**	WITHOUT ACTIVATION											
	200 Proof Ethanol	100 µL	24	5	140	24	10[a]	2	7	2	330	49
	2-Nitrofluorene	2.5	844	84								
	Sodium Azide	1.0			485	27	456	30				
	ICR-191 Acridine	0.5							559	33		
	4-Nitroquinoline-N-oxide	2.0									1147	103
	WITH ACTIVATION											
	200 Proof Ethanol	100 µL	20[a]	1	152	12	16	5	7	2	453	22
	2-Aminoanthracene	2.5	1980	210	1319	250	89	23	139	26		
	2-Aminoanthracene	10.0									1053	187

	Treatment Group	µg/plate	TA98		TA100		TA1535		TA1537		TA102	
			Mean	SD	Mean	SD	Mean	SD	Mean	SD	Mean	SD
**Autumn Tobacco (18 mg/mL) Non Intense**	WITHOUT ACTIVATION											
	200 Proof Ethanol	100 µL	17	4	97	10	8	3	6	4	349	17
	2-Nitrofluorene	2.5	665	56								
	Sodium Azide	1.0			645	102	657	20				
	ICR-191 Acridine	0.5							179	23		
	4-Nitroquinoline-N-oxide	2.0									1126	113
	WITH ACTIVATION											
	200 Proof Ethanol	100 µL	16	4	120	17	10	7	4	1	248	19
	2-Aminoanthracene	2.5	1764	186	1812	397	180	14	239	9		
	2-Aminoanthracene	10.0									2451	98
**Autumn Tobacco (18 mg/mL) Intense**	WITHOUT ACTIVATION											
	200 Proof Ethanol	100 µL	17	4	97	10	8	3	6	4	349	17
	2-Nitrofluorene	2.5	665	56								
	Sodium Azide	1.0			645	102	657	20				
	ICR-191 Acridine	0.5							179	23		
	4-Nitroquinoline-N-oxide	2.0									1126	113
	WITH ACTIVATION											
	200 Proof Ethanol	100 µL	16[a]	0	98	5	10	7	8	2	325	51
	2-Aminoanthracene	2.5	2100	466	1979	425	180	14	134	66		
	2-Aminoanthracene	10.0									3230	268

	Treatment Group	µg/plate	TA98		TA100		TA1535		TA1537		TA102	
			Mean	SD	Mean	SD	Mean	SD	Mean	SD	Mean	SD
**Ruby Menthol (18 mg/mL) Non Intense**	WITHOUT ACTIVATION											
	200 Proof Ethanol	100 µL	10	6	95	5	6	2	3	1	217[a]	8
	2-Nitrofluorene	2.5	852	257								
	Sodium Azide	1.0			598	28	853	147				
	ICR-191 Acridine	0.5							99	7		
	4-Nitroquinoline-N-oxide	2.0									815	110
	WITH ACTIVATION											
	200 Proof Ethanol	100 µL	21	7	93	19	7	3	8	5	260[a]	16
	2-Aminoanthracene	2.5	1775	240	2332	480	253	18	400	35		
	2-Aminoanthracene	10.0									2437	336
**Ruby Menthol (18 mg/mL) Intense**	WITHOUT ACTIVATION											
	200 Proof Ethanol	100 µL	12	5	97	10	8	3	6	4	217[a]	8
	2-Nitrofluorene	2.5	560	35								
	Sodium Azide	1.0			645	102	657	20				
	ICR-191 Acridine	0.5							179	23		
	4-Nitroquinoline-N-oxide	2.0									815	110
	WITH ACTIVATION											
	200 Proof Ethanol	100 µL	21	7	93	19	7	3	8	5	260[a]	16
	2-Aminoanthracene	2.5	1775	240	2332	480	253	18	400	35		
	2-Aminoanthracene	10.0									2437	336

	Treatment Group	µg/plate	TA98		TA100		TA1535		TA1537		TA102	
			Mean	SD	Mean	SD	Mean	SD	Mean	SD	Mean	SD
**Summer Menthol (18 mg/mL) Non Intense**	WITHOUT ACTIVATION											
	200 Proof Ethanol	100 µL	14	3	110	15	10	6	15	4	298	8
	2-Nitrofluorene	2.5	825	72								
	Sodium Azide	1.0			349	51	827	68				
	ICR-191 Acridine	0.5							84	40		
	4-Nitroquinoline-N-oxide	2.0									845	96
	WITH ACTIVATION											
	200 Proof Ethanol	100 µL	17	4	119	7	13	2	21	4	370	55
	2-Aminoanthracene	2.5	2189	215	2412	262	122	25	132	12		
	2-Aminoanthracene	10.0									2154	182
**Summer Menthol (18 mg/mL) Intense**	WITHOUT ACTIVATION											
	200 Proof Ethanol	100 µL	14	3	110	15	10	5	15	4	277	42
	2-Nitrofluorene	2.5	825	72								
	Sodium Azide	1.0			349	51	541	38				
	ICR-191 Acridine	0.5							84	40		
	4-Nitroquinoline-N-oxide	2.0									1168	153
	WITH ACTIVATION											
	200 Proof Ethanol	100 µL	17	4	119	7	16	2	21	4	370	55
	2-Aminoanthracene	2.5	2189	215	2412	262	244	45	132	12		
	2-Aminoanthracene	10.0									2154	182

Note: Increased mean revertant counts of *S. typhimurium* indicator strains treated with positive control chemicals relative to treatment with vehicle control (ethanol) indicates the sensitivity of the assay.

SD=standard deviation.

[a] Calculated from duplicate plates.

Figure S3. Mean MN Response (Left Panel) and Cytotoxicity (Right Panel) Of JUUL2 Aerosol Condensates (Non-Intense Puffing Regime) and 3R4F (Non-Intense Puffing Regime) Condensate in the MN Assay at the Short Term (4h) Without (Top) and With Metabolic Activation (Bottom) and Long Term (27h) Treatments Without Metabolic Activation (Middle)

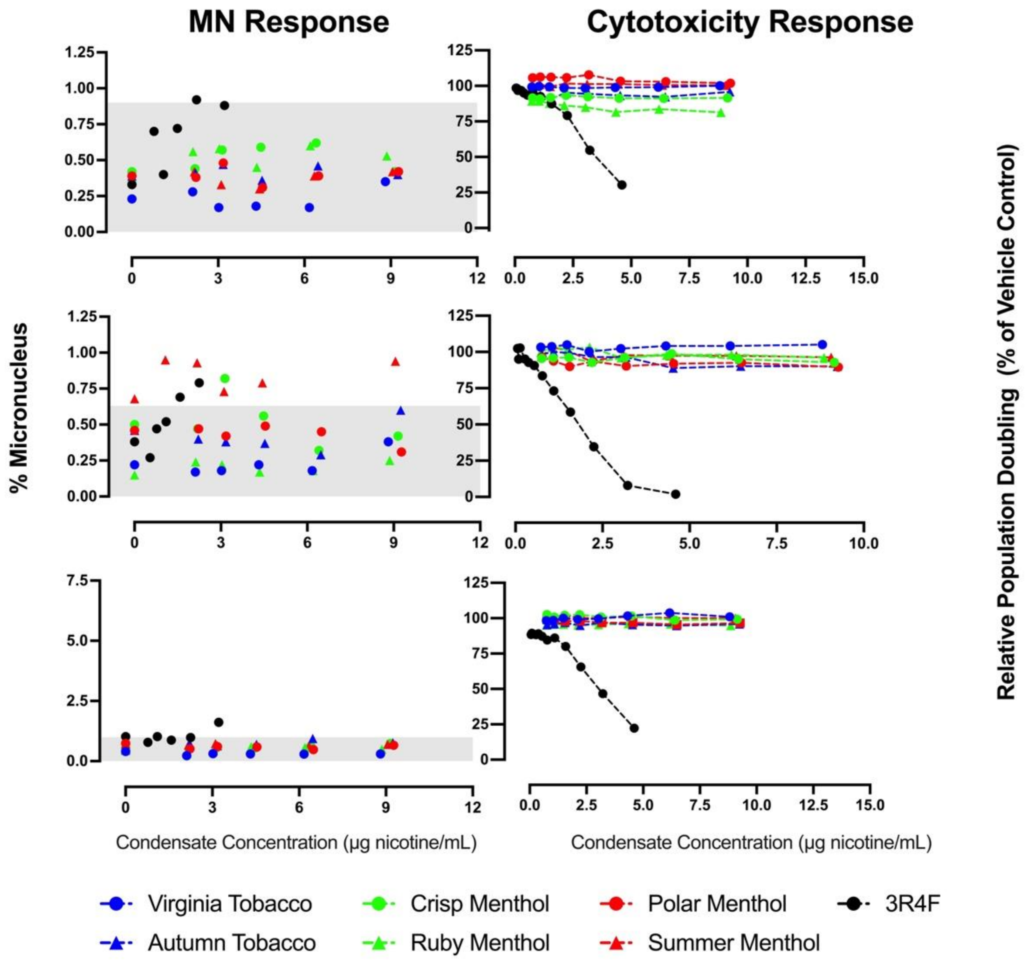

Historical negative control 95% Confidence Interval (%MN): 4hr-S9: 0.00-0.90; 27hr-S9: 0.00-0.63, 4hr+S9: 0.00-0.99. The shaded area denotes the historical negative control 95% Confidence Interval.

Table S5. Vehicle and Positive Control Mean and SD Micronucleus Counts in MN Assays for all JUUL2 products.

Virginia Tobacco (18 mg/mL) MN Assay Vehicle and Positive Control Responses

Treatment (%)	Culture	# Nuclei	# MN	Sum Nuclei	Sum MN	MN (%)	SD
4-Hour without S9 Metabolic Activation							
200 Proof Ethanol (1%)	A	10000	24	20000	45	0.23	0.0003
	B	10000	21				
MMC 0.0625 µg/mL	A	10000	116	20000	229	1.15	0.0008
	B	10000	113				
MMC 0.125 µg/mL	A	10000	340	20000	692	3.46	0.0013
	B	10000	352				
27-Hour without S9 Metabolic Activation							
200 Proof Ethanol (1%)	A	10014	21	20019	44	0.22	0.0003
	B	10005	23				
VIN 0.0025 µg/mL	A	10017	135	20046	336	1.68	0.0009
	B	10029	201				
VIN 0.003 µg/mL	A	10000	216	19998	547	2.74	0.0012
	B	9998	331				
4-Hour with S9 Metabolic Activation							
200 Proof Ethanol (1%)	A	10000	40	20000	79	0.40	0.0004
	B	10000	39				
CP 4.7 µg/mL	A	10000	150	20000	317	1.59	0.0009
	B	10000	167				
CP 11.9 µg/mL	A	10000	486	20000	973	4.87	0.0015
	B	10000	487				

Note: Increased MN counts in TK6 Cultures treated with positive control chemicals relative to treatment with vehicle control (ethanol) indicates the sensitivity of the assay.
CP = Cyclophosphamide Monohydrate; MMC = Mitomycin C; MN = Micronuclei; VIN = Vinblastine Sulfate.

Crisp Menthol MN Assay Vehicle and Positive Control Responses

Treatment (%)	Culture	# Nuclei	# MN	Sum Nuclei	Sum MN	MN (%)	SD
4-Hour without S9 Metabolic Activation							
200 Proof Ethanol (1%)	A	10025	53	20164	84	0.42	0.0005
	B	10139	31				
MMC 0.0625 µg/mL	A	10259	271	20294	494	2.43	0.0011
	B	10035	223				
MMC 0.125 µg/mL	A	10074	562	20234	1316	6.50	0.0017
	B	10160	754				
27-Hour without S9 Metabolic Activation							
200 Proof Ethanol (1%)	A	10020	25	20127	100	0.50	0.0005
	B	10107	75				
VIN 0.0025 µg/mL	A	10121	527	20299	977	4.81	0.0015
	B	10178	450				
VIN 0.003 µg/mL	A	10093	519	20158	1078	5.35	0.0016
	B	10065	559				
4-Hour with S9 Metabolic Activation							
200 Proof Ethanol (1%)	A	10020	52	20034	108	0.54	0.0005
	B	10014	56				
CP 4.7 µg/mL	A	10021	219	20075	446	2.22	0.0010
	B	10054	227				
CP 11.9 µg/mL	A	10187	1020	20309	1912	9.41	0.0020
	B	10122	892				

Note: Increased MN counts in TK6 Cultures treated with positive control chemicals relative to treatment with vehicle control (ethanol) indicates the sensitivity of the assay.
CP = Cyclophosphamide Monohydrate; MMC = Mitomycin C; MN = Micronuclei; VIN = Vinblastine Sulfate.

Polar Menthol MN Assay Vehicle and Positive Control Responses

Treatment (%)	Culture	# Nuclei	# MN	Sum Nuclei	Sum MN	MN (%)	SD
4-Hour without S9 Metabolic Activation							
200 Proof Ethanol (1%)	A	10168	40	20288	79	0.39	0.0004
	B	10120	39				
MMC 0.0625 µg/mL	A	10398	163	20781	380	1.83	0.0009
	B	10383	217				
MMC 0.125 µg/mL	A	10693	660	21491	1355	6.30	0.0017
	B	10798	695				
27-Hour without S9 Metabolic Activation							
200 Proof Ethanol (1%)	A	10145	60	20224	93	0.46	0.0005
	B	10079	33				
VIN 0.0025 µg/mL	A	10226	455	20405	819	4.01	0.0014
	B	10179	364				
VIN 0.003 µg/mL	A	10435	469	20727	912	4.40	0.0014
	B	10292	443				
4-Hour with S9 Metabolic Activation							
200 Proof Ethanol (1%)	A	10263	71	20364	150	0.74	0.0006
	B	10101	79				
CP 4.7 µg/mL	A	10287	202	20777	394	1.90	0.0009
	B	10490	192				
CP 11.9 µg/mL	A	11512	884	22556	1775	7.87	0.0018
	B	11044	891				

Note: Increased MN counts in TK6 Cultures treated with positive control chemicals relative to treatment with vehicle control (ethanol) indicates the sensitivity of the assay.
CP = Cyclophosphamide Monohydrate; MMC = Mitomycin C; MN = Micronuclei; VIN = Vinblastine Sulfate.

Autumn Tobacco MN Assay Vehicle and Positive Control Responses

Treatment (%)	Culture	# Nuclei	# MN	Sum Nuclei	Sum MN	MN (%)	SD
4-Hour without S9 Metabolic Activation							
200 Proof Ethanol (1%)	A	10000	42	20000	77	0.39	0.0004
	B	10000	35				
MMC 0.0625 µg/mL	A	10000	257	20000	483	2.42	0.0011
	B	10000	226				
MMC 0.125 µg/mL	A	10000	529	20000	1032	5.16	0.0016
	B	10000	503				
27-Hour without S9 Metabolic Activation							
200 Proof Ethanol (1%)	A	10000	55	20000	92	0.46	0.0005
	B	10000	37				
VIN 0.0025 µg/mL	A	10000	1583	20000	4011	20.06	0.0028
	B	10000	2428				
VIN 0.003 µg/mL	A	10000	2163	20000	4131	20.66	0.0029
	B	10000	1968				
4-Hour with S9 Metabolic Activation							
200 Proof Ethanol (1%)	A	10000	42	20000	96	0.48	0.0005
	B	10000	54				
CP 4.7 µg/mL	A	10000	687	20000	1332	6.66	0.0018
	B	10000	645				
CP 11.9 µg/mL	A	10000	2098	20000	3988	19.94	0.0028
	B	10000	1890				

Note: Increased MN counts in TK6 Cultures treated with positive control chemicals relative to treatment with vehicle control (ethanol) indicates the sensitivity of the assay.
CP = Cyclophosphamide Monohydrate; MMC = Mitomycin C; MN = Micronuclei; VIN = Vinblastine Sulfate.

Ruby Menthol MN Assay Vehicle and Positive Control Responses

Treatment (%)	Culture	# Nuclei	# MN	Sum Nuclei	Sum MN	MN (%)	SD
4-Hour without S9 Metabolic Activation							
200 Proof Ethanol (1%)	A	10000	34	20000	76	0.38	0.0004
	B	10000	42				
MMC 0.0625 µg/mL	A	10000	206	20000	407	2.04	0.0010
	B	10000	201				
MMC 0.125 µg/mL	A	10000	523	20000	1111	5.56	0.0016
	B	10000	588				
27-Hour without S9 Metabolic Activation							
200 Proof Ethanol (1%)	A	10000	21	20000	29	0.15	0.0003
	B	10000	8				
VIN 0.0025 µg/mL	A	10000	216	20000	457	2.29	0.0011
	B	10000	241				
VIN 0.003 µg/mL	A	10000	454	20000	900	4.50	0.0015
	B	10000	446				
4-Hour with S9 Metabolic Activation							
200 Proof Ethanol (1%)	A	10000	45	20000	88	0.44	0.0005
	B	10000	43				
CP 4.7 µg/mL	A	10000	608	20000	1098	5.49	0.0016
	B	10000	490				
CP 11.9 µg/mL	A	7315	990	10797	1505	13.94	0.0033
	B	3482	515				

Note: Increased MN counts in TK6 Cultures treated with positive control chemicals relative to treatment with vehicle control (ethanol) indicates the sensitivity of the assay.
CP = Cyclophosphamide Monohydrate; MMC = Mitomycin C; MN = Micronuclei; VIN = Vinblastine Sulfate.

Summer Menthol MN Assay Vehicle and Positive Control Responses

Treatment (%)	Culture	# Nuclei	# MN	Sum Nuclei	Sum MN	MN (%)	SD
4-Hour without S9 Metabolic Activation							
200 Proof Ethanol (1%)	A	10017	41	20017	71	0.35	0.0004
	B	10000	30				
MMC 0.0625 µg/mL	A	10000	271	20000	559	2.80	0.0012
	B	10000	288				
MMC 0.125 µg/mL	A	10000	749	20000	1473	7.37	0.0018
	B	10000	724				
27-Hour without S9 Metabolic Activation							
200 Proof Ethanol (1%)	A	10000	67	20000	136	0.68	0.0006
	B	10000	69				
VIN 0.0025 µg/mL	A	10000	648	20000	1096	5.48	0.0016
	B	10000	448				
VIN 0.003 µg/mL	A	10000	690	20000	1392	6.96	0.0018
	B	10000	702				
4-Hour with S9 Metabolic Activation							
200 Proof Ethanol (1%)	A	10000	71	20000	143	0.72	0.0006
	B	10000	72				
CP 4.7 µg/mL	A	10000	531	20000	1113	5.57	0.0016
	B	10000	582				
CP 11.9 µg/mL	A	10000	1425	20000	2930	14.65	0.0025
	B	10000	1505				

Note: Increased MN counts in TK6 Cultures treated with positive control chemicals relative to treatment with vehicle control (ethanol) indicates the sensitivity of the assay.
CP = Cyclophosphamide Monohydrate; MMC = Mitomycin C; MN = Micronuclei; VIN = Vinblastine Sulfate.
