# Supplementary material for: A Practical Framework for Novel Electronic Nicotine Delivery System Evaluation: Chemical and Toxicological Characterization of JUUL2 Aerosol and Comparison with Reference Cigarettes
Source: Toxics. 2024 Jan 4;12(1):41. doi: 10.3390/toxics12010041 (PMC10820849; doi:10.3390/toxics12010041)
Supplement: Supplementary file 1 [file toxics-12-00041-s001.zip › Supplementary Material Table of Contents_Final.pdf]

Supplementary Material Table of Contents

Supplemental Materials File S1

1. Methodologies for Targeted Chemical Characterization of Aerosol
    1.1. Analysis of Primary Constituents
    1.2. Analysis of Water by Karl Fischer
    1.3. Analysis of Carbonyls
    1.4. Analysis of Nicotine Degradants
    1.5. Analysis of Tobacco Specific Nitrosamines (TSNAs)
    1.6. Analysis of Organic Acids
    1.7. Analysis of Volatile Organic Compounds (VOCs)
    1.8. Analysis of Glycidol
    1.9. Analysis of Metals
    1.10. Analysis of Aromatic Flavourants
    1.11. Analysis of pH in Aerosol
        1.11.1. Table 1. Chemical Characterization Methodologies
        1.11.2. Figure 1. Example Aerosol Collection Apparatus
2. Chemical Analyses of Aerosol Condensates for Biological Analysis
    2.1. Primary Constituents
    2.2. Organic Acids (Benzoic Acid)
    2.3. Carbonyl Compounds
3. Methodologies for Targeted Chemical Characterization of Combustible Cigarette Smoke
    3.1. Glycidol
4. Comparison to Combustible Cigarettes
    4.1. 1-EOL Yield Calculation
        4.1.1. Equation 1. Calculation of JUUL2 System 1-EOL ENDS Aerosol Yield Normalized to Nicotine
    4.2. Background Subtraction
        4.2.1. Table 2. Background Subtraction Approach for Aerosol Samples
5. Limit of Detection and Quantification
    5.1. Determination of Limit of Detection and Quantification
        5.1.1. Equation 2. Method Detection Limit Calculation
        5.1.2. Equation 3. Per Collection LOD and LOQ Calculation
        5.1.3. Equation 4. Per Puff LOD and LOQ Calculations
    5.2. Approximation of LOD or LOQ Results
        5.2.1. Table 3. Numeric Imputation of Limit of Detection and Limit of Quantitation Values
6. Aerosol Comparison Reporting Methodology
    6.1. Percent Difference
        6.1.1. Equation 2 Calculation of Percent Difference
7. Stability Trends and Analysis
    7.1. Primary Constituents, Organic Acids, and pH
    7.2. Thermal Degradants

7.3. Esters and Alcohols
7.4. Nicotine Degradants
7.5. Metals
7.6. VOCs
7.7. TSNAs

Supplemental Materials File S2

1. Per Collection Limits Summary
   1.1. Virginia Tobacco 18 mg/mL Non-Intense Per Collection Limit of Detection and Quantification Values
   1.2. Virginia Tobacco 18 mg/mL Intense Per Collection Limit of Detection and Quantification Values
   1.3. Crisp Menthol 18 mg/mL Non-Intense Per Collection Limit of Detection and Quantification Values
   1.4. Crisp Menthol 18 mg/mL Intense Per Collection Limit of Detection and Quantification Values
   1.5. Polar Menthol 18 mg/mL Non-Intense Per Collection Limit of Detection and Quantification Values
   1.6. Polar Menthol 18 mg/mL Intense Per Collection Limit of Detection and Quantification Values
   1.7. Autumn Tobacco 18 mg/mL Non-Intense Per Collection Limit of Detection and Quantification Values
   1.8. Autumn Tobacco 18 mg/mL Intense Per Collection Limit of Detection and Quantification Values
   1.9. Ruby Menthol 18 mg/mL Non-Intense Per Collection Limit of Detection and Quantification Values
   1.10. Ruby Menthol 18 mg/mL Intense Per Collection Limit of Detection and Quantification Values
   1.11. Summer Menthol 18 mg/mL Non-Intense Per Collection Limit of Detection and Quantification Values
   1.12. Summer Menthol 18 mg/mL Intense Per Collection Limit of Detection and Quantification Values

Supplemental Materials File S3

1. Non-Intense and Intense Per Collection Analytical Stability Results
   1.1. Virginia Tobacco 18 mg/mL Non-Intense Per Collection Analytical Stability Results
   1.2. Virginia Tobacco 18 mg/mL Intense Per Collection Analytical Stability Results
   1.3. Crisp Menthol 18 mg/mL Non-Intense Per Collection Analytical Stability Results
   1.4. Crisp Menthol 18 mg/mL Intense Per Collection Analytical Stability Results
   1.5. Polar Menthol 18 mg/mL Non-Intense Per Collection Analytical Stability Results
   1.6. Polar Menthol 18 mg/mL Intense Per Collection Analytical Stability Results
   1.7. Autumn Tobacco 18 mg/mL Non-Intense Per Collection Analytical Stability Results
   1.8. Autumn Tobacco 18 mg/mL Intense Per Collection Analytical Stability Results

1.9. Ruby Menthol 18 mg/mL Non-Intense Per Collection Analytical Stability Results
1.10. Ruby Menthol 18 mg/mL Intense Per Collection Analytical Stability Results
1.11. Summer Menthol 18 mg/mL Per Collection Analytical Stability Results
1.12. Summer Menthol 18 mg/mL Intense Per Collection Per Collection Analytical Stability Results

Supplemental Materials File S4

1. Non-Intense and Intense (mg/mg) Nicotine Normalized Analytical Stability Results
   1.1. Virginia Tobacco 18 mg/mL Non-Intense mg/mg Nicotine Normalized Analytical Stability Results
   1.2. Virginia Tobacco 18 mg/mL Intense Nicotine Normalized Analytical Stability Results
   1.3. Crisp Menthol 18 mg/mL Non-Intense Nicotine Normalized Analytical Stability Results
   1.4. Crisp Menthol 18 mg/mL Intense Nicotine Normalized Analytical Stability Results
   1.5. Polar Menthol 18 mg/mL Non-Intense Nicotine Normalized Analytical Stability Results
   1.6. Polar Menthol 18 mg/mL Intense Nicotine Normalized Analytical Stability Results
   1.7. Autumn Tobacco 18 mg/mL Non-Intense Nicotine Normalized Analytical Stability Results
   1.8. Autumn Tobacco 18 mg/mL Intense Nicotine Normalized Analytical Stability Results
   1.9. Ruby Menthol 18 mg/mL Non-Intense Nicotine Normalized Analytical Stability Results
   1.10. Ruby Menthol 18 mg/mL Intense Nicotine Normalized Analytical Stability Results
   1.11. Summer Menthol 18 mg/mL Non-Intense Nicotine Normalized Analytical Stability Results
   1.12. Summer Menthol 18 mg/mL Intense Nicotine Normalized Analytical Stability Results
   1.13. Non-Intense (ISO 3308:2012) Combustible Reference Cigarette Constituent Values
   1.14. Intense (ISO 20778:2018) Combustible Reference Cigarette Constituent Values

Supplemental Materials File S5

1. Percent Difference to Combustible Cigarettes
   1.1. Virginia Tobacco 18 mg/mL Non-Intense Percent Difference to Combustible Cigarettes
   1.2. Virginia Tobacco 18 mg/mL Intense Percent Difference to Combustible Cigarettes
   1.3. Crisp Menthol 18 mg/mL Non-Intense Percent Difference to Combustible Cigarettes
   1.4. Crisp Menthol 18 mg/mL Intense mg/mg Percent Difference to Combustible Cigarettes
   1.5. Polar Menthol 18 mg/mL Non-Intense Percent Difference to Combustible Cigarettes
   1.6. Polar Menthol 18 mg/mL Intense Percent Difference to Combustible Cigarettes
   1.7. Autumn Tobacco 18 mg/mL Non-Intense Percent Difference to Combustible Cigarettes
   1.8. Autumn Tobacco 18 mg/mL Intense Percent Difference to Combustible Cigarettes
   1.9. Ruby Menthol 18 mg/mL Non-Intense Percent Difference to Combustible Cigarettes
   1.10. Ruby Menthol 18 mg/mL Intense Percent Difference to Combustible Cigarettes
   1.11. Summer Menthol 18 mg/mL mg/mg Percent Difference to Combustible Cigarettes

1.12. Summer Menthol 18 mg/mL Intense Percent Difference to Combustible Cigarettes
1.13. Average Non-Intense and Intense Percent Difference to Combustible Cigarettes
1.14. Statistical Comparison of Non-Intense 1R6F COA Values to Quantifiable Non-Intense JUUL2 Virginia Tobacco Constituents
1.15. Statistical Comparison of Intense 1R6F COA Values to Quantifiable Intense JUUL2 Virginia Tobacco Constituents

Supplemental Materials File S6

1. In Vitro Methodologies for Toxicological Characterization of Aerosol
    1.1. Cell Culture
    1.2. S9 Metabolic Activation System
    1.3. Neutral Red Uptake (NRUI) Cytotoxicity (OECD Guideline 129)
        1.3.1. Table 1. Neutral Red Uptake Dosing
    1.4. Ames Reverse Mutation Assay (OECD Guidance 471)
        1.4.1. Table 2. Ames Treatment Groups
    1.5. Micronucleus (MN) Assay (OECD Guidance 487)
    1.6. Condensate Stability
        1.6.1. Table 3. Concentrations of Water and Primary Constituents in Condensates Measured 4 and 8 Weeks After Production Expressed as Percentage of Analyte Concentration at week 0.
2. Supplemental Biological Results
    2.1. Figure 1. BALB/c 3T3 and A549 Cytotoxicity of Intense and Non-intense ENDS Aerosol (reported as ACM) and 3R4F Smoke Condensates (reported as TPM), Measured in the NRU Assay. The Dotted Line Represented the 70% Relative Viability Cut-Off Defining Cytotoxicity.
    2.2. Figure 2. Ames assay results in *S. typhimurium* strains with and without metabolic activation conditions in which no increase in revertant counts were observed.
    2.3. Table 4. Vehicle and Positive Control Mean and SD Revertant Counts/Plate for Ames Assays of all JUUL2 products.
    2.4. Figure 2. Mean MN Response (Left Panel) and Cytotoxicity (Right Panel) Of JUUL2 Aerosol Condensates (Non-Intense Puffing Regime) and 3R4F (Non-Intense Puffing Regime) Condensate in the MN Assay at the Short Term (4h) Without (Top) and With Metabolic Activation (Bottom) and Long Term (27h) Treatments Without Metabolic Activation (Middle).
    2.5. Table 5. Vehicle and Positive Control Mean and SD Micronucleus Counts in MN Assays for all JUUL2 products.
