# Supplementary material for: A Practical Framework for Novel Electronic Nicotine Delivery System Evaluation: Chemical and Toxicological Characterization of JUUL2 Aerosol and Comparison with Reference Cigarettes
Source: Toxics. 2024 Jan 4;12(1):41. doi: 10.3390/toxics12010041 (PMC10820849; doi:10.3390/toxics12010041)
Supplement: Supplementary file 1 [file toxics-12-00041-s001.zip › Supplementary Material_File S1_Analysis of Chemical Constituents and Method Details__Clean.pdf]

Supplementary Materials File S1

1 Methodologies for Targeted Chemical Characterization of Aerosol

All chemical methods described below were validated and were included in the laboratory’s A2LA scope of accreditation at the time of testing (Enthalpy Analytical Richmond Certificate number 1873.01; Enthalpy Analytical Durham Certificate Number 3198.01), and all internal quality control procedures were followed.

1.1 Analysis of Primary Constituents

Aerosol samples were collected by passing aerosol through a glass fiber filter pad (GFFP). Pads were extracted in a solution of IPA containing 1,4-butanediol and quinolone (internal standards). Extracts were analyzed via a gas chromatograph equipped with a flame injection detector and thermal conductivity detector using an Agilent DB-ALC1 30m x 0.32mm x 1.8µm capillary column.

1.2 Analysis of Water by Karl Fischer

An e-liquid is aliquoted into a glass autosampler vial and weighed in the vial with the cap on before analysis. The e-liquid is then analyzed by KF titration and, again, weighed in the vial with the cap on after analysis. The percentage of water is then calculated based on the results from the instrument and the difference in mass between the pre-weight and post-weight for each sample.

1.3 Analysis of Carbonyls

Aerosol samples were collected by passing aerosol through a GFFP and a single impinger containing a 1:1 solution of acetonitrile, isopropyl alcohol, and internal standards kept at -35 °C by submersion in a water/methanol bath. Following collection, the filter pad was extracted in the impinger solution and the mixture was derivatized with 2,4-Dinitrophenylhydrazine. Samples were analyzed via an ultra-performance liquid chromatograph equipped with a tandem mass spectrometry detector in Selective Ion Monitoring mode using a Waters Acquity BEH C18, 2.1mm x 50mm column, with 1.7µm pore size.

1.4 Analysis of Nicotine Degradants

Aerosol samples were collected onto a GFFP and extracted with a 70:30 solution of methanol and water containing internal standard. The extract is then analyzed by a Liquid Chromatograph (LC) with Tandem Mass Spectrometry Detection (LC-MS/MS).

1.5 Analysis of Tobacco Specific Nitrosamines (TSNAs)

Aerosol samples were collected by passing aerosol through a GFFP and extracted with deionized water (DI, Millipore). An aliquot of the subsequent aerosol extract is solvent-exchanged into a solution more suitable for analysis on a Gas Chromatograph (GC) with Tandem Mass Spectrometry Detection (MS/MS).

1.6 Analysis of Organic Acids

Aerosol samples were collected into a series of two fritted tipped impingers/bubblers containing a DI water (Millipore system) trap at pH 7. Each impinger/bubbler was immersed into an ice-water bath during the aerosol collection. The collected impinger/bubbler contents are combined into a conical tube and mixed together to ensure homogeneity. The subsequent aerosol extract is analyzed by an Ion Chromatograph (IC) with Conductivity Detection utilizing an Anion Exchange column.

1.7 Analysis of Volatile Organic Compounds

Aerosol samples were collected onto a coconut shell charcoal (CSC) sorbent tube (SKC, Anasorb CSC). The CSC front sorbent with the surrounding glass wool was removed and added to an 8 mL glass extraction vial containing a carbon disulfide (CS2) trapping solution. During the process of desorbing the CSC tubes, the extraction vials containing CS2 were capped and immersed into an ice-water bath to prevent rapid volatilization of the extract. The subsequent extract was transferred to a vial with zero headspace for analysis on a GC with Mass Spectrometry Detection (MS) in Selective Ion Monitoring (SIM) mode.

1.8 Analysis of Glycidol

Aerosol samples were collected by passing aerosol through an impinger containing a trapping solution with internal standard, hydrochloric acid, and p-toluenesulfonyl chloride. The derivatized Glycidol was extracted from the trapping solution with hexane and analyzed via a gas chromatograph equipped with a mass spectrometry detector using a 5m section of Restek Rxi-17Sil MS 0.25mm o.d. x 0.25µm column followed by a Restek Rxi-5Sil MS 30m x 0.25mm x 0.5µm column.

1.9 Analysis of Metals

Aerosol samples were collected using a Cerulean SM450e 20 port linear e-cigarette testing machine equipped with an electrostatic precipitation (EP) unit, including quartz glass containing a tungsten electrode. A high voltage was applied between the tube and electrode, causing aerosol particles to acquire charge and deposit on the tube walls. The EP tube was then rinsed with semi-conductor grade methanol to extract the collected aerosol. Sample extracts were digested with a concentrated nitric acid solution and analyzed via inductively coupled plasma mass spectrometry.

1.10 Analysis of Aromatic Flavourants

Aerosol samples were collected by passing aerosol through a GFFP in-line with a single fritted tipped impinger/bubbler containing a trapping solution of acetone with internal standard. The GFFPs were extracted with their respective impinger contents and internal standard. The subsequent aerosol extract was analyzed on a GC-MS.

1.11 Analysis of pH in Aerosol

E-Cigarettes are vaped using an analytical vaping machine. The vapor is collected into a single impinger containing 0.1 M potassium chloride. The impinger contents are mixed and pH is measured with a pH probe.

Table S1. Chemical Characterization Methodologies

Method Name	Constituent	CAS	Unit of Measure	Method of Capture	Analysis Method	Instrument	Method Reference Code, Accredited
**Aromatic Flavorants**							
Analysis of Aromatic Flavorants and Esters in E-Cigarette Aerosol by GC/MS	1-Butanol	71-36-3	mcg/collection	pad/impinger	Pads are extracted with Acetone	GC/MS	ENT218, Accredited
	Benzyl Acetate	140-11-4	mcg/collection				
	Ethyl Acetate	141-78-6	mcg/collection				
	Furfural	98-01-1	mcg/collection				
	Isoamyl Acetate	123-92-2	mcg/collection				
	Isobutyl Acetate	110-19-0	mcg/collection				
	Methyl Acetate	79-20-9	mcg/collection				
**Carbonyls**							
Carbonyls in E-Liquids and Aerosol by LC-MS/MS	Acetaldehyde	75-07-0	mcg/collection	pad/impinger	Pad extracted in Acetonitrile/IPA impinger solution and the mixture derivatized with 2,4-Dinitrophenylhydrazine	LC-MS/MS	AM-244, Accredited
	Acetyl Propionyl	600-14-6	mcg/collection				
	Acrolein	107-02-8	mcg/collection				
	Crotonaldehyde	123-73-9	mcg/collection				
	Diacetyl	431-03-08	mcg/collection				
	Ethyl Acetoacetate	141-97-9	mcg/collection				
	Formaldehyde	50-00-0	mcg/collection				
	n-Butyraldehyde	123-72-8	mcg/collection				
**Nicotine Degradants**							
Nicotine degradants in E-Cigarette Aerosol by LC-MS/MS	β-Nicotyrine	487-19-4	mcg/collection	pad	Pad extracted with 70:30 Methanol:Water	LC-MS/MS	AM-238, Accredited
	Anabasine	34366-21-7	mcg/collection				
	Anatabine	126454-22-6	mcg/collection				
	Cotinine	15569-85-4	mcg/collection				
	Myosmine	0532-12-7	mcg/collection				
	Nicotine-N-Oxide	51095-86-4	mcg/collection				
	Nornicotine	5746-86-1	mcg/collection				

**Primary Constituents**							
Analysis of Primary Constituents in E-Cigarette Aerosol by GC-FID / TCD	Benzoic Acid	65-85-0	mg/collection	pad	Pad extracted with IPA	GC-FID/TCD	ENT185, Accredited
	Diethylene Glycol	111-46-6	mg/collection				
	Ethylene Glycol	107-21-1	mg/collection				
	Glycerol	56-81-5	mg/collection				
	Menthol	89-78-1	mg/collection				
	Nicotine	54-11-5	mg/collection				
	Propylene Glycol	57-55-6	mg/collection				
	Water	7732-18-5	mg/collection				
Analysis of Water in E-Cigarette Liquids and Aerosol by Karl Fischer Titration	Water	7732-18-5	mg/collection		Titration	Karl Fischer	ENT073 Accredited
**Propionic Acid**							
Analysis of Organic Acids in E-Cigarette Liquids and Aerosol by HPLC/IC	Propionic Acid	79-09-4	mcg/collection	pad/impinger	The impinger solution containing pH7 DI Water is analyzed directly	IC-Conductivity	ENT327, Accredited
**Tobacco Specific Nitrosamines**							
Analysis of TSNAs in E-Cigarette Liquids and Aerosol by GC/MS/MS	NNK	64091-91-4	ng/collection	pad	Pad extracted with DI Water; solvent exchanged	GC-MS/MS	ENT211, Accredited
	NNN	16543-55-8	ng/collection				
	**Metals**						
Selected Metals in ENDS aerosol by ICP-MS	Arsenic	7440-38-2	ng/collection	electrostatic precipitation with quartz EP tube connected to the unit with a tungsten electrode.	Quartz Tube extracted with Methanol and digested with nitric acid	ICP-MS	AM-249, Accredited
	Beryllium	7440-41-7	ng/collection				
	Cadmium	7440-43-9	ng/collection				
	Chromium	7440-47-3	ng/collection				
	Cobalt	7440-48-4	ng/collection				
	Copper	7440-50-8	ng/collection				
	Gold	7440-57-5	ng/collection				
	Iron	7439-89-6	ng/collection				
	Lead	7439-92-1	ng/collection				
	Nickel	7440-02-0	ng/collection				
	Selenium	7782-49-2	ng/collection				
	Silver	7440-22-4	ng/collection				

	Tin	7440-31-5	ng/collection				
	Zinc	7440-66-6	ng/collection				
**Volatile Organic Compounds**							
Analysis of Low-Level Volatile Organic Compounds in E-Cigarette Liquids and Aerosol by GC/MS	1,3-butadiene	106-99-0	mcg/collection	coconut shell charcoal (CSC) sorbent tube	extraction vial containing a carbon disulfide (CS_2)	GC-MS (SIM)	ENT208A, Accredited
	Acrylonitrile	107-13-1	mcg/collection				
	Benzene	71-43-2	mcg/collection				
	Isoprene	78-79-5	mcg/collection				
	Propylene Oxide	75-56-9	mcg/collection				
	Toluene	108-88-3	mcg/collection				
**Glycidol**							
Analysis of Glycidol in Aerosol & E-liquid and Glycidol by GC/MS	Glycidol	556-52-5	mcg/collection	pad/impinger	Impinger solution containing HCl and pTSA extracted with Hexanes	GC-MS	ENT203, Accredited
**pH**							
Analysis of pH in E-Cigarette Aerosol	pH	N/A	pH	impinger	pH electrode	electrode	ENT056, Accredited

Figure S1. Example Aerosol Collection Apparatus

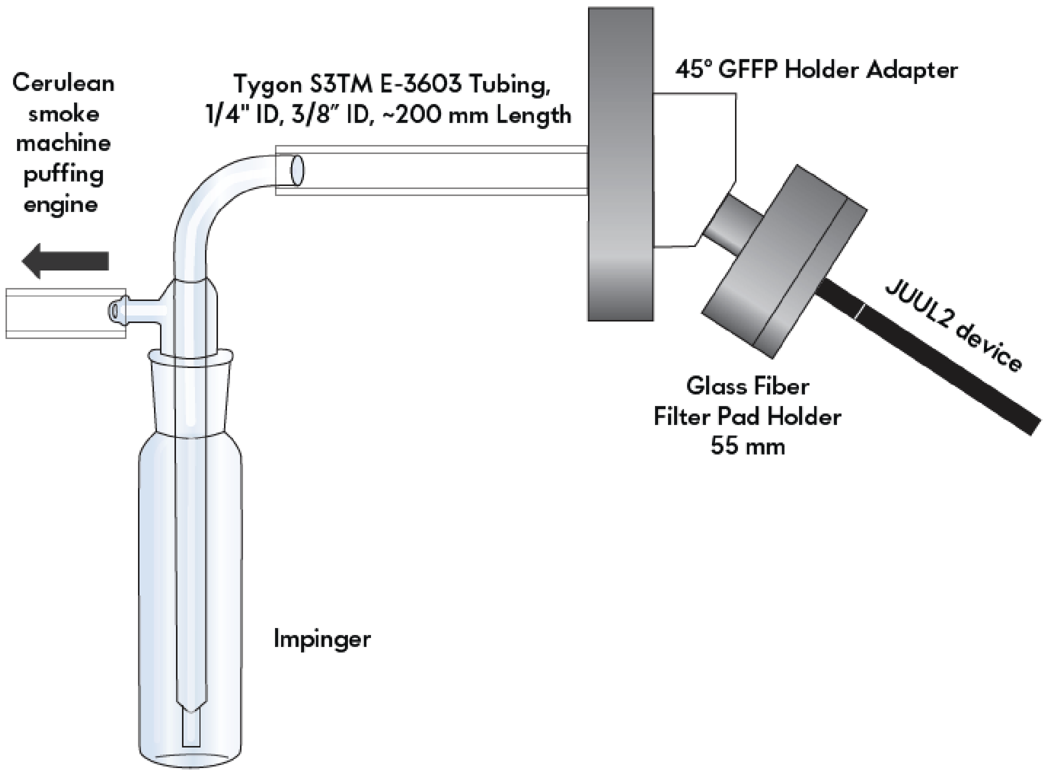

2 Chemical Analyses of Aerosol Condensates for Biological Analysis

All chemical methods described below were validated and were included in the laboratory's A2LA scope of accreditation at the time of testing (Enthalpy Analytical Richmond Certificate number 1873.01; Enthalpy Analytical Durham Certificate Number 3198.01), and all internal quality control procedures were followed. Samples were analyzed in quintuplicate.

2.1 Primary Constituents

Nicotine, menthol, water, propylene glycol, and glycerol concentrations were measured by Enthalpy Analytical (Richmond, VA), LLC in accordance with Enthalpy SOP AM-224. For the analysis, 1.0 mL of the aerosol condensate was added to 5.0 mL of extraction solution containing the internal standards (quinoline and 1,4-butanediol) and mixed. Extracts were analyzed by gas chromatography with flame ionization detection and thermal conductivity detection.

2.2 Organic Acids (Benzoic Acid)

Benzoic acid concentrations were measured by Enthalpy Analytical (Durham, VA), LLC in accordance with Enthalpy SOP ENT-327. Aerosol condensate was diluted no less than 5-fold with deionized water. Analysis was performed by ion chromatography with suppressed conductivity detection. Compounds were separated by charge and size and then detected based on mobile phase conductivity changes as it passed through the detector.

2.3 Carbonyl Compounds

Acetaldehyde, formaldehyde, acrolein, and crotonaldehyde concentrations were measured by Enthalpy Analytical (Richmond, VA), LLC in accordance with Enthalpy SOP AM-244. An aliquot of the aerosol condensate was derivatized with 2,4-Dinitrophenylhydrazine. Internal standards (Formaldehyde-d2, Acetaldehyde-d4, and Crotonaldehye-d3) were added to the sample prior to extraction. Separation and quantification were performed using ultra-high performance liquid chromatography tandem mass spectroscopy and were analyzed in single ion recording mode.

3 Methodologies for Targeted Chemical Characterization of Combustible Cigarette Smoke

3.1 Glycidol

Tobacco smoke particulate matter is collected using a Cambridge Filter Pad. After smoking, the pad is placed in a centrifuge tube with extraction/derivatization solution (acetone with hydrochloric acid, p-toluenesulfonyl chloride, and internal standard) and mixed at room temperature to ensure derivatization. Hexane and water are added to the trapping solution and the samples are mixed again. After centrifugation to separate the layers, the hexane layer is collected for analysis by GC/MS. The concentration of the analyte is determined using an internal standard and reported as μg/collection.

4 Comparison to Combustible Cigarettes

Combustible reference cigarette smoke yield values for either the 1R6F or 3R4F Reference Cigarette (University of Kentucky) were obtained from:

- University of Kentucky Certificate of Analysis (COA) for 1R6F Reference Cigarette (Center for Tobacco Reference Products, 2018),

- Peer-reviewed literature (Forster et al., 2018; Fresquez et al., 2017; Jaccard et al., 2019; Jain et al., 2021; Moldoveanu et al., 2017; Pappas, 2011; Pappas et al., 2014; Schaller et al., 2016; St. Helen et al., 2018; Uchiyama et al., 2018), or
- Sponsored laboratory testing

The primary combustible reference cigarette used for comparison was 1R6F. When available, 1R6F COA values were recorded as the analyte value for comparison. Where 1R6F COA values did not exist, 1R6F data presented by Jaccard et al. (2019), was used. 3R4F values reported by Jaccard et al. (2019), were used if there was no 1R6F data available for the analyte of interest.

For analytes where neither COA values nor Jaccard et al. (2019) values were available for 1R6F or 3R4F, the remaining literature sources and internal dataset(s) were used to record analyte values. If multiple values were available for an analyte of interest using these remaining sources, the lowest concentration value reported was used.

Where BLOD and BLOQ values were available, the value is imputed and displayed as described within the Section 5. All values were normalized on a nicotine basis for both ISO Non-Intense (ISO, 2012) and ISO Intense (ISO, 2018b) smoking regimes.

4.1 1-EOL Yield Calculation

The quantitative targeted analysis yield data used for comparison are presented as 1-EOL collections. 1-EOL collections were normalized to nicotine (Equation 1) and reported as analyte per mg nicotine. Normalization to nicotine allows for direct yield comparison between ENDS and combustible cigarettes.[i]

Equation (S1). Calculation of JUUL2 System 1-EOL ENDS Aerosol Yield Normalized to Nicotine

$$1 - EOL\ Aerosol\ Yield\ Normalized\ to\ Nicotine = \frac{1 - EOL\ Analyte\ Yield}{1 - EOL\ Nicotine\ Yield}$$

For all data, when analyte measurements consisted of a mixture of replicate measurements that were both greater than the LOQ and BLOQ, the numerical mean analyte yield was computed as described in Section 5.2 and reported only if that mean yield exceeded the LOQ.

4.2 Background Subtraction

To mitigate the impact of environmental background during aerosol collection, which can lead to false positives and/or an overestimation of results, laboratory background control (air blank) measurements were performed (Margham et al., 2016). Blank background subtraction was applied to select aerosol sample datasets where applicable. The collection blanks correspond with the samples collected in the same analytical run. The background subtraction approach (Table 2) was applied to the respective batch in which quantifiable air blank levels were measured.

Table S2. Background Subtraction Approach for Aerosol Samples

Condition	Measured Test Result	Reported Test Result
Air blank ≥ Sample	NA	NDFB
Air blank < Sample	Students' t-test if p-value < α	Difference between air blank and sample

	Students’ t-test if p-value ≥ α	NDFB

NA = not applicable; NDFB = not different from blank (i.e., sample ≤ air blank).
Note: “Air blank” is the air blank average test result
Note: Sample is an individual harmful or potentially harmful compound or chemical (i.e., analyte) reported as the measured test result; p-value is the probability of the null hypothesis (α); and α is the null hypothesis

In cases where results for the air blank average value were below the LOD or LOQ, no action was taken. In cases where results for the air blank average value and analyte average value were above the LOQ, and the average air blank value was greater than or equal to the average analyte value, the analyte value was reported as NDFB. In cases where results for the air blank value were non-zero, a statistical analysis was performed using Student’s t-test (unpaired, nonparametric, 2-tailed) to establish whether the sample and the blank results were significantly different ($p<0.05$). If there was no statistically significant difference, the batch result was reported as NDFB. If the background and sample results were statistically different, the difference between the sample and the background was computed (sample mean minus blank mean).

5 Limit of Detection and Quantitation

5.1 Determination of Limit of Detection and Quantitation

The typical process outlined by the contract research organization is as follow, LOQ is equal to the concentration of the lowest calibration standard level used in generating the calibration curve. The lowest calibration standard (LOQ) is prepared and injected 10 times. To be deemed fit for purpose, the LOQ results must meet the typical precision and accuracy acceptance criteria:

- Accuracy of LOQ: 100 ± 15%.
- Percent Relative Standard Deviation of LOQ: ≤ 10%)

The minimum (or method) detection limit (MDL, also referred to as LOD) is defined as the minimum compound concentration that can be measured with a 99 percent confidence level that the concentration is greater than zero. To determine MDL, the approach followed is in general accordance with EPA method 40 CFR 135 Appendix B:

Equation (S2). **Method Detection Limit Calculation**

$$MDL\ Concentration = StDev\ \times Students\ T\ Factor$$

Where:

StDev= Standard Deviation of the 10 replicate (LOQ) injections

Students T Factor = the student’s t-value appropriate for the single-tailed 99th percentile t statistic and a standard deviation estimate with n-1 degrees of freedom.

The MDL determined for the method must be lower than the LOQ. Typical procedure MDL set to ≤ 1/10th the concentration of the lowest calibration standard level.

For working LOD/LOQ, the calculations are as follows:

Equation (S3). **Per Collection LOD and LOQ Calculation**

$$Analyte\ Amount\ \left(\frac{ng}{collection}\right) = \frac{Concentration\ \left(\frac{ng}{mL}\right)\ x\ Diultion\ Factor\ x\ Extraction\ Volume\ (mL)}{Total\ \#\ of\ Devices}$$

If desired, LOD/LOQ concentrations can also be converted to per puff:

Equation (S4). **Per Puff LOD and LOQ Calculation**

$$Analyte\ Amount\ \left(\frac{ng}{puff}\right) = \frac{Concentration\ \left(\frac{ng}{mL}\right)\ x\ Diultion\ Factor\ x\ Extraction\ Volume\ (mL)}{Total\ \#\ of\ Puffs}$$

Note, example calculations nanograms (ng) are shown, however, the results may be expressed in other units (mg, mcg, or pg). Dilution factors may also be used in some methods depending on the sample preparation procedure.

5.2 Approximation of LOD or LOQ Results

Approximation of Per Collection and 1-EOL values required imputation to a numeric value when utilizing datasets that included replicates with analyte levels that were reported BLOD or BLOQ.

The criteria applied for representing LOD or LOQ in a calculation for aerosol targeted analysis (T0 through T12) is summarized in Table 3.

Where present, values with "~" are BLOQ or BLOD. The value shown is the method LOQ/2 for BLOD values or (LOD +LOQ)/2 for BLOQ.

Table S3 **Numeric Imputation of Limit of Detection and Limit of Quantitation Values**

Measured or Literature Value	Applied Calculation (Imputation)	Reported Representation
< LOD	LOD / 2	BLOD [~LOD/2]
LOD ≤ x < LOQ	LOQ + LOD / 2	BLOQ [~(LOQ + LOD)/2]
≥ LOQ	NA	Numeric

BLOD = below the limit of detection; BLOQ = below the limit of quantitation; LOD = limit of detection; LOQ = limit of quantitation; NA = not applicable

6 Aerosol Comparison Reporting Methodology

The highest value from any long-term (LT) timepoint of any applicable stability study is used when comparing the JUUL2 System to a comparator:

- LT Storage, Non-Intense Puffing Regime
- LT Storage, Intense Puffing Regime

Quantifiable values are compared to applicable blanks to determine if the value is NDFB. Not applicable (NA) denotes when a comparator value is not available.

The % Difference value is not comparable (NC) if:

- Both JUUL2 System and comparator are BLOQ or BLOD, or
- If the JUUL2 System aerosol yield was quantifiable and the comparator LOQ is greater than the JUUL2 System quantifiable yield, or
- If the comparator aerosol yield was quantifiable and the JUUL2 System LOQ is greater than the comparator quantifiable yield

If the JUUL2 System aerosol yield was quantifiable and the comparator LOQ is less than the JUUL2 System quantifiable yield, then the value is reported as ↑≥X% (JUUL2 System percent difference greater than or equal to X). If the JUUL2 System aerosol yield was BLOQ and the comparator yield was quantifiable, then the value is reported as ↓≤X% (JUUL2 System percent difference less than or equal to X).

6.1 Percent Difference

The following equation (Equation 2) was used to calculate the percent difference between JUUL2 pod and comparator product analyte yields:

Equation (S5) Calculation of Percent Difference

$$\% \, Difference = \frac{JUUL2 \; System \; Analyte \; Yield \; (normalized \; by \; nicotine)}{Comparator \; Analyte \; Yield \; (normalized \; by \; nicotine)} - 1 \times 100\%$$

7 Stability Trends and Analysis

7.1 Primary Constituents, Organic Acids, and pH

Across non-intense and intense results for all JUUL2 formulations, on average, the nicotine, propylene glycol, glycerol, and menthol (for mentholated formulations), levels remained consistent exhibiting no greater than a 20% change. Benzoic acid measurement variability was observed across select formulations, timepoints, or puff regimes, however, the pH remained stable. On average, water uptake from T0-T12 was elevated under non-intense puffing increasing by 103%, while intense puffing increased by 60%. Notably, formulation aerosols containing higher water concentrations as an ingredient (VT, CM, PM) demonstrated decreased water uptake from T0-T12 compared to formulations with lower water concentrations as an ingredient (AT,RM,SM). Ethylene glycol, diethylene glycol, and propionic acid were BLOD across all formulations and timepoints.

7.2 Thermal Degradants

Across non-intense and intense results for all JUUL2 formulations, select carbonyl compounds (acetaldehyde, acrolein, formaldehyde) and glycidol were found quantifiable at one or more timepoints. Levels of carbonyl compounds (acetaldehyde, acrolein, formaldehyde) appeared formulation specific with consistent, based on expected analytical measurement variability, or increasing trends observed. In general, intense puffing results exhibited elevated levels of carbonyl compounds in comparison to non-intense, likely attributed to the longer puff duration. Glycidol levels for VT, CM, and PM aerosols slightly decreased under non-intense and intense puff regimes, however, remained consistent or slightly increased for AT, RM, and SM aerosols under non-intense and intense puff regimes. Similar to the carbonyl compounds, glycidol intense puffing levels exhibited elevated levels as compared to the non-intense levels. Across formulations, despite the fluctuating levels of thermal degradants (acetaldehyde, acrolein, formaldehyde, and glycidol), average percent reductions to CC ranged from >97% to >98% under non-intense puffing and >95% to >97% under intense puffing. Diacetyl, acetyl propionyl, butyraldehyde, crotonaldehyde, and furfural were BLOD or BLOQ across all formulations and timepoints.

7.3 Esters and Alcohols

Esters and alcohols (1-butanol, benzyl acetate, ethyl acetate, ethyl acetoacetate, isoamyl acetate, isobutyl acetate, and methyl acetate) were only analyzed at T0. The only detectable flavourant was ethyl acetate which is on the proposed additions to the proposed HPHC list (FDA, 2019).

7.4 Nicotine Degradants

β-Nicotyrine, cotinine, myosmine, nicotine-n-oxide, and nornicotine were quantifiable across all six formulations. Relative to measured nicotine concentration, quantifiable nicotine degradants remained below the USP and European Pharmacopeia percent impurity limits (USP/NF, 2020; European Pharmacopoeia, 2020) and also exhibited significant reductions to CC (<95%).

7.5 Metals

Arsenic, beryllium, cadmium, cobalt, copper, gold, lead, nickel, silver, tin and zinc were not measured in the JUUL2 aerosol emissions. Chromium, iron, and selenium were quantifiable in VT, CM, and PM at the following time points: Chromium at T0 in under intense puffing (VT), Iron at T0 under non-intense (CM) and intense puffing (VT, CM), and Selenium at T6 under non-intense (PM) and intense puffing (PM). Further evaluation of quantifiable replicates demonstrated sporadic measurements with concentrations decreasing sequentially suggesting potential carry over. Based on inconsistent quantifiable measurements across timepoints and high replicate to replicate variability and the potential for contamination results for metals in JUUL2 aerosols could not be trended and were deemed equivocal.

7.6 VOCs

The VOCs 1,3-butadiene, acrylonitrile, benzene, isoprene, propylene oxide, and toluene were not quantifiable in the JUUL2 aerosol emissions. Based on a lack of presence at T0, VOC analysis was removed from testing for subsequent timepoints after T0 analysis for VT, CM, and PM and at T3 for AT, RM, and SM.

7.7 TSNAs

NNN was only quantifiable in the RM formulation but demonstrated significant reductions (>99%) compared to levels in CC.
