# Supplementary material for: A Practical Framework for Novel Electronic Nicotine Delivery System Evaluation: Chemical and Toxicological Characterization of JUUL2 Aerosol and Comparison with Reference Cigarettes
Source: Toxics. 2024 Jan 4;12(1):41. doi: 10.3390/toxics12010041 (PMC10820849; doi:10.3390/toxics12010041)
Supplement: Supplementary file 1 [file toxics-12-00041-s001.zip › Supplementary_Material_File S2_Per Collection Limit of Detection and Limit of Quanti.pdf]

Supplementary Materials File S2

1 Per Collection Limits Summary

1.1 Virginia Tobacco 18 mg/mL Non-Intense Per Collection Limit of Detection and Quantification Values

Parameter/ Constituent	Units	T0		T3		T6		T9		T12	
**pH**	NA	NA	NA	NA	NA	NA	NA	NA	NA	NA	NA
**Primary Constituents**		**LOD**	**LOQ**	**LOD**	**LOQ**	**LOD**	**LOQ**	**LOD**	**LOQ**	**LOD**	**LOQ**
**Nicotine**	mg/collection	0.20	2.02	0.20	1.99	0.20	2.03	0.20	2.02	0.20	2.09
**Glycerol**	mg/collection	0.50	12.58	0.50	5.02	0.50	5.03	0.50	5.04	0.50	5.04
**Propylene Glycol**	mg/collection	0.21	2.06	0.21	2.06	0.21	2.08	0.21	2.01	0.21	2.14
**Water**	mg/collection	24.63	24.63	24.60	24.60	24.53	24.53	24.52	24.52	24.43	24.43
**Menthol**	mg/collection	0.16	1.58	0.16	1.61	0.16	1.60	0.16	1.59	0.16	1.64
**Diethylene Glycol**	mg/collection	0.17	1.76	0.16	1.65	0.16	1.67	0.16	1.67	0.17	1.66
**Ethylene Glycol**	mg/collection	0.18	1.77	0.18	1.76	0.18	1.78	0.18	1.79	0.18	1.77
**Carbonyls & Glycidol**		**LOD**	**LOQ**	**LOD**	**LOQ**	**LOD**	**LOQ**	**LOD**	**LOQ**	**LOD**	**LOQ**
**Acetaldehyde**	mcg/collection	0.17	1.13	0.17	1.13	0.20	1.13	0.20	1.13	0.20	1.13
**Acrolein**	mcg/collection	0.11	1.13	0.11	1.13	0.15	1.13	0.15	1.13	0.15	1.13
**Diacetyl**	mcg/collection	0.12	1.14	0.12	1.15	0.11	1.15	0.11	1.12	0.11	1.12
**Formaldehyde**	mcg/collection	0.25	1.13	0.25	1.13	0.38	1.13	0.38	1.13	0.38	1.13
**Glycidol**	mcg/collection	0.003	0.03	0.003	0.03	0.003	0.03	0.003	0.03	0.003	0.03
**Acetyl Propionyl**	mcg/collection	0.11	1.12	0.11	1.13	0.11	1.13	0.11	1.14	0.11	1.14
**Butyraldehyde**	mcg/collection	0.12	1.13	0.12	1.13	0.11	1.13	0.11	1.13	0.11	1.13
**Crotonaldehyde**	mcg/collection	0.11	1.12	0.11	1.12	0.18	1.13	0.18	1.13	0.18	1.11
**Furfural**	mcg/collection	0.15	1.13	0.15	1.13	0.19	1.13	0.19	1.13	0.19	1.13
**Nicotine Degradants & TSNAs**		**LOD**	**LOQ**	**LOD**	**LOQ**	**LOD**	**LOQ**	**LOD**	**LOQ**	**LOD**	**LOQ**
**β-Nicotyrine**	mcg/collection	0.09	0.90	0.09	0.90	0.09	0.90	0.09	0.90	0.09	0.90
**Cotinine**	mcg/collection	0.09	0.90	0.09	0.90	0.09	0.90	0.09	0.90	0.09	0.90

**Myosmine**	mcg/collection	0.09	0.90	0.09	0.90	0.09	0.90	0.09	0.90	0.09	0.90
**Nicotine N Oxide**	mcg/collection	0.31	0.90	0.31	0.90	0.09	0.90	0.09	0.90	0.09	0.90
**Nornicotine**	mcg/collection	0.14	0.90	0.14	0.90	0.09	0.90	0.09	0.90	0.09	0.90
**Anabasine**	mcg/collection	0.09	0.89	0.09	0.91	0.09	0.91	0.09	0.91	0.09	0.91
**Anatabine**	mcg/collection	0.09	0.90	0.09	0.90	0.09	0.90	0.09	0.90	0.09	0.90
**NNK**	mcg/collection	0.003	0.03	NT	NT	NT	NT	NT	NT	NT	NT
**NNN**	mcg/collection	0.003	0.03	NT	NT	NT	NT	NT	NT	NT	NT
**Metals**		**LOD**	**LOQ**	**LOD**	**LOQ**	**LOD**	**LOQ**	**LOD**	**LOQ**	**LOD**	**LOQ**
**Arsenic**	ng/collection	10.00	50.00	10.00	50.00	10.00	50.00	10.00	50.00	10.00	50.00
**Beryllium**	ng/collection	2.20	20.00	2.20	20.00	2.20	20.00	2.20	20.00	2.20	20.00
**Cadmium**	ng/collection	1.00	5.00	1.00	5.00	1.00	5.00	1.00	5.00	1.00	5.00
**Chromium**	ng/collection	10.00	50.00	10.00	50.00	10.00	50.00	10.00	50.00	10.00	50.00
**Cobalt**	ng/collection	0.50	5.00	0.50	5.00	0.50	5.00	0.50	5.00	0.50	5.00
**Copper**	ng/collection	25.00	75.00	25.00	75.00	25.00	75.00	25.00	75.00	25.00	75.00
**Gold**	ng/collection	170.00	200.00	NT	NT	NT	NT	NT	NT	NT	NT
**Iron**	ng/collection	100.00	200.00	100.00	200.00	100.00	200.00	100.00	200.00	100.00	200.00
**Lead**	ng/collection	2.50	10.00	2.50	10.00	2.50	10.00	2.50	10.00	2.50	10.00
**Nickel**	ng/collection	5.00	10.00	5.00	10.00	5.00	10.00	5.00	10.00	5.00	10.00
**Selenium**	ng/collection	3.90	20.00	3.90	20.00	3.90	20.00	3.90	20.00	3.90	20.00
**Silver**	ng/collection	1.00	5.00	1.00	5.00	1.00	5.00	1.00	5.00	1.00	5.00
**Tin**	ng/collection	10.00	50.00	10.00	50.00	10.00	50.00	10.00	50.00	10.00	50.00
**Zinc**	ng/collection	150.00	350.00	150.00	350.00	150.00	350.00	150.00	350.00	150.00	350.00
**Organic Acids**		**LOD**	**LOQ**	**LOD**	**LOQ**	**LOD**	**LOQ**	**LOD**	**LOQ**	**LOD**	**LOQ**
**Benzoic Acid**	mg/collection	0.09	0.88	0.09	0.88	0.06	0.35	0.15	0.88	0.09	0.88
**Propionic Acid**	mcg/collection	3.53	35.31	3.53	35.31	61.89	352.71	3.53	35.31	3.53	35.31
**VOCs**		**LOD**	**LOQ**	**LOD**	**LOQ**	**LOD**	**LOQ**	**LOD**	**LOQ**	**LOD**	**LOQ**
**1,3-Butadiene**	mcg/collection	0.05	0.20	NT	NT	NT	NT	NT	NT	NT	NT
**Acrylonitrile**	mcg/collection	0.04	0.21	NT	NT	NT	NT	NT	NT	NT	NT
**Benzene**	mcg/collection	0.06	0.30	NT	NT	NT	NT	NT	NT	NT	NT

**Isoprene**	mcg/collection	0.08	0.25	NT	NT	NT	NT	NT	NT	NT	NT
**Propylene Oxide**	mcg/collection	0.15	0.85	NT	NT	NT	NT	NT	NT	NT	NT
**Toluene**	mcg/collection	0.09	0.35	NT	NT	NT	NT	NT	NT	NT	NT
**Esters & Alcohols**		**LOD**	**LOQ**	**LOD**	**LOQ**	**LOD**	**LOQ**	**LOD**	**LOQ**	**LOD**	**LOQ**
**1-Butanol**	mcg/collection	1.62	16.20	NT	NT	NT	NT	NT	NT	NT	NT
**Benzyl Acetate**	mcg/collection	2.51	25.12	NT	NT	NT	NT	NT	NT	NT	NT
**Ethyl Acetate**	mcg/collection	2.88	28.76	NT	NT	NT	NT	NT	NT	NT	NT
**Ethyl Acetoacetate**	mcg/collection	2.05	20.54	NT	NT	NT	NT	NT	NT	NT	NT
**Isoamyl Acetate**	mcg/collection	1.75	17.52	NT	NT	NT	NT	NT	NT	NT	NT
**Isobutyl Acetate**	mcg/collection	1.74	17.36	NT	NT	NT	NT	NT	NT	NT	NT
**Methyl Acetate**	mcg/collection	1.87	18.66	NT	NT	NT	NT	NT	NT	NT	NT

1.2 Virginia Tobacco 18 mg/mL Intense Per Collection Limit of Detection and Quantification Values

Parameter/ Constituent	Units	T0		T3		T6		T9		T12	
**pH**	NA	NA	NA	NA	NA	NA	NA	NA	NA	NA	NA
**Primary Constituents**		**LOD**	**LOQ**	**LOD**	**LOQ**	**LOD**	**LOQ**	**LOD**	**LOQ**	**LOD**	**LOQ**
**Nicotine**	mg/collection	0.20	2.02	0.20	1.99	0.20	2.03	0.20	2.02	0.20	2.09
**Glycerol**	mg/collection	0.50	12.58	0.50	5.02	0.50	5.03	0.45	4.53	0.45	4.54
**Propylene Glycol**	mg/collection	0.21	2.06	0.21	2.06	0.21	2.08	0.21	2.01	0.21	2.14
**Water**	mg/collection	24.62	24.68	24.66	24.66	24.46	24.46	24.48	24.48	24.45	24.45
**Menthol**	mg/collection	0.16	1.58	0.16	1.61	0.16	1.60	0.16	1.59	0.16	1.64
**Diethylene Glycol**	mg/collection	0.17	1.76	0.16	1.65	0.16	1.67	0.16	1.67	0.17	1.66
**Ethylene Glycol**	mg/collection	0.18	1.77	0.18	1.76	0.18	1.78	0.18	1.79	0.18	1.77
**Carbonyls & Glycidol**		**LOD**	**LOQ**	**LOD**	**LOQ**	**LOD**	**LOQ**	**LOD**	**LOQ**	**LOD**	**LOQ**
**Acetaldehyde**	mcg/collection	0.20	1.32	0.20	1.32	0.24	1.31	0.24	1.31	0.24	1.32
**Acrolein**	mcg/collection	0.13	1.32	0.13	1.32	0.18	1.32	0.18	1.32	0.18	1.31
**Diacetyl**	mcg/collection	0.14	1.33	0.14	1.34	0.13	1.34	0.13	1.30	0.13	1.30

**Formaldehyde**	mcg/collection	0.29	1.31	0.29	1.31	0.44	1.31	0.44	1.31	0.44	1.32
**Glycidol**	mcg/collection	0.003	0.03	0.003	0.03	0.003	0.03	0.003	0.03	0.003	0.03
**Acetyl Propionyl**	mcg/collection	0.13	1.31	0.13	1.32	0.13	1.32	0.13	1.33	0.13	1.33
**Butyraldehyde**	mcg/collection	0.14	1.32	0.14	1.32	0.13	1.32	0.13	1.30	0.13	1.30
**Crotonaldehyde**	mcg/collection	0.13	1.31	0.13	1.31	0.21	1.32	0.21	1.32	0.21	1.29
**Furfural**	mcg/collection	0.18	1.32	0.18	1.32	0.22	1.32	0.22	1.32	0.22	1.32
**Nicotine Degradants & TSNAs**		**LOD**	**LOQ**	**LOD**	**LOQ**	**LOD**	**LOQ**	**LOD**	**LOQ**	**LOD**	**LOQ**
**β-Nicotyrine**	mcg/collection	0.10	1.05	0.10	1.05	0.11	1.06	0.11	1.06	0.11	1.06
**Cotinine**	mcg/collection	0.10	1.05	0.10	1.05	0.11	1.06	0.11	1.06	0.11	1.06
**Myosmine**	mcg/collection	0.10	1.05	0.10	1.05	0.11	1.05	0.11	1.05	0.11	1.05
**Nicotine N Oxide**	mcg/collection	0.37	1.06	0.37	1.05	0.11	1.05	0.11	1.05	0.11	1.05
**Nornicotine**	mcg/collection	0.17	1.05	0.17	1.05	0.11	1.05	0.11	1.05	0.11	1.05
**Anabasine**	mcg/collection	0.11	1.04	0.11	1.05	0.11	1.06	0.11	1.06	0.11	1.06
**Anatabine**	mcg/collection	0.11	1.06	0.11	1.06	0.11	1.06	0.11	1.06	0.11	1.06
**NNK**	mcg/collection	0.002	0.03	NT	NT	NT	NT	NT	NT	NT	NT
**NNN**	mcg/collection	0.003	0.03	NT	NT	NT	NT	NT	NT	NT	NT
**Metals**		**LOD**	**LOQ**	**LOD**	**LOQ**	**LOD**	**LOQ**	**LOD**	**LOQ**	**LOD**	**LOQ**
**Arsenic**	ng/collection	10.00	50.00	10.00	50.00	10.00	50.00	10.00	50.00	10.00	50.00
**Beryllium**	ng/collection	2.20	20.00	2.20	20.00	2.20	20.00	2.20	20.00	2.20	20.00
**Cadmium**	ng/collection	1.00	5.00	1.00	5.00	1.00	5.00	1.00	5.00	1.00	5.00
**Chromium**	ng/collection	10.00	50.00	10.00	50.00	10.00	50.00	10.00	50.00	10.00	50.00
**Cobalt**	ng/collection	0.50	5.00	0.50	5.00	0.50	5.00	0.50	5.00	0.50	5.00
**Copper**	ng/collection	25.00	75.00	25.00	75.00	25.00	75.00	25.00	75.00	25.00	75.00
**Gold**	ng/collection	170.00	200.00	NT	NT	NT	NT	NT	NT	NT	NT
**Iron**	ng/collection	100.00	200.00	100.00	200.00	100.00	200.00	100.00	200.00	100.00	200.00
**Lead**	ng/collection	2.50	10.00	2.50	10.00	2.50	10.00	2.50	10.00	2.50	10.00
**Nickel**	ng/collection	5.00	10.00	5.00	10.00	5.00	10.00	5.00	10.00	5.00	10.00
**Selenium**	ng/collection	3.90	20.00	3.90	20.00	3.90	20.00	3.90	20.00	3.90	20.00
**Silver**	ng/collection	1.00	5.00	1.00	5.00	1.00	5.00	1.00	5.00	1.00	5.00

**Tin**	ng/collection	10.00	50.00	10.00	50.00	10.00	50.00	10.00	50.00	10.00	50.00
**Zinc**	ng/collection	150.00	350.00	150.00	350.00	150.00	350.00	150.00	350.00	150.00	350.00
**Organic Acids**		**LOD**	**LOQ**	**LOD**	**LOQ**	**LOD**	**LOQ**	**LOD**	**LOQ**	**LOD**	**LOQ**
**Benzoic Acid**	mg/collection	0.09	0.88	0.09	0.88	0.07	0.67	0.16	0.88	0.16	0.88
**Propionic Acid**	mcg/collection	3.53	35.31	3.53	35.31	87.72	670.15	3.53	35.31	3.53	35.31
**VOCs**		**LOD**	**LOQ**	**LOD**	**LOQ**	**LOD**	**LOQ**	**LOD**	**LOQ**	**LOD**	**LOQ**
**1,3-Butadiene**	mcg/collection	0.05	0.20	NT	NT	NT	NT	NT	NT	NT	NT
**Acrylonitrile**	mcg/collection	0.04	0.21	NT	NT	NT	NT	NT	NT	NT	NT
**Benzene**	mcg/collection	0.06	0.30	NT	NT	NT	NT	NT	NT	NT	NT
**Isoprene**	mcg/collection	0.08	0.25	NT	NT	NT	NT	NT	NT	NT	NT
**Propylene Oxide**	mcg/collection	0.15	0.85	NT	NT	NT	NT	NT	NT	NT	NT
**Toluene**	mcg/collection	0.09	0.35	NT	NT	NT	NT	NT	NT	NT	NT
**Esters & Alcohols**		**LOD**	**LOQ**	**LOD**	**LOQ**	**LOD**	**LOQ**	**LOD**	**LOQ**	**LOD**	**LOQ**
**1-Butanol**	mcg/collection	1.62	16.20	NT	NT	NT	NT	NT	NT	NT	NT
**Benzyl Acetate**	mcg/collection	2.51	25.12	NT	NT	NT	NT	NT	NT	NT	NT
**Ethyl Acetate**	mcg/collection	2.88	28.76	NT	NT	NT	NT	NT	NT	NT	NT
**Ethyl Acetoacetate**	mcg/collection	2.05	20.54	NT	NT	NT	NT	NT	NT	NT	NT
**Isoamyl Acetate**	mcg/collection	1.75	17.52	NT	NT	NT	NT	NT	NT	NT	NT
**Isobutyl Acetate**	mcg/collection	1.74	17.36	NT	NT	NT	NT	NT	NT	NT	NT
**Methyl Acetate**	mcg/collection	1.87	18.66	NT	NT	NT	NT	NT	NT	NT	NT

1.3 Crisp Menthol 18 mg/mL Non-Intense Per Collection Limit of Detection and Quantification Values

Parameter/ Constituent	Units	T0		T3		T6		T9		T12	
**pH**	NA	NA	NA	NA	NA	NA	NA	NA	NA	NA	NA
**Primary Constituents**		**LOD**	**LOQ**	**LOD**	**LOQ**	**LOD**	**LOQ**	**LOD**	**LOQ**	**LOD**	**LOQ**
**Nicotine**	mg/collection	0.20	2.02	0.20	1.99	0.20	2.03	0.20	2.02	0.20	2.09
**Glycerol**	mg/collection	0.25	6.29	0.25	2.51	0.25	2.52	0.25	2.52	0.25	2.52

**Propylene Glycol**	mg/collection	0.42	4.12	0.41	4.11	0.41	4.16	0.41	4.02	0.41	4.28
**Water**	mg/collection	24.79	24.85	24.60	24.60	24.52	24.52	24.64	24.64	24.57	24.57
**Menthol**	mg/collection	0.16	1.58	0.16	1.61	0.16	1.60	0.16	1.59	0.16	1.64
**Diethylene Glycol**	mg/collection	0.17	1.76	0.16	1.65	0.16	1.67	0.16	1.67	0.17	1.66
**Ethylene Glycol**	mg/collection	0.18	1.77	0.18	1.76	0.18	1.78	0.18	1.79	0.18	1.77
**Carbonyls & Glycidol**		**LOD**	**LOQ**	**LOD**	**LOQ**	**LOD**	**LOQ**	**LOD**	**LOQ**	**LOD**	**LOQ**
**Acetaldehyde**	mcg/collection	0.17	1.13	0.17	1.13	0.20	1.13	0.20	1.13	0.20	1.13
**Acrolein**	mcg/collection	0.11	1.13	0.11	1.13	0.15	1.13	0.15	1.13	0.15	1.13
**Diacetyl**	mcg/collection	0.12	1.14	0.12	1.15	0.11	1.15	0.11	1.12	0.11	1.12
**Formaldehyde**	mcg/collection	0.25	1.13	0.25	1.13	0.38	1.13	0.38	1.13	0.38	1.13
**Glycidol**	mcg/collection	0.003	0.03	0.003	0.03	0.003	0.03	0.003	0.03	0.003	0.03
**Acetyl Propionyl**	mcg/collection	0.11	1.12	0.11	1.13	0.11	1.13	0.11	1.14	0.11	1.14
**Butyraldehyde**	mcg/collection	0.12	1.13	0.12	1.13	0.11	1.13	0.11	1.13	0.11	1.13
**Crotonaldehyde**	mcg/collection	0.11	1.12	0.11	1.12	0.18	1.13	0.18	1.13	0.18	1.11
**Furfural**	mcg/collection	0.15	1.13	0.15	1.13	0.19	1.13	0.19	1.13	0.19	1.13
**Nicotine Degradants & TSNAs**		**LOD**	**LOQ**	**LOD**	**LOQ**	**LOD**	**LOQ**	**LOD**	**LOQ**	**LOD**	**LOQ**
**β-Nicotyrine**	mcg/collection	0.09	0.90	0.09	0.90	0.09	0.90	0.09	0.90	0.09	0.90
**Cotinine**	mcg/collection	0.09	0.90	0.09	0.90	0.09	0.90	0.09	0.90	0.09	0.90
**Myosmine**	mcg/collection	0.09	0.90	0.09	0.90	0.09	0.90	0.09	0.90	0.09	0.90
**Nicotine N Oxide**	mcg/collection	0.31	0.90	0.31	0.90	0.09	0.90	0.09	0.90	0.09	0.90
**Nornicotine**	mcg/collection	0.14	0.90	0.14	0.90	0.09	0.90	0.09	0.90	0.09	0.90
**Anabasine**	mcg/collection	0.09	0.89	0.09	0.91	0.09	0.91	0.09	0.91	0.09	0.91
**Anatabine**	mcg/collection	0.09	0.90	0.09	0.90	0.09	0.90	0.09	0.90	0.09	0.90
**NNK**	mcg/collection	0.003	0.03	NT	NT	NT	NT	NT	NT	NT	NT
**NNN**	mcg/collection	0.003	0.03	NT	NT	NT	NT	NT	NT	NT	NT
**Metals**		**LOD**	**LOQ**	**LOD**	**LOQ**	**LOD**	**LOQ**	**LOD**	**LOQ**	**LOD**	**LOQ**
**Arsenic**	ng/collection	10.00	50.00	10.00	50.00	10.00	50.00	10.00	50.00	10.00	50.00
**Beryllium**	ng/collection	2.20	20.00	2.20	20.00	2.20	20.00	2.20	20.00	2.20	20.00
**Cadmium**	ng/collection	1.00	5.00	1.00	5.00	1.00	5.00	1.00	5.00	1.00	5.00

**Chromium**	ng/collection	10.00	50.00	10.00	50.00	10.00	50.00	10.00	50.00	10.00	50.00
**Cobalt**	ng/collection	0.50	5.00	0.50	5.00	0.50	5.00	0.50	5.00	0.50	5.00
**Copper**	ng/collection	25.00	75.00	25.00	75.00	25.00	75.00	25.00	75.00	25.00	75.00
**Gold**	ng/collection	170.00	200.00	NT	NT	NT	NT	NT	NT	NT	NT
**Iron**	ng/collection	100.00	200.00	100.00	200.00	100.00	200.00	100.00	200.00	100.00	200.00
**Lead**	ng/collection	2.50	10.00	2.50	10.00	2.50	10.00	2.50	10.00	2.50	10.00
**Nickel**	ng/collection	5.00	10.00	5.00	10.00	5.00	10.00	5.00	10.00	5.00	10.00
**Selenium**	ng/collection	3.90	20.00	3.90	20.00	3.90	20.00	3.90	20.00	3.90	20.00
**Silver**	ng/collection	1.00	5.00	1.00	5.00	1.00	5.00	1.00	5.00	1.00	5.00
**Tin**	ng/collection	10.00	50.00	10.00	50.00	10.00	50.00	10.00	50.00	10.00	50.00
**Zinc**	ng/collection	150.00	350.00	150.00	350.00	150.00	350.00	150.00	350.00	150.00	350.00
**Organic Acids**		**LOD**	**LOQ**	**LOD**	**LOQ**	**LOD**	**LOQ**	**LOD**	**LOQ**	**LOD**	**LOQ**
**Benzoic Acid**	mg/collection	0.09	0.88	0.09	0.88	0.06	0.35	0.09	0.88	0.16	0.88
**Propionic Acid**	mcg/collection	6.19	35.48	6.19	35.27	35.27	352.71	3.53	35.31	3.53	35.31
**VOCs**		**LOD**	**LOQ**	**LOD**	**LOQ**	**LOD**	**LOQ**	**LOD**	**LOQ**	**LOD**	**LOQ**
**1,3-Butadiene**	mcg/collection	0.05	0.20	NT	NT	NT	NT	NT	NT	NT	NT
**Acrylonitrile**	mcg/collection	0.04	0.21	NT	NT	NT	NT	NT	NT	NT	NT
**Benzene**	mcg/collection	0.06	0.30	NT	NT	NT	NT	NT	NT	NT	NT
**Isoprene**	mcg/collection	0.08	0.25	NT	NT	NT	NT	NT	NT	NT	NT
**Propylene Oxide**	mcg/collection	0.15	0.85	NT	NT	NT	NT	NT	NT	NT	NT
**Toluene**	mcg/collection	0.09	0.35	NT	NT	NT	NT	NT	NT	NT	NT
**Esters & Alcohols**		**LOD**	**LOQ**	**LOD**	**LOQ**	**LOD**	**LOQ**	**LOD**	**LOQ**	**LOD**	**LOQ**
**1-Butanol**	mcg/collection	1.62	16.20	NT	NT	NT	NT	NT	NT	NT	NT
**Benzyl Acetate**	mcg/collection	2.51	25.12	NT	NT	NT	NT	NT	NT	NT	NT
**Ethyl Acetate**	mcg/collection	2.88	28.76	NT	NT	NT	NT	NT	NT	NT	NT
**Ethyl Acetoacetate**	mcg/collection	2.05	20.54	NT	NT	NT	NT	NT	NT	NT	NT
**Isoamyl Acetate**	mcg/collection	1.75	17.52	NT	NT	NT	NT	NT	NT	NT	NT
**Isobutyl Acetate**	mcg/collection	1.74	17.36	NT	NT	NT	NT	NT	NT	NT	NT
**Methyl Acetate**	mcg/collection	1.87	18.66	NT	NT	NT	NT	NT	NT	NT	NT

1.4 Crisp Menthol 18 mg/mL Intense Per Collection Limit of Detection and Quantification Values

Parameter/ Constituent	Units	T0		T3		T6		T9		T12	
**pH**	NA	NA	NA	NA	NA	NA	NA	NA	NA	NA	NA
**Primary Constituents**		**LOD**	**LOQ**	**LOD**	**LOQ**	**LOD**	**LOQ**	**LOD**	**LOQ**	**LOD**	**LOQ**
**Nicotine**	mg/collection	0.20	2.02	0.20	1.99	0.20	2.02	0.20	2.02	0.20	2.09
**Glycerol**	mg/collection	0.25	6.29	0.25	2.51	0.25	2.52	0.25	2.52	0.25	2.52
**Propylene Glycol**	mg/collection	0.42	4.12	0.41	4.11	0.41	4.02	0.41	4.02	0.41	4.28
**Water**	mg/collection	24.63	24.63	24.73	24.73	24.64	24.64	24.58	24.58	24.09	24.09
**Menthol**	mg/collection	0.16	1.58	0.16	1.61	0.16	1.59	0.16	1.59	0.16	1.64
**Diethylene Glycol**	mg/collection	0.17	1.76	0.16	1.65	0.16	1.67	0.16	1.67	0.17	1.66
**Ethylene Glycol**	mg/collection	0.18	1.77	0.18	1.76	0.18	1.79	0.18	1.79	0.18	1.77
**Carbonyls & Glycidol**		**LOD**	**LOQ**	**LOD**	**LOQ**	**LOD**	**LOQ**	**LOD**	**LOQ**	**LOD**	**LOQ**
**Acetaldehyde**	mcg/collection	0.20	1.32	0.20	1.32	0.20	1.13	0.24	1.31	0.25	1.32
**Acrolein**	mcg/collection	0.13	1.32	0.13	1.32	0.15	1.13	0.18	1.32	0.38	1.31
**Diacetyl**	mcg/collection	0.14	1.33	0.14	1.34	0.11	1.12	0.13	1.30	0.26	1.30
**Formaldehyde**	mcg/collection	0.29	1.31	0.29	1.31	0.38	1.13	0.44	1.31	0.76	1.32
**Glycidol**	mcg/collection	0.003	0.03	0.003	0.03	0.003	0.03	0.003	0.03	0.003	0.03
**Acetyl Propionyl**	mcg/collection	0.13	1.31	0.13	1.32	0.11	1.14	0.13	1.33	0.18	1.33
**Butyraldehyde**	mcg/collection	0.14	1.32	0.14	1.32	0.11	1.13	0.13	1.30	0.31	1.32
**Crotonaldehyde**	mcg/collection	0.13	1.31	0.13	1.31	0.18	1.13	0.21	1.32	0.37	1.29
**Furfural**	mcg/collection	0.18	1.32	0.18	1.32	0.19	1.13	0.22	1.32	0.30	1.32
**Nicotine Degradants & TSNAs**		**LOD**	**LOQ**	**LOD**	**LOQ**	**LOD**	**LOQ**	**LOD**	**LOQ**	**LOD**	**LOQ**
**β-Nicotyrine**	mcg/collection	0.10	1.05	0.10	1.05	0.09	0.90	0.11	1.06	0.11	1.06
**Cotinine**	mcg/collection	0.10	1.05	0.10	1.05	0.09	0.90	0.11	1.06	0.11	1.06
**Myosmine**	mcg/collection	0.10	1.05	0.10	1.05	0.09	0.90	0.11	1.05	0.11	1.05
**Nicotine N Oxide**	mcg/collection	0.37	1.06	0.37	1.06	0.09	0.90	0.11	1.05	0.11	1.05

**Nornicotine**	mcg/collection	0.17	1.05	0.17	1.05	0.09	0.90	0.11	1.05	0.11	1.05
**Anabasine**	mcg/collection	0.11	1.04	0.11	1.04	0.09	0.91	0.11	1.06	0.11	1.06
**Anatabine**	mcg/collection	0.11	1.06	0.11	1.06	0.09	0.90	0.11	1.06	0.11	1.06
**NNK**	mcg/collection	0.00	0.03	NT	NT	NT	NT	NT	NT	NT	NT
**NNN**	mcg/collection	0.00	0.03	NT	NT	NT	NT	NT	NT	NT	NT
**Metals**		**LOD**	**LOQ**	**LOD**	**LOQ**	**LOD**	**LOQ**	**LOD**	**LOQ**	**LOD**	**LOQ**
**Arsenic**	ng/collection	10.00	50.00	10.00	50.00	10.00	50.00	10.00	50.00	10.00	50.00
**Beryllium**	ng/collection	2.20	20.00	2.20	20.00	2.20	20.00	2.20	20.00	2.20	20.00
**Cadmium**	ng/collection	1.00	5.00	1.00	5.00	1.00	5.00	1.00	5.00	1.00	5.00
**Chromium**	ng/collection	10.00	50.00	10.00	50.00	10.00	50.00	10.00	50.00	10.00	50.00
**Cobalt**	ng/collection	0.50	5.00	0.50	5.00	0.50	5.00	0.50	5.00	0.50	5.00
**Copper**	ng/collection	25.00	75.00	25.00	75.00	25.00	75.00	25.00	75.00	25.00	75.00
**Gold**	ng/collection	170.00	200.00	NT	NT	NT	NT	NT	NT	NT	NT
**Iron**	ng/collection	100.00	200.00	100.00	200.00	100.00	200.00	100.00	200.00	100.00	200.00
**Lead**	ng/collection	2.50	10.00	2.50	10.00	2.50	10.00	2.50	10.00	2.50	10.00
**Nickel**	ng/collection	5.00	10.00	5.00	10.00	5.00	10.00	5.00	10.00	5.00	10.00
**Selenium**	ng/collection	3.90	20.00	3.90	20.00	3.90	20.00	3.90	20.00	3.90	20.00
**Silver**	ng/collection	1.00	5.00	1.00	5.00	1.00	5.00	1.00	5.00	1.00	5.00
**Tin**	ng/collection	10.00	50.00	10.00	50.00	10.00	50.00	10.00	50.00	10.00	50.00
**Zinc**	ng/collection	150.00	350.00	150.00	350.00	150.00	350.00	150.00	350.00	150.00	350.00
**Organic Acids**		**LOD**	**LOQ**	**LOD**	**LOQ**	**LOD**	**LOQ**	**LOD**	**LOQ**	**LOD**	**LOQ**
**Benzoic Acid**	mg/collection	0.09	0.88	0.09	0.88	0.09	0.88	0.09	0.88	0.09	0.88
**Propionic Acid**	mcg/collection	6.19	35.48	4.62	35.27	3.53	35.31	3.53	35.31	3.53	35.31
**VOCs**		**LOD**	**LOQ**	**LOD**	**LOQ**	**LOD**	**LOQ**	**LOD**	**LOQ**	**LOD**	**LOQ**
**1,3-Butadiene**	mcg/collection	5.49E-02	2.00E-01	NT	NT	NT	NT	NT	NT	NT	NT
**Acrylonitrile**	mcg/collection	3.60E-02	2.11E-01	NT	NT	NT	NT	NT	NT	NT	NT
**Benzene**	mcg/collection	6.30E-02	3.00E-01	NT	NT	NT	NT	NT	NT	NT	NT
**Isoprene**	mcg/collection	8.46E-02	2.53E-01	NT	NT	NT	NT	NT	NT	NT	NT
**Propylene Oxide**	mcg/collection	1.46E-01	8.52E-01	NT	NT	NT	NT	NT	NT	NT	NT

**Toluene**	mcg/collection	9.25E-02	3.52E-01	NT	NT	NT	NT	NT	NT	NT	NT
**Esters & Alcohols**		**LOD**	**LOQ**	**LOD**	**LOQ**	**LOD**	**LOQ**	**LOD**	**LOQ**	**LOD**	**LOQ**
**1-Butanol**	mcg/collection	1.62	16.20	NT	NT	NT	NT	NT	NT	NT	NT
**Benzyl Acetate**	mcg/collection	2.51	25.12	NT	NT	NT	NT	NT	NT	NT	NT
**Ethyl Acetate**	mcg/collection	2.88	28.76	NT	NT	NT	NT	NT	NT	NT	NT
**Ethyl Acetoacetate**	mcg/collection	2.05	20.54	NT	NT	NT	NT	NT	NT	NT	NT
**Isoamyl Acetate**	mcg/collection	1.75	17.52	NT	NT	NT	NT	NT	NT	NT	NT
**Isobutyl Acetate**	mcg/collection	1.74	17.36	NT	NT	NT	NT	NT	NT	NT	NT
**Methyl Acetate**	mcg/collection	1.87	18.66	NT	NT	NT	NT	NT	NT	NT	NT

1.5 Polar Menthol 18 mg/mL Non-Intense Per Collection Limit of Detection and Quantification Values

**Parameter/ Constituent**	**Units**	**T0**		**T3**		**T6**		**T9**		**T12**	
**pH**	NA	NA	NA	NA	NA	NA	NA	NA	NA	NA	NA
**Primary Constituents**		**LOD**	**LOQ**	**LOD**	**LOQ**	**LOD**	**LOQ**	**LOD**	**LOQ**	**LOD**	**LOQ**
**Nicotine**	mg/collection	0.20	2.02	0.20	1.99	0.20	2.03	0.20	2.02	0.20	2.09
**Glycerol**	mg/collection	0.25	6.29	0.25	2.51	0.25	2.52	0.25	2.52	0.25	2.52
**Propylene Glycol**	mg/collection	0.42	4.12	0.41	4.11	0.41	4.16	0.41	4.02	0.37	3.85
**Water**	mg/collection	24.64	24.64	24.60	24.60	24.50	24.50	24.62	24.62	24.53	24.53
**Menthol**	mg/collection	0.16	1.58	0.16	1.61	0.16	1.60	0.16	1.59	0.16	1.64
**Diethylene Glycol**	mg/collection	0.17	1.76	0.16	1.65	0.16	1.67	0.16	1.67	0.17	1.66
**Ethylene Glycol**	mg/collection	0.18	1.77	0.18	1.76	0.18	1.78	0.18	1.79	0.18	1.77
**Carbonyls & Glycidol**		**LOD**	**LOQ**	**LOD**	**LOQ**	**LOD**	**LOQ**	**LOD**	**LOQ**	**LOD**	**LOQ**
**Acetaldehyde**	mcg/collection	0.17	1.13	0.17	1.13	0.20	1.13	0.20	1.13	0.20	1.13
**Acrolein**	mcg/collection	0.11	1.13	0.11	1.13	0.15	1.13	0.15	1.13	0.15	1.13
**Diacetyl**	mcg/collection	0.12	1.14	0.12	1.15	0.11	1.15	0.11	1.12	0.11	1.12
**Formaldehyde**	mcg/collection	0.25	1.13	0.25	1.13	0.38	1.13	0.38	1.13	0.38	1.13
**Glycidol**	mcg/collection	0.003	0.03	0.003	0.03	0.003	0.03	0.003	0.03	0.003	0.03

**Acetyl Propionyl**	mcg/collection	0.11	1.12	0.11	1.13	0.11	1.13	0.11	1.14	0.11	1.14
**Butyraldehyde**	mcg/collection	0.12	1.13	0.12	1.13	0.11	1.13	0.11	1.13	0.11	1.13
**Crotonaldehyde**	mcg/collection	0.11	1.12	0.11	1.12	0.18	1.13	0.18	1.13	0.18	1.11
**Furfural**	mcg/collection	0.15	1.13	0.15	1.13	0.19	1.13	0.19	1.13	0.19	1.13
**Nicotine Degradants & TSNAs**		**LOD**	**LOQ**	**LOD**	**LOQ**	**LOD**	**LOQ**	**LOD**	**LOQ**	**LOD**	**LOQ**
**β-Nicotyrine**	mcg/collection	0.09	0.90	0.09	0.90	0.09	0.90	0.09	0.90	0.09	0.90
**Cotinine**	mcg/collection	0.09	0.90	0.09	0.90	0.09	0.90	0.09	0.90	0.09	0.90
**Myosmine**	mcg/collection	0.09	0.90	0.09	0.90	0.09	0.90	0.09	0.90	0.09	0.90
**Nicotine N Oxide**	mcg/collection	0.31	0.90	0.31	0.90	0.09	0.90	0.09	0.90	0.09	0.90
**Nornicotine**	mcg/collection	0.14	0.90	0.14	0.90	0.09	0.90	0.09	0.90	0.09	0.90
**Anabasine**	mcg/collection	0.09	0.89	0.09	0.91	0.09	0.91	0.09	0.91	0.09	0.91
**Anatabine**	mcg/collection	0.09	0.90	0.09	0.90	0.09	0.90	0.09	0.90	0.09	0.90
**NNK**	mcg/collection	0.003	0.03	NT	NT	NT	NT	NT	NT	NT	NT
**NNN**	mcg/collection	0.003	0.03	NT	NT	NT	NT	NT	NT	NT	NT
**Metals**		**LOD**	**LOQ**	**LOD**	**LOQ**	**LOD**	**LOQ**	**LOD**	**LOQ**	**LOD**	**LOQ**
**Arsenic**	ng/collection	10.00	50.00	10.00	50.00	10.00	50.00	10.00	50.00	10.00	50.00
**Beryllium**	ng/collection	2.20	20.00	2.20	20.00	2.20	20.00	2.20	20.00	2.20	20.00
**Cadmium**	ng/collection	1.00	5.00	1.00	5.00	1.00	5.00	1.00	5.00	1.00	5.00
**Chromium**	ng/collection	10.00	50.00	10.00	50.00	10.00	50.00	10.00	50.00	10.00	50.00
**Cobalt**	ng/collection	0.50	5.00	0.50	5.00	0.50	5.00	0.50	5.00	0.50	5.00
**Copper**	ng/collection	25.00	75.00	25.00	75.00	25.00	75.00	25.00	75.00	25.00	75.00
**Gold**	ng/collection	170.00	200.00	NT	NT	NT	NT	NT	NT	NT	NT
**Iron**	ng/collection	100.00	200.00	100.00	200.00	100.00	200.00	100.00	200.00	100.00	200.00
**Lead**	ng/collection	2.50	10.00	2.50	10.00	2.50	10.00	2.50	10.00	2.50	10.00
**Nickel**	ng/collection	5.00	10.00	5.00	10.00	5.00	10.00	5.00	10.00	5.00	10.00
**Selenium**	ng/collection	3.90	20.00	3.90	20.00	3.90	20.00	3.90	20.00	3.90	20.00
**Silver**	ng/collection	1.00	5.00	1.00	5.00	1.00	5.00	1.00	5.00	1.00	5.00
**Tin**	ng/collection	10.00	50.00	10.00	50.00	10.00	50.00	10.00	50.00	10.00	50.00
**Zinc**	ng/collection	150.00	350.00	150.00	350.00	150.00	350.00	150.00	350.00	150.00	350.00

Organic Acids		LOD	LOQ	LOD	LOQ	LOD	LOQ	LOD	LOQ	LOD	LOQ
**Benzoic Acid**	mg/collection	0.09	0.88	0.09	0.88	0.04	0.35	0.09	0.88	0.15	0.88
**Propionic Acid**	mcg/collection	3.53	35.31	3.53	35.31	43.99	352.71	3.53	35.31	6.19	35.31
**VOCs**		**LOD**	**LOQ**	**LOD**	**LOQ**	**LOD**	**LOQ**	**LOD**	**LOQ**	**LOD**	**LOQ**
**1,3-Butadiene**	mcg/collection	0.05	0.20	NT	NT	NT	NT	NT	NT	NT	NT
**Acrylonitrile**	mcg/collection	0.04	0.21	NT	NT	NT	NT	NT	NT	NT	NT
**Benzene**	mcg/collection	0.06	0.30	NT	NT	NT	NT	NT	NT	NT	NT
**Isoprene**	mcg/collection	0.08	0.25	NT	NT	NT	NT	NT	NT	NT	NT
**Propylene Oxide**	mcg/collection	0.15	0.85	NT	NT	NT	NT	NT	NT	NT	NT
**Toluene**	mcg/collection	0.09	0.35	NT	NT	NT	NT	NT	NT	NT	NT
**Esters & Alcohols**		**LOD**	**LOQ**	**LOD**	**LOQ**	**LOD**	**LOQ**	**LOD**	**LOQ**	**LOD**	**LOQ**
**1-Butanol**	mcg/collection	1.62	16.20	NT	NT	NT	NT	NT	NT	NT	NT
**Benzyl Acetate**	mcg/collection	2.51	25.12	NT	NT	NT	NT	NT	NT	NT	NT
**Ethyl Acetate**	mcg/collection	2.88	28.76	NT	NT	NT	NT	NT	NT	NT	NT
**Ethyl Acetoacetate**	mcg/collection	2.05	20.54	NT	NT	NT	NT	NT	NT	NT	NT
**Isoamyl Acetate**	mcg/collection	1.75	17.52	NT	NT	NT	NT	NT	NT	NT	NT
**Isobutyl Acetate**	mcg/collection	1.74	17.36	NT	NT	NT	NT	NT	NT	NT	NT
**Methyl Acetate**	mcg/collection	1.87	18.66	NT	NT	NT	NT	NT	NT	NT	NT

1.6 Polar Menthol 18 mg/mL Intense Per Collection Limit of Detection and Quantification Values

Parameter/ Constituent	Units	T0		T3		T6		T9		T12	
**pH**	NA	NA	NA	NA	NA	NA	NA	NA	NA	NA	NA
**Primary Constituents**		**LOD**	**LOQ**	**LOD**	**LOQ**	**LOD**	**LOQ**	**LOD**	**LOQ**	**LOD**	**LOQ**
**Nicotine**	mg/collection	0.20	2.02	0.20	1.99	0.20	2.03	0.20	2.02	0.20	2.09
**Glycerol**	mg/collection	0.25	6.29	0.25	2.51	0.25	2.52	0.25	2.52	0.25	2.52
**Propylene Glycol**	mg/collection	0.42	4.12	0.41	4.11	0.41	4.16	0.41	4.02	0.41	4.28
**Water**	mg/collection	24.76	24.81	24.54	24.54	24.54	24.54	24.54	24.54	24.16	24.16

**Menthol**	mg/collection	0.16	1.58	0.16	1.61	0.16	1.60	0.16	1.59	0.16	1.64
**Diethylene Glycol**	mg/collection	0.17	1.76	0.16	1.65	0.16	1.67	0.16	1.67	0.17	1.66
**Ethylene Glycol**	mg/collection	0.18	1.77	0.18	1.76	0.18	1.78	0.18	1.79	0.18	1.77
**Carbonyls & Glycidol**		**LOD**	**LOQ**	**LOD**	**LOQ**	**LOD**	**LOQ**	**LOD**	**LOQ**	**LOD**	**LOQ**
**Acetaldehyde**	mcg/collection	0.20	1.32	0.20	1.32	0.24	1.31	0.24	1.31	0.25	1.32
**Acrolein**	mcg/collection	0.13	1.32	0.13	1.32	0.18	1.32	0.18	1.32	0.38	1.31
**Diacetyl**	mcg/collection	0.14	1.33	0.14	1.34	0.13	1.34	0.13	1.30	0.26	1.30
**Formaldehyde**	mcg/collection	0.29	1.31	0.29	1.31	0.44	1.31	0.44	1.31	0.76	1.32
**Glycidol**	mcg/collection	0.003	0.03	0.003	0.03	0.003	0.03	0.003	0.03	0.003	0.03
**Acetyl Propionyl**	mcg/collection	0.13	1.31	0.13	1.32	0.13	1.32	0.13	1.33	0.18	1.33
**Butyraldehyde**	mcg/collection	0.14	1.32	0.14	1.32	0.13	1.32	0.13	1.30	0.31	1.32
**Crotonaldehyde**	mcg/collection	0.13	1.31	0.13	1.31	0.21	1.32	0.21	1.32	0.37	1.29
**Furfural**	mcg/collection	0.18	1.32	0.18	1.32	0.22	1.32	0.22	1.32	0.30	1.32
**Nicotine Degradants & TSNAs**		**LOD**	**LOQ**	**LOD**	**LOQ**	**LOD**	**LOQ**	**LOD**	**LOQ**	**LOD**	**LOQ**
**β-Nicotyrine**	mcg/collection	0.10	1.05	0.10	1.05	0.11	1.06	0.11	1.06	0.11	1.06
**Cotinine**	mcg/collection	0.10	1.05	0.10	1.05	0.11	1.06	0.11	1.06	0.11	1.06
**Myosmine**	mcg/collection	0.10	1.05	0.10	1.05	0.11	1.05	0.11	1.05	0.11	1.05
**Nicotine N Oxide**	mcg/collection	0.37	1.06	0.37	1.06	0.11	1.05	0.11	1.05	0.11	1.05
**Nornicotine**	mcg/collection	0.17	1.05	0.17	1.05	0.11	1.05	0.11	1.05	0.11	1.05
**Anabasine**	mcg/collection	0.11	1.04	0.11	1.04	0.11	1.06	0.11	1.06	0.11	1.06
**Anatabine**	mcg/collection	0.11	1.06	0.11	1.06	0.11	1.06	0.11	1.06	0.11	1.06
**NNK**	mcg/collection	0.002	0.03	NT	NT	NT	NT	NT	NT	NT	NT
**NNN**	mcg/collection	0.003	0.03	NT	NT	NT	NT	NT	NT	NT	NT
**Metals**		**LOD**	**LOQ**	**LOD**	**LOQ**	**LOD**	**LOQ**	**LOD**	**LOQ**	**LOD**	**LOQ**
**Arsenic**	ng/collection	10.00	50.00	10.00	50.00	10.00	50.00	10.00	50.00	10.00	50.00
**Beryllium**	ng/collection	2.20	20.00	2.20	20.00	2.20	20.00	2.20	20.00	2.20	20.00
**Cadmium**	ng/collection	1.00	5.00	1.00	5.00	1.00	5.00	1.00	5.00	1.00	5.00
**Chromium**	ng/collection	10.00	50.00	10.00	50.00	10.00	50.00	10.00	50.00	10.00	50.00
**Cobalt**	ng/collection	0.50	5.00	0.50	5.00	0.50	5.00	0.50	5.00	0.50	5.00

**Copper**	ng/collection	25.00	75.00	25.00	75.00	25.00	75.00	25.00	75.00	25.00	75.00
**Gold**	ng/collection	170.00	200.00	NT	NT	NT	NT	NT	NT	NT	NT
**Iron**	ng/collection	100.00	200.00	100.00	200.00	100.00	200.00	100.00	200.00	100.00	200.00
**Lead**	ng/collection	2.50	10.00	2.50	10.00	2.50	10.00	2.50	10.00	2.50	10.00
**Nickel**	ng/collection	5.00	10.00	5.00	10.00	5.00	10.00	5.00	10.00	5.00	10.00
**Selenium**	ng/collection	3.90	20.00	3.90	20.00	3.90	20.00	3.90	20.00	3.90	20.00
**Silver**	ng/collection	1.00	5.00	1.00	5.00	1.00	5.00	1.00	5.00	1.00	5.00
**Tin**	ng/collection	10.00	50.00	10.00	50.00	10.00	50.00	10.00	50.00	10.00	50.00
**Zinc**	ng/collection	150.00	350.00	150.00	350.00	150.00	350.00	150.00	350.00	150.00	350.00
**Organic Acids**		**LOD**	**LOQ**	**LOD**	**LOQ**	**LOD**	**LOQ**	**LOD**	**LOQ**	**LOD**	**LOQ**
**Benzoic Acid**	mg/collection	0.09	0.88	0.09	0.88	0.06	0.35	0.09	0.88	0.15	0.88
**Propionic Acid**	mcg/collection	3.53	35.31	3.53	35.31	61.89	352.71	3.53	35.31	6.19	35.31
**VOCs**		**LOD**	**LOQ**	**LOD**	**LOQ**	**LOD**	**LOQ**	**LOD**	**LOQ**	**LOD**	**LOQ**
**1,3-Butadiene**	mcg/collection	0.05	0.20	NT	NT	NT	NT	NT	NT	NT	NT
**Acrylonitrile**	mcg/collection	0.04	0.21	NT	NT	NT	NT	NT	NT	NT	NT
**Benzene**	mcg/collection	0.06	0.30	NT	NT	NT	NT	NT	NT	NT	NT
**Isoprene**	mcg/collection	0.08	0.25	NT	NT	NT	NT	NT	NT	NT	NT
**Propylene Oxide**	mcg/collection	0.15	0.85	NT	NT	NT	NT	NT	NT	NT	NT
**Toluene**	mcg/collection	0.09	0.35	NT	NT	NT	NT	NT	NT	NT	NT
**Esters & Alcohols**		**LOD**	**LOQ**	**LOD**	**LOQ**	**LOD**	**LOQ**	**LOD**	**LOQ**	**LOD**	**LOQ**
**1-Butanol**	mcg/collection	1.62	16.20	NT	NT	NT	NT	NT	NT	NT	NT
**Benzyl Acetate**	mcg/collection	2.51	25.12	NT	NT	NT	NT	NT	NT	NT	NT
**Ethyl Acetate**	mcg/collection	2.88	28.76	NT	NT	NT	NT	NT	NT	NT	NT
**Ethyl Acetoacetate**	mcg/collection	2.05	20.54	NT	NT	NT	NT	NT	NT	NT	NT
**Isoamyl Acetate**	mcg/collection	1.75	17.52	NT	NT	NT	NT	NT	NT	NT	NT
**Isobutyl Acetate**	mcg/collection	1.74	17.36	NT	NT	NT	NT	NT	NT	NT	NT
**Methyl Acetate**	mcg/collection	1.87	18.66	NT	NT	NT	NT	NT	NT	NT	NT

1.7 Autumn Tobacco 18 mg/mL Non-Intense Per Collection Limit of Detection and Quantification Values

Parameter/ Constituent	Units	T0		T3		T6		T9		T12	
**pH**	NA	NA	NA	NA	NA	NA	NA	NA	NA	NA	NA
**Primary Constituents**		**LOD**	**LOQ**	**LOD**	**LOQ**	**LOD**	**LOQ**	**LOD**	**LOQ**	**LOD**	**LOQ**
**Nicotine**	mg/collection	0.15	1.50	0.15	1.50	0.05	0.45	0.05	0.45	0.23	2.25
**Glycerol**	mg/collection	1.25	12.54	1.25	12.63	0.38	3.78	0.59	5.90	1.88	18.96
**Propylene Glycol**	mg/collection	0.40	4.03	0.40	4.01	0.29	2.93	0.60	6.02	0.60	6.02
**Water**	mg/collection	4.63	9.97	4.63	9.96	1.39	3.08	1.39	3.01	6.95	15.10
**Menthol**	mg/collection	0.05	0.48	0.05	0.48	0.01	0.14	0.01	0.14	0.07	0.74
**Diethylene Glycol**	mg/collection	0.04	0.24	0.04	0.28	0.01	0.08	0.01	0.08	0.07	0.41
**Ethylene Glycol**	mg/collection	0.03	0.25	0.03	0.25	0.01	0.07	0.01	0.08	0.05	0.46
**Carbonyls & Glycidol**		**LOD**	**LOQ**	**LOD**	**LOQ**	**LOD**	**LOQ**	**LOD**	**LOQ**	**LOD**	**LOQ**
**Acetaldehyde**	mcg/collection	0.20	1.13	0.20	1.13	0.20	1.13	0.20	1.13	0.19	1.13
**Acrolein**	mcg/collection	0.15	1.13	0.15	1.13	0.15	1.13	0.15	1.13	0.14	1.13
**Diacetyl**	mcg/collection	0.11	1.15	0.11	1.12	0.11	1.12	0.11	1.13	0.16	1.13
**Formaldehyde**	mcg/collection	0.38	1.13	0.38	1.13	0.38	1.13	0.38	1.13	0.15	1.13
**Glycidol**	mcg/collection	0.003	0.03	0.003	0.03	0.003	0.03	0.003	0.03	0.005	0.05
**Acetyl Propionyl**	mcg/collection	0.11	1.13	0.11	1.14	0.11	1.14	0.11	1.14	0.15	1.14
**Butyraldehyde**	mcg/collection	0.11	1.13	0.11	1.11	0.11	1.13	0.11	1.13	0.12	1.13
**Crotonaldehyde**	mcg/collection	0.18	1.11	0.18	1.13	0.18	1.13	0.18	1.13	0.15	1.13
**Furfural**	mcg/collection	0.19	1.13	0.19	1.13	0.19	1.13	0.19	1.13	0.21	1.13
**Nicotine Degradants & TSNAs**		**LOD**	**LOQ**	**LOD**	**LOQ**	**LOD**	**LOQ**	**LOD**	**LOQ**	**LOD**	**LOQ**
**β-Nicotyrine**	mcg/collection	0.09	0.90	0.09	0.90	0.09	0.90	0.09	0.89	0.09	0.89
**Cotinine**	mcg/collection	0.09	0.90	0.09	0.90	0.09	0.90	0.09	0.90	0.19	0.90
**Myosmine**	mcg/collection	0.09	0.90	0.09	0.90	0.09	0.90	0.09	0.91	0.09	0.91
**Nicotine N Oxide**	mcg/collection	0.09	0.90	0.09	0.90	0.09	0.90	0.09	0.89	0.22	0.89
**Nornicotine**	mcg/collection	0.09	0.90	0.09	0.90	0.09	0.90	0.09	0.90	0.23	0.90
**Anabasine**	mcg/collection	0.09	0.91	0.10	0.91	0.09	0.91	0.09	0.91	0.13	0.91
**Anatabine**	mcg/collection	0.09	0.90	0.09	0.90	0.09	0.90	0.09	0.91	0.11	0.91

**NNK**	mcg/collection	0.001	0.003	0.001	0.003	NT	NT	NT	NT	NT	NT
**NNN**	mcg/collection	0.001	0.003	0.001	0.003	NT	NT	NT	NT	NT	NT
**Metals**		**LOD**	**LOQ**	**LOD**	**LOQ**	**LOD**	**LOQ**	**LOD**	**LOQ**	**LOD**	**LOQ**
**Arsenic**	ng/collection	10.00	50.00	10.00	50.00	10.00	50.00	10.00	50.00	10.00	50.00
**Beryllium**	ng/collection	2.20	20.00	2.20	20.00	2.20	20.00	2.20	20.00	2.20	20.00
**Cadmium**	ng/collection	1.00	5.00	1.00	5.00	1.00	5.00	1.00	5.00	1.00	5.00
**Chromium**	ng/collection	10.00	50.00	10.00	50.00	10.00	50.00	10.00	50.00	10.00	50.00
**Cobalt**	ng/collection	0.50	5.00	0.50	5.00	0.50	5.00	0.50	5.00	0.50	5.00
**Copper**	ng/collection	25.00	75.00	25.00	75.00	25.00	75.00	25.00	75.00	25.00	75.00
**Gold**	ng/collection	170.00	200.00	170.00	200.20	170.00	200.00	170.00	200.00	170.00	200.00
**Iron**	ng/collection	100.00	200.00	100.00	200.00	100.00	200.00	100.00	200.00	100.00	200.00
**Lead**	ng/collection	2.50	10.00	2.50	10.00	2.50	10.00	2.50	10.00	2.50	10.00
**Nickel**	ng/collection	5.00	10.00	5.00	10.00	5.00	10.00	5.00	10.00	5.00	10.00
**Selenium**	ng/collection	3.90	20.00	3.90	20.00	3.90	20.00	3.90	20.00	3.90	20.00
**Silver**	ng/collection	1.00	5.00	1.00	5.00	1.00	5.00	1.00	5.00	1.00	5.00
**Tin**	ng/collection	10.00	50.00	10.00	50.00	10.00	50.00	10.00	50.00	10.00	50.00
**Zinc**	ng/collection	150.00	350.00	150.00	350.00	150.00	350.00	150.00	350.00	150.00	350.00
**Organic Acids**		**LOD**	**LOQ**	**LOD**	**LOQ**	**LOD**	**LOQ**	**LOD**	**LOQ**	**LOD**	**LOQ**
**Benzoic Acid**	mg/collection	0.06	0.35	0.06	0.35	0.04	0.35	0.06	0.35	0.07	0.70
**Propionic Acid**	mcg/collection	35.27	352.71	61.89	354.83	35.27	354.83	35.27	354.83	70.61	706.10
**VOCs**		**LOD**	**LOQ**	**LOD**	**LOQ**	**LOD**	**LOQ**	**LOD**	**LOQ**	**LOD**	**LOQ**
**1,3-butadiene**	mcg/collection	0.05	0.20	0.07	0.20	NT	NT	NT	NT	NT	NT
**Acrylonitrile**	mcg/collection	0.04	0.21	0.02	0.20	NT	NT	NT	NT	NT	NT
**Benzene**	mcg/collection	0.06	0.31	0.06	0.31	NT	NT	NT	NT	NT	NT
**Isoprene**	mcg/collection	0.08	0.27	0.06	0.27	NT	NT	NT	NT	NT	NT
**Propylene Oxide**	mcg/collection	0.15	0.87	0.23	0.87	NT	NT	NT	NT	NT	NT
**Toluene**	mcg/collection	0.09	0.35	0.04	0.35	NT	NT	NT	NT	NT	NT
**Esters & Alcohols**		**LOD**	**LOQ**	**LOD**	**LOQ**	**LOD**	**LOQ**	**LOD**	**LOQ**	**LOD**	**LOQ**
**1-Butanol**	mcg/collection	1.91	19.47	1.91	19.71	NT	NT	NT	NT	NT	NT

**Benzyl Acetate**	mcg/collection	2.52	25.22	2.52	25.17	NT	NT	NT	NT	NT	NT
**Ethyl Acetate**	mcg/collection	2.00	20.48	2.00	20.24	NT	NT	NT	NT	NT	NT
**Ethyl Acetoacetate**	mcg/collection	3.90	27.83	3.90	24.81	NT	NT	NT	NT	NT	NT
**Isoamyl Acetate**	mcg/collection	2.03	20.78	2.03	20.83	NT	NT	NT	NT	NT	NT
**Isobutyl Acetate**	mcg/collection	2.02	20.59	2.02	20.50	NT	NT	NT	NT	NT	NT
**Methyl Acetate**	mcg/collection	2.09	21.60	2.09	21.07	NT	NT	NT	NT	NT	NT

1.8 Autumn Tobacco 18 mg/mL Intense Per Collection Limit of Detection and Quantification Values

**Parameter/ Constituent**	**Units**	**T0**		**T3**		**T6**		**T9**		**T12**	
**pH**	NA	NA	NA	NA	NA	NA	NA	NA	NA	NA	NA
**Primary Constituents**		**LOD**	**LOQ**	**LOD**	**LOQ**	**LOD**	**LOQ**	**LOD**	**LOQ**	**LOD**	**LOQ**
**Nicotine**	mg/collection	0.15	1.50	0.15	1.50	0.05	0.45	0.05	0.45	0.23	2.25
**Glycerol**	mg/collection	1.25	12.54	1.25	12.63	0.38	3.78	0.38	3.75	1.88	18.96
**Propylene Glycol**	mg/collection	0.40	4.03	0.40	4.01	0.36	3.62	0.53	5.33	0.60	6.02
**Water**	mg/collection	4.63	9.97	4.63	9.96	1.39	3.08	1.39	3.01	6.95	15.10
**Menthol**	mg/collection	0.05	0.48	0.05	0.48	0.01	0.14	0.01	0.14	0.07	0.74
**Diethylene Glycol**	mg/collection	0.04	0.24	0.04	0.26	0.01	0.08	0.01	0.08	0.07	0.41
**Ethylene Glycol**	mg/collection	0.03	0.25	0.03	0.27	0.01	0.07	0.01	0.08	0.05	0.46
**Carbonyls & Glycidol**		**LOD**	**LOQ**	**LOD**	**LOQ**	**LOD**	**LOQ**	**LOD**	**LOQ**	**LOD**	**LOQ**
**Acetaldehyde**	mcg/collection	0.24	1.32	0.24	1.31	0.20	1.13	0.24	1.32	0.22	1.32
**Acrolein**	mcg/collection	0.18	1.31	0.18	1.32	0.15	1.13	0.18	1.32	0.16	1.32
**Diacetyl**	mcg/collection	0.13	1.34	0.13	1.30	0.11	1.12	0.13	1.32	0.18	1.32
**Formaldehyde**	mcg/collection	0.44	1.32	0.44	1.31	0.38	1.13	0.44	1.32	0.17	1.31
**Glycidol**	mcg/collection	0.004	0.03	0.003	0.03	0.003	0.03	0.003	0.03	0.005	0.05
**Acetyl Propionyl**	mcg/collection	0.13	1.32	0.13	1.33	0.11	1.14	0.13	1.33	0.18	1.33
**Butyraldehyde**	mcg/collection	0.13	1.32	0.13	1.30	0.11	1.13	0.13	1.32	0.14	1.32
**Crotonaldehyde**	mcg/collection	0.21	1.29	0.21	1.32	0.18	1.13	0.21	1.32	0.18	1.31

**Furfural**	mcg/collection	0.22	1.32	0.22	1.32	0.19	1.13	0.22	1.32	0.25	1.32
**Nicotine Degradants & TSNAs**		**LOD**	**LOQ**	**LOD**	**LOQ**	**LOD**	**LOQ**	**LOD**	**LOQ**	**LOD**	**LOQ**
**β-Nicotyrine**	mcg/collection	0.11	1.06	0.10	1.06	0.11	1.06	0.11	1.04	0.10	1.04
**Cotinine**	mcg/collection	0.11	1.06	0.10	1.06	0.11	1.06	0.11	1.05	0.22	1.05
**Myosmine**	mcg/collection	0.11	1.05	0.10	1.05	0.11	1.05	0.11	1.06	0.11	1.06
**Nicotine N Oxide**	mcg/collection	0.11	1.05	0.10	1.05	0.11	1.05	0.11	1.04	0.26	1.04
**Nornicotine**	mcg/collection	0.11	1.05	0.10	1.05	0.11	1.05	0.11	1.04	0.27	1.04
**Anabasine**	mcg/collection	0.11	1.06	0.12	1.06	0.11	1.06	0.11	1.06	0.15	1.06
**Anatabine**	mcg/collection	0.11	1.06	0.11	1.06	0.11	1.06	0.11	1.06	0.12	1.06
**NNK**	mcg/collection	0.001	0.003	0.001	0.003	NT	NT	NT	NT	NT	NT
**NNN**	mcg/collection	0.001	0.003	0.001	0.003	NT	NT	NT	NT	NT	NT
**Metals**		**LOD**	**LOQ**	**LOD**	**LOQ**	**LOD**	**LOQ**	**LOD**	**LOQ**	**LOD**	**LOQ**
**Arsenic**	ng/collection	10.00	50.00	10.00	50.00	10.00	50.00	10.00	50.00	10.00	50.00
**Beryllium**	ng/collection	2.20	20.00	2.20	20.00	2.20	20.00	2.20	20.00	2.20	20.00
**Cadmium**	ng/collection	1.00	5.00	1.00	5.00	1.00	5.00	1.00	5.00	1.00	5.00
**Chromium**	ng/collection	10.00	50.00	10.00	50.00	10.00	50.00	10.00	50.00	10.00	50.00
**Cobalt**	ng/collection	0.50	5.00	0.50	5.00	0.50	5.00	0.50	5.00	0.50	5.00
**Copper**	ng/collection	25.00	75.00	25.00	75.00	25.00	75.00	25.00	75.00	25.00	75.00
**Gold**	ng/collection	170.00	200.00	170.00	200.20	170.00	200.00	170.00	200.00	170.00	200.00
**Iron**	ng/collection	100.00	200.00	100.00	200.00	100.00	200.00	100.00	200.00	100.00	200.00
**Lead**	ng/collection	2.50	10.00	2.50	10.00	2.50	10.00	2.50	10.00	2.50	10.00
**Nickel**	ng/collection	5.00	10.00	5.00	10.00	5.00	10.00	5.00	10.00	5.00	10.00
**Selenium**	ng/collection	3.90	20.00	3.90	20.00	3.90	20.00	3.90	20.00	3.90	20.00
**Silver**	ng/collection	1.00	5.00	1.00	5.00	1.00	5.00	1.00	5.00	1.00	5.00
**Tin**	ng/collection	10.00	50.00	10.00	50.00	10.00	50.00	10.00	50.00	10.00	50.00
**Zinc**	ng/collection	150.00	350.00	150.00	350.00	150.00	350.00	150.00	350.00	150.00	350.00
**Organic Acids**		**LOD**	**LOQ**	**LOD**	**LOQ**	**LOD**	**LOQ**	**LOD**	**LOQ**	**LOD**	**LOQ**
**Benzoic Acid**	mg/collection	0.06	0.35	0.06	0.35	0.06	0.35	0.04	0.35	0.07	0.70
**Propionic Acid**	mcg/collection	35.27	352.71	61.89	354.83	61.89	354.83	35.31	354.83	70.61	706.10

VOCs		LOD	LOQ	LOD	LOQ	LOD	LOQ	LOD	LOQ	LOD	LOQ
**1,3-butadiene**	mcg/collection	0.05	0.20	0.05	0.20	NT	NT	NT	NT	NT	NT
**Acrylonitrile**	mcg/collection	0.04	0.21	0.04	0.21	NT	NT	NT	NT	NT	NT
**Benzene**	mcg/collection	0.06	0.31	0.06	0.31	NT	NT	NT	NT	NT	NT
**Isoprene**	mcg/collection	0.08	0.27	0.08	0.27	NT	NT	NT	NT	NT	NT
**Propylene Oxide**	mcg/collection	0.15	0.88	0.15	0.87	NT	NT	NT	NT	NT	NT
**Toluene**	mcg/collection	0.09	0.35	0.09	0.35	NT	NT	NT	NT	NT	NT
**Esters & Alcohols**		**LOD**	**LOQ**	**LOD**	**LOQ**	**LOD**	**LOQ**	**LOD**	**LOQ**	**LOD**	**LOQ**
**1-Butanol**	mcg/collection	1.91	19.47	1.91	19.66	NT	NT	NT	NT	NT	NT
**Benzyl Acetate**	mcg/collection	2.52	25.22	2.52	25.65	NT	NT	NT	NT	NT	NT
**Ethyl Acetate**	mcg/collection	2.00	20.48	2.00	20.38	NT	NT	NT	NT	NT	NT
**Ethyl Acetoacetate**	mcg/collection	3.90	27.83	3.90	25.01	NT	NT	NT	NT	NT	NT
**Isoamyl Acetate**	mcg/collection	2.03	20.78	2.03	21.02	NT	NT	NT	NT	NT	NT
**Isobutyl Acetate**	mcg/collection	2.02	20.59	2.02	20.78	NT	NT	NT	NT	NT	NT
**Methyl Acetate**	mcg/collection	2.09	21.60	2.09	21.98	NT	NT	NT	NT	NT	NT

1.9 Ruby Menthol 18 mg/mL Non-Intense Per Collection Limit of Detection and Quantification Values

Parameter/ Constituent	Units	T0		T3		T6		T9		T12	
**pH**	NA	NA	NA	NA	NA	NA	NA	NA	NA	NA	NA
**Primary Constituents**		**LOD**	**LOQ**	**LOD**	**LOQ**	**LOD**	**LOQ**	**LOD**	**LOQ**	**LOD**	**LOQ**
**Nicotine**	mg/collection	0.15	1.50	0.15	1.50	0.05	0.45	0.05	0.45	0.23	2.25
**Glycerol**	mg/collection	1.25	12.54	1.25	12.63	0.59	5.93	1.02	10.19	1.88	18.96
**Propylene Glycol**	mg/collection	0.40	4.03	0.40	4.01	0.24	2.41	0.60	6.02	0.60	6.02
**Water**	mg/collection	4.63	9.97	4.63	9.96	1.39	3.08	1.39	3.01	6.95	15.10
**Menthol**	mg/collection	0.05	0.48	0.05	0.48	0.01	0.14	0.01	0.14	0.07	0.74
**Diethylene Glycol**	mg/collection	0.04	0.24	0.04	0.28	0.01	0.08	0.01	0.08	0.07	0.41
**Ethylene Glycol**	mg/collection	0.03	0.25	0.03	0.25	0.01	0.07	0.01	0.08	0.05	0.46

Carbonyls & Glycidol		LOD	LOQ	LOD	LOQ	LOD	LOQ	LOD	LOQ	LOD	LOQ
**Acetaldehyde**	mcg/collection	0.20	1.13	0.20	1.13	0.20	1.13	0.20	1.13	0.19	1.13
**Acrolein**	mcg/collection	0.15	1.13	0.15	1.13	0.15	1.13	0.15	1.13	0.14	1.13
**Diacetyl**	mcg/collection	0.11	1.15	0.11	1.12	0.11	1.12	0.11	1.13	0.16	1.13
**Formaldehyde**	mcg/collection	0.38	1.13	0.38	1.13	0.38	1.13	0.38	1.13	0.15	1.13
**Glycidol**	mcg/collection	0.003	0.03	0.003	0.03	0.003	0.03	0.003	0.03	0.005	0.05
**Acetyl Propionyl**	mcg/collection	0.11	1.13	0.11	1.14	0.11	1.14	0.11	1.14	0.15	1.14
**Butyraldehyde**	mcg/collection	0.11	1.13	0.11	1.11	0.11	1.13	0.11	1.13	0.12	1.13
**Crotonaldehyde**	mcg/collection	0.18	1.11	0.18	1.13	0.18	1.13	0.18	1.13	0.15	1.13
**Furfural**	mcg/collection	0.19	1.13	0.19	1.13	0.19	1.13	0.19	1.13	0.21	1.13
**Nicotine Degradants & TSNAs**		**LOD**	**LOQ**	**LOD**	**LOQ**	**LOD**	**LOQ**	**LOD**	**LOQ**	**LOD**	**LOQ**
**β-Nicotyrine**	mcg/collection	0.09	0.90	0.09	0.90	0.09	0.90	0.09	0.89	0.09	0.89
**Cotinine**	mcg/collection	0.09	0.90	0.09	0.90	0.09	0.90	0.09	0.90	0.19	0.90
**Myosmine**	mcg/collection	0.09	0.90	0.09	0.90	0.09	0.90	0.09	0.91	0.09	0.91
**Nicotine N Oxide**	mcg/collection	0.09	0.90	0.09	0.90	0.09	0.90	0.09	0.89	0.22	0.89
**Nornicotine**	mcg/collection	0.09	0.90	0.09	0.90	0.09	0.90	0.09	0.90	0.23	0.90
**Anabasine**	mcg/collection	0.09	0.91	0.10	0.91	0.09	0.91	0.09	0.91	0.13	0.91
**Anatabine**	mcg/collection	0.09	0.90	0.09	0.90	0.09	0.90	0.09	0.91	0.11	0.91
**NNK**	mcg/collection	0.001	0.003	0.001	0.003	NT	NT	NT	NT	NT	NT
**NNN**	mcg/collection	0.001	0.003	0.001	0.003	NT	NT	NT	NT	NT	NT
**Metals**		**LOD**	**LOQ**	**LOD**	**LOQ**	**LOD**	**LOQ**	**LOD**	**LOQ**	**LOD**	**LOQ**
**Arsenic**	ng/collection	10.00	50.00	10.00	50.00	10.00	50.00	10.00	50.00	10.00	50.00
**Beryllium**	ng/collection	2.20	20.00	2.20	20.00	2.20	20.00	2.20	20.00	2.20	20.00
**Cadmium**	ng/collection	1.00	5.00	1.00	5.00	1.00	5.00	1.00	5.00	1.00	5.00
**Chromium**	ng/collection	10.00	50.00	10.00	50.00	10.00	50.00	10.00	50.00	10.00	50.00
**Cobalt**	ng/collection	0.50	5.00	0.50	5.00	0.50	5.00	0.50	5.00	0.50	5.00
**Copper**	ng/collection	25.00	75.00	25.00	75.00	25.00	75.00	25.00	75.00	25.00	75.00
**Gold**	ng/collection	170.00	200.00	170.00	200.20	170.00	200.00	170.00	200.00	170.00	200.00
**Iron**	ng/collection	100.00	200.00	100.00	200.00	100.00	200.00	100.00	200.00	100.00	200.00

**Lead**	ng/collection	2.50	10.00	2.50	10.00	2.50	10.00	2.50	10.00	2.50	10.00
**Nickel**	ng/collection	5.00	10.00	5.00	10.00	5.00	10.00	5.00	10.00	5.00	10.00
**Selenium**	ng/collection	3.90	20.00	3.90	20.00	3.90	20.00	3.90	20.00	3.90	20.00
**Silver**	ng/collection	1.00	5.00	1.00	5.00	1.00	5.00	1.00	5.00	1.00	5.00
**Tin**	ng/collection	10.00	50.00	10.00	50.00	10.00	50.00	10.00	50.00	10.00	50.00
**Zinc**	ng/collection	150.00	350.00	150.00	350.00	150.00	350.00	150.00	350.00	150.00	350.00
**Organic Acids**		**LOD**	**LOQ**	**LOD**	**LOQ**	**LOD**	**LOQ**	**LOD**	**LOQ**	**LOD**	**LOQ**
**Benzoic Acid**	mg/collection	0.04	0.35	0.06	0.35	0.04	0.35	0.06	0.35	0.07	0.70
**Propionic Acid**	mcg/collection	46.17	352.71	61.89	354.83	35.27	354.83	35.27	354.83	70.61	706.10
**VOCs**		**LOD**	**LOQ**	**LOD**	**LOQ**	**LOD**	**LOQ**	**LOD**	**LOQ**	**LOD**	**LOQ**
**1,3-butadiene**	mcg/collection	0.05	0.20	0.07	0.20	NT	NT	NT	NT	NT	NT
**Acrylonitrile**	mcg/collection	0.04	0.21	0.02	0.20	NT	NT	NT	NT	NT	NT
**Benzene**	mcg/collection	0.06	0.31	0.06	0.31	NT	NT	NT	NT	NT	NT
**Isoprene**	mcg/collection	0.08	0.27	0.06	0.27	NT	NT	NT	NT	NT	NT
**Propylene Oxide**	mcg/collection	0.15	0.87	0.23	0.87	NT	NT	NT	NT	NT	NT
**Toluene**	mcg/collection	0.09	0.35	0.04	0.35	NT	NT	NT	NT	NT	NT
**Esters & Alcohols**		**LOD**	**LOQ**	**LOD**	**LOQ**	**LOD**	**LOQ**	**LOD**	**LOQ**	**LOD**	**LOQ**
**1-Butanol**	mcg/collection	1.91	19.47	1.91	19.71	NT	NT	NT	NT	NT	NT
**Benzyl Acetate**	mcg/collection	2.52	25.22	2.52	25.17	NT	NT	NT	NT	NT	NT
**Ethyl Acetate**	mcg/collection	2.00	20.48	2.00	20.24	NT	NT	NT	NT	NT	NT
**Ethyl Acetoacetate**	mcg/collection	3.90	27.83	3.90	24.81	NT	NT	NT	NT	NT	NT
**Isoamyl Acetate**	mcg/collection	2.03	20.78	2.03	20.83	NT	NT	NT	NT	NT	NT
**Isobutyl Acetate**	mcg/collection	2.02	20.59	2.02	20.50	NT	NT	NT	NT	NT	NT
**Methyl Acetate**	mcg/collection	2.09	21.60	2.09	21.07	NT	NT	NT	NT	NT	NT

1.10 Ruby Menthol 18 mg/mL Intense Per Collection Limit of Detection and Quantification Values

Parameter/ Constituent	Units	T0	T3	T6	T9	T12

**pH**	NA	NA	NA	NA	NA	NA	NA	NA	NA	NA	NA
**Primary Constituents**		**LOD**	**LOQ**	**LOD**	**LOQ**	**LOD**	**LOQ**	**LOD**	**LOQ**	**LOD**	**LOQ**
**Nicotine**	mg/collection	0.15	1.50	0.15	1.50	0.05	0.45	0.05	0.45	0.23	2.25
**Glycerol**	mg/collection	1.25	12.54	1.25	12.63	0.48	4.85	1.66	16.62	1.88	18.96
**Propylene Glycol**	mg/collection	0.40	4.03	0.40	4.01	0.36	3.62	0.60	6.02	0.60	6.02
**Water**	mg/collection	4.63	9.97	4.63	9.96	1.39	3.08	1.39	3.01	6.95	15.10
**Menthol**	mg/collection	0.05	0.48	0.05	0.48	0.01	0.14	0.01	0.14	0.07	0.74
**Diethylene Glycol**	mg/collection	0.04	0.24	0.04	0.26	0.01	0.08	0.01	0.08	0.07	0.41
**Ethylene Glycol**	mg/collection	0.03	0.25	0.03	0.27	0.01	0.07	0.01	0.08	0.05	0.46
**Carbonyls & Glycidol**		**LOD**	**LOQ**	**LOD**	**LOQ**	**LOD**	**LOQ**	**LOD**	**LOQ**	**LOD**	**LOQ**
**Acetaldehyde**	mcg/collection	0.24	1.32	0.24	1.31	0.20	1.13	0.24	1.32	0.22	1.32
**Acrolein**	mcg/collection	0.18	1.31	0.18	1.32	0.15	1.13	0.18	1.32	0.16	1.32
**Diacetyl**	mcg/collection	0.13	1.34	0.13	1.30	0.11	1.12	0.13	1.32	0.18	1.32
**Formaldehyde**	mcg/collection	0.44	1.32	0.44	1.31	0.38	1.13	0.44	1.32	0.17	1.31
**Glycidol**	mcg/collection	0.004	0.03	0.003	0.03	0.003	0.03	0.003	0.03	0.005	0.05
**Acetyl Propionyl**	mcg/collection	0.13	1.32	0.13	1.33	0.11	1.14	0.13	1.33	0.18	1.33
**Butyraldehyde**	mcg/collection	0.13	1.32	0.13	1.30	0.11	1.13	0.13	1.32	0.14	1.32
**Crotonaldehyde**	mcg/collection	0.21	1.29	0.21	1.32	0.18	1.13	0.21	1.32	0.18	1.31
**Furfural**	mcg/collection	0.22	1.32	0.22	1.32	0.19	1.13	0.22	1.32	0.25	1.32
**Nicotine Degradants & TSNAs**		**LOD**	**LOQ**	**LOD**	**LOQ**	**LOD**	**LOQ**	**LOD**	**LOQ**	**LOD**	**LOQ**
**β-Nicotyrine**	mcg/collection	0.11	1.06	0.10	1.06	0.11	1.06	0.11	1.04	0.10	1.04
**Cotinine**	mcg/collection	0.11	1.06	0.10	1.06	0.11	1.06	0.11	1.05	0.22	1.05
**Myosmine**	mcg/collection	0.11	1.05	0.10	1.05	0.11	1.05	0.11	1.06	0.11	1.06
**Nicotine N Oxide**	mcg/collection	0.11	1.05	0.10	1.05	0.11	1.05	0.11	1.04	0.26	1.04
**Nornicotine**	mcg/collection	0.11	1.05	0.10	1.05	0.11	1.05	0.11	1.04	0.27	1.04
**Anabasine**	mcg/collection	0.11	1.06	0.12	1.06	0.11	1.06	0.11	1.06	0.15	1.06
**Anatabine**	mcg/collection	0.11	1.06	0.11	1.06	0.11	1.06	0.11	1.06	0.12	1.06
**NNK**	mcg/collection	0.001	0.003	0.001	0.003	NT	NT	NT	NT	NT	NT
**NNN**	mcg/collection	0.001	0.003	0.001	0.003	NT	NT	NT	NT	NT	NT

Metals		LOD	LOQ	LOD	LOQ	LOD	LOQ	LOD	LOQ	LOD	LOQ
**Arsenic**	ng/collection	10.00	50.00	10.00	50.00	10.00	50.00	10.00	50.00	10.00	50.00
**Beryllium**	ng/collection	2.20	20.00	2.20	20.00	2.20	20.00	2.20	20.00	2.20	20.00
**Cadmium**	ng/collection	1.00	5.00	1.00	5.00	1.00	5.00	1.00	5.00	1.00	5.00
**Chromium**	ng/collection	10.00	50.00	10.00	50.00	10.00	50.00	10.00	50.00	10.00	50.00
**Cobalt**	ng/collection	0.50	5.00	0.50	5.00	0.50	5.00	0.50	5.00	0.50	5.00
**Copper**	ng/collection	25.00	75.00	25.00	75.00	25.00	75.00	25.00	75.00	25.00	75.00
**Gold**	ng/collection	170.00	200.00	170.00	200.20	170.00	200.00	170.00	200.00	170.00	200.00
**Iron**	ng/collection	100.00	200.00	100.00	200.00	100.00	200.00	100.00	200.00	100.00	200.00
**Lead**	ng/collection	2.50	10.00	2.50	10.00	2.50	10.00	2.50	10.00	2.50	10.00
**Nickel**	ng/collection	5.00	10.00	5.00	10.00	5.00	10.00	5.00	10.00	5.00	10.00
**Selenium**	ng/collection	3.90	20.00	3.90	20.00	3.90	20.00	3.90	20.00	3.90	20.00
**Silver**	ng/collection	1.00	5.00	1.00	5.00	1.00	5.00	1.00	5.00	1.00	5.00
**Tin**	ng/collection	10.00	50.00	10.00	50.00	10.00	50.00	10.00	50.00	10.00	50.00
**Zinc**	ng/collection	150.00	350.00	150.00	350.00	150.00	350.00	150.00	350.00	150.00	350.00
**Organic Acids**		**LOD**	**LOQ**	**LOD**	**LOQ**	**LOD**	**LOQ**	**LOD**	**LOQ**	**LOD**	**LOQ**
**Benzoic Acid**	mg/collection	0.06	0.71	0.06	0.35	0.06	0.35	0.05	0.50	0.07	0.70
**Propionic Acid**	mcg/collection	61.89	352.71	61.89	354.83	61.89	354.83	50.44	506.90	70.61	706.10
**VOCs**		**LOD**	**LOQ**	**LOD**	**LOQ**	**LOD**	**LOQ**	**LOD**	**LOQ**	**LOD**	**LOQ**
**1,3-butadiene**	mcg/collection	0.05	0.20	0.05	0.20	NT	NT	NT	NT	NT	NT
**Acrylonitrile**	mcg/collection	0.04	0.21	0.04	0.21	NT	NT	NT	NT	NT	NT
**Benzene**	mcg/collection	0.06	0.31	0.06	0.31	NT	NT	NT	NT	NT	NT
**Isoprene**	mcg/collection	0.08	0.27	0.08	0.27	NT	NT	NT	NT	NT	NT
**Propylene Oxide**	mcg/collection	0.15	0.88	0.15	0.87	NT	NT	NT	NT	NT	NT
**Toluene**	mcg/collection	0.09	0.35	0.09	0.35	NT	NT	NT	NT	NT	NT
**Esters & Alcohols**		**LOD**	**LOQ**	**LOD**	**LOQ**	**LOD**	**LOQ**	**LOD**	**LOQ**	**LOD**	**LOQ**
**1-Butanol**	mcg/collection	1.91	19.47	1.91	19.66	NT	NT	NT	NT	NT	NT
**Benzyl Acetate**	mcg/collection	2.52	25.22	2.52	25.65	NT	NT	NT	NT	NT	NT
**Ethyl Acetate**	mcg/collection	2.00	20.48	2.00	20.38	NT	NT	NT	NT	NT	NT

**Ethyl Acetoacetate**	mcg/collection	3.90	27.83	3.90	25.01	NT	NT	NT	NT	NT	NT
**Isoamyl Acetate**	mcg/collection	2.03	20.78	2.03	21.02	NT	NT	NT	NT	NT	NT
**Isobutyl Acetate**	mcg/collection	2.02	20.59	2.02	20.78	NT	NT	NT	NT	NT	NT
**Methyl Acetate**	mcg/collection	2.09	21.60	2.09	21.98	NT	NT	NT	NT	NT	NT

1.11 Summer Menthol 18 mg/mL Non-Intense Per Collection Limit of Detection and Quantification Values

**Parameter/ Constituent**	**Units**	**T0**		**T3**		**T6**		**T9**		**T12**	
**pH**	NA	NA	NA	NA	NA	NA	NA	NA	NA	NA	NA
**Primary Constituents**		**LOD**	**LOQ**	**LOD**	**LOQ**	**LOD**	**LOQ**	**LOD**	**LOQ**	**LOD**	**LOQ**
**Nicotine**	mg/collection	0.15	1.50	0.15	1.50	0.05	0.45	0.05	0.45	0.23	2.25
**Glycerol**	mg/collection	1.25	12.54	1.25	12.63	0.70	7.01	1.66	16.62	1.88	18.96
**Propylene Glycol**	mg/collection	0.40	4.03	0.40	4.01	0.24	2.41	0.60	6.02	0.60	6.02
**Water**	mg/collection	4.63	9.97	4.63	9.96	1.39	3.08	1.39	3.01	6.95	15.10
**Menthol**	mg/collection	0.05	0.48	0.05	0.48	0.01	0.14	0.01	0.14	0.07	0.74
**Diethylene Glycol**	mg/collection	0.04	0.24	0.04	0.28	0.01	0.08	0.01	0.08	0.07	0.41
**Ethylene Glycol**	mg/collection	0.03	0.25	0.03	0.25	0.01	0.07	0.01	0.08	0.05	0.46
**Carbonyls & Glycidol**		**LOD**	**LOQ**	**LOD**	**LOQ**	**LOD**	**LOQ**	**LOD**	**LOQ**	**LOD**	**LOQ**
**Acetaldehyde**	mcg/collection	0.20	1.13	0.20	1.13	0.20	1.13	0.20	1.13	0.19	1.13
**Acrolein**	mcg/collection	0.15	1.13	0.15	1.13	0.15	1.13	0.15	1.13	0.14	1.13
**Diacetyl**	mcg/collection	0.11	1.15	0.11	1.12	0.11	1.12	0.11	1.13	0.16	1.13
**Formaldehyde**	mcg/collection	0.38	1.13	0.38	1.13	0.38	1.13	0.38	1.13	0.15	1.13
**Glycidol**	mcg/collection	0.003	0.03	0.003	0.03	0.003	0.03	0.003	0.03	0.005	0.05
**Acetyl Propionyl**	mcg/collection	0.11	1.13	0.11	1.14	0.11	1.14	0.11	1.14	0.15	1.14
**Butyraldehyde**	mcg/collection	0.11	1.13	0.11	1.11	0.11	1.13	0.11	1.13	0.12	1.13
**Crotonaldehyde**	mcg/collection	0.18	1.11	0.18	1.13	0.18	1.13	0.18	1.13	0.15	1.13
**Furfural**	mcg/collection	0.19	1.13	0.19	1.13	0.19	1.13	0.19	1.13	0.21	1.13
**Nicotine Degradants & TSNAs**		**LOD**	**LOQ**	**LOD**	**LOQ**	**LOD**	**LOQ**	**LOD**	**LOQ**	**LOD**	**LOQ**

**β-Nicotyrine**	mcg/collection	0.09	0.90	0.09	0.90	0.09	0.90	0.09	0.89	0.09	0.89
**Cotinine**	mcg/collection	0.09	0.90	0.09	0.90	0.09	0.90	0.09	0.90	0.19	0.90
**Myosmine**	mcg/collection	0.09	0.90	0.09	0.90	0.09	0.90	0.09	0.91	0.09	0.91
**Nicotine N Oxide**	mcg/collection	0.09	0.90	0.09	0.90	0.09	0.90	0.09	0.89	0.22	0.89
**Nornicotine**	mcg/collection	0.09	0.90	0.09	0.90	0.09	0.90	0.09	0.90	0.23	0.90
**Anabasine**	mcg/collection	0.09	0.91	0.10	0.91	0.09	0.91	0.09	0.91	0.13	0.91
**Anatabine**	mcg/collection	0.09	0.90	0.09	0.90	0.09	0.90	0.09	0.91	0.11	0.91
**NNK**	mcg/collection	0.001	0.003	0.001	0.003	NT	NT	NT	NT	NT	NT
**NNN**	mcg/collection	0.001	0.003	0.001	0.003	NT	NT	NT	NT	NT	NT
**Metals**		**LOD**	**LOQ**	**LOD**	**LOQ**	**LOD**	**LOQ**	**LOD**	**LOQ**	**LOD**	**LOQ**
**Arsenic**	ng/collection	10.00	50.00	10.00	50.00	10.00	50.00	10.00	50.00	10.00	50.00
**Beryllium**	ng/collection	2.20	20.00	2.20	20.00	2.20	20.00	2.20	20.00	2.20	20.00
**Cadmium**	ng/collection	1.00	5.00	1.00	5.00	1.00	5.00	1.00	5.00	1.00	5.00
**Chromium**	ng/collection	10.00	50.00	10.00	50.00	10.00	50.00	10.00	50.00	10.00	50.00
**Cobalt**	ng/collection	0.50	5.00	0.50	5.00	0.50	5.00	0.50	5.00	0.50	5.00
**Copper**	ng/collection	25.00	75.00	25.00	75.00	25.00	75.00	25.00	75.00	25.00	75.00
**Gold**	ng/collection	170.00	200.00	170.00	200.20	170.00	200.00	170.00	200.00	170.00	200.00
**Iron**	ng/collection	100.00	200.00	100.00	200.00	100.00	200.00	100.00	200.00	100.00	200.00
**Lead**	ng/collection	2.50	10.00	2.50	10.00	2.50	10.00	2.50	10.00	2.50	10.00
**Nickel**	ng/collection	5.00	10.00	5.00	10.00	5.00	10.00	5.00	10.00	5.00	10.00
**Selenium**	ng/collection	3.90	20.00	3.90	20.00	3.90	20.00	3.90	20.00	3.90	20.00
**Silver**	ng/collection	1.00	5.00	1.00	5.00	1.00	5.00	1.00	5.00	1.00	5.00
**Tin**	ng/collection	10.00	50.00	10.00	50.00	10.00	50.00	10.00	50.00	10.00	50.00
**Zinc**	ng/collection	150.00	350.00	150.00	350.00	150.00	350.00	150.00	350.00	150.00	350.00
**Organic Acids**		**LOD**	**LOQ**	**LOD**	**LOQ**	**LOD**	**LOQ**	**LOD**	**LOQ**	**LOD**	**LOQ**
**Benzoic Acid**	mg/collection	0.04	0.35	0.06	0.35	0.04	0.35	0.07	0.40	0.07	0.70
**Propionic Acid**	mcg/collection	46.17	352.71	61.89	354.83	35.27	354.83	40.31	405.52	70.61	706.10
**VOCs**		**LOD**	**LOQ**	**LOD**	**LOQ**	**LOD**	**LOQ**	**LOD**	**LOQ**	**LOD**	**LOQ**
**1,3-butadiene**	mcg/collection	0.05	0.20	0.07	0.20	NT	NT	NT	NT	NT	NT

**Acrylonitrile**	mcg/collection	0.04	0.21	0.02	0.20	NT	NT	NT	NT	NT	NT
**Benzene**	mcg/collection	0.06	0.31	0.06	0.31	NT	NT	NT	NT	NT	NT
**Isoprene**	mcg/collection	0.08	0.27	0.06	0.27	NT	NT	NT	NT	NT	NT
**Propylene Oxide**	mcg/collection	0.15	0.87	0.23	0.87	NT	NT	NT	NT	NT	NT
**Toluene**	mcg/collection	0.09	0.35	0.04	0.35	NT	NT	NT	NT	NT	NT
**Esters & Alcohols**		**LOD**	**LOQ**	**LOD**	**LOQ**	**LOD**	**LOQ**	**LOD**	**LOQ**	**LOD**	**LOQ**
**1-Butanol**	mcg/collection	1.91	19.47	1.91	19.71	NT	NT	NT	NT	NT	NT
**Benzyl Acetate**	mcg/collection	2.52	25.22	2.52	25.17	NT	NT	NT	NT	NT	NT
**Ethyl Acetate**	mcg/collection	2.00	20.48	2.00	20.24	NT	NT	NT	NT	NT	NT
**Ethyl Acetoacetate**	mcg/collection	3.90	27.83	3.90	24.81	NT	NT	NT	NT	NT	NT
**Isoamyl Acetate**	mcg/collection	2.03	20.78	2.03	20.83	NT	NT	NT	NT	NT	NT
**Isobutyl Acetate**	mcg/collection	2.02	20.59	2.02	20.50	NT	NT	NT	NT	NT	NT
**Methyl Acetate**	mcg/collection	2.09	21.60	2.09	21.07	NT	NT	NT	NT	NT	NT

1.12 Summer Menthol 18 mg/mL Intense Per Collection Limit of Detection and Quantification Values

**Parameter/ Constituent**	**Units**	**T0**		**T3**		**T6**		**T9**		**T12**	
**pH**	NA	NA	NA	NA	NA	NA	NA	NA	NA	NA	NA
**Primary Constituents**		**LOD**	**LOQ**	**LOD**	**LOQ**	**LOD**	**LOQ**	**LOD**	**LOQ**	**LOD**	**LOQ**
**Nicotine**	mg/collection	0.15	1.50	0.15	1.50	0.05	0.45	0.05	0.45	0.23	2.25
**Glycerol**	mg/collection	1.25	12.54	1.25	12.63	0.59	5.93	1.66	16.62	1.88	18.96
**Propylene Glycol**	mg/collection	0.40	4.03	0.40	4.01	0.36	3.62	0.60	6.02	0.60	6.02
**Water**	mg/collection	4.63	9.97	4.63	9.96	1.39	3.08	1.39	3.01	6.95	15.10
**Menthol**	mg/collection	0.05	0.48	0.05	0.48	0.01	0.14	0.01	0.14	0.07	0.74
**Diethylene Glycol**	mg/collection	0.04	0.24	0.04	0.26	0.01	0.08	0.01	0.08	0.07	0.41
**Ethylene Glycol**	mg/collection	0.03	0.25	0.03	0.27	0.01	0.07	0.01	0.08	0.05	0.46
**Carbonyls & Glycidol**		**LOD**	**LOQ**	**LOD**	**LOQ**	**LOD**	**LOQ**	**LOD**	**LOQ**	**LOD**	**LOQ**
**Acetaldehyde**	mcg/collection	0.24	1.32	0.24	1.31	0.20	1.13	0.24	1.32	0.22	1.32

**Acrolein**	mcg/collection	0.18	1.31	0.18	1.32	0.15	1.13	0.18	1.32	0.16	1.32
**Diacetyl**	mcg/collection	0.13	1.34	0.13	1.30	0.11	1.12	0.13	1.32	0.18	1.32
**Formaldehyde**	mcg/collection	0.44	1.32	0.44	1.31	0.38	1.13	0.44	1.32	0.17	1.31
**Glycidol**	mcg/collection	0.004	0.03	0.003	0.03	0.003	0.03	0.003	0.03	0.005	0.05
**Acetyl Propionyl**	mcg/collection	0.13	1.32	0.13	1.33	0.11	1.14	0.13	1.33	0.18	1.33
**Butyraldehyde**	mcg/collection	0.13	1.32	0.13	1.30	0.11	1.13	0.13	1.32	0.14	1.32
**Crotonaldehyde**	mcg/collection	0.21	1.29	0.21	1.32	0.18	1.13	0.21	1.32	0.18	1.31
**Furfural**	mcg/collection	0.22	1.32	0.22	1.32	0.19	1.13	0.22	1.32	0.25	1.32
**Nicotine Degradants & TSNAs**		**LOD**	**LOQ**	**LOD**	**LOQ**	**LOD**	**LOQ**	**LOD**	**LOQ**	**LOD**	**LOQ**
**β-Nicotyrine**	mcg/collection	0.11	1.06	0.10	1.06	0.11	1.06	0.11	1.04	0.10	1.04
**Cotinine**	mcg/collection	0.11	1.06	0.10	1.06	0.11	1.06	0.11	1.05	0.22	1.05
**Myosmine**	mcg/collection	0.11	1.05	0.10	1.05	0.11	1.05	0.11	1.06	0.11	1.06
**Nicotine N Oxide**	mcg/collection	0.11	1.05	0.10	1.05	0.11	1.05	0.11	1.04	0.26	1.04
**Nornicotine**	mcg/collection	0.11	1.05	0.10	1.05	0.11	1.05	0.11	1.04	0.27	1.04
**Anabasine**	mcg/collection	0.11	1.06	0.12	1.06	0.11	1.06	0.11	1.06	0.15	1.06
**Anatabine**	mcg/collection	0.11	1.06	0.11	1.06	0.11	1.06	0.11	1.06	0.12	1.06
**NNK**	mcg/collection	0.001	0.003	0.001	0.003	NT	NT	NT	NT	NT	NT
**NNN**	mcg/collection	0.001	0.003	0.001	0.003	NT	NT	NT	NT	NT	NT
**Metals**		**LOD**	**LOQ**	**LOD**	**LOQ**	**LOD**	**LOQ**	**LOD**	**LOQ**	**LOD**	**LOQ**
**Arsenic**	ng/collection	10.00	50.00	10.00	50.00	10.00	50.00	10.00	50.00	10.00	50.00
**Beryllium**	ng/collection	2.20	20.00	2.20	20.00	2.20	20.00	2.20	20.00	2.20	20.00
**Cadmium**	ng/collection	1.00	5.00	1.00	5.00	1.00	5.00	1.00	5.00	1.00	5.00
**Chromium**	ng/collection	10.00	50.00	10.00	50.00	10.00	50.00	10.00	50.00	10.00	50.00
**Cobalt**	ng/collection	0.50	5.00	0.50	5.00	0.50	5.00	0.50	5.00	0.50	5.00
**Copper**	ng/collection	25.00	75.00	25.00	75.00	25.00	75.00	25.00	75.00	25.00	75.00
**Gold**	ng/collection	170.00	200.00	170.00	200.20	170.00	200.00	170.00	200.00	170.00	200.00
**Iron**	ng/collection	100.00	200.00	100.00	200.00	100.00	200.00	100.00	200.00	100.00	200.00
**Lead**	ng/collection	2.50	10.00	2.50	10.00	2.50	10.00	2.50	10.00	2.50	10.00
**Nickel**	ng/collection	5.00	10.00	5.00	10.00	5.00	10.00	5.00	10.00	5.00	10.00

**Selenium**	ng/collection	3.90	20.00	3.90	20.00	3.90	20.00	3.90	20.00	3.90	20.00
**Silver**	ng/collection	1.00	5.00	1.00	5.00	1.00	5.00	1.00	5.00	1.00	5.00
**Tin**	ng/collection	10.00	50.00	10.00	50.00	10.00	50.00	10.00	50.00	10.00	50.00
**Zinc**	ng/collection	150.00	350.00	150.00	350.00	150.00	350.00	150.00	350.00	150.00	350.00
**Organic Acids**		**LOD**	**LOQ**	**LOD**	**LOQ**	**LOD**	**LOQ**	**LOD**	**LOQ**	**LOD**	**LOQ**
**Benzoic Acid**	mg/collection	0.06	0.71	0.06	0.35	0.06	0.35	0.06	0.55	0.07	0.70
**Propionic Acid**	mcg/collection	61.89	352.71	61.89	354.83	61.89	354.83	55.48	557.58	70.61	706.10
**VOCs**		**LOD**	**LOQ**	**LOD**	**LOQ**	**LOD**	**LOQ**	**LOD**	**LOQ**	**LOD**	**LOQ**
**1,3-butadiene**	mcg/collection	0.05	0.20	0.05	0.20	NT	NT	NT	NT	NT	NT
**Acrylonitrile**	mcg/collection	0.04	0.21	0.04	0.21	NT	NT	NT	NT	NT	NT
**Benzene**	mcg/collection	0.06	0.31	0.06	0.31	NT	NT	NT	NT	NT	NT
**Isoprene**	mcg/collection	0.08	0.27	0.08	0.27	NT	NT	NT	NT	NT	NT
**Propylene Oxide**	mcg/collection	0.15	0.88	0.15	0.87	NT	NT	NT	NT	NT	NT
**Toluene**	mcg/collection	0.09	0.35	0.09	0.35	NT	NT	NT	NT	NT	NT
**Esters & Alcohols**		**LOD**	**LOQ**	**LOD**	**LOQ**	**LOD**	**LOQ**	**LOD**	**LOQ**	**LOD**	**LOQ**
**1-Butanol**	mcg/collection	1.91	19.47	1.91	19.66	NT	NT	NT	NT	NT	NT
**Benzyl Acetate**	mcg/collection	2.52	25.22	2.52	25.65	NT	NT	NT	NT	NT	NT
**Ethyl Acetate**	mcg/collection	2.00	20.48	2.00	20.38	NT	NT	NT	NT	NT	NT
**Ethyl Acetoacetate**	mcg/collection	3.90	27.83	3.90	25.01	NT	NT	NT	NT	NT	NT
**Isoamyl Acetate**	mcg/collection	2.03	20.78	2.03	21.02	NT	NT	NT	NT	NT	NT
**Isobutyl Acetate**	mcg/collection	2.02	20.59	2.02	20.78	NT	NT	NT	NT	NT	NT
**Methyl Acetate**	mcg/collection	2.09	21.60	2.09	21.98	NT	NT	NT	NT	NT	NT
