# Supplementary material for: A Practical Framework for Novel Electronic Nicotine Delivery System Evaluation: Chemical and Toxicological Characterization of JUUL2 Aerosol and Comparison with Reference Cigarettes
Source: Toxics. 2024 Jan 4;12(1):41. doi: 10.3390/toxics12010041 (PMC10820849; doi:10.3390/toxics12010041)
Supplement: Supplementary file 1 [file toxics-12-00041-s001.zip › Supplementary_Material_File S3_Per Collection T0-T12 Analytical Stability Results_Final_Clean.pdf]

Supplementary Materials File S3

1 Non-Intense and Intense Per Collection Analytical Stability Results

1.1 Virginia Tobacco 18 mg/mL Non-Intense Per Collection Analytical Stability Results

Parameter/ Constituent	Units	T0		T3		T6		T9		T12	
**# of puffs**	NA	150		134		141		132		114	
**pH**	NA	6.01	0.03	6.19	0.06	6.14	0.03	5.90	0.03	5.93	0.02
**Primary Constituents**		**Mean**	**SD**	**Mean**	**SD**	**Mean**	**SD**	**Mean**	**SD**	**Mean**	**SD**
**Nicotine**	mg/collection	18.37	1.50	16.81	0.63	17.59	1.09	18.73	0.74	15.78	0.43
**Glycerol**	mg/collection	700.87	61.10	704.93	22.99	709.29	51.07	788.91	33.42	686.60	30.83
**Propylene Glycol**	mg/collection	438.43	36.56	420.23	16.52	430.05	27.37	457.94	18.08	390.86	9.29
**Water**	mg/collection	83.01	6.33	94.60	1.51	99.93	3.65	87.42	1.67	82.23	2.11
**Menthol**	mg/collection	BLOD	NA	BLOQ	NA	BLOD	NA	BLOD	NA	BLOQ	NA
**Diethylene Glycol**	mg/collection	BLOD	NA	BLOD	NA	BLOD	NA	BLOD	NA	BLOD	NA
**Ethylene Glycol**	mg/collection	BLOD	NA	BLOD	NA	BLOD	NA	BLOD	NA	BLOD	NA
**Carbonyls & Glycidol**		**Mean**	**SD**	**Mean**	**SD**	**Mean**	**SD**	**Mean**	**SD**	**Mean**	**SD**
**Acetaldehyde**	mcg/collection	3.54	0.42	2.83	0.47	3.19	0.59	4.05	0.42	4.32	0.36
**Acrolein**	mcg/collection	1.62	0.38	1.21	0.55	BLOQ	NA	BLOQ	NA	2.81	0.27
**Diacetyl**	mcg/collection	BLOQ	NA	BLOQ	NA	BLOD	NA	BLOD	NA	BLOQ	NA
**Formaldehyde**	mcg/collection	15.47	2.36	16.00	1.96	15.29	3.40	19.55	1.53	17.87	0.33
**Glycidol**	mcg/collection	4.11	0.41	3.29	0.61	4.21	0.68	4.40	0.35	3.09	0.26
**Acetyl Propionyl**	mcg/collection	BLOD	NA	BLOD	NA	BLOD	NA	BLOD	NA	BLOD	NA
**Butyraldehyde**	mcg/collection	BLOD	NA	BLOD	NA	BLOD	NA	BLOD	NA	BLOD	NA
**Crotonaldehyde**	mcg/collection	BLOD	NA	BLOD	NA	BLOD	NA	BLOD	NA	BLOD	NA
**Furfural**	mcg/collection	BLOD	NA	BLOD	NA	BLOD	NA	BLOD	NA	BLOD	NA
**Nicotine Degradants & TSNAs**		**Mean**	**SD**	**Mean**	**SD**	**Mean**	**SD**	**Mean**	**SD**	**Mean**	**SD**

**β-Nicotyrine**	mcg/collection	1.55	0.06	1.16	0.08	1.33	0.19	2.53	0.10	1.75	0.10
**Cotinine**	mcg/collection	34.27	2.05	40.53	3.84	31.71	5.05	39.19	1.68	35.46	2.10
**Myosmine**	mcg/collection	31.51	1.77	33.50	3.02	28.04	4.32	30.22	1.18	26.10	1.41
**Nicotine N Oxide**	mcg/collection	BLOQ	NA	BLOQ	NA	1.36	0.86	BLOQ	NA	BLOQ	NA
**Nornicotine**	mcg/collection	6.10	0.45	9.21	0.76	9.85	1.61	12.40	0.57	12.49	0.91
**Anabasine**	mcg/collection	BLOD	NA	BLOD	NA	BLOD	NA	BLOD	NA	BLOD	NA
**Anatabine**	mcg/collection	BLOD	NA	BLOD	NA	BLOD	NA	BLOQ	NA	BLOD	NA
**NNK**	mcg/collection	BLOQ	NA	NT	NT	NT	NT	NT	NT	NT	NT
**NNN**	mcg/collection	BLOD	NA	NT	NT	NT	NT	NT	NT	NT	NT
**Metals**		**Mean**	**SD**	**Mean**	**SD**	**Mean**	**SD**	**Mean**	**SD**	**Mean**	**SD**
**Arsenic**	ng/collection	BLOD	NA	BLOQ	NA	BLOD	NA	BLOQ	NA	BLOD	NA
**Beryllium**	ng/collection	BLOD	NA	BLOD	NA	BLOD	NA	BLOD	NA	BLOD	NA
**Cadmium**	ng/collection	BLOD	NA	BLOD	NA	BLOD	NA	BLOD	NA	BLOD	NA
**Chromium**	ng/collection	BLOQ	NA	BLOD	NA	BLOD	NA	BLOD	NA	BLOQ	NA
**Cobalt**	ng/collection	BLOD	NA	BLOD	NA	BLOD	NA	BLOD	NA	BLOD	NA
**Copper**	ng/collection	BLOQ	NA	BLOD	NA	BLOD	NA	BLOD	NA	BLOD	NA
**Gold**	ng/collection	BLOD	NA	NT	NT	NT	NT	NT	NT	NT	NT
**Iron**	ng/collection	BLOQ	NA	BLOD	NA	BLOD	NA	BLOD	NA	BLOD	NA
**Lead**	ng/collection	BLOD	NA	BLOD	NA	BLOD	NA	BLOD	NA	BLOD	NA
**Nickel**	ng/collection	BLOD	NA	BLOD	NA	BLOD	NA	BLOD	NA	BLOD	NA
**Selenium**	ng/collection	BLOQ	NA	23.49	12.51	BLOQ	NA	BLOQ	NA	BLOQ	NA
**Silver**	ng/collection	BLOD	NA	BLOD	NA	BLOD	NA	BLOD	NA	BLOD	NA
**Tin**	ng/collection	BLOQ	NA	BLOD	NA	BLOD	NA	BLOQ	NA	BLOD	NA
**Zinc**	ng/collection	BLOQ	NA	BLOD	NA	BLOD	NA	BLOD	NA	BLOD	NA
**Organic Acids**		**Mean**	**SD**	**Mean**	**SD**	**Mean**	**SD**	**Mean**	**SD**	**Mean**	**SD**
**Benzoic Acid**	mg/collection	9.86	1.09	8.24	0.42	9.97	0.31	9.30	0.39	8.30	0.59
**Propionic Acid**	mcg/collection	BLOD	NA	BLOD	NA	BLOD	NA	BLOQ	NA	BLOD	NA
**VOCs**		**Mean**	**SD**	**Mean**	**SD**	**Mean**	**SD**	**Mean**	**SD**	**Mean**	**SD**

**1,3-Butadiene**	mcg/collection	BLOD	NA	NT	NT	NT	NT	NT	NT	NT	NT
**Acrylonitrile**	mcg/collection	BLOD	NA	NT	NT	NT	NT	NT	NT	NT	NT
**Benzene**	mcg/collection	BLOD	NA	NT	NT	NT	NT	NT	NT	NT	NT
**Isoprene**	mcg/collection	BLOD	NA	NT	NT	NT	NT	NT	NT	NT	NT
**Propylene Oxide**	mcg/collection	BLOD	NA	NT	NT	NT	NT	NT	NT	NT	NT
**Toluene**	mcg/collection	BLOQ	NA	NT	NT	NT	NT	NT	NT	NT	NT
**Esters & Alcohols**		**Mean**	**SD**	**Mean**	**SD**	**Mean**	**SD**	**Mean**	**SD**	**Mean**	**SD**
**1-Butanol**	mcg/collection	BLOD	NA	NT	NT	NT	NT	NT	NT	NT	NT
**Benzyl Acetate**	mcg/collection	BLOD	NA	NT	NT	NT	NT	NT	NT	NT	NT
**Ethyl Acetate**	mcg/collection	BLOD	NA	NT	NT	NT	NT	NT	NT	NT	NT
**Ethyl Acetoacetate**	mcg/collection	BLOD	NA	NT	NT	NT	NT	NT	NT	NT	NT
**Isoamyl Acetate**	mcg/collection	BLOD	NA	NT	NT	NT	NT	NT	NT	NT	NT
**Isobutyl Acetate**	mcg/collection	BLOD	NA	NT	NT	NT	NT	NT	NT	NT	NT
**Methyl Acetate**	mcg/collection	BLOD	NA	NT	NT	NT	NT	NT	NT	NT	NT

1.2 Virginia Tobacco 18 mg/mL Intense Per Collection Analytical Stability Results

**Parameter/ Constituent**	**Units**	**T0**		**T3**		**T6**		**T9**		**T12**	
**# of puffs**	NA	85 (*86)		86		87		84		83	
**pH**	NA	6.17	0.02	6.46	0.04	5.96	0.04	6.17	0.12	5.96	0.08
***Primary Constituents**		**Mean**	**SD**	**Mean**	**SD**	**Mean**	**SD**	**Mean**	**SD**	**Mean**	**SD**
**Nicotine**	mg/collection	17.55	1.18	18.35	0.82	18.07	1.11	17.82	2.62	16.82	2.66
**Glycerol**	mg/collection	662.20	41.85	756.14	32.64	743.65	40.63	722.64	90.53	698.73	98.83
**Propylene Glycol**	mg/collection	420.44	27.99	463.43	20.45	445.13	28.01	445.16	64.40	415.85	66.08
**Water**	mg/collection	69.63	1.85	84.55	2.02	82.28	3.34	89.10	4.98	81.48	6.26
**Menthol**	mg/collection	BLOD	NA	BLOD	NA	BLOD	NA	BLOD	NA	BLOD	NA
**Diethylene Glycol**	mg/collection	BLOD	NA	BLOD	NA	BLOD	NA	BLOD	NA	BLOD	NA
**Ethylene Glycol**	mg/collection	BLOD	NA	BLOD	NA	BLOD	NA	BLOD	NA	BLOD	NA

Carbonyls & Glycidol		Mean	SD	Mean	SD	Mean	SD	Mean	SD	Mean	SD
**Acetaldehyde**	mcg/collection	4.49	0.39	3.85	0.47	4.16	0.10	4.44	0.55	6.66	0.51
**Acrolein**	mcg/collection	3.17	0.42	2.32	0.43	2.06	0.19	2.20	1.31	6.82	0.58
**Diacetyl**	mcg/collection	BLOQ	NA	BLOQ	NA	BLOQ	NA	BLOD	NA	BLOQ	NA
**Formaldehyde**	mcg/collection	22.81	1.07	21.66	2.51	22.93	0.88	23.06	5.93	24.95	1.13
**Glycidol**	mcg/collection	5.91	2.22	3.88	0.81	5.33	0.78	3.75	0.66	4.45	0.36
**Acetyl Propionyl**	mcg/collection	BLOD	NA	BLOD	NA	BLOD	NA	BLOD	NA	BLOD	NA
**Butyraldehyde**	mcg/collection	BLOD	NA	BLOD	NA	BLOQ	NA	BLOD	NA	BLOD	NA
**Crotonaldehyde**	mcg/collection	BLOD	NA	BLOD	NA	BLOD	NA	BLOD	NA	BLOD	NA
**Furfural**	mcg/collection	BLOD	NA	BLOD	NA	BLOD	NA	BLOQ	NA	BLOD	NA
**Nicotine Degradants & TSNAs**		**Mean**	**SD**	**Mean**	**SD**	**Mean**	**SD**	**Mean**	**SD**	**Mean**	**SD**
**β-Nicotyrine**	mcg/collection	2.40	0.35	2.13	0.10	2.87	0.18	3.09	0.86	2.39	0.19
**Cotinine**	mcg/collection	35.45	3.73	36.39	2.88	41.42	2.15	34.43	9.22	42.75	2.24
**Myosmine**	mcg/collection	33.03	3.54	29.96	2.29	35.74	1.53	26.35	6.61	32.27	1.89
**Nicotine N Oxide**	mcg/collection	BLOQ	NA	BLOQ	NA	1.14	0.07	BLOQ	NA	BLOQ	NA
**Nornicotine**	mcg/collection	6.59	0.66	8.04	0.86	11.98	0.89	10.82	3.18	14.64	0.83
**Anabasine**	mcg/collection	BLOD	NA	BLOD	NA	BLOD	NA	BLOD	NA	BLOD	NA
**Anatabine**	mcg/collection	BLOD	NA	BLOD	NA	BLOD	NA	BLOQ	NA	BLOD	NA
**NNK**	mcg/collection	BLOQ	NA	NT	NT	NT	NT	NT	NT	NT	NT
**NNN**	mcg/collection	BLOD	NA	NT	NT	NT	NT	NT	NT	NT	NT
**Metals**		**Mean**	**SD**	**Mean**	**SD**	**Mean**	**SD**	**Mean**	**SD**	**Mean**	**SD**
**Arsenic**	ng/collection	BLOD	NA	BLOD	NA	BLOD	NA	BLOD	NA	BLOD	NA
**Beryllium**	ng/collection	BLOD	NA	BLOD	NA	BLOD	NA	BLOD	NA	BLOD	NA
**Cadmium**	ng/collection	BLOD	NA	BLOD	NA	BLOD	NA	BLOD	NA	BLOD	NA
**Chromium**	ng/collection	72.07	50.94	BLOD	NA	BLOD	NA	BLOD	NA	BLOD	NA
**Cobalt**	ng/collection	BLOD	NA	BLOD	NA	BLOD	NA	BLOD	NA	BLOD	NA
**Copper**	ng/collection	BLOD	NA	BLOD	NA	BLOD	NA	BLOD	NA	BLOD	NA
**Gold**	ng/collection	BLOD	NA	NT	NT	NT	NT	NT	NT	NT	NT

**Iron**	ng/collection	596.74	475.36	BLOD	NA	BLOD	NA	BLOD	NA	BLOD	NA
**Lead**	ng/collection	BLOD	NA	BLOD	NA	BLOD	NA	BLOD	NA	BLOD	NA
**Nickel**	ng/collection	BLOQ	NA	BLOD	NA	BLOD	NA	BLOD	NA	BLOD	NA
**Selenium**	ng/collection	BLOQ	NA	BLOQ	NA	BLOQ	NA	BLOQ	NA	BLOQ	NA
**Silver**	ng/collection	BLOD	NA	BLOQ	NA	BLOD	NA	BLOD	NA	BLOD	NA
**Tin**	ng/collection	BLOQ	NA	BLOQ	NA	BLOQ	NA	BLOD	NA	BLOD	NA
**Zinc**	ng/collection	BLOD	NA	BLOD	NA	BLOD	NA	BLOD	NA	BLOD	NA
**Organic Acids**		**Mean**	**SD**	**Mean**	**SD**	**Mean**	**SD**	**Mean**	**SD**	**Mean**	**SD**
**Benzoic Acid**	mg/collection	9.31	1.17	11.47	0.74	14.17	1.77	11.52	0.82	9.86	2.22
**Propionic Acid**	mcg/collection	BLOD	NA	BLOQ	NA	BLOD	NA	BLOD	NA	BLOQ	NA
**VOCs**		**Mean**	**SD**	**Mean**	**SD**	**Mean**	**SD**	**Mean**	**SD**	**Mean**	**SD**
**1,3-Butadiene**	mcg/collection	BLOD	NA	NT	NT	NT	NT	NT	NT	NT	NT
**Acrylonitrile**	mcg/collection	BLOD	NA	NT	NT	NT	NT	NT	NT	NT	NT
**Benzene**	mcg/collection	BLOD	NA	NT	NT	NT	NT	NT	NT	NT	NT
**Isoprene**	mcg/collection	BLOD	NA	NT	NT	NT	NT	NT	NT	NT	NT
**Propylene Oxide**	mcg/collection	BLOD	NA	NT	NT	NT	NT	NT	NT	NT	NT
**Toluene**	mcg/collection	BLOQ	NA	NT	NT	NT	NT	NT	NT	NT	NT
**Esters & Alcohols**		**Mean**	**SD**	**Mean**	**SD**	**Mean**	**SD**	**Mean**	**SD**	**Mean**	**SD**
**1-Butanol**	mcg/collection	BLOD	NA	NT	NT	NT	NT	NT	NT	NT	NT
**Benzyl Acetate**	mcg/collection	BLOD	NA	NT	NT	NT	NT	NT	NT	NT	NT
**Ethyl Acetate**	mcg/collection	BLOD	NA	NT	NT	NT	NT	NT	NT	NT	NT
**Ethyl Acetoacetate**	mcg/collection	BLOD	NA	NT	NT	NT	NT	NT	NT	NT	NT
**Isoamyl Acetate**	mcg/collection	BLOD	NA	NT	NT	NT	NT	NT	NT	NT	NT
**Isobutyl Acetate**	mcg/collection	BLOD	NA	NT	NT	NT	NT	NT	NT	NT	NT
**Methyl Acetate**	mcg/collection	BLOD	NA	NT	NT	NT	NT	NT	NT	NT	NT

1.3 Crisp Menthol 18 mg/mL Non-Intense Per Collection Analytical Stability Results

Parameter/ Constituent	Units	T0		T3		T6		T9		T12	
**# of puffs**	NA	125		112		121		120		115	
**pH**	NA	6.26	0.03	6.36	0.02	6.33	0.02	6.30	0.06	6.11	0.06
**Primary Constituents**		**Mean**	**SD**	**Mean**	**SD**	**Mean**	**SD**	**Mean**	**SD**	**Mean**	**SD**
**Nicotine**	mg/collection	17.52	0.72	16.57	1.37	16.72	0.68	17.07	2.66	16.22	0.43
**Glycerol**	mg/collection	447.80	20.89	444.82	36.58	430.90	19.42	450.65	69.80	429.32	10.68
**Propylene Glycol**	mg/collection	609.23	27.86	624.50	52.74	635.13	41.91	641.90	100.91	594.88	16.17
**Water**	mg/collection	78.21	3.71	62.73	3.29	73.73	4.35	69.39	5.58	75.50	6.89
**Menthol**	mg/collection	12.57	0.65	12.05	1.04	11.98	0.47	12.61	2.07	11.41	0.31
**Diethylene Glycol**	mg/collection	BLOD	NA	BLOD	NA	BLOD	NA	BLOD	NA	BLOD	NA
**Ethylene Glycol**	mg/collection	BLOD	NA	BLOD	NA	BLOD	NA	BLOD	NA	BLOD	NA
**Carbonyls & Glycidol**		**Mean**	**SD**	**Mean**	**SD**	**Mean**	**SD**	**Mean**	**SD**	**Mean**	**SD**
**Acetaldehyde**	mcg/collection	2.40	0.32	2.65	0.65	2.67	0.33	2.50	0.21	2.70	0.48
**Acrolein**	mcg/collection	BLOQ	NA	BLOQ	NA	BLOQ	NA	BLOQ	NA	1.72	0.70
**Diacetyl**	mcg/collection	BLOQ	NA	BLOQ	NA	BLOQ	NA	BLOD	NA	BLOQ	NA
**Formaldehyde**	mcg/collection	8.96	1.68	11.56	2.58	10.34	0.65	11.34	1.01	11.63	1.78
**Glycidol**	mcg/collection	2.90	0.19	2.09	0.14	2.33	0.39	2.69	0.65	2.67	0.70
**Acetyl Propionyl**	mcg/collection	BLOD	NA	BLOD	NA	BLOD	NA	BLOD	NA	BLOD	NA
**Butyraldehyde**	mcg/collection	BLOD	NA	BLOD	NA	BLOD	NA	BLOD	NA	BLOD	NA
**Crotonaldehyde**	mcg/collection	BLOD	NA	BLOD	NA	BLOD	NA	BLOD	NA	BLOD	NA
**Furfural**	mcg/collection	BLOD	NA	BLOD	NA	BLOD	NA	BLOD	NA	BLOD	NA
**Nicotine Degradants & TSNAs**		**Mean**	**SD**	**Mean**	**SD**	**Mean**	**SD**	**Mean**	**SD**	**Mean**	**SD**
**β-Nicotyrine**	mcg/collection	1.88	0.11	2.40	0.33	2.83	0.15	4.20	0.42	3.65	0.50
**Cotinine**	mcg/collection	BLOQ	NA	BLOQ	NA	BLOQ	NA	BLOQ	NA	BLOQ	NA
**Myosmine**	mcg/collection	1.53	0.10	1.74	0.22	2.20	0.22	2.46	0.27	2.69	0.33
**Nicotine N Oxide**	mcg/collection	BLOQ	NA	1.64	0.70	1.35	0.49	1.76	0.32	0.98	0.28
**Nornicotine**	mcg/collection	7.21	0.38	8.93	1.64	12.83	0.69	12.39	1.28	14.35	2.19
**Anabasine**	mcg/collection	BLOD	NA	BLOD	NA	BLOD	NA	BLOD	NA	BLOD	NA

**Anatabine**	mcg/collection	BLOD	NA	BLOD	NA	BLOD	NA	BLOD	NA	BLOD	NA
**NNK**	mcg/collection	BLOQ	NA	NT	NT	NT	NT	NT	NT	NT	NT
**NNN**	mcg/collection	BLOD	NA	NT	NT	NT	NT	NT	NT	NT	NT
**Metals**		**Mean**	**SD**	**Mean**	**SD**	**Mean**	**SD**	**Mean**	**SD**	**Mean**	**SD**
**Arsenic**	ng/collection	BLOD	NA	BLOD	NA	BLOD	NA	BLOD	NA	BLOD	NA
**Beryllium**	ng/collection	BLOD	NA	BLOD	NA	BLOD	NA	BLOD	NA	BLOD	NA
**Cadmium**	ng/collection	BLOD	NA	BLOD	NA	BLOD	NA	BLOD	NA	BLOD	NA
**Chromium**	ng/collection	BLOQ	NA	BLOD	NA	BLOD	NA	BLOD	NA	BLOD	NA
**Cobalt**	ng/collection	BLOD	NA	BLOD	NA	BLOD	NA	BLOD	NA	BLOD	NA
**Copper**	ng/collection	BLOQ	NA	BLOD	NA	BLOD	NA	BLOD	NA	BLOD	NA
**Gold**	ng/collection	BLOD	NA	NT	NT	NT	NT	NT	NT	NT	NT
**Iron**	ng/collection	214.12	203.93	BLOD	NA	BLOD	NA	BLOD	NA	BLOD	NA
**Lead**	ng/collection	BLOD	NA	BLOD	NA	BLOD	NA	BLOD	NA	BLOD	NA
**Nickel**	ng/collection	BLOD	NA	BLOD	NA	BLOD	NA	BLOD	NA	BLOD	NA
**Selenium**	ng/collection	BLOQ	NA	BLOQ	NA	BLOQ	NA	BLOQ	NA	BLOQ	NA
**Silver**	ng/collection	BLOD	NA	BLOD	NA	BLOD	NA	BLOD	NA	BLOD	NA
**Tin**	ng/collection	BLOQ	NA	BLOQ	NA	BLOD	NA	BLOD	NA	BLOD	NA
**Zinc**	ng/collection	BLOQ	NA	BLOD	NA	BLOD	NA	BLOD	NA	BLOD	NA
**Organic Acids**		**Mean**	**SD**	**Mean**	**SD**	**Mean**	**SD**	**Mean**	**SD**	**Mean**	**SD**
**Benzoic Acid**	mg/collection	10.86	3.49	8.55	2.14	9.80	0.54	9.19	0.39	8.44	0.79
**Propionic Acid**	mcg/collection	BLOD	NA	BLOD	NA	BLOD	NA	BLOQ	NA	BLOQ	NA
**VOCs**		**Mean**	**SD**	**Mean**	**SD**	**Mean**	**SD**	**Mean**	**SD**	**Mean**	**SD**
**1,3-Butadiene**	mcg/collection	BLOD	NA	NT	NT	NT	NT	NT	NT	NT	NT
**Acrylonitrile**	mcg/collection	BLOD	NA	NT	NT	NT	NT	NT	NT	NT	NT
**Benzene**	mcg/collection	BLOD	NA	NT	NT	NT	NT	NT	NT	NT	NT
**Isoprene**	mcg/collection	BLOD	NA	NT	NT	NT	NT	NT	NT	NT	NT
**Propylene Oxide**	mcg/collection	BLOD	NA	NT	NT	NT	NT	NT	NT	NT	NT
**Toluene**	mcg/collection	BLOD	NA	NT	NT	NT	NT	NT	NT	NT	NT
**Esters & Alcohols**		**Mean**	**SD**	**Mean**	**SD**	**Mean**	**SD**	**Mean**	**SD**	**Mean**	**SD**

**1-Butanol**	mcg/collection	BLOD	NA	NT	NT	NT	NT	NT	NT	NT	NT
**Benzyl Acetate**	mcg/collection	BLOD	NA	NT	NT	NT	NT	NT	NT	NT	NT
**Ethyl Acetate**	mcg/collection	110.62	6.75	NT	NT	NT	NT	NT	NT	NT	NT
**Ethyl Acetoacetate**	mcg/collection	BLOD	NA	NT	NT	NT	NT	NT	NT	NT	NT
**Isoamyl Acetate**	mcg/collection	BLOD	NA	NT	NT	NT	NT	NT	NT	NT	NT
**Isobutyl Acetate**	mcg/collection	BLOD	NA	NT	NT	NT	NT	NT	NT	NT	NT
**Methyl Acetate**	mcg/collection	BLOD	NA	NT	NT	NT	NT	NT	NT	NT	NT

1.4 Crisp Menthol 18 mg/mL Intense Per Collection Analytical Stability Results

Parameter/ Constituent	Units	T0		T3		T6		T9		T12	
**# of puffs**	NA	81		88		82		87		81	
**pH**	NA	6.24	0.02	6.53	0.05	6.27	0.03	6.20	0.06	6.17	0.10
**Primary Constituents**		**Mean**	**SD**	**Mean**	**SD**	**Mean**	**SD**	**Mean**	**SD**	**Mean**	**SD**
**Nicotine**	mg/collection	17.99	1.08	18.02	0.45	17.86	0.54	19.66	0.14	18.26	0.57
**Glycerol**	mg/collection	452.86	25.80	483.47	11.68	465.17	13.98	520.53	5.74	488.44	13.95
**Propylene Glycol**	mg/collection	620.15	43.76	678.08	15.45	664.71	20.56	737.84	1.76	678.13	21.65
**Water**	mg/collection	68.93	0.80	70.12	1.81	76.48	2.74	74.65	1.88	78.37	2.65
**Menthol**	mg/collection	12.98	0.74	12.77	0.28	12.27	0.42	14.11	0.08	12.73	0.41
**Diethylene Glycol**	mg/collection	BLOD	NA	BLOD	NA	BLOD	NA	BLOD	NA	BLOD	NA
**Ethylene Glycol**	mg/collection	BLOD	NA	BLOD	NA	BLOD	NA	BLOD	NA	BLOD	NA
**Carbonyls & Glycidol**		**Mean**	**SD**	**Mean**	**SD**	**Mean**	**SD**	**Mean**	**SD**	**Mean**	**SD**
**Acetaldehyde**	mcg/collection	2.78	0.24	2.35	0.40	2.63	0.17	4.28	0.73	4.07	0.48
**Acrolein**	mcg/collection	BLOQ	NA	BLOQ	NA	BLOQ	NA	1.66	0.84	4.02	0.29
**Diacetyl**	mcg/collection	BLOQ	NA	BLOQ	NA	BLOQ	NA	BLOQ	NA	BLOQ	NA
**Formaldehyde**	mcg/collection	13.96	2.12	14.26	1.43	12.91	1.10	20.48	7.12	14.14	0.84
**Glycidol**	mcg/collection	2.94	1.08	3.02	0.30	4.99	0.71	2.71	0.56	2.68	0.89

**Acetyl Propionyl**	mcg/collection	BLOD	NA	BLOD	NA	BLOD	NA	BLOD	NA	BLOD	NA
**Butyraldehyde**	mcg/collection	BLOD	NA	BLOD	NA	BLOD	NA	BLOD	NA	BLOD	NA
**Crotonaldehyde**	mcg/collection	BLOD	NA	BLOD	NA	BLOD	NA	BLOD	NA	BLOD	NA
**Furfural**	mcg/collection	BLOD	NA	BLOD	NA	BLOD	NA	BLOD	NA	BLOD	NA
**Nicotine Degradants & TSNAs**		**Mean**	**SD**	**Mean**	**SD**	**Mean**	**SD**	**Mean**	**SD**	**Mean**	**SD**
**β-Nicotyrine**	mcg/collection	2.27	0.09	2.73	0.19	3.47	0.15	4.10	0.41	3.70	0.27
**Cotinine**	mcg/collection	BLOQ	NA	BLOQ	NA	BLOQ	NA	1.20	0.36	BLOQ	NA
**Myosmine**	mcg/collection	1.87	0.10	2.27	0.17	2.58	0.12	3.10	0.23	2.66	0.18
**Nicotine N Oxide**	mcg/collection	1.14	0.10	BLOQ	NA	BLOQ	NA	1.18	0.35	1.08	0.30
**Nornicotine**	mcg/collection	8.06	0.60	10.79	0.45	13.41	0.42	15.09	1.70	13.00	1.22
**Anabasine**	mcg/collection	BLOD	NA	BLOD	NA	BLOD	NA	BLOD	NA	BLOD	NA
**Anatabine**	mcg/collection	BLOD	NA	BLOD	NA	BLOD	NA	BLOD	NA	BLOD	NA
**NNK**	mcg/collection	BLOQ	NA	NT	NT	NT	NT	NT	NT	NT	NT
**NNN**	mcg/collection	BLOD	NA	NT	NT	NT	NT	NT	NT	NT	NT
**Metals**		**Mean**	**SD**	**Mean**	**SD**	**Mean**	**SD**	**Mean**	**SD**	**Mean**	**SD**
**Arsenic**	ng/collection	BLOD	NA	BLOD	NA	BLOD	NA	BLOD	NA	BLOD	NA
**Beryllium**	ng/collection	BLOD	NA	BLOD	NA	BLOD	NA	BLOD	NA	BLOD	NA
**Cadmium**	ng/collection	BLOD	NA	BLOD	NA	BLOD	NA	BLOD	NA	BLOD	NA
**Chromium**	ng/collection	BLOQ	NA	BLOD	NA	BLOD	NA	BLOD	NA	BLOD	NA
**Cobalt**	ng/collection	BLOD	NA	BLOD	NA	BLOD	NA	BLOD	NA	BLOD	NA
**Copper**	ng/collection	BLOD	NA	BLOD	NA	BLOD	NA	BLOD	NA	BLOD	NA
**Gold**	ng/collection	BLOD	NA	NT	NT	NT	NT	NT	NT	NT	NT
**Iron**	ng/collection	221.24	106.93	BLOD	NA	BLOD	NA	BLOD	NA	BLOD	NA
**Lead**	ng/collection	BLOD	NA	BLOD	NA	BLOD	NA	BLOD	NA	BLOD	NA
**Nickel**	ng/collection	BLOD	NA	BLOD	NA	BLOD	NA	BLOD	NA	BLOD	NA
**Selenium**	ng/collection	BLOQ	NA	BLOQ	NA	BLOQ	NA	BLOQ	NA	BLOQ	NA
**Silver**	ng/collection	BLOD	NA	BLOQ	NA	BLOD	NA	BLOD	NA	BLOD	NA
**Tin**	ng/collection	BLOQ	NA	BLOQ	NA	BLOQ	NA	BLOQ	NA	BLOD	NA
**Zinc**	ng/collection	BLOD	NA	BLOD	NA	BLOD	NA	BLOD	NA	BLOD	NA

Organic Acids		Mean	SD	Mean	SD	Mean	SD	Mean	SD	Mean	SD
**Benzoic Acid**	mg/collection	13.20	1.19	12.29	1.59	9.56	3.13	12.03	1.41	11.49	1.49
**Propionic Acid**	mcg/collection	BLOD	NA	BLOD	NA	BLOD	NA	BLOQ	NA	BLOQ	NA
**VOCs**		**Mean**	**SD**	**Mean**	**SD**	**Mean**	**SD**	**Mean**	**SD**	**Mean**	**SD**
**1,3-Butadiene**	mcg/collection	BLOD	NA	NT	NT	NT	NT	NT	NT	NT	NT
**Acrylonitrile**	mcg/collection	BLOD	NA	NT	NT	NT	NT	NT	NT	NT	NT
**Benzene**	mcg/collection	BLOD	NA	NT	NT	NT	NT	NT	NT	NT	NT
**Isoprene**	mcg/collection	BLOD	NA	NT	NT	NT	NT	NT	NT	NT	NT
**Propylene Oxide**	mcg/collection	BLOD	NA	NT	NT	NT	NT	NT	NT	NT	NT
**Toluene**	mcg/collection	BLOQ	NA	NT	NT	NT	NT	NT	NT	NT	NT
**Esters & Alcohols**		**Mean**	**SD**	**Mean**	**SD**	**Mean**	**SD**	**Mean**	**SD**	**Mean**	**SD**
**1-Butanol**	mcg/collection	BLOD	NA	NT	NT	NT	NT	NT	NT	NT	NT
**Benzyl Acetate**	mcg/collection	BLOD	NA	NT	NT	NT	NT	NT	NT	NT	NT
**Ethyl Acetate**	mcg/collection	118.72	3.37	NT	NT	NT	NT	NT	NT	NT	NT
**Ethyl Acetoacetate**	mcg/collection	BLOD	NA	NT	NT	NT	NT	NT	NT	NT	NT
**Isoamyl Acetate**	mcg/collection	BLOD	NA	NT	NT	NT	NT	NT	NT	NT	NT
**Isobutyl Acetate**	mcg/collection	BLOD	NA	NT	NT	NT	NT	NT	NT	NT	NT
**Methyl Acetate**	mcg/collection	BLOD	NA	NT	NT	NT	NT	NT	NT	NT	NT

1.5 Polar Menthol 18 mg/mL Non-Intense Per Collection Analytical Stability Results

Parameter/ Constituent	Units	T0		T3		T6		T9		T12	
**# of puffs**	NA	100		132		115		122		106	
**pH**	NA	6.31	0.05	6.55	0.02	6.32	0.03	6.38	0.05	6.54	0.03
**Primary Constituents**		**Mean**	**SD**	**Mean**	**SD**	**Mean**	**SD**	**Mean**	**SD**	**Mean**	**SD**
**Nicotine**	mg/collection	16.02	0.68	17.72	0.71	16.56	0.88	19.31	0.33	15.21	1.79
**Glycerol**	mg/collection	410.15	19.55	482.42	18.92	440.06	22.96	515.48	9.40	412.34	48.16

**Propylene Glycol**	mg/collection	566.40	28.43	679.43	25.88	744.50	69.18	729.91	13.55	566.07	54.97
**Water**	mg/collection	58.69	2.00	84.49	2.49	73.28	2.64	74.13	1.71	74.80	6.20
**Menthol**	mg/collection	12.36	0.57	13.54	0.68	12.70	0.78	15.22	0.30	11.51	1.41
**Diethylene Glycol**	mg/collection	BLOD	NA	BLOD	NA	BLOD	NA	BLOD	NA	BLOD	NA
**Ethylene Glycol**	mg/collection	BLOD	NA	BLOD	NA	BLOD	NA	BLOD	NA	BLOD	NA
**Carbonyls & Glycidol**		**Mean**	**SD**	**Mean**	**SD**	**Mean**	**SD**	**Mean**	**SD**	**Mean**	**SD**
**Acetaldehyde**	mcg/collection	BLOQ	NA	3.11	0.43	2.58	0.44	3.66	0.39	4.41	0.52
**Acrolein**	mcg/collection	BLOQ	NA	BLOQ	NA	BLOQ	NA	BLOQ	NA	1.95	0.10
**Diacetyl**	mcg/collection	BLOD	NA	BLOD	NA	BLOD	NA	BLOD	NA	BLOD	NA
**Formaldehyde**	mcg/collection	5.09	0.70	11.93	1.69	8.99	1.55	12.36	1.14	13.34	0.97
**Glycidol**	mcg/collection	1.83	0.17	2.31	0.14	2.39	0.33	2.72	0.50	2.50	0.46
**Acetyl Propionyl**	mcg/collection	BLOD	NA	BLOD	NA	BLOD	NA	BLOD	NA	BLOD	NA
**Butyraldehyde**	mcg/collection	BLOD	NA	BLOD	NA	BLOD	NA	BLOD	NA	BLOD	NA
**Crotonaldehyde**	mcg/collection	BLOD	NA	BLOD	NA	BLOD	NA	BLOD	NA	BLOD	NA
**Furfural**	mcg/collection	BLOD	NA	BLOD	NA	BLOD	NA	BLOD	NA	BLOD	NA
**Nicotine Degradants & TSNAs**		**Mean**	**SD**	**Mean**	**SD**	**Mean**	**SD**	**Mean**	**SD**	**Mean**	**SD**
**β-Nicotyrine**	mcg/collection	BLOQ	NA	2.50	0.41	2.39	0.35	5.31	0.62	4.69	0.90
**Cotinine**	mcg/collection	BLOQ	NA	1.55	0.25	1.43	0.17	1.30	0.12	2.39	0.35
**Myosmine**	mcg/collection	1.37	0.18	3.75	0.59	3.83	0.57	5.34	0.43	6.08	0.70
**Nicotine N Oxide**	mcg/collection	BLOQ	NA	1.30	0.50	1.94	1.03	1.87	0.18	0.95	0.28
**Nornicotine**	mcg/collection	2.14	0.30	7.49	1.33	8.57	1.37	12.17	1.30	14.24	1.79
**Anabasine**	mcg/collection	BLOD	NA	BLOD	NA	BLOD	NA	BLOD	NA	BLOD	NA
**Anatabine**	mcg/collection	BLOQ	NA	BLOQ	NA	BLOQ	NA	BLOQ	NA	BLOQ	NA
**NNK**	mcg/collection	BLOQ	NA	NT	NT	NT	NT	NT	NT	NT	NT
**NNN**	mcg/collection	BLOD	NA	NT	NT	NT	NT	NT	NT	NT	NT
**Metals**		**Mean**	**SD**	**Mean**	**SD**	**Mean**	**SD**	**Mean**	**SD**	**Mean**	**SD**
**Arsenic**	ng/collection	BLOD	NA	BLOQ	NA	BLOQ	NA	BLOQ	NA	BLOD	NA
**Beryllium**	ng/collection	BLOD	NA	BLOD	NA	BLOD	NA	BLOD	NA	BLOD	NA
**Cadmium**	ng/collection	BLOD	NA	BLOD	NA	BLOD	NA	BLOD	NA	BLOD	NA

**Chromium**	ng/collection	BLOQ	NA	BLOD	NA	BLOD	NA	BLOD	NA	BLOD	NA
**Cobalt**	ng/collection	BLOD	NA	BLOD	NA	BLOD	NA	BLOD	NA	BLOD	NA
**Copper**	ng/collection	BLOQ	NA	BLOD	NA	BLOQ	NA	BLOD	NA	BLOQ	NA
**Gold**	ng/collection	BLOD	NA	NT	NT	NT	NT	NT	NT	NT	NT
**Iron**	ng/collection	BLOQ	NA	BLOD	NA	BLOD	NA	BLOD	NA	BLOD	NA
**Lead**	ng/collection	BLOD	NA	BLOD	NA	BLOD	NA	BLOD	NA	BLOD	NA
**Nickel**	ng/collection	BLOD	NA	BLOQ	NA	BLOQ	NA	BLOD	NA	BLOD	NA
**Selenium**	ng/collection	BLOQ	NA	BLOQ	NA	20.06	12.11	BLOQ	NA	BLOQ	NA
**Silver**	ng/collection	BLOD	NA	BLOD	NA	BLOD	NA	BLOD	NA	BLOD	NA
**Tin**	ng/collection	BLOD	NA	BLOD	NA	BLOD	NA	BLOD	NA	BLOD	NA
**Zinc**	ng/collection	BLOQ	NA	BLOD	NA	BLOD	NA	BLOD	NA	BLOD	NA
**Organic Acids**		**Mean**	**SD**	**Mean**	**SD**	**Mean**	**SD**	**Mean**	**SD**	**Mean**	**SD**
**Benzoic Acid**	mg/collection	8.14	0.53	10.19	0.61	9.62	0.27	10.06	0.57	8.30	1.01
**Propionic Acid**	mcg/collection	BLOD	NA	BLOD	NA	BLOD	NA	BLOQ	NA	BLOD	NA
**VOCs**		**Mean**	**SD**	**Mean**	**SD**	**Mean**	**SD**	**Mean**	**SD**	**Mean**	**SD**
**1,3-Butadiene**	mcg/collection	BLOD	NA	NT	NT	NT	NT	NT	NT	NT	NT
**Acrylonitrile**	mcg/collection	BLOD	NA	NT	NT	NT	NT	NT	NT	NT	NT
**Benzene**	mcg/collection	BLOD	NA	NT	NT	NT	NT	NT	NT	NT	NT
**Isoprene**	mcg/collection	BLOD	NA	NT	NT	NT	NT	NT	NT	NT	NT
**Propylene Oxide**	mcg/collection	BLOD	NA	NT	NT	NT	NT	NT	NT	NT	NT
**Toluene**	mcg/collection	BLOD	NA	NT	NT	NT	NT	NT	NT	NT	NT
**Esters & Alcohols**		**Mean**	**SD**	**Mean**	**SD**	**Mean**	**SD**	**Mean**	**SD**	**Mean**	**SD**
**1-Butanol**	mcg/collection	BLOD	NA	NT	NT	NT	NT	NT	NT	NT	NT
**Benzyl Acetate**	mcg/collection	BLOD	NA	NT	NT	NT	NT	NT	NT	NT	NT
**Ethyl Acetate**	mcg/collection	BLOQ	NA	NT	NT	NT	NT	NT	NT	NT	NT
**Ethyl Acetoacetate**	mcg/collection	BLOD	NA	NT	NT	NT	NT	NT	NT	NT	NT
**Isoamyl Acetate**	mcg/collection	BLOD	NA	NT	NT	NT	NT	NT	NT	NT	NT
**Isobutyl Acetate**	mcg/collection	BLOD	NA	NT	NT	NT	NT	NT	NT	NT	NT

**Methyl Acetate**	mcg/collection	BLOD	NA	NT	NT	NT	NT	NT	NT	NT	NT

1.6 Polar Menthol 18 mg/mL Intense Per Collection Analytical Stability Results

**Parameter/ Constituent**	**Units**	**T0**		**T3**		**T6**		**T9**		**T12**	
**# of puffs**	NA	81		81		81		81		80	
**pH**	NA	6.24	0.04	6.57	0.04	6.36	0.03	6.22	0.06	6.42	0.05
**Primary Constituents**		**Mean**	**SD**	**Mean**	**SD**	**Mean**	**SD**	**Mean**	**SD**	**Mean**	**SD**
**Nicotine**	mg/collection	17.82	1.18	17.71	1.65	17.56	0.59	19.55	0.25	18.02	0.32
**Glycerol**	mg/collection	455.73	28.72	483.23	43.54	467.18	12.42	524.99	6.87	492.91	7.25
**Propylene Glycol**	mg/collection	629.21	38.52	715.07	103.73	660.56	21.54	740.35	10.85	676.06	12.86
**Water**	mg/collection	72.38	1.80	70.84	7.44	71.58	4.22	70.62	1.60	75.86	3.85
**Menthol**	mg/collection	13.67	0.93	13.42	1.22	12.77	0.45	15.05	0.23	13.29	0.17
**Diethylene Glycol**	mg/collection	BLOD	NA	BLOD	NA	BLOD	NA	BLOD	NA	BLOD	NA
**Ethylene Glycol**	mg/collection	BLOD	NA	BLOD	NA	BLOD	NA	BLOD	NA	BLOD	NA
**Carbonyls & Glycidol**		**Mean**	**SD**	**Mean**	**SD**	**Mean**	**SD**	**Mean**	**SD**	**Mean**	**SD**
**Acetaldehyde**	mcg/collection	1.99	0.11	2.68	0.33	3.20	0.47	4.65	0.33	5.66	1.12
**Acrolein**	mcg/collection	1.64	0.13	1.61	0.52	BLOQ	NA	1.44	0.42	5.03	2.20
**Diacetyl**	mcg/collection	BLOD	NA	BLOQ	NA	BLOQ	NA	BLOD	NA	BLOQ	NA
**Formaldehyde**	mcg/collection	11.88	0.85	13.48	0.73	13.29	1.52	16.90	2.08	15.60	3.38
**Glycidol**	mcg/collection	3.67	0.38	3.11	0.38	3.36	0.83	2.28	0.53	3.57	0.61
**Acetyl Propionyl**	mcg/collection	BLOD	NA	BLOD	NA	BLOD	NA	BLOD	NA	BLOD	NA
**Butyraldehyde**	mcg/collection	BLOD	NA	BLOD	NA	BLOD	NA	BLOD	NA	BLOD	NA
**Crotonaldehyde**	mcg/collection	BLOD	NA	BLOD	NA	BLOD	NA	BLOD	NA	BLOD	NA
**Furfural**	mcg/collection	BLOD	NA	BLOD	NA	BLOD	NA	BLOD	NA	BLOD	NA
**Nicotine Degradants & TSNAs**		**Mean**	**SD**	**Mean**	**SD**	**Mean**	**SD**	**Mean**	**SD**	**Mean**	**SD**
**β-Nicotyrine**	mcg/collection	BLOQ	NA	2.47	0.29	3.14	1.11	6.61	0.63	4.60	1.09
**Cotinine**	mcg/collection	BLOQ	NA	1.05	0.28	1.36	0.11	1.97	0.16	2.08	0.36

**Myosmine**	mcg/collection	2.18	0.17	3.81	0.43	4.94	0.46	5.71	0.41	5.91	1.24
**Nicotine N Oxide**	mcg/collection	BLOQ	NA	BLOQ	NA	BLOQ	NA	BLOQ	NA	BLOQ	NA
**Nornicotine**	mcg/collection	2.63	0.20	6.72	0.97	8.40	2.72	12.64	1.09	12.75	2.56
**Anabasine**	mcg/collection	BLOD	NA	BLOD	NA	BLOD	NA	BLOQ	NA	BLOD	NA
**Anatabine**	mcg/collection	BLOQ	NA	BLOQ	NA	BLOQ	NA	BLOQ	NA	BLOQ	NA
**NNK**	mcg/collection	BLOQ	NA	NT	NT	NT	NT	NT	NT	NT	NT
**NNN**	mcg/collection	BLOD	NA	NT	NT	NT	NT	NT	NT	NT	NT
**Metals**		**Mean**	**SD**	**Mean**	**SD**	**Mean**	**SD**	**Mean**	**SD**	**Mean**	**SD**
**Arsenic**	ng/collection	BLOD	NA	BLOD	NA	BLOQ	NA	BLOD	NA	BLOD	NA
**Beryllium**	ng/collection	BLOD	NA	BLOD	NA	BLOD	NA	BLOD	NA	BLOD	NA
**Cadmium**	ng/collection	BLOD	NA	BLOD	NA	BLOD	NA	BLOD	NA	BLOD	NA
**Chromium**	ng/collection	BLOQ	NA	BLOD	NA	BLOD	NA	BLOD	NA	BLOQ	NA
**Cobalt**	ng/collection	BLOD	NA	BLOD	NA	BLOD	NA	BLOD	NA	BLOD	NA
**Copper**	ng/collection	BLOD	NA	BLOD	NA	BLOD	NA	BLOD	NA	BLOD	NA
**Gold**	ng/collection	BLOD	NA	NT	NT	NT	NT	NT	NT	NT	NT
**Iron**	ng/collection	BLOQ	NA	BLOD	NA	BLOD	NA	BLOD	NA	BLOD	NA
**Lead**	ng/collection	BLOD	NA	BLOD	NA	BLOD	NA	BLOD	NA	BLOD	NA
**Nickel**	ng/collection	BLOD	NA	BLOQ	NA	BLOD	NA	BLOD	NA	BLOD	NA
**Selenium**	ng/collection	BLOQ	NA	BLOQ	NA	24.53	12.14	BLOQ	NA	BLOQ	NA
**Silver**	ng/collection	BLOD	NA	BLOQ	NA	BLOD	NA	BLOD	NA	BLOD	NA
**Tin**	ng/collection	BLOD	NA	BLOQ	NA	BLOQ	NA	BLOQ	NA	BLOD	NA
**Zinc**	ng/collection	BLOD	NA	BLOD	NA	BLOQ	NA	BLOD	NA	BLOD	NA
**Organic Acids**		**Mean**	**SD**	**Mean**	**SD**	**Mean**	**SD**	**Mean**	**SD**	**Mean**	**SD**
**Benzoic Acid**	mg/collection	12.87	3.98	12.65	0.95	11.16	1.17	12.41	0.74	10.82	1.07
**Propionic Acid**	mcg/collection	BLOD	NA	BLOQ	NA	BLOD	NA	BLOQ	NA	BLOD	NA
**VOCs**		**Mean**	**SD**	**Mean**	**SD**	**Mean**	**SD**	**Mean**	**SD**	**Mean**	**SD**
**1,3-Butadiene**	mcg/collection	BLOD	NA	NT	NT	NT	NT	NT	NT	NT	NT
**Acrylonitrile**	mcg/collection	BLOD	NA	NT	NT	NT	NT	NT	NT	NT	NT

**Benzene**	mcg/collection	BLOD	NA	NT	NT	NT	NT	NT	NT	NT	NT
**Isoprene**	mcg/collection	BLOD	NA	NT	NT	NT	NT	NT	NT	NT	NT
**Propylene Oxide**	mcg/collection	BLOD	NA	NT	NT	NT	NT	NT	NT	NT	NT
**Toluene**	mcg/collection	BLOD	NA	NT	NT	NT	NT	NT	NT	NT	NT
**Esters & Alcohols**		**Mean**	**SD**	**Mean**	**SD**	**Mean**	**SD**	**Mean**	**SD**	**Mean**	**SD**
**1-Butanol**	mcg/collection	BLOD	NA	NT	NT	NT	NT	NT	NT	NT	NT
**Benzyl Acetate**	mcg/collection	BLOD	NA	NT	NT	NT	NT	NT	NT	NT	NT
**Ethyl Acetate**	mcg/collection	BLOQ	NA	NT	NT	NT	NT	NT	NT	NT	NT
**Ethyl Acetoacetate**	mcg/collection	BLOD	NA	NT	NT	NT	NT	NT	NT	NT	NT
**Isoamyl Acetate**	mcg/collection	BLOD	NA	NT	NT	NT	NT	NT	NT	NT	NT
**Isobutyl Acetate**	mcg/collection	BLOD	NA	NT	NT	NT	NT	NT	NT	NT	NT
**Methyl Acetate**	mcg/collection	BLOD	NA	NT	NT	NT	NT	NT	NT	NT	NT

1.7 Autumn Tobacco 18 mg/mL Non-Intense Per Collection Analytical Stability Results

Parameter/ Constituent	Units	T0		T3		T6		T9		T12	
**# of puffs**	NA	128		128		119		122		124	
**pH**	NA	6.36	0.06	6.16	0.07	6.37	0.05	6.41	0.13	6.14	0.03
**Primary Constituents**		**Mean**	**SD**	**Mean**	**SD**	**Mean**	**SD**	**Mean**	**SD**	**Mean**	**SD**
**Nicotine**	mg/collection	16.46	1.65	16.54	0.81	16.69	1.20	17.66	0.57	17.99	0.29
**Glycerol**	mg/collection	427.06	41.69	427.12	22.52	414.24	29.35	430.50	12.54	440.29	5.03
**Propylene Glycol**	mg/collection	559.89	57.30	548.60	28.83	527.77	42.27	560.23	15.11	596.62	10.41
**Water**	mg/collection	53.15	4.83	74.07	1.95	69.81	4.32	78.37	2.07	112.33	15.49
**Menthol**	mg/collection	BLOD	NA	BLOD	NA	BLOD	NA	BLOD	NA	BLOD	NA
**Diethylene Glycol**	mg/collection	BLOD	NA	BLOD	NA	BLOD	NA	BLOD	NA	BLOD	NA
**Ethylene Glycol**	mg/collection	BLOD	NA	BLOD	NA	BLOD	NA	BLOD	NA	BLOD	NA
**Carbonyls & Glycidol**		**Mean**	**SD**	**Mean**	**SD**	**Mean**	**SD**	**Mean**	**SD**	**Mean**	**SD**
**Acetaldehyde**	mcg/collection	1.69	0.21	2.27	0.22	2.68	0.26	2.75	0.16	2.58	0.48

**Acrolein**	mcg/collection	1.16	0.37	BLOQ	NA	BLOQ	NA	BLOQ	NA	BLOQ	NA
**Diacetyl**	mcg/collection	BLOD	NA	BLOQ	NA	BLOD	NA	BLOQ	NA	BLOQ	NA
**Formaldehyde**	mcg/collection	9.16	0.33	7.28	0.20	7.81	0.43	8.77	0.59	9.69	1.20
**Glycidol**	mcg/collection	1.89	0.61	2.16	0.47	2.06	0.34	1.51	0.28	2.90	0.51
**Acetyl Propionyl**	mcg/collection	BLOD	NA	BLOD	NA	BLOD	NA	BLOD	NA	BLOD	NA
**Butyraldehyde**	mcg/collection	BLOD	NA	BLOD	NA	BLOD	NA	BLOQ	NA	BLOD	NA
**Crotonaldehyde**	mcg/collection	BLOD	NA	BLOD	NA	BLOD	NA	BLOD	NA	BLOD	NA
**Furfural**	mcg/collection	BLOD	NA	BLOD	NA	BLOD	NA	BLOD	NA	BLOD	NA
**Nicotine Degradants & TSNAs**		**Mean**	**SD**	**Mean**	**SD**	**Mean**	**SD**	**Mean**	**SD**	**Mean**	**SD**
**β-Nicotyrine**	mcg/collection	0.93	0.22	BLOQ	NA	1.96	0.21	1.21	0.34	1.83	0.27
**Cotinine**	mcg/collection	1.36	0.21	BLOQ	NA	1.25	0.17	3.33	1.88	1.76	0.21
**Myosmine**	mcg/collection	1.91	0.11	2.52	0.14	2.87	0.34	3.21	0.35	3.39	0.24
**Nicotine N Oxide**	mcg/collection	1.40	0.35	BLOQ	NA	1.40	0.17	4.43	2.98	1.34	0.69
**Nornicotine**	mcg/collection	3.52	0.22	4.41	0.67	7.84	0.81	10.43	2.97	9.95	0.63
**Anabasine**	mcg/collection	BLOD	NA	BLOD	NA	BLOD	NA	BLOD	NA	BLOD	NA
**Anatabine**	mcg/collection	BLOD	NA	BLOD	NA	BLOD	NA	BLOD	NA	BLOD	NA
**NNK**	mcg/collection	BLOQ	NA	BLOQ	NA	NT	NT	NT	NT	NT	NT
**NNN**	mcg/collection	BLOD	NA	BLOD	NA	NT	NT	NT	NT	NT	NT
**Metals**		**Mean**	**SD**	**Mean**	**SD**	**Mean**	**SD**	**Mean**	**SD**	**Mean**	**SD**
**Arsenic**	ng/collection	BLOQ	NA	BLOD	NA	BLOQ	NA	BLOD	NA	BLOD	NA
**Beryllium**	ng/collection	BLOD	NA	BLOD	NA	BLOD	NA	BLOD	NA	BLOD	NA
**Cadmium**	ng/collection	BLOD	NA	BLOD	NA	BLOD	NA	BLOD	NA	BLOD	NA
**Chromium**	ng/collection	BLOD	NA	BLOD	NA	BLOQ	NA	BLOD	NA	BLOD	NA
**Cobalt**	ng/collection	BLOD	NA	BLOD	NA	BLOD	NA	BLOD	NA	BLOD	NA
**Copper**	ng/collection	BLOD	NA	BLOD	NA	BLOD	NA	BLOD	NA	BLOD	NA
**Gold**	ng/collection	BLOD	NA	BLOD	NA	BLOD	NA	BLOD	NA	BLOD	NA
**Iron**	ng/collection	BLOD	NA	BLOD	NA	BLOD	NA	BLOD	NA	BLOD	NA
**Lead**	ng/collection	BLOD	NA	BLOD	NA	BLOD	NA	BLOD	NA	BLOD	NA

**Nickel**	ng/collection	BLOD	NA	BLOD	NA	BLOD	NA	BLOD	NA	BLOD	NA
**Selenium**	ng/collection	BLOQ	NA	BLOQ	NA	BLOQ	NA	BLOQ	NA	BLOD	NA
**Silver**	ng/collection	BLOD	NA	BLOD	NA	BLOD	NA	BLOD	NA	BLOD	NA
**Tin**	ng/collection	BLOD	NA	BLOD	NA	BLOQ	NA	BLOQ	NA	BLOD	NA
**Zinc**	ng/collection	BLOD	NA	BLOD	NA	BLOD	NA	BLOD	NA	BLOD	NA
**Organic Acids**		**Mean**	**SD**	**Mean**	**SD**	**Mean**	**SD**	**Mean**	**SD**	**Mean**	**SD**
**Benzoic Acid**	mg/collection	9.31	1.66	9.38	1.12	10.49	0.63	12.50	0.38	9.03	1.07
**Propionic Acid**	mcg/collection	BLOD	NA	BLOD	NA	BLOD	NA	BLOD	NA	BLOD	NA
**VOCs**		**Mean**	**SD**	**Mean**	**SD**	**Mean**	**SD**	**Mean**	**SD**	**Mean**	**SD**
**1,3-butadiene**	mcg/collection	BLOD	NA	BLOD	NA	NT	NT	NT	NT	NT	NT
**Acrylonitrile**	mcg/collection	BLOD	NA	BLOD	NA	NT	NT	NT	NT	NT	NT
**Benzene**	mcg/collection	BLOQ	NA	BLOQ	NA	NT	NT	NT	NT	NT	NT
**Isoprene**	mcg/collection	BLOD	NA	BLOD	NA	NT	NT	NT	NT	NT	NT
**Propylene Oxide**	mcg/collection	BLOD	NA	BLOD	NA	NT	NT	NT	NT	NT	NT
**Toluene**	mcg/collection	BLOQ	NA	BLOD	NA	NT	NT	NT	NT	NT	NT
**Esters & Alcohols**		**Mean**	**SD**	**Mean**	**SD**	**Mean**	**SD**	**Mean**	**SD**	**Mean**	**SD**
**1-Butanol**	mcg/collection	BLOD	NA	BLOQ	NA	NT	NT	NT	NT	NT	NT
**Benzyl Acetate**	mcg/collection	BLOD	NA	BLOD	NA	NT	NT	NT	NT	NT	NT
**Ethyl Acetate**	mcg/collection	BLOD	NA	BLOD	NA	NT	NT	NT	NT	NT	NT
**Ethyl Acetoacetate**	mcg/collection	BLOD	NA	BLOD	NA	NT	NT	NT	NT	NT	NT
**Isoamyl Acetate**	mcg/collection	BLOD	NA	BLOD	NA	NT	NT	NT	NT	NT	NT
**Isobutyl Acetate**	mcg/collection	BLOD	NA	BLOD	NA	NT	NT	NT	NT	NT	NT
**Methyl Acetate**	mcg/collection	BLOD	NA	BLOQ	NA	NT	NT	NT	NT	NT	NT

1.8 Autumn Tobacco 18 mg/mL Intense Per Collection Analytical Stability Results

Parameter/ Constituent	Units	T0	T3	T6	T9	T12
**# of puffs**	NA	80	76	68	72	68

**pH**	NA	6.43	0.06	6.27	0.03	6.35	0.06	6.06	0.05	6.12	0.03
**Primary Constituents**		**Mean**	**SD**	**Mean**	**SD**	**Mean**	**SD**	**Mean**	**SD**	**Mean**	**SD**
**Nicotine**	mg/collection	15.58	1.83	14.86	1.36	15.76	0.94	15.22	1.87	15.75	1.62
**Glycerol**	mg/collection	408.86	45.77	385.12	33.34	396.62	23.63	379.80	45.48	389.05	40.50
**Propylene Glycol**	mg/collection	530.42	64.90	502.73	47.25	499.31	32.39	487.07	56.68	523.30	53.66
**Water**	mg/collection	48.86	3.47	68.44	2.46	63.13	2.16	69.53	4.55	85.29	16.13
**Menthol**	mg/collection	BLOD	NA	BLOD	NA	BLOQ	NA	BLOD	NA	BLOD	NA
**Diethylene Glycol**	mg/collection	BLOD	NA	BLOD	NA	BLOD	NA	BLOD	NA	BLOD	NA
**Ethylene Glycol**	mg/collection	BLOD	NA	BLOD	NA	BLOD	NA	BLOD	NA	BLOD	NA
**Carbonyls & Glycidol**		**Mean**	**SD**	**Mean**	**SD**	**Mean**	**SD**	**Mean**	**SD**	**Mean**	**SD**
**Acetaldehyde**	mcg/collection	1.97	0.13	2.52	0.24	2.53	0.20	2.92	0.37	2.19	0.11
**Acrolein**	mcg/collection	1.98	0.46	BLOQ	NA	BLOQ	NA	BLOQ	NA	BLOQ	NA
**Diacetyl**	mcg/collection	BLOQ	NA	BLOQ	NA	BLOQ	NA	BLOQ	NA	BLOD	NA
**Formaldehyde**	mcg/collection	10.41	2.40	10.38	0.47	8.23	0.53	10.51	0.67	10.29	1.12
**Glycidol**	mcg/collection	3.87	0.82	3.26	0.47	2.80	0.53	2.19	0.36	4.25	0.45
**Acetyl Propionyl**	mcg/collection	BLOD	NA	BLOD	NA	BLOD	NA	BLOQ	NA	BLOD	NA
**Butyraldehyde**	mcg/collection	BLOD	NA	BLOD	NA	BLOD	NA	BLOD	NA	BLOD	NA
**Crotonaldehyde**	mcg/collection	BLOD	NA	BLOD	NA	BLOD	NA	BLOD	NA	BLOD	NA
**Furfural**	mcg/collection	BLOD	NA	BLOD	NA	BLOD	NA	BLOD	NA	BLOD	NA
**Nicotine Degradants & TSNAs**		**Mean**	**SD**	**Mean**	**SD**	**Mean**	**SD**	**Mean**	**SD**	**Mean**	**SD**
**β-Nicotyrine**	mcg/collection	BLOQ	NA	BLOQ	NA	1.85	0.20	1.30	0.36	1.69	0.35
**Cotinine**	mcg/collection	BLOQ	NA	BLOQ	NA	BLOQ	NA	4.18	3.81	1.69	0.26
**Myosmine**	mcg/collection	1.70	0.61	2.74	0.24	2.70	0.21	3.23	0.35	3.18	0.49
**Nicotine N Oxide**	mcg/collection	BLOQ	NA	BLOQ	NA	BLOQ	NA	4.44	3.65	BLOQ	NA
**Nornicotine**	mcg/collection	3.20	0.78	5.52	0.75	6.77	0.89	11.01	3.21	6.72	3.15
**Anabasine**	mcg/collection	BLOD	NA	BLOD	NA	BLOD	NA	BLOD	NA	BLOD	NA
**Anatabine**	mcg/collection	BLOD	NA	BLOD	NA	BLOD	NA	BLOD	NA	BLOD	NA
**NNK**	mcg/collection	BLOQ	NA	BLOQ	NA	NT	NT	NT	NT	NT	NT

**NNN**	mcg/collection	BLOD	NA	BLOQ	NA	NT	NT	NT	NT	NT	NT
**Metals**		**Mean**	**SD**	**Mean**	**SD**	**Mean**	**SD**	**Mean**	**SD**	**Mean**	**SD**
**Arsenic**	ng/collection	BLOD	NA	BLOD	NA	BLOQ	NA	BLOD	NA	BLOD	NA
**Beryllium**	ng/collection	BLOD	NA	BLOD	NA	BLOD	NA	BLOD	NA	BLOD	NA
**Cadmium**	ng/collection	BLOD	NA	BLOD	NA	BLOD	NA	BLOD	NA	BLOD	NA
**Chromium**	ng/collection	BLOD	NA	BLOD	NA	BLOQ	NA	BLOD	NA	BLOD	NA
**Cobalt**	ng/collection	BLOD	NA	BLOD	NA	BLOD	NA	BLOD	NA	BLOD	NA
**Copper**	ng/collection	BLOD	NA	BLOD	NA	BLOD	NA	BLOD	NA	BLOD	NA
**Gold**	ng/collection	BLOD	NA	BLOD	NA	BLOD	NA	BLOD	NA	BLOD	NA
**Iron**	ng/collection	BLOD	NA	BLOD	NA	BLOD	NA	BLOD	NA	BLOD	NA
**Lead**	ng/collection	BLOD	NA	BLOD	NA	BLOD	NA	BLOD	NA	BLOD	NA
**Nickel**	ng/collection	BLOD	NA	BLOD	NA	BLOD	NA	BLOD	NA	BLOD	NA
**Selenium**	ng/collection	BLOQ	NA	BLOQ	NA	BLOQ	NA	BLOQ	NA	BLOQ	NA
**Silver**	ng/collection	BLOD	NA	BLOD	NA	BLOD	NA	BLOD	NA	BLOD	NA
**Tin**	ng/collection	BLOD	NA	BLOQ	NA	BLOQ	NA	BLOQ	NA	BLOD	NA
**Zinc**	ng/collection	BLOD	NA	BLOD	NA	BLOD	NA	BLOD	NA	BLOD	NA
**Organic Acids**		**Mean**	**SD**	**Mean**	**SD**	**Mean**	**SD**	**Mean**	**SD**	**Mean**	**SD**
**Benzoic Acid**	mg/collection	11.63	1.17	11.39	0.82	9.19	1.25	12.69	1.02	9.81	0.87
**Propionic Acid**	mcg/collection	BLOD	NA	BLOD	NA	BLOD	NA	BLOD	NA	BLOD	NA
**VOCs**		**Mean**	**SD**	**Mean**	**SD**	**Mean**	**SD**	**Mean**	**SD**	**Mean**	**SD**
**1,3-butadiene**	mcg/collection	BLOD	NA	BLOD	NA	NT	NT	NT	NT	NT	NT
**Acrylonitrile**	mcg/collection	BLOD	NA	BLOD	NA	NT	NT	NT	NT	NT	NT
**Benzene**	mcg/collection	BLOQ	NA	BLOQ	NA	NT	NT	NT	NT	NT	NT
**Isoprene**	mcg/collection	BLOD	NA	BLOD	NA	NT	NT	NT	NT	NT	NT
**Propylene Oxide**	mcg/collection	BLOD	NA	BLOD	NA	NT	NT	NT	NT	NT	NT
**Toluene**	mcg/collection	BLOQ	NA	BLOQ	NA	NT	NT	NT	NT	NT	NT
**Esters & Alcohols**		**Mean**	**SD**	**Mean**	**SD**	**Mean**	**SD**	**Mean**	**SD**	**Mean**	**SD**
**1-Butanol**	mcg/collection	BLOQ	NA	BLOQ	NA	NT	NT	NT	NT	NT	NT
**Benzyl Acetate**	mcg/collection	BLOD	NA	BLOD	NA	NT	NT	NT	NT	NT	NT

**Ethyl Acetate**	mcg/collection	BLOD	NA	BLOD	NA	NT	NT	NT	NT	NT	NT
**Ethyl Acetoacetate**	mcg/collection	BLOD	NA	BLOD	NA	NT	NT	NT	NT	NT	NT
**Isoamyl Acetate**	mcg/collection	BLOD	NA	BLOD	NA	NT	NT	NT	NT	NT	NT
**Isobutyl Acetate**	mcg/collection	BLOD	NA	BLOD	NA	NT	NT	NT	NT	NT	NT
**Methyl Acetate**	mcg/collection	BLOD	NA	BLOD	NA	NT	NT	NT	NT	NT	NT

1.9 Ruby Menthol 18 mg/mL Non-Intense Per Collection Analytical Stability Results

Parameter/ Constituent	Units	T0		T3		T6		T9		T12	
**# of puffs**	NA	130		127		135		124		124	
**pH**	NA	5.90	0.08	5.60	0.03	5.90	0.04	5.70	0.05	5.62	0.06
**Primary Constituents**		**Mean**	**SD**	**Mean**	**SD**	**Mean**	**SD**	**Mean**	**SD**	**Mean**	**SD**
**Nicotine**	mg/collection	16.55	0.62	15.88	0.55	17.95	1.17	17.94	0.95	16.39	0.70
**Glycerol**	mg/collection	428.33	15.40	412.38	16.18	436.34	19.79	436.41	14.14	402.59	16.93
**Propylene Glycol**	mg/collection	578.80	22.62	552.64	19.36	587.70	40.37	582.89	30.95	558.46	22.81
**Water**	mg/collection	36.69	2.03	66.21	2.19	68.56	6.73	68.45	2.24	134.36	6.11
**Menthol**	mg/collection	9.49	0.38	8.75	0.32	9.57	0.64	9.63	0.53	8.58	0.34
**Diethylene Glycol**	mg/collection	BLOD	NA	BLOD	NA	BLOD	NA	BLOD	NA	BLOD	NA
**Ethylene Glycol**	mg/collection	BLOD	NA	BLOD	NA	BLOD	NA	BLOD	NA	BLOD	NA
**Carbonyls & Glycidol**		**Mean**	**SD**	**Mean**	**SD**	**Mean**	**SD**	**Mean**	**SD**	**Mean**	**SD**
**Acetaldehyde**	mcg/collection	3.49	0.33	3.52	0.66	5.27	0.95	5.09	0.61	4.67	0.97
**Acrolein**	mcg/collection	BLOQ	NA	BLOQ	NA	BLOQ	NA	BLOQ	NA	BLOQ	NA
**Diacetyl**	mcg/collection	BLOQ	NA	BLOQ	NA	BLOQ	NA	BLOQ	NA	BLOQ	NA
**Formaldehyde**	mcg/collection	9.73	0.66	8.48	1.10	11.48	1.52	12.42	1.32	13.40	1.63
**Glycidol**	mcg/collection	2.05	0.56	2.55	0.20	2.53	0.23	2.38	0.16	3.02	0.29
**Acetyl Propionyl**	mcg/collection	BLOD	NA	BLOD	NA	BLOD	NA	BLOD	NA	BLOD	NA
**Butyraldehyde**	mcg/collection	BLOD	NA	BLOD	NA	BLOD	NA	BLOD	NA	BLOD	NA

**Crotonaldehyde**	mcg/collection	BLOD	NA	BLOD	NA	BLOD	NA	BLOD	NA	BLOD	NA
**Furfural**	mcg/collection	BLOD	NA	BLOD	NA	BLOD	NA	BLOD	NA	BLOD	NA
**Nicotine Degradants & TSNAs**		**Mean**	**SD**	**Mean**	**SD**	**Mean**	**SD**	**Mean**	**SD**	**Mean**	**SD**
**β-Nicotyrine**	mcg/collection	1.50	0.12	1.49	0.77	3.72	1.63	3.00	1.20	2.65	1.54
**Cotinine**	mcg/collection	1.04	0.14	BLOQ	NA	2.21	0.44	4.35	1.02	2.56	0.55
**Myosmine**	mcg/collection	2.01	0.15	3.12	0.61	4.67	0.77	5.21	0.70	4.94	0.81
**Nicotine N Oxide**	mcg/collection	0.98	0.41	1.07	1.40	0.91	0.33	4.60	2.41	BLOQ	NA
**Nornicotine**	mcg/collection	2.99	0.36	6.00	2.80	8.00	3.59	11.99	5.34	10.48	6.22
**Anabasine**	mcg/collection	BLOD	NA	BLOD	NA	BLOD	NA	BLOD	NA	BLOD	NA
**Anatabine**	mcg/collection	BLOD	NA	BLOD	NA	BLOD	NA	BLOD	NA	BLOD	NA
**NNK**	mcg/collection	BLOQ	NA	BLOQ	NA	NT	NT	NT	NT	NT	NT
**NNN**	mcg/collection	0.01	0.0005	BLOQ	NA	NT	NT	NT	NT	NT	NT
**Metals**		**Mean**	**SD**	**Mean**	**SD**	**Mean**	**SD**	**Mean**	**SD**	**Mean**	**SD**
**Arsenic**	ng/collection	BLOD	NA	BLOD	NA	BLOD	NA	BLOD	NA	BLOD	NA
**Beryllium**	ng/collection	BLOD	NA	BLOD	NA	BLOD	NA	BLOD	NA	BLOD	NA
**Cadmium**	ng/collection	BLOD	NA	BLOD	NA	BLOD	NA	BLOD	NA	BLOD	NA
**Chromium**	ng/collection	BLOD	NA	BLOD	NA	BLOD	NA	BLOD	NA	BLOD	NA
**Cobalt**	ng/collection	BLOD	NA	BLOD	NA	BLOD	NA	BLOD	NA	BLOD	NA
**Copper**	ng/collection	BLOD	NA	BLOD	NA	BLOD	NA	BLOD	NA	BLOD	NA
**Gold**	ng/collection	BLOD	NA	BLOD	NA	BLOD	NA	BLOD	NA	BLOD	NA
**Iron**	ng/collection	BLOD	NA	BLOD	NA	BLOD	NA	BLOD	NA	BLOD	NA
**Lead**	ng/collection	BLOD	NA	BLOD	NA	BLOD	NA	BLOD	NA	BLOD	NA
**Nickel**	ng/collection	BLOD	NA	BLOD	NA	BLOD	NA	BLOD	NA	BLOD	NA
**Selenium**	ng/collection	BLOQ	NA	BLOQ	NA	BLOQ	NA	BLOQ	NA	BLOQ	NA
**Silver**	ng/collection	BLOD	NA	BLOD	NA	BLOQ	NA	BLOD	NA	BLOD	NA
**Tin**	ng/collection	BLOD	NA	BLOD	NA	BLOD	NA	BLOD	NA	BLOD	NA
**Zinc**	ng/collection	BLOD	NA	BLOD	NA	BLOD	NA	BLOD	NA	BLOD	NA
**Organic Acids**		**Mean**	**SD**	**Mean**	**SD**	**Mean**	**SD**	**Mean**	**SD**	**Mean**	**SD**
**Benzoic Acid**	mg/collection	8.33	0.89	11.42	0.78	11.53	0.81	11.98	0.92	9.37	0.69

**Propionic Acid**	mcg/collection	BLOD	NA	BLOD	NA	BLOD	NA	BLOD	NA	BLOD	NA
**VOCs**		**Mean**	**SD**	**Mean**	**SD**	**Mean**	**SD**	**Mean**	**SD**	**Mean**	**SD**
**1,3-butadiene**	mcg/collection	BLOD	NA	BLOD	NA	NT	NT	NT	NT	NT	NT
**Acrylonitrile**	mcg/collection	BLOD	NA	BLOD	NA	NT	NT	NT	NT	NT	NT
**Benzene**	mcg/collection	BLOQ	NA	BLOQ	NA	NT	NT	NT	NT	NT	NT
**Isoprene**	mcg/collection	BLOD	NA	BLOD	NA	NT	NT	NT	NT	NT	NT
**Propylene Oxide**	mcg/collection	BLOD	NA	BLOD	NA	NT	NT	NT	NT	NT	NT
**Toluene**	mcg/collection	BLOQ	NA	BLOD	NA	NT	NT	NT	NT	NT	NT
**Esters & Alcohols**		**Mean**	**SD**	**Mean**	**SD**	**Mean**	**SD**	**Mean**	**SD**	**Mean**	**SD**
**1-Butanol**	mcg/collection	BLOD	NA	BLOD	NA	NT	NT	NT	NT	NT	NT
**Benzyl Acetate**	mcg/collection	BLOD	NA	BLOD	NA	NT	NT	NT	NT	NT	NT
**Ethyl Acetate**	mcg/collection	BLOD	NA	BLOD	NA	NT	NT	NT	NT	NT	NT
**Ethyl Acetoacetate**	mcg/collection	BLOD	NA	BLOD	NA	NT	NT	NT	NT	NT	NT
**Isoamyl Acetate**	mcg/collection	BLOD	NA	BLOD	NA	NT	NT	NT	NT	NT	NT
**Isobutyl Acetate**	mcg/collection	BLOD	NA	BLOD	NA	NT	NT	NT	NT	NT	NT
**Methyl Acetate**	mcg/collection	BLOD	NA	BLOQ	NA	NT	NT	NT	NT	NT	NT

1.10 Ruby Menthol 18 mg/mL Intense Per Collection Analytical Stability Results

Parameter/ Constituent	Units	T0		T3		T6		T9		T12	
**# of puffs**	NA	80		76		79		85		73	
**pH**	NA	6.09	0.14	5.96	0.09	5.89	0.13	5.61	0.05	5.68	0.08
**Primary Constituents**		**Mean**	**SD**	**Mean**	**SD**	**Mean**	**SD**	**Mean**	**SD**	**Mean**	**SD**
**Nicotine**	mg/collection	14.61	1.42	15.00	1.25	16.77	1.44	18.70	1.11	15.87	1.34
**Glycerol**	mg/collection	382.94	37.90	387.76	32.23	416.76	27.33	444.51	13.76	393.29	32.54
**Propylene Glycol**	mg/collection	511.76	52.76	522.18	44.49	540.31	47.07	602.83	33.68	545.58	47.37
**Water**	mg/collection	43.34	2.67	57.91	1.74	67.49	4.84	72.39	2.59	98.16	3.31

**Menthol**	mg/collection	8.15	0.87	8.21	0.65	8.89	0.83	9.67	0.59	8.25	0.79
**Diethylene Glycol**	mg/collection	BLOD	NA	BLOD	NA	BLOD	NA	BLOD	NA	BLOD	NA
**Ethylene Glycol**	mg/collection	BLOD	NA	BLOD	NA	BLOD	NA	BLOD	NA	BLOD	NA
**Carbonyls & Glycidol**		**Mean**	**SD**	**Mean**	**SD**	**Mean**	**SD**	**Mean**	**SD**	**Mean**	**SD**
**Acetaldehyde**	mcg/collection	3.81	0.44	4.45	0.62	5.42	0.73	6.76	0.77	4.72	0.84
**Acrolein**	mcg/collection	1.82	0.55	BLOQ	NA	1.14	0.36	BLOQ	NA	BLOQ	NA
**Diacetyl**	mcg/collection	BLOQ	NA	BLOQ	NA	BLOQ	NA	BLOQ	NA	BLOQ	NA
**Formaldehyde**	mcg/collection	10.34	1.13	11.16	0.97	11.64	1.08	15.57	1.22	14.77	1.72
**Glycidol**	mcg/collection	4.69	1.20	3.01	0.88	3.73	0.84	3.15	0.39	4.82	0.78
**Acetyl Propionyl**	mcg/collection	BLOD	NA	BLOD	NA	BLOD	NA	BLOD	NA	BLOD	NA
**Butyraldehyde**	mcg/collection	BLOD	NA	BLOD	NA	BLOD	NA	BLOD	NA	BLOD	NA
**Crotonaldehyde**	mcg/collection	BLOD	NA	BLOD	NA	BLOD	NA	BLOD	NA	BLOD	NA
**Furfural**	mcg/collection	BLOD	NA	BLOD	NA	BLOD	NA	BLOD	NA	BLOD	NA
**Nicotine Degradants & TSNAs**		**Mean**	**SD**	**Mean**	**SD**	**Mean**	**SD**	**Mean**	**SD**	**Mean**	**SD**
**β-Nicotyrine**	mcg/collection	BLOQ	NA	1.95	0.65	4.08	1.35	5.50	1.92	4.53	1.22
**Cotinine**	mcg/collection	1.08	0.36	BLOQ	NA	2.08	0.26	4.95	1.46	2.67	0.32
**Myosmine**	mcg/collection	1.77	0.25	3.47	0.59	4.55	0.73	6.01	0.40	4.81	0.51
**Nicotine N Oxide**	mcg/collection	BLOQ	NA	BLOQ	NA	BLOQ	NA	4.40	2.59	BLOQ	NA
**Nornicotine**	mcg/collection	2.81	0.47	5.10	1.69	8.14	2.94	16.57	2.60	11.66	3.38
**Anabasine**	mcg/collection	BLOD	NA	BLOD	NA	BLOD	NA	BLOD	NA	BLOD	NA
**Anatabine**	mcg/collection	BLOD	NA	BLOD	NA	BLOD	NA	BLOD	NA	BLOD	NA
**NNK**	mcg/collection	BLOQ	NA	BLOQ	NA	NT	NT	NT	NT	NT	NT
**NNN**	mcg/collection	0.01	0.0005	0.004	0.0003	NT	NT	NT	NT	NT	NT
**Metals**		**Mean**	**SD**	**Mean**	**SD**	**Mean**	**SD**	**Mean**	**SD**	**Mean**	**SD**
**Arsenic**	ng/collection	BLOD	NA	BLOD	NA	BLOD	NA	BLOD	NA	BLOD	NA
**Beryllium**	ng/collection	BLOD	NA	BLOD	NA	BLOD	NA	BLOD	NA	BLOD	NA
**Cadmium**	ng/collection	BLOD	NA	BLOD	NA	BLOD	NA	BLOD	NA	BLOD	NA
**Chromium**	ng/collection	BLOD	NA	BLOD	NA	BLOD	NA	BLOD	NA	BLOD	NA
**Cobalt**	ng/collection	BLOD	NA	BLOD	NA	BLOD	NA	BLOD	NA	BLOD	NA

**Copper**	ng/collection	BLOD	NA	BLOD	NA	BLOD	NA	BLOD	NA	BLOD	NA
**Gold**	ng/collection	BLOD	NA	BLOD	NA	BLOD	NA	BLOD	NA	BLOD	NA
**Iron**	ng/collection	BLOD	NA	BLOD	NA	BLOD	NA	BLOD	NA	BLOD	NA
**Lead**	ng/collection	BLOD	NA	BLOQ	NA	BLOD	NA	BLOD	NA	BLOD	NA
**Nickel**	ng/collection	BLOD	NA	BLOD	NA	BLOD	NA	BLOD	NA	BLOD	NA
**Selenium**	ng/collection	BLOQ	NA	BLOQ	NA	BLOQ	NA	BLOQ	NA	BLOQ	NA
**Silver**	ng/collection	BLOD	NA	BLOD	NA	BLOQ	NA	BLOD	NA	BLOD	NA
**Tin**	ng/collection	BLOD	NA	BLOQ	NA	BLOD	NA	BLOD	NA	BLOD	NA
**Zinc**	ng/collection	BLOD	NA	BLOD	NA	BLOD	NA	BLOD	NA	BLOD	NA
**Organic Acids**		**Mean**	**SD**	**Mean**	**SD**	**Mean**	**SD**	**Mean**	**SD**	**Mean**	**SD**
**Benzoic Acid**	mg/collection	11.72	1.61	11.91	0.69	10.47	1.41	13.93	1.98	8.83	0.88
**Propionic Acid**	mcg/collection	BLOD	NA	BLOD	NA	BLOD	NA	BLOD	NA	BLOD	NA
**VOCs**		**Mean**	**SD**	**Mean**	**SD**	**Mean**	**SD**	**Mean**	**SD**	**Mean**	**SD**
**1,3-butadiene**	mcg/collection	BLOD	NA	BLOD	NA	NT	NT	NT	NT	NT	NT
**Acrylonitrile**	mcg/collection	BLOD	NA	BLOD	NA	NT	NT	NT	NT	NT	NT
**Benzene**	mcg/collection	BLOQ	NA	BLOQ	NA	NT	NT	NT	NT	NT	NT
**Isoprene**	mcg/collection	BLOD	NA	BLOD	NA	NT	NT	NT	NT	NT	NT
**Propylene Oxide**	mcg/collection	BLOD	NA	BLOD	NA	NT	NT	NT	NT	NT	NT
**Toluene**	mcg/collection	BLOQ	NA	BLOQ	NA	NT	NT	NT	NT	NT	NT
**Esters & Alcohols**		**Mean**	**SD**	**Mean**	**SD**	**Mean**	**SD**	**Mean**	**SD**	**Mean**	**SD**
**1-Butanol**	mcg/collection	BLOD	NA	BLOD	NA	NT	NT	NT	NT	NT	NT
**Benzyl Acetate**	mcg/collection	BLOD	NA	BLOD	NA	NT	NT	NT	NT	NT	NT
**Ethyl Acetate**	mcg/collection	BLOD	NA	BLOD	NA	NT	NT	NT	NT	NT	NT
**Ethyl Acetoacetate**	mcg/collection	BLOD	NA	BLOD	NA	NT	NT	NT	NT	NT	NT
**Isoamyl Acetate**	mcg/collection	BLOD	NA	BLOD	NA	NT	NT	NT	NT	NT	NT
**Isobutyl Acetate**	mcg/collection	BLOD	NA	BLOD	NA	NT	NT	NT	NT	NT	NT
**Methyl Acetate**	mcg/collection	BLOD	NA	BLOQ	NA	NT	NT	NT	NT	NT	NT

1.11 Summer Menthol 18 mg/mL Per Collection Analytical Stability Results

Parameter/ Constituent	Units	T0		T3		T6		T9		T12	
**# of puffs**	NA	126		127		138		127		117	
**pH**	NA	6.42	0.03	6.31	0.02	6.46	0.06	6.29	0.03	6.28	0.03
**Primary Constituents**		**Mean**	**SD**	**Mean**	**SD**	**Mean**	**SD**	**Mean**	**SD**	**Mean**	**SD**
**Nicotine**	mg/collection	16.77	0.67	16.14	0.93	18.64	1.23	18.95	0.40	15.84	0.89
**Glycerol**	mg/collection	431.72	16.21	414.91	26.39	441.48	22.38	441.26	4.57	383.93	20.92
**Propylene Glycol**	mg/collection	560.18	22.80	535.87	30.67	582.59	40.60	583.44	11.70	514.03	29.23
**Water**	mg/collection	41.89	2.28	69.54	3.28	80.53	14.76	79.41	2.22	132.76	6.10
**Menthol**	mg/collection	9.48	0.39	8.76	0.50	9.77	0.69	9.97	0.30	8.28	0.49
**Diethylene Glycol**	mg/collection	BLOD	NA	BLOD	NA	BLOD	NA	BLOD	NA	BLOD	NA
**Ethylene Glycol**	mg/collection	BLOD	NA	BLOD	NA	BLOD	NA	BLOD	NA	BLOD	NA
**Carbonyls & Glycidol**		**Mean**	**SD**	**Mean**	**SD**	**Mean**	**SD**	**Mean**	**SD**	**Mean**	**SD**
**Acetaldehyde**	mcg/collection	1.28	0.09	1.53	0.11	1.77	0.11	1.73	0.23	1.78	0.65
**Acrolein**	mcg/collection	BLOQ	NA	BLOQ	NA	BLOQ	NA	BLOQ	NA	BLOQ	NA
**Diacetyl**	mcg/collection	BLOD	NA	BLOQ	NA	BLOD	NA	BLOQ	NA	BLOQ	NA
**Formaldehyde**	mcg/collection	8.22	0.42	6.45	0.28	7.94	0.34	7.72	0.32	8.53	1.28
**Glycidol**	mcg/collection	1.83	0.20	1.88	0.45	1.87	0.44	1.77	0.33	1.77	0.22
**Acetyl Propionyl**	mcg/collection	BLOD	NA	BLOD	NA	BLOD	NA	BLOQ	NA	BLOD	NA
**Butyraldehyde**	mcg/collection	BLOD	NA	BLOD	NA	BLOD	NA	BLOD	NA	BLOD	NA
**Crotonaldehyde**	mcg/collection	BLOD	NA	BLOD	NA	BLOD	NA	BLOD	NA	BLOD	NA
**Furfural**	mcg/collection	BLOD	NA	BLOD	NA	BLOD	NA	BLOD	NA	BLOD	NA
**Nicotine Degradants & TSNAs**		**Mean**	**SD**	**Mean**	**SD**	**Mean**	**SD**	**Mean**	**SD**	**Mean**	**SD**
**β-Nicotyrine**	mcg/collection	BLOQ	NA	BLOQ	NA	1.47	0.10	1.03	0.15	BLOQ	NA
**Cotinine**	mcg/collection	BLOQ	NA	BLOD	NA	BLOQ	NA	2.23	0.92	BLOQ	NA
**Myosmine**	mcg/collection	BLOQ	NA	1.44	0.05	1.58	0.13	1.81	0.20	1.80	0.65
**Nicotine N Oxide**	mcg/collection	1.62	0.82	BLOQ	NA	0.94	0.20	3.52	1.40	BLOQ	NA

**Nornicotine**	mcg/collection	2.35	0.14	4.12	0.38	5.74	0.36	8.60	1.34	6.56	0.97
**Anabasine**	mcg/collection	BLOD	NA	BLOD	NA	BLOD	NA	BLOD	NA	BLOD	NA
**Anatabine**	mcg/collection	BLOD	NA	BLOD	NA	BLOD	NA	BLOD	NA	BLOD	NA
**NNK**	mcg/collection	BLOQ	NA	BLOQ	NA	NT	NT	NT	NT	NT	NT
**NNN**	mcg/collection	BLOD	NA	BLOD	NA	NT	NT	NT	NT	NT	NT
**Metals**		**Mean**	**SD**	**Mean**	**SD**	**Mean**	**SD**	**Mean**	**SD**	**Mean**	**SD**
**Arsenic**	ng/collection	BLOD	NA	BLOD	NA	BLOD	NA	BLOD	NA	BLOD	NA
**Beryllium**	ng/collection	BLOD	NA	BLOD	NA	BLOD	NA	BLOD	NA	BLOD	NA
**Cadmium**	ng/collection	BLOD	NA	BLOD	NA	BLOD	NA	BLOD	NA	BLOD	NA
**Chromium**	ng/collection	BLOD	NA	BLOD	NA	BLOD	NA	BLOD	NA	BLOD	NA
**Cobalt**	ng/collection	BLOD	NA	BLOD	NA	BLOD	NA	BLOD	NA	BLOD	NA
**Copper**	ng/collection	BLOD	NA	BLOD	NA	BLOD	NA	BLOD	NA	BLOD	NA
**Gold**	ng/collection	BLOD	NA	BLOD	NA	BLOD	NA	BLOD	NA	BLOD	NA
**Iron**	ng/collection	BLOD	NA	BLOD	NA	BLOD	NA	BLOD	NA	BLOD	NA
**Lead**	ng/collection	BLOD	NA	BLOD	NA	BLOD	NA	BLOD	NA	BLOD	NA
**Nickel**	ng/collection	BLOD	NA	BLOD	NA	BLOD	NA	BLOD	NA	BLOD	NA
**Selenium**	ng/collection	BLOQ	NA	BLOQ	NA	BLOQ	NA	BLOD	NA	BLOD	NA
**Silver**	ng/collection	BLOD	NA	BLOD	NA	BLOQ	NA	BLOD	NA	BLOD	NA
**Tin**	ng/collection	BLOD	NA	BLOD	NA	BLOD	NA	BLOD	NA	BLOD	NA
**Zinc**	ng/collection	BLOQ	NA	BLOD	NA	BLOD	NA	BLOD	NA	BLOD	NA
**Organic Acids**		**Mean**	**SD**	**Mean**	**SD**	**Mean**	**SD**	**Mean**	**SD**	**Mean**	**SD**
**Benzoic Acid**	mg/collection	8.41	1.01	11.09	0.75	10.87	0.79	12.89	1.36	9.13	1.08
**Propionic Acid**	mcg/collection	BLOD	NA	BLOD	NA	BLOD	NA	BLOD	NA	BLOD	NA
**VOCs**		**Mean**	**SD**	**Mean**	**SD**	**Mean**	**SD**	**Mean**	**SD**	**Mean**	**SD**
**1,3-butadiene**	mcg/collection	BLOD	NA	BLOD	NA	NT	NT	NT	NT	NT	NT
**Acrylonitrile**	mcg/collection	BLOD	NA	BLOD	NA	NT	NT	NT	NT	NT	NT
**Benzene**	mcg/collection	BLOQ	NA	BLOQ	NA	NT	NT	NT	NT	NT	NT
**Isoprene**	mcg/collection	BLOD	NA	BLOD	NA	NT	NT	NT	NT	NT	NT

**Propylene Oxide**	mcg/collection	BLOD	NA	BLOD	NA	NT	NT	NT	NT	NT	NT
**Toluene**	mcg/collection	BLOQ	NA	BLOD	NA	NT	NT	NT	NT	NT	NT
**Esters & Alcohols**		**Mean**	**SD**	**Mean**	**SD**	**Mean**	**SD**	**Mean**	**SD**	**Mean**	**SD**
**1-Butanol**	mcg/collection	BLOD	NA	BLOD	NA	NT	NT	NT	NT	NT	NT
**Benzyl Acetate**	mcg/collection	BLOD	NA	BLOD	NA	NT	NT	NT	NT	NT	NT
**Ethyl Acetate**	mcg/collection	170.84	18.00	130.88	12.86	NT	NT	NT	NT	NT	NT
**Ethyl Acetoacetate**	mcg/collection	BLOD	NA	BLOD	NA	NT	NT	NT	NT	NT	NT
**Isoamyl Acetate**	mcg/collection	BLOD	NA	BLOD	NA	NT	NT	NT	NT	NT	NT
**Isobutyl Acetate**	mcg/collection	BLOD	NA	BLOD	NA	NT	NT	NT	NT	NT	NT
**Methyl Acetate**	mcg/collection	BLOD	NA	BLOQ	NA	NT	NT	NT	NT	NT	NT

1.12 Summer Menthol 18 mg/mL Intense Per Collection Per Collection Analytical Stability Results

Parameter/ Constituent	Units	T0		T3		T6		T9		T12	
**# of puffs**	NA	86		80		74		86		69	
**pH**	NA	6.40	0.06	6.51	0.05	6.40	0.11	6.25	0.07	6.29	0.04
**Primary Constituents**		**Mean**	**SD**	**Mean**	**SD**	**Mean**	**SD**	**Mean**	**SD**	**Mean**	**SD**
**Nicotine**	mg/collection	16.74	1.45	16.85	0.85	17.44	1.61	18.56	0.78	15.15	1.66
**Glycerol**	mg/collection	432.87	36.94	432.90	23.99	425.10	25.37	437.62	12.04	371.89	38.35
**Propylene Glycol**	mg/collection	562.00	51.65	561.75	27.39	539.18	47.62	572.17	23.49	495.34	57.27
**Water**	mg/collection	53.05	2.22	68.72	0.92	72.22	3.79	81.61	1.78	98.02	5.69
**Menthol**	mg/collection	9.37	0.87	9.20	0.41	9.21	0.89	9.53	0.37	7.77	0.91
**Diethylene Glycol**	mg/collection	BLOD	NA	BLOD	NA	BLOD	NA	BLOD	NA	BLOD	NA
**Ethylene Glycol**	mg/collection	BLOD	NA	BLOD	NA	BLOD	NA	BLOD	NA	BLOD	NA
**Carbonyls & Glycidol**		**Mean**	**SD**	**Mean**	**SD**	**Mean**	**SD**	**Mean**	**SD**	**Mean**	**SD**
**Acetaldehyde**	mcg/collection	1.95	0.21	1.88	0.21	1.56	0.12	2.23	0.30	BLOQ	NA
**Acrolein**	mcg/collection	2.07	0.29	BLOQ	NA	BLOQ	NA	BLOQ	NA	BLOQ	NA
**Diacetyl**	mcg/collection	BLOD	NA	BLOQ	NA	BLOQ	NA	BLOQ	NA	BLOQ	NA

**Formaldehyde**	mcg/collection	10.98	1.01	9.86	0.85	7.74	0.70	9.84	0.70	8.21	0.48
**Glycidol**	mcg/collection	3.57	0.86	3.07	0.49	2.87	0.45	2.35	1.00	3.96	0.53
**Acetyl Propionyl**	mcg/collection	BLOD	NA	BLOD	NA	BLOD	NA	BLOD	NA	BLOD	NA
**Butyraldehyde**	mcg/collection	BLOD	NA	BLOD	NA	BLOD	NA	BLOD	NA	BLOD	NA
**Crotonaldehyde**	mcg/collection	BLOD	NA	BLOD	NA	BLOD	NA	BLOD	NA	BLOD	NA
**Furfural**	mcg/collection	BLOD	NA	BLOD	NA	BLOD	NA	BLOD	NA	BLOD	NA
**Nicotine Degradants & TSNAs**		**Mean**	**SD**	**Mean**	**SD**	**Mean**	**SD**	**Mean**	**SD**	**Mean**	**SD**
**β-Nicotyrine**	mcg/collection	BLOQ	NA	BLOQ	NA	1.36	0.12	1.20	0.33	1.14	0.27
**Cotinine**	mcg/collection	BLOQ	NA	BLOD	NA	BLOQ	NA	2.29	1.23	BLOQ	NA
**Myosmine**	mcg/collection	BLOQ	NA	1.69	0.22	1.46	0.10	2.00	0.18	1.33	0.16
**Nicotine N Oxide**	mcg/collection	BLOQ	NA	BLOQ	NA	BLOQ	NA	3.19	1.93	BLOQ	NA
**Nornicotine**	mcg/collection	2.67	0.33	4.44	0.55	4.52	0.28	8.33	1.86	5.10	0.70
**Anabasine**	mcg/collection	BLOD	NA	BLOD	NA	BLOD	NA	BLOD	NA	BLOD	NA
**Anatabine**	mcg/collection	BLOD	NA	BLOD	NA	BLOD	NA	BLOD	NA	BLOD	NA
**NNK**	mcg/collection	BLOQ	NA	BLOQ	NA	NT	NT	NT	NT	NT	NT
**NNN**	mcg/collection	BLOD	NA	BLOD	NA	NT	NT	NT	NT	NT	NT
**Metals**		**Mean**	**SD**	**Mean**	**SD**	**Mean**	**SD**	**Mean**	**SD**	**Mean**	**SD**
**Arsenic**	ng/collection	BLOD	NA	BLOD	NA	BLOD	NA	BLOD	NA	BLOD	NA
**Beryllium**	ng/collection	BLOD	NA	BLOD	NA	BLOD	NA	BLOD	NA	BLOD	NA
**Cadmium**	ng/collection	BLOD	NA	BLOD	NA	BLOD	NA	BLOD	NA	BLOD	NA
**Chromium**	ng/collection	BLOD	NA	BLOD	NA	BLOD	NA	BLOD	NA	BLOD	NA
**Cobalt**	ng/collection	BLOD	NA	BLOD	NA	BLOD	NA	BLOD	NA	BLOD	NA
**Copper**	ng/collection	BLOD	NA	BLOD	NA	BLOD	NA	BLOD	NA	BLOD	NA
**Gold**	ng/collection	BLOD	NA	BLOD	NA	BLOD	NA	BLOD	NA	BLOD	NA
**Iron**	ng/collection	BLOD	NA	BLOD	NA	BLOD	NA	BLOD	NA	BLOD	NA
**Lead**	ng/collection	BLOD	NA	BLOD	NA	BLOD	NA	BLOD	NA	BLOD	NA
**Nickel**	ng/collection	BLOD	NA	BLOD	NA	BLOD	NA	BLOD	NA	BLOD	NA
**Selenium**	ng/collection	BLOQ	NA	BLOQ	NA	BLOQ	NA	BLOQ	NA	BLOQ	NA

**Silver**	ng/collection	BLOD	NA	BLOD	NA	BLOQ	NA	BLOD	NA	BLOD	NA
**Tin**	ng/collection	BLOD	NA	BLOD	NA	BLOD	NA	BLOD	NA	BLOD	NA
**Zinc**	ng/collection	BLOD	NA	BLOD	NA	BLOD	NA	BLOD	NA	BLOD	NA
**Organic Acids**		**Mean**	**SD**	**Mean**	**SD**	**Mean**	**SD**	**Mean**	**SD**	**Mean**	**SD**
**Benzoic Acid**	mg/collection	13.13	0.84	11.50	1.28	10.04	0.95	14.25	1.25	8.76	0.67
**Propionic Acid**	mcg/collection	BLOD	NA	BLOD	NA	BLOD	NA	BLOD	NA	BLOD	NA
**VOCs**		**Mean**	**SD**	**Mean**	**SD**	**Mean**	**SD**	**Mean**	**SD**	**Mean**	**SD**
**1,3-butadiene**	mcg/collection	BLOD	NA	BLOD	NA	NT	NT	NT	NT	NT	NT
**Acrylonitrile**	mcg/collection	BLOD	NA	BLOD	NA	NT	NT	NT	NT	NT	NT
**Benzene**	mcg/collection	BLOQ	NA	BLOQ	NA	NT	NT	NT	NT	NT	NT
**Isoprene**	mcg/collection	BLOD	NA	BLOD	NA	NT	NT	NT	NT	NT	NT
**Propylene Oxide**	mcg/collection	BLOD	NA	BLOD	NA	NT	NT	NT	NT	NT	NT
**Toluene**	mcg/collection	BLOQ	NA	BLOQ	NA	NT	NT	NT	NT	NT	NT
**Esters & Alcohols**		**Mean**	**SD**	**Mean**	**SD**	**Mean**	**SD**	**Mean**	**SD**	**Mean**	**SD**
**1-Butanol**	mcg/collection	BLOD	NA	BLOD	NA	NT	NT	NT	NT	NT	NT
**Benzyl Acetate**	mcg/collection	BLOD	NA	BLOD	NA	NT	NT	NT	NT	NT	NT
**Ethyl Acetate**	mcg/collection	178.52	9.38	144.40	3.17	NT	NT	NT	NT	NT	NT
**Ethyl Acetoacetate**	mcg/collection	BLOD	NA	BLOD	NA	NT	NT	NT	NT	NT	NT
**Isoamyl Acetate**	mcg/collection	BLOD	NA	BLOD	NA	NT	NT	NT	NT	NT	NT
**Isobutyl Acetate**	mcg/collection	BLOD	NA	BLOD	NA	NT	NT	NT	NT	NT	NT
**Methyl Acetate**	mcg/collection	BLOD	NA	BLOQ	NA	NT	NT	NT	NT	NT	NT
